# 1,3-Dipolar cyclisation reactions of nitriles with sterically encumbered cyclic triphosphanes: synthesis and electronic structure of phosphorus-rich heterocycles with tunable colour†

**DOI:** 10.1039/d4sc02497d

**Published:** 2024-06-19

**Authors:** Mitchell A. Nascimento, Etienne A. LaPierre, Brian O. Patrick, Jade E. T. Watson, Lara Watanabe, Jeremy Rawson, Christian Hering-Junghans, Ian Manners

**Affiliations:** a Department of Chemistry, University of Victoria 3800 Finnerty Rd Victoria British Columbia V8P 5C2 Canada elapierre@uvic.ca; b Department of Chemistry, University of British Columbia 2036 Main Mall Vancouver British Columbia V6T 1Z1 Canada; c Department of Chemistry and Biochemistry, University of Windsor 401 Sunset Avenue Windsor Ontario N9B 3P4 Canada; d Leibniz Institut für Katalyse e.V. (LIKAT) A.-Einstein-Str.3a 18059 Rostock Germany christian.hering-junghans@catalysis.de; e Institut für Chemie, Otto-von-Guericke-Universität Magdeburg, Universitätsplatz 2 39106 Magdeburg Germany

## Abstract

We describe the synthesis, solid state and electronic structures of a series of tunable five-membered cationic and charge-neutral inorganic heterocycles featuring a P_3_CN core. 1-Aza-2,3,4-triphospholenium cations [(PR)_3_N(H)CR′]^+^, [1_R_]^+^ (R′ = Me, Ph, 4-MeOC_6_H_4_, 4-CF_3_C_6_H_4_) were formed as triflate salts by the formal [3 + 2]-cyclisation reactions of strained cyclic triphosphanes (PR)_3_ (R = ^*t*^Bu, 2,4,6-Me_3_C_6_H_2_ (Mes), 2,6-^*i*^Pr_2_C_6_H_3_ (Dipp), 2,4,6-^*i*^Pr_3_C_6_H_2_ (Tipp)) with nitriles R′CN in the presence of triflic acid. The corresponding neutral free bases (PR)_3_NCR′ (2_R_) were readily obtained by subsequent deprotonation with NEt_3_. The P_3_CN cores in 2_R_ show an envelope conformation typical for cyclopentenes and present as yellow to orange compounds in the solid state as well as in solution depending on both substituents R and R′ in (PR)_3_NCR′. The P_3_CN cores in [1_R_]^+^ show a significant deviation from planarity with increasing steric bulk of the R groups at phosphorus, which results in a decrease in the HOMO–LUMO gap and distinct low-energy UV-Visible absorption bands. This allows access to colours spanning red, blue, indigo, and magenta. TD-DFT calculations provide valuable insight into this phenomenon and indicate an intramolecular charge-transfer from the HOMO located on the P_3_ framework to the N=C–R′-based LUMO in the cationic species. The cations [1_R_]^+^ represent rare examples of phosphorus-rich heterocycles with tunable colour, which can be incorporated into polymers by post-polymerization modification to afford coloured polymers, which demonstrate utility as both proton and ammonia sensors.

## Abstract 2

The acid-mediated cyclisation of sterically encumbered cyclic triphosphanes with nitriles provides access to five-membered cationic and neutral P_3_CN heterocycles with tuneable colour.

## Graphical abstract

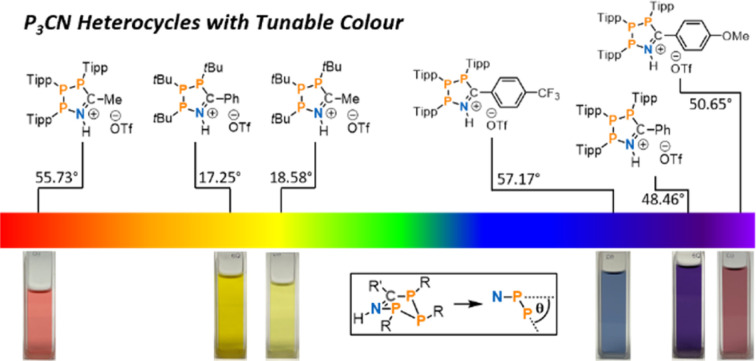

## Introduction

Decades of intensive research into catalytic and stoichiometric organic transformations have resulted in myriad routes for C–C and C–X bond formation.^1–5^ In contrast, analogous routes to bonds between main group elements are poorly developed.^6–10^ In main group chemistry reductive coupling reactions or salt metatheses continue to represent the state of the art, but often involve aggressive reaction conditions that restrict the introduction of functional groups and lead to the formation of unwanted byproducts. The adaptation of well-established organic methodologies to inorganic substrates offers the possibility of new or improved routes to molecular and polymeric inorganic species with a wide range of potential applications.^11–15^

**Scheme 1:**
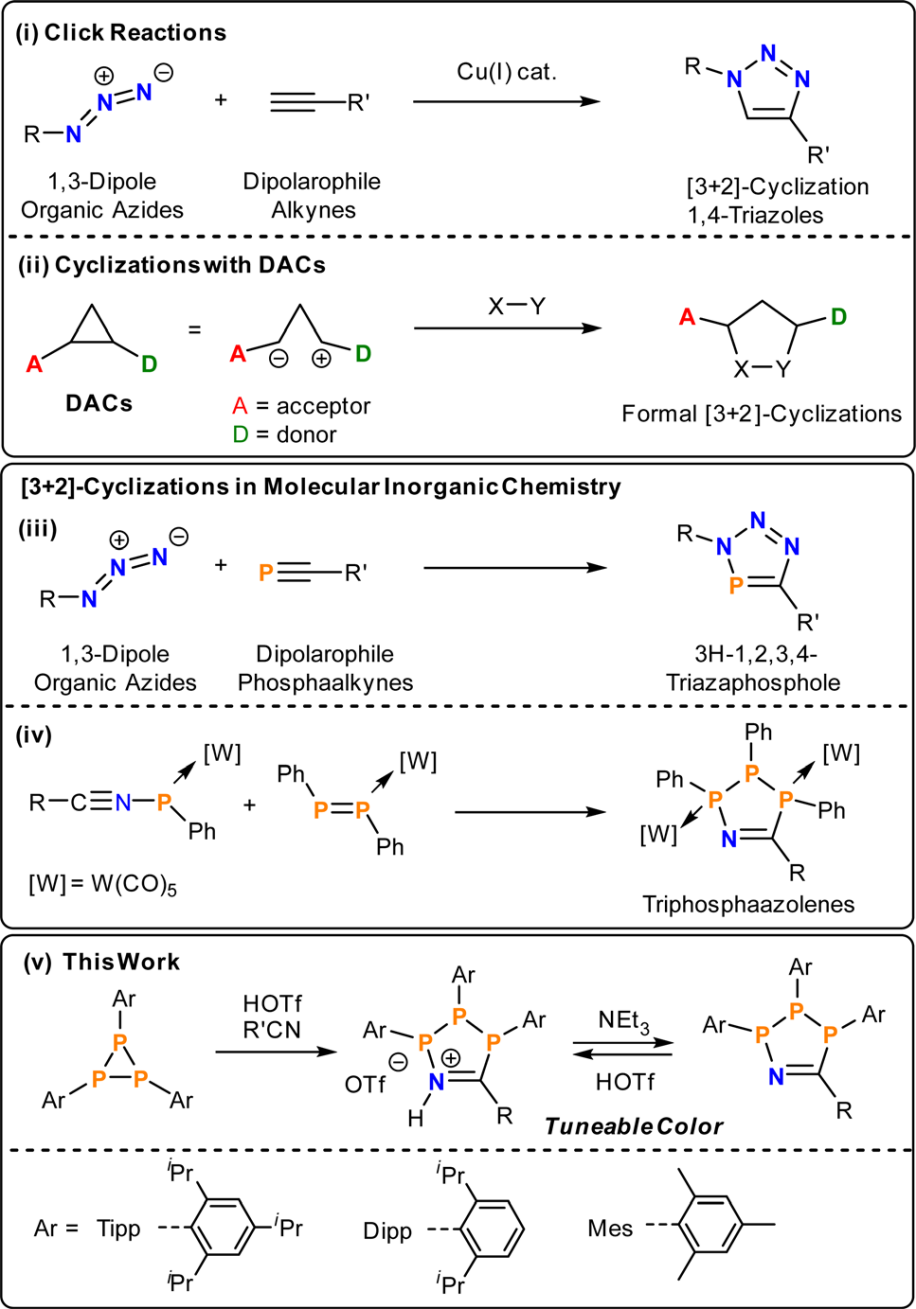
(i) Prototypical Huisgen–Sharpless cycloadditions; (ii) generalized donor–acceptor cyclopropane cycloaddition; (iii) [3 + 2]-cycloaddition between phosphaalkynes and azides; (iv) formation of P_3_CN-heterocycles in the coordination sphere of W(CO_5_); (v) summary of this study.

A common atom-economic route to organic heterocycles is the Huisgen cycloaddition of 1,3-dipoles with dipolarophiles. Huisgen first demonstrated the thermal [3 + 2]-cycloaddition of organic azides with alkynes, which proceeds without regioselectivity.^16^ Through the use of Cu-catalysts, Meldal,^17^ Sharpless and Fokin,^18^ concurrently and independently developed a regioselective process. Accordingly, a wide range of 4-triazoles can be easily accessed at room temperature (Scheme 1, i). In terms of using main-group multiply bonded systems as dipolarophiles, the formal [3 + 2]-cycloadditions with organic azides have been extended to phosphaalkynes (Scheme 1, iii),^19–22^ cyaphides,^23,24^ arsadiazoniums and arsaalkynes^25^ to give 5-membered heterocycles selectively with 100% atom-economy. Vicinal donor–acceptor cyclopropanes (DACs) represent a class of “disguised” or “masked” 1,3-dipoles. Electron donating and withdrawing groups in vicinal position polarise the C–C bond and stabilise partial positive and negative charges (Scheme 1, ii), respectively, thereby introducing significant 1,3-dipolar character.^26,27^ These DACs react with dipolarophiles to yield a plethora of organic heterocycles akin to the Huisgen–Sharpless cycloaddition. Given their utility, cyclisation reactions are an emergent methodology in phosphorus chemistry for the synthesis of inorganic ring systems.^28–34^

Triphosphiranes, cyclic phosphanes of the general formula (PR)_3_, can be considered as heavier cyclopropane analogs by isolobal replacement of CR_2_ for PR.^35^ Accordingly, triphosphiranes have a long history as starting materials for the synthesis of inorganic ring systems and the field was recently reviewed.^36^ Recent examples include phosphenium ion insertion and Lewis acid activation reactions.^37,38^ In general, the insertion of organic molecules into P–P bonds is a valuable tool for the construction of new phosphorus compounds.^39^

By analogy to DACs, the triphosphirane (^*t*^BuP)_3_ can be activated by either Brønsted (HOTf, OTf = [SO_3_CF_3_]^−^) or Lewis acids such as (Ph_3_Sb(Cl)OTf), through polarization of one P–P bond and subsequently react with nitriles R′CN to give five-membered P_3_CN-species.^40^ In the absence of HOTf or MeOTf no reaction with nitriles is observed. This methodology provides an operationally simple and rapid route to 1-aza-2,3,4-triphospholenium salts [P_3_^*t*^Bu_3_N(H)CR′]OTf and their neutral 1-aza-2,3,4-triphospholene P_3_^*t*^Bu_3_NCR′ congeners, which can be cycled between neutral and cationic states by addition of acid or base, respectively. The neutral rings were previously only accessible through formal [3 +2]-cyclisations of W(CO)_5_-coordinated diphosphenes and nitrilium phosphanylides, respectively (Scheme 1, iv).^41^ However, both the cationic [P_3_^*t*^Bu_3_N(H)CR′]^+^ as well as the neutral P_3_^*t*^Bu_3_NCR′ rings are either colourless or pale yellow and show no evidence for low-energy electronic transitions.

**Fig. 1:**
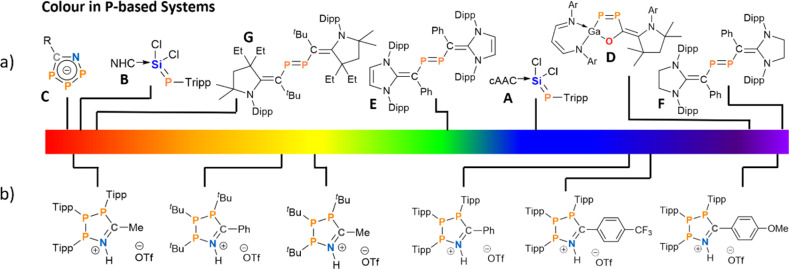
Previously reported coloured (poly-)phosphorus species (a) and coloured phosphorus species reported in this work (b), shown in line with their visible colours.

Phosphorus containing acyclic and cyclic species are of general interest as OLED emitters^42–44^ and fluorophores^45–47^ – with examples ranging from small molecule photoswitches^48^ to phosphorus analogues of common dyes.^49–51^ Phosphamethine cyanines were first reported as red crystalline solids by Dimroth in 1964, and subsequent examples exhibit colours spanning yellow, orange, and red as a function of the P-substituents and thus these species have been used as phosphorus-based dyes.^52–59^ The colour in the base-stabilised phosphasilene carbene-SiCl_2_=P-Tipp (Tipp = 2,4,6-*i*Pr_3_C_6_H_2_) ranges from deep blue, when cyclic alkyl amino carbenes (cAACs) are employed (Fig. 1a, A) or red when using the NHC IPr (IPr = (HCNDipp)_2_C, Dipp = 2,6-^*i*^Pr_2_C_6_H_3_; Fig. 1a, B).^60^ Phosphonium species^47,61,62^ and phospholes^48,61–68^ have also shown colours that include red, orange, green, blue, and purple, although these species achieve their colour on account of large polycyclic aromatic systems with appended or incorporated phosphorus centres.^69^ Phosphorus radicals, as expected, form intensely-coloured compounds across the visible spectrum as well.^70–73^ However, there are few instances of colour in closed-shell phosphorus-rich or catenated phosphorus species. Among those are deep red cyclic 1-aza-2,3,4-triphospholide anions (Fig. 1a, C),^74^ or a unique example of a five-membered GaP_2_CO species with a 1,2-diphospha-1,3-butadiene recently reported by Schulz (Fig. 1a, D).^75^

Another class of P-rich compounds are diphosphenes, which commonly present as yellow to orange solids.^76^ Introduction of N-heterocyclic vinyl (NHV) substituents at phosphorus, offer a tool to effectively tune the colour of such diphosphenes of the type (NHCCR)_2_P_2_ through extended conjugation. Colours include green when (IPr)CPh-substituents are utilized (Fig. 1a, E), or magenta when (^S^IPr)CPh-substituents (^S^IPr = (H_2_CNDipp)_2_C; Fig. 1a, F) are used.^77^ When (^Et^cAAC)C^*t*^Bu-substituents are introduced, deep red crystalline solids are obtained (Fig. 1a, G).^78^ Notably, the colour in these examples predominantly arises from π–π* transitions, and tuning the energy of absorption through distal or late-stage modification is often non-trivial.

In this contribution we show that the colour of 1-aza-2,3,4-triphospholenium salts can be effectively tuned through modification of the steric demand of the P-substituents, and through the electronic properties of the nitrile coupling partner (Fig. 1b). This is achieved by using aryl-substituted triphosphiranes (PAr)_3_ (Ar = Mes, 2,4,6-Me_3_C_6_H_2_; Dipp; Tipp, 2,4,6-^*i*^Pr_3_C_6_H_2_) as starting materials.^79^ The colouration can be traced to intramolecular charge transfer processes, and we also show switchable colour when going from the cationic to the neutral state through addition of external base. This feature was used to prepare a Brønsted acid–base responsive polymer.

## Results

### Synthesis and NMR spectroscopic characterisation of cationic and neutral P_3_CN heterocycles [1_R_]^+^ and 2_R_

**Scheme 2:**
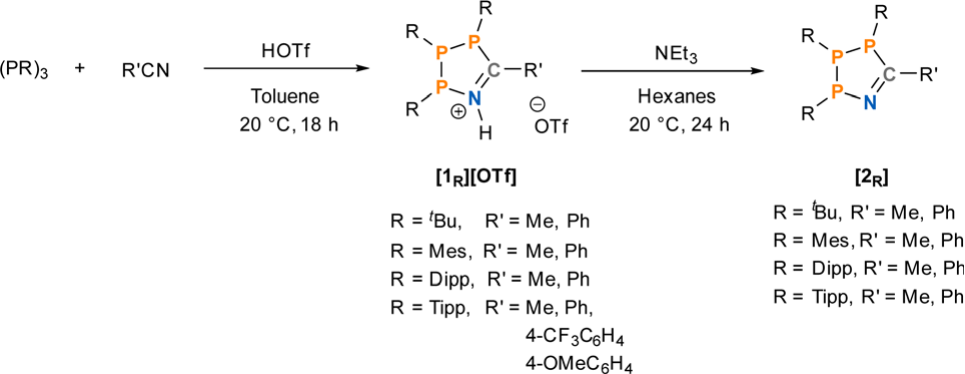
Synthesis of 1-aza-2,3,4-triphospholenium triflate salts and their corresponding free bases in this work with a labelling notation, where R describes the substitution at phosphorus and R′ describes the nitrile substituent.

**Fig. 2:**
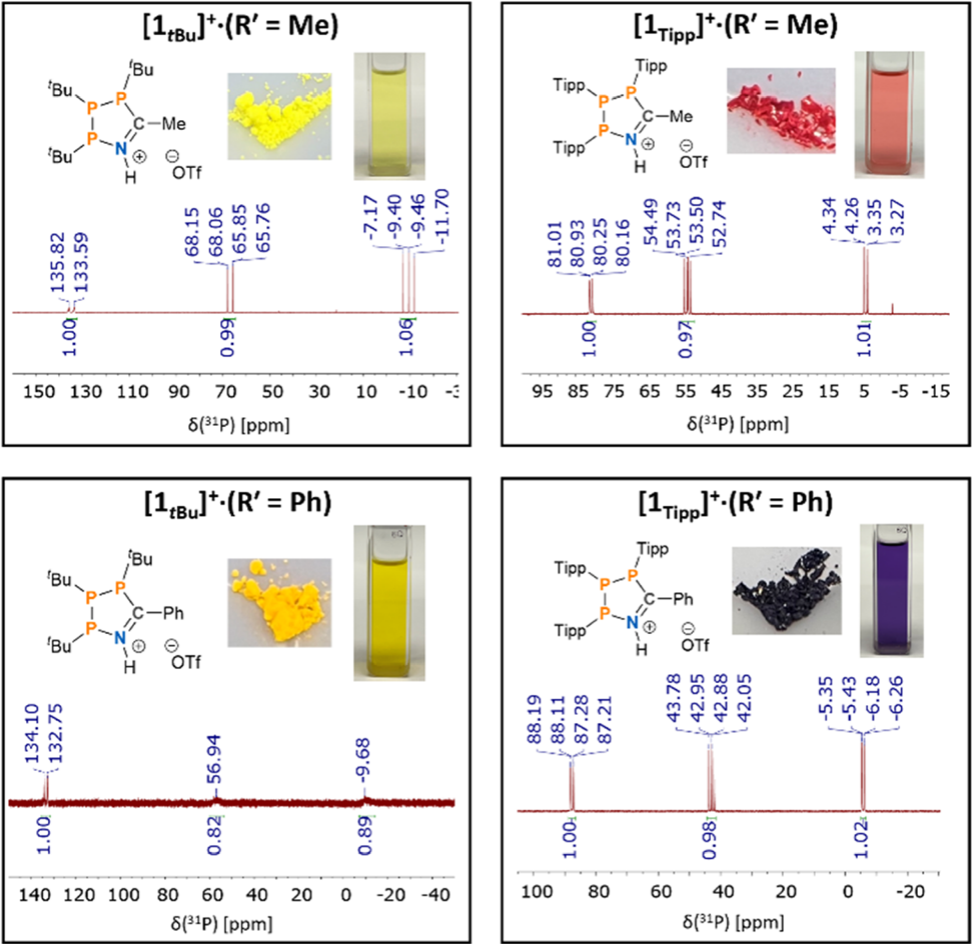
^31^P{^1^H} NMR spectra in CDCl_3_ for a comparative set of 1-aza-2,3,4-triphospholenium cations, and photographs of their [OTf]^−^ salts in the solid state and in solution (CH_2_Cl_2_).

Our investigation into the effect of steric hindrance on the Brønsted acid-mediated [3 + 2]-cyclisation of cyclic triphosphanes began with the reaction of (PTipp)_3_ with an excess of acetonitrile (MeCN) in the presence of HOTf (Scheme 2). (PTipp)_3_ was dissolved in a 1 : 1 v/v mixture of toluene/MeCN, which, after addition of one equivalent of neat HOTf at 20 °C, resulted in a deep red homogeneous solution. This colouration was unexpected, given that analogous reactions with (P^*t*^Bu)_3_ yield yellow solutions and pale-yellow solid products (Fig. 2). An aliquot of the red reaction mixture analysed by ^31^P{^1^H} NMR spectroscopy showed conversion to a single phosphorus containing species with an AMX spin system consistent with formation of the 1-aza-2,3,4-triphospholenium ring [1_Tipp_]^+^ (R′ = Me) (Fig. 2, top right).

Compared to previously synthesized [1*_t_*_Bu_]^+^ (R′ = Me) the most downfield signal in species [1_Tipp_]^+^ (R′ = Me) is shielded by approximately 52 ppm, while both *J*_PP_ coupling constants are smaller than in [1*_t_*_Bu_]^+^ (R′ = Me) by approximately 120 Hz and 70 Hz, respectively (Fig. 2, top left). Removal of the volatiles followed by washing of the tacky residue with *n*-hexane yielded a red powder. ^1^H NMR spectra in CDCl_3_ (ESI Fig. S-1†) revealed inequivalent methine environments for the Tipp substituents, as well as a characteristic doublet of doublets (*J*_HP_ = 10.2 Hz, *J*_HH_ = 4.6 Hz) corresponding to the methyl resonance of the incorporated acetonitrile-derived moiety. A broad singlet at 12.61 ppm is assigned to the H–N resonance of the protonated skeletal nitrogen atom. In the ^19^F{^1^H} NMR spectrum in CDCl_3_ (ESI Fig. S-2†) a singlet at *δ*_F_ = −78.7 ppm, indicated a non-interacting triflate anion (*cf.* [Me_3_SiN(C_6_H_10_)P(C_6_H_10_)][O_3_SCF_3_] *δ*_F_ = −78.8 ppm).^80^

Given that (PTipp)_3_ demonstrated facile MeCN insertion chemistry, we sought to extend the synthetic protocol to (PDipp)_3_ and (PMes)_3_. [1_Dipp_]^+^ (R′ = Me) was prepared similarly and was found to be virtually identical to [1_Tipp_]^+^ (R′ = Me) in colour, with similar ^31^P, ^1^H and ^19^F NMR spectroscopic characteristics (ESI Fig. S-41 to S-43†); in contrast, the attempted synthesis of [1_Mes_]^+^ (R′ = Me) was less straightforward. After conducting a similar work-up to that used for the isolation of [1_Tipp_]^+^ (R′ = Me) and [1_Dipp_]^+^ (R′ = Me), a yellow powder was obtained. Recrystallisation of the powder from PhF/*n*-pentane afforded crystals which, when dissolved in CDCl_3_ and subsequently analysed by ^31^P{^1^H} NMR spectroscopy, showed the anticipated AMX spin system, alongside impurities characterized by resonances at −59.5 ppm (singlet, *ca.* 7%) and −18.9 (two peaks, *ca.* 2%) (ESI Fig. S-70†).

A second recrystallisation of [1_Mes_]^+^ (R′ = Me) by allowing a PhF/Et_2_O solution layered with *n*-pentane to stand for a month at −30 °C resulted in a mixture of yellow prisms of [1_Mes_]^+^ (R′ = Me) and a small amount of yellow plates that were crystallographically identified as the Et_2_O solvate of (PMes)_6_ (ESI Section 5.18†). (PMes)_6_ has been previously observed as a side-product of the reaction between Na_2_[P_4_Mes_4_] with [Rh(COD)Cl]_2_ by Hey-Hawkins and coworkers by means of single-crystal X-ray crystallography.^81^ The mechanism of the formation of (PMes)_6_ is currently unknown.

Next, we synthesized the benzonitrile-derived series [1_Tipp_]^+^ (R′ = Ph), [1_Dipp_]^+^ (R′ = Ph), [1_Mes_]^+^ (R′ = Ph) and [1*_t_*_Bu_]^+^ (R′ = Ph) to provide a comparison in their properties with those of [1_R_]^+^ (R′ = Me). In this case (PTipp)_3_ was dissolved in a 2 : 1 v/v solution of toluene/PhCN, giving a colourless solution. Addition of one equivalent of neat triflic acid at 20 °C resulted in a deep blue solution. After removal of the volatiles, the dark blue residue of [1_Tipp_]^+^ (R′ = Ph) was washed with *n*-hexane to yield a blue powder, whose ^31^P{^1^H} NMR spectrum in CDCl_3_ consists of an AMX spin system, which is virtually identical to that of [1_Tipp_]^+^ (R′ = Me) (Fig. 2, bottom right). The analogous reaction was repeated with (PDipp)_3_, again yielding a blue powder of [1_Dipp_]^+^ (R′ = Ph) possessing the characteristic AMX spin system in the ^31^P{^1^H} NMR spectrum (ESI Fig. S-56†), likewise virtually identical to [1_Dipp_]^+^ (R′ = Me). In the case of [1_Mes_]^+^ (R′ = Ph), we obtained an orange powder, as opposed to the yellow [1_Mes_]^+^ (R′ = Me), that gave rise to an AXY spin system with second order effects in the ^31^P{^1^H} NMR spectrum in CDCl_3_, similar to that observed in [1_Mes_]^+^ (R′ = Me). We note that [1_Mes_]^+^ (R′ = Ph) has greater stability in solution than [1_Mes_]^+^ (R′ = Me) and that the only impurities that form upon dissolution correlate to (PMes)_4_ as evidenced by ^31^P NMR spectroscopy. Finally, [1*_t_*_Bu_]^+^ (R′ = Ph), was synthesized and isolated as a yellow powder with an AMX ^31^P{^1^H} NMR spectrum featuring broad signals (Fig. 2, bottom left).

To probe the effect of electron-donating and electron withdrawing substituents on [1_Tipp_]^+^ (R′ = Ph), *p*-MeOC_6_H_4_CN and *p*-CF_3_C_6_H_4_CN were used in place of PhCN to synthesize [1_Tipp_]^+^ (R′ = *p*-MeOC_6_H_4_) and [1_Tipp_]^+^ (R′ = *p*-CF_3_C_6_H_4_), respectively. [1_Tipp_]^+^ (R′ = *p*-MeOC_6_H_4_) formed a magenta solution with ^31^P{^1^H} NMR spectra bearing an AMX spin system in which the M and X nuclei are more shielded relative to [1_Tipp_]^+^ (R′ = Ph). [1_Tipp_]^+^ (R′ = *p*-CF_3_C_6_H_4_), contrarily, afforded royal blue solutions, with a similar AMX spin system in the ^31^P{^1^H} NMR spectrum, in which the M and X nuclei are shifted downfield relative to [1_Tipp_]^+^ (R′ = Ph). As these compounds could be potentially used as tunable P-based dyes, the photo-stability of a prototypical species was investigated. Irradiation of [1_Tipp_]^+^ (R′ = Ph) with a broad band solar irradiation LED (480 mW, see ESI Fig. S-115 and S-116† for lamp and ^31^P NMR spectra) for 72 h revealed no discernible decomposition as assayed by ^31^P{^1^H} NMR spectrometry in CDCl_3_, demonstrating the photostability of these 1-aza-2,3,4-triphospholenium salts.

Subsequently, the formation and properties of the corresponding free bases, 1-aza-2,3,4-triphospholenes 2_R_ (R′ = Me and Ph) were explored, to determine if this unexpected and intriguing colouration was unique to the protonated heterocycles. The free base 2_Tipp_ (R′ = Me) was synthesized through the addition of NEt_3_ to a slurry of [1_Tipp_]^+^ (R′ = Me) in *n*-hexane, and was isolated as a yellow solid after removal of [Et_3_NH]OTf by filtration and of volatiles *in vacuo*. The ^1^H NMR spectrum lacks the N–H signal, consistent with quantitative deprotonation. Loss of triflate, and thus formation of [HNEt_3_]OTf was indicated by the absence of detectable ^19^F NMR signals in isolated 2_Tipp_ (R′ = Me). The ^31^P{^1^H} NMR spectrum of 2_Tipp_ (R′ = Me) also consists of an AMX spin system, however, with notably shielded signals for the A and M nuclei compared to previously reported 2*_t_*_Bu_ (R′ = Me).^40^2_Dipp_ (R′ = Me) had similar colouration to 2_Tipp_ (R′ = Me), while the ^31^P{^1^H} NMR spectrum exhibits a second order AXY spin system with the A nucleus shifting downfield to 98.6 ppm compared to the chemical shift of 82.3 ppm observed for the A nucleus of 2_Tipp_ (R′ = Me). 2_Mes_ (R′ = Me) is colourless in solution and exhibits an AMX spin system distinct from that of 2_Dipp_ (R′ = Me) and 2_Tipp_ (R′ = Me). 2_Mes_ (R′ = Me) and also proved to be stable in solution, in contrast to its protonated analogue [1_Mes_]^+^ (R′ = Me), as NMR spectra collected on the solution after several weeks showed no significant change. In the case of the 2_R_ (R′ = Ph) series, no significant changes were observed in the ^31^P{^1^H} NMR chemical shifts compared to the 2_R_ (R′ = Me) series. When single crystals of 2_Mes_ (R′ = Ph) were dissolved in CDCl_3_, partial decomposition was noted, as evidenced by formation of an unknown impurity which appears as a singlet in the ^31^P{^1^H} NMR spectrum at 62.4 ppm in CDCl_3_.

Not only are the neutral 1-aza-2,3,4-triphospholenes readily synthesized through deprotonation with NEt_3_, these free bases are near quantitatively re-protonated with triflic acid to again form the 1-aza-2,3,4-triphospholenium salts [1_R_]OTf, which may again be reversibly deprotonated. This ability to reversibly convert between cationic and free base rings provides a second route to forming these heterocycles in high purity, and also provides a useful colorimetric switch (*vide infra*).

### X-ray structural characterisation of the P_3_NC heterocycles [1_R_]^+^ and 2

**Fig. 3:**
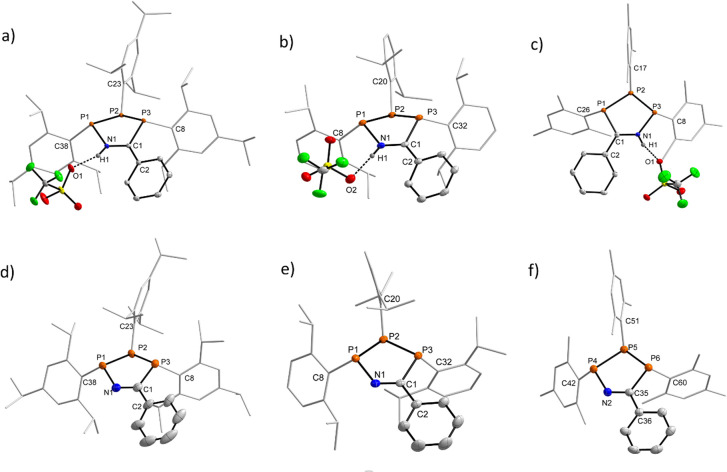
Solid-state structures of [1_Tipp_]^+^ (R′ = Ph) (a), [1_Dipp_]^+^ (R′ = Ph) (b), [1_Mes_]^+^ (R′ = Ph) (c), 2_Tipp_ (R′ = Ph) (d), 2_Dipp_ (R′ = Ph) (e), and 2_Mes_ (R′ = Ph) (f). Thermal ellipsoids are drawn at the 50% probability level. Tipp, Dipp, and Mes substituents depicted as wireframe for clarity, and all hydrogen atoms except H1 in [1_R_]^+^ have been omitted for clarity. For 2_Mes_ (R′ = Ph) only one of two independent molecules in the asymmetric unit is shown.

Characterisation of each of the aforementioned heterocycles [1_R_]^+^ and 2_R_ in the solid state was performed by single crystal X-ray diffraction (SC-XRD) experiments to investigate a potential structural rationale for the unexpected colouration. Structures, key bond lengths and angles for all species can be found in Section 5 of the ESI,† while representative structures of [1_R_]^+^ (R′ = Ph) and of [2_R_] (R′ = Ph) for R = Tipp, Dipp, and Mes are shown in Fig. 3. For this discussion, we will refer to the phosphorus atoms of the central P_3_CN framework as P_C_ (phosphorus bound to the nitrile-derived carbon atom), P_N_ (phosphorus bound to the nitrile nitrogen atom), and P_P_ for the phosphorus atom between P_C_ and P_N_. First, the down, down, up arrangement of the aryl substituents with respect to the P_3_ unit is maintained in all species when compared to (PAr)_3_. Additionally, there is a close O1–H1 contact (*d*(O–H) *ca.* 1.8 Å) between the nitrilium unit in [1_R_]^+^ and the triflate counter anion, indicative of H-bonding in the solid state, while the ^19^F NMR data clearly indicates a weakly-interacting OTf ion in solution. One feature common to the three cationic rings are minimally longer P_N_–P_P_ (R′ = Ph, [1_Tipp_]^+^ 2.2458(6), [1_Dipp_]^+^ 2.243(1), [1_Mes_]^+^ 2.238(1) Å) compared to the P_P_–P_C_ distances (R′ = Ph, [1_Tipp_]^+^ 2.2262(4), [1_Dipp_]^+^ 2.212(1), [1_Mes_]^+^ 2.206(1) Å), though all of these are within the range of typical P–P single bonds (*cf.* Σ*r*_cov_(P–P) = 2.22 Å),^82^ also when compared to triphosphirane precursor (PDipp)_3_ (*cf. d*(P–P) 2.1991(4), 2.2440(4), 2.2124(3) Å).^79^ The C–N distances are *ca.* 1.31 Å, minimally longer than an ideal double bond (*cf.* Σ*r*_cov_(C=N) = 1.27 Å),^82^ while the P_N_–N and P_C_–C distances are best described as minimally shortened single bonds. All P atoms are in a trigonal pyramidal coordination environment with the smallest sum of angles at the central P_P_ atoms (*Σ* < (P); R′ = Ph, [1_Tipp_]^+^ 296.46, [1_Dipp_]^+^ 299.69, [1_Mes_]^+^ 301.49°), with similar values for P_N_, while *Σ* < (P) at the P_C_ atom is considerably larger by *ca.* 10 °C. In the corresponding neutral species 2_R_ the P_N_–P_P_ (R′ = Ph; 2_Tipp_ 2.256(6); 2_Dipp_ 2.2725(6); 2_Mes_ 2.2496(8), 2.256(1) Å) bonds are again longer than the P_P_–P_C_ (R′ = Ph; 2_Tipp_ 2.201(1); 2_Dipp_ 2.1972(5); 2_Mes_ 2.1907(8), 2.1862(8) Å) bonds, with a greater difference when compared to the cationic rings. Upon proton abstraction the C–N distance decreases to *ca.* 1.28 Å, in line with a double bond. The P_N_–N distances shorten considerably (*d*_avg._(P_N_–N) = 1.70 Å), while the P_C_–C distances increase to an average value of *ca.* 1.86 Å. The sum of angles at the P atoms are similar to those in the cationic derivatives.

When analysing the molecular structures of all synthesized species, two distinct structural features are apparent in the Dipp and Tipp series when compared to the Mes and ^*t*^Bu series. Firstly, a clear shortening in the distance between P_N_ and P_C_ atoms in [1_Tipp_]^+^ and [1_Dipp_]^+^ (R′ = Me and Ph) species is observed, resulting in 4.5–5.5% shorter distances compared to the analogous [1*_t_*_Bu_]^+^ (R′ = Me and Ph) species (*e.g. cf.*[1_Tipp_]^+^ (R′ = Ph) 3.1149(7) Å; [1*_t_*_Bu_]^+^ (R′ = Ph) 3.2549(6) Å, see ESI Section 5.19† for full details). Conversely, the free bases show no appreciable trend in lengthening or shortening of the P_N_–P_C_ distance, indicating that this phenomenon is specific to the cationic rings bearing sterically encumbering aryl substituents.

**Fig. 4:**
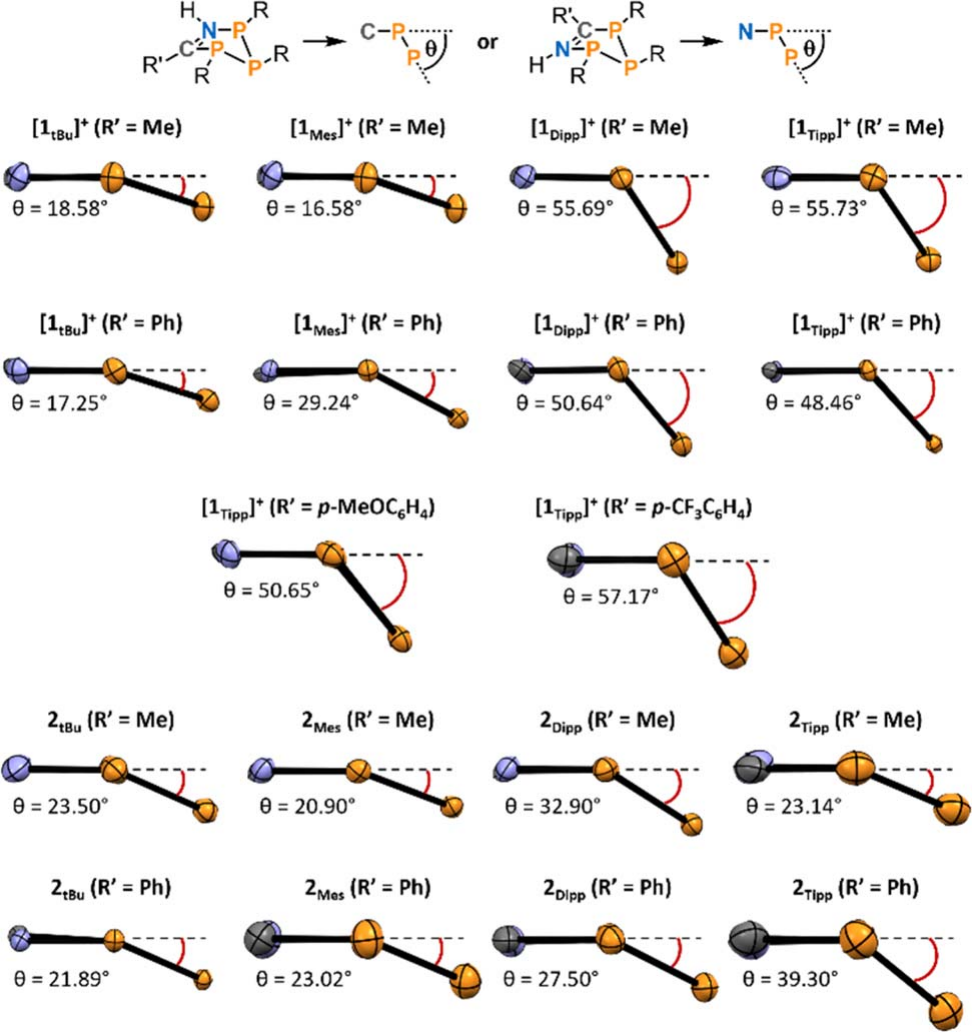
Side-on view of the central P_3_CN rings in the molecular structures as determined by SC-XRD experiments of [1_R_]^+^ and 2_R_ demonstrating the angle of bending of each species (P = orange, C = grey, N = blue). View was chosen such that the bent-out-of-plane P_P_ atom sits to the right, and all *θ* values are measured from the horizontal P_C_–C–N–P_N_ plane (represented as a dotted line parallel to the bottom of the page) downward to the plane formed by the three phosphorus atoms. Thermal ellipsoids are drawn at the 50% probability level.

Secondly, we observed a substantially increased bending in both [1_Tipp_]^+^ and [1_Dipp_]^+^ (R′ = Me and Ph) heterocycles compared to their [1*_t_*_Bu_]^+^ and [1_Mes_]^+^ congeners (Fig. 4). The angle of bending, *θ*, is measured from the horizontal P_C_–C=N–P_N_ plane downward to the plane formed by the three catenated phosphorus atoms (see top of Fig. 4).

Using this parameter, [1*_t_*_Bu_]^+^ (R′ = Me and Ph) are minimally bent, with the angles between the P_N_–P_P_–P_C_ plane and the P_N_–N=C–P_C_ plane being 18.58° and 17.25°, typical of cyclopentanes, while these angles are substantially increased to 55.73° and 48.46° in [1_Tipp_]^+^ (R′ = Me and Ph), respectively. Comparing the complete set of metrical parameters for [1_R_]^+^ (R′ = Ph) and 2_R_ (R′ = Ph), it can be seen that the change in bending angle as steric bulk increases is greater for the [1_R_]^+^·(R′ = Ph) series than in their neutral analogues. Similar structural distortions are also observed in the electronically modified compounds [1_Tipp_]^+^ (R′ = *p*-MeOC_6_H_4_, *p*-CF_3_C_6_H_4_), which display bending angles of 50.65° and 57.17°, respectively, the latter being the most bent of the series. This substantial bending is also linked to changes in the P_N_–P_C_ distance in these species, with [1_Tipp_]^+^ (R′ = *p*-MeOC_6_H_4_CN) displaying a 4.86% shortening and [1_Tipp_]^+^ (R′ = *p*-CF_3_C_6_H_4_CN) displaying a 6.76% shortening (ESI Section 5.19†).

### UV-Vis spectroscopic studies of [1_R_]^+^ and 2_R_

UV-Visible spectra were obtained in CH_2_Cl_2_ solution for each of the cyclic species to assess whether the observed data would be correlated to the steric profiles of the aryl substituents, or the characteristics of the nitrile used. Given that we were unable to successfully crystallise sufficiently pure 2_Mes_ (R′ = Me), and that [1_Mes_]^+^ (R′ = Me) and 2_Mes_·(R′ = Ph) always undergo partial decomposition in solution, even when using single crystals, UV-Vis spectra were not acquired for these species.

**Fig. 5:**
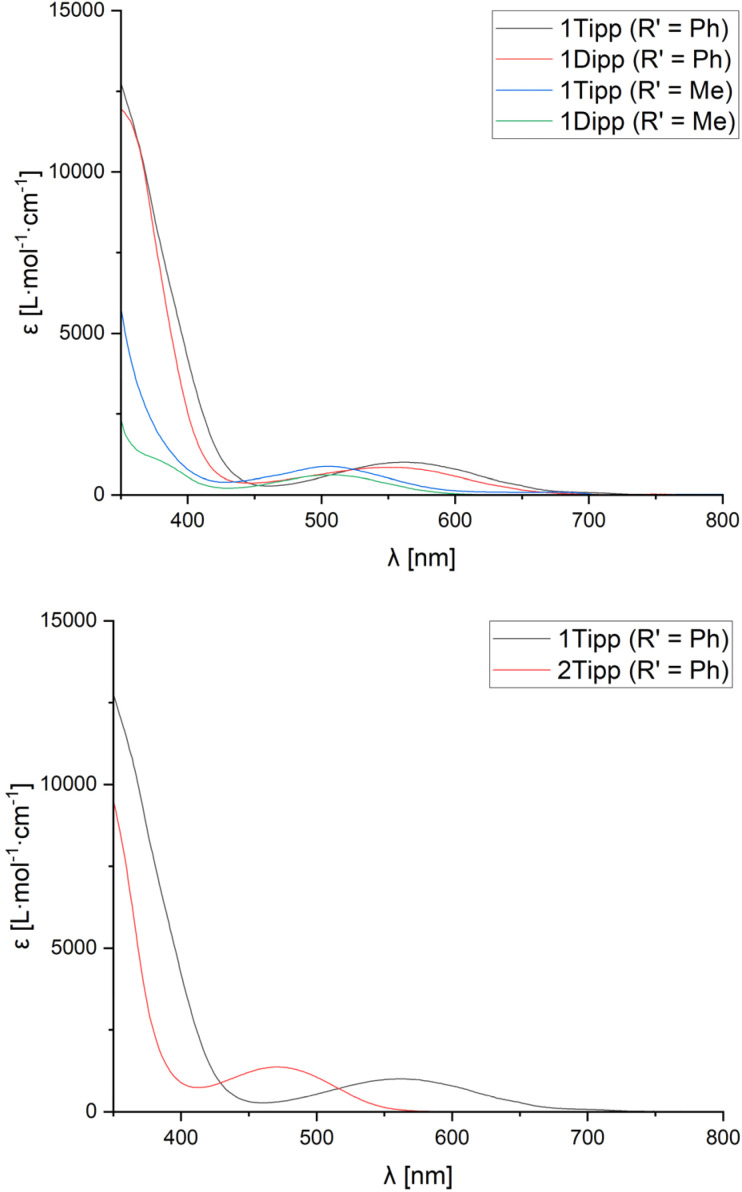
UV-visible spectra of [1_Tipp_]^+^ (R′ = Ph, Me) and [1_Dipp_]^+^ (R′ = Ph, Me) in CH_2_Cl_2_ solution at room temperature (top) and of 1_Tipp_ (R′ = Ph) compared to 2_Tipp_ (R′ = Ph) (bottom).

All compounds studied by UV-Vis spectroscopy show broad absorption bands in the 300–400 nm region typically associated with the aryl substituents at phosphorus (Table 1, *λ*_2_). However, the spectra of all cationic rings (except [1*_t_*_Bu_]^+^ (R′ = Me)) also exhibit a comparatively low-energy absorption band (Table 1, *λ*_1_) which follows Beer's Law. The low-energy absorption band observed in [1_R_]^+^ (R′ = Ph) is, on average, bathochromically shifted by 59 nm compared to the absorption band of the analogous [1_R_]^+^ (R′ = Me) species (Fig. 5, top), which is attributed to increased electronic conjugation involving the π-system of the aromatic Ph substituent (*vide infra*). When the steric bulk is increased at phosphorus (where ^*t*^Bu < Mes < Dipp ≈ Tipp), the bending angle of the P_3_CN-rings increases significantly, resulting in a progressive red-shift in the value of the low-energy absorption bands.

**Table 1:** Comparison of angle of ring bending and charge-transfer absorption band for crystallized [1_R_]^+^ and 2_R_ (R′ = Ph) species

Species	Angle (°)	*λ*_CT_ (nm)	Species	Angle (°)	*λ*_CT_ (nm)
[1*_t_*_Bu_]^+^ (R′ = Ph)	17.25	419	2*_t_*_Bu_ (R′ = Ph)	21.89	382
[1_Mes_]^+^ (R′ = Ph)	29.24	488	2_Mes_ (R′ = Ph)	23.02	NA
[1_Dipp_]^+^ (R′ = Ph)	50.64	550	2_Dipp_ (R′ = Ph)	27.5	461
[1_Tipp_]^+^ (R′ = Ph)	48.46	560	2_Tipp_ (R′ = Ph)	39.3	472
[1_Tipp_]^+^ (R′ = *p*-MeOC_6_H_4_)	50.65	555			
[1_Tipp_]^+^ (R′ = *p*-CF_3_C_6_H_4_)	57.17	583			

Unlike [1_R_]^+^ (R′ = Me), the spectra of 2_R_ (R′ = Me) showed no evidence of low-energy absorption bands between 300-800 nm, while spectra of 2_R_ (R′ = Ph) species showed low-energy absorption bands that are hypsochromically shifted relative to those observed in [1_R_]^+^ (R′ = Ph) (Table 1) and manifest in a change of observed colouration to colourless, yellow, or orange in all species 2_R_ (Fig. 5, bottom). In comparing [1_Tipp_]^+^ (R′ = Ph) to [1_Tipp_]^+^ (R′ = *p*-MeOC_6_H_4_ or *p*-CF_3_C_6_H_4_), we note that [1_Tipp_]^+^ (R′ = *p*-MeOC_6_H_4_) bears a hypsochromically shifted *λ*_1_ compared to [1_Tipp_]^+^ (R′ = Ph), while the *λ*_1_ associated with [1_Tipp_]^+^ (R′ = *p*-CF_3_C_6_H_4_) is bathochromically shifted by 20 nm compared to [1_Tipp_]^+^ (R′ = Ph). When considering the Hammett parameter of the *para*-substituent at R′ a correlation is found between an increasing Hammett parameter *σ*_p_, and an increase in the wavelength of the low-energy absorption band *λ*_1_ (*p*-MeO-C_6_H_4_ < *p*-H-C_6_H_4_ < *p*-CF_3_-C_6_H_4_).

### DFT studies of [1_R_]^+^ and 2_R_

**Fig. 6:**
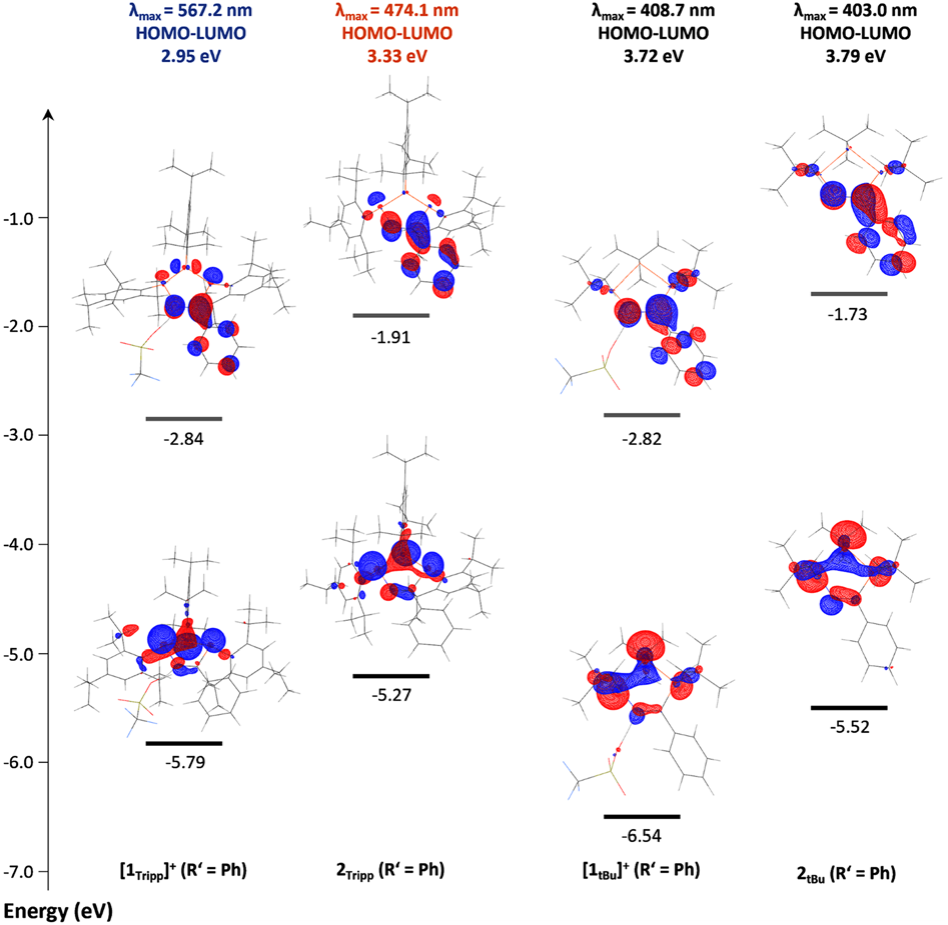
HOMO and LUMO frontier orbitals of [1_Tipp_]^+^ (R′ = Ph), 2_Tipp_ (R′ = Ph), [1*_t_*_Bu_]^+^ (R′ = Ph), and 2*_t_*_Bu_ (R′ = Ph), with lowest-energy charge transfer energies and corresponding absorption maxima.

To shed further light on the unusual nature of the UV-visible absorption bands and their seemingly strong correlation to the bending angle in cations [1_R_]^+^, the electronic structures of [1_Tipp_]^+^ and [1*_t_*_Bu_]^+^ (R′ = Me), 2_Tipp_ and 2*_t_*_Bu_ (R′ = Me), [1_R_]^+^ (R′ = Ph), and 2_R_ (R′ = Ph) were investigated by DFT calculations on the BP86-D3/def2SVP level of theory, while taking the molecular structures from SC-XRD experiments as a structural basis. In all cationic species [1_R_]^+^ the triflate ion was explicitly retained. Time-dependent density functional theory (TD-DFT) calculations were performed at the B3LYP/cc-pVTZ (in both the gas phase and using the polarizable continuum solvation model (PCM) for CH_2_Cl_2_ (smd = DCM), for comparison) level of density functional theory using the respective BP86-D3/def2-SVP optimized gas-phase S^0^ geometries, which were found to be in excellent agreement with the experimentally determined solid state structures (for complete computational details, including a comparison of the structural parameters, please refer to the ESI p. S-150 ff.†). The UV-Vis spectra of all species were reproduced well by the calculations (*cf.* compare ESI p. S-155 ff.†) in the gas phase. There is no significant difference between the data calculated in the gas phase and those using a PCM for solvation. The calculated lowest energy transitions (*λ*_1_) of [1_R_]^+^ correspond to a HOMO–LUMO transition, with all other transitions associated with transitions from the HOMO−1 and HOMO−2 orbitals to the LUMO or higher unoccupied molecular orbitals (ESI Section 6†). To better understand the origin of colour in these species, it is useful to assess the differences in the HOMO and LUMO orbitals of [1*_t_*_Bu_]^+^ (blue-shifted absorption), and those in [1_Tipp_]^+^ which absorbs at longer wavelengths (Fig. 6), and shows a considerably larger bending angle *θ*. In the case of [1_Tipp_]^+^ (R′ = Ph), the HOMO is best described mainly (*ca.* 74%) as linear combination of the n(P) orbitals (*e.g.* non-bonding combination, P lone pairs), with minimal delocalization into the C=N (*ca.* 5%) and Tipp (*ca.* 7%) π-systems. The LUMO, by comparison, is mainly delocalized over the NCPh unit and has π* character, with small contributions at P_C_, P_N_ and P_P_. The HOMO in [1*_t_*_Bu_]^+^ (R′ = Ph) is similar to that of [1_Tipp_]^+^ (R′ = Ph), while the LUMO again has mainly NCPh π* character, however, it lacks the contribution from P_P_ observed in [1_Tipp_]^+^ (R′ = Ph).

While the electronic differences in [1*_t_*_Bu_]^+^ and [1_Tipp_]^+^ (R′ = Ph) are seemingly small, the structural effects imposed by the bulky Tipp substituents on the molecule have a large influence on the HOMO–LUMO gap in these two species. For example, the ring bending in [1_Tipp_]^+^ (R′ = Ph) substantially destabilises the HOMO compared to that in [1*_t_*_Bu_]^+^ (R′ = Ph) while leaving the energy level of the LUMO virtually identical, resulting in a smaller HOMO–LUMO gap that allows a lower energy absorption in [1_Tipp_]^+^ (R′ = Ph). In the case of 2_R_, it should first be noted that deprotonation of both [1_Tipp_]^+^ and [1*_t_*_Bu_]^+^ (R′ = Ph) results in destabilisation of both the HOMO and LUMO, resulting in a greater delocalisation of the HOMO across the entire P_N_–P_P_–P_C_ framework and onto the PhCN moiety. However, Tipp substitution in 2_Tipp_ (R′ = Ph) results in destabilisation of the HOMO but stabilisation of the LUMO relative to 2*_t_*_Bu_ (R′ = Ph), again resulting in a smaller HOMO–LUMO gap and allowing for orange colouration to occur compared to the pale yellow 2*_t_*_Bu_ (R′ = Ph). To shed light on the nature of the HOMO–LUMO transition and the resultant unexpected range of colouration, we calculated the *D* indices of each species.

**Fig. 7:**
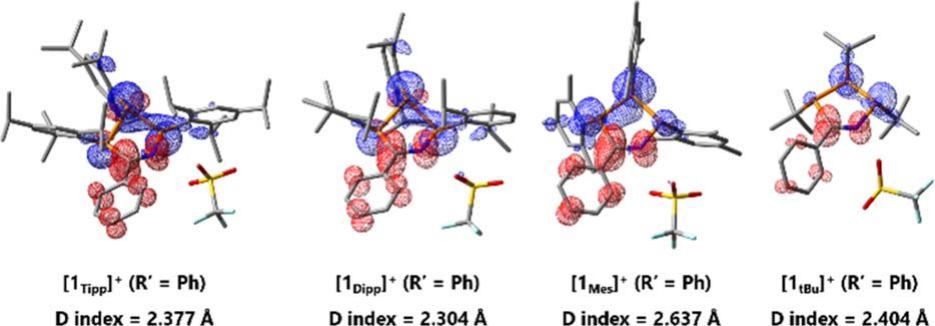
Charge density difference diagrams and *D* indices of the first excited state (HOMO–LUMO) illustrating charge transfers during excitation of [1_Tipp_]^+^, [1_Dipp_]^+^, [1_Mes_]^+^, and [1*_t_*_Bu_]^+^ (R′ = Ph); blue regions correspond to electron donor regions and red regions correspond to electron acceptor regions.

The *D* index is used to qualitatively assess the distance of donor–acceptor electron regions within a molecule by calculating the positive and negative barycenters of the orbitals involved in an electronic transition – in this case, the HOMO and LUMO – and then subsequently calculating the distance between these two barycenters. For values greater than 1.6 Å it can be determined that charge is flowing between the two barycenters, especially in cases molecules are not symmetric and where orbitals involved in the electronic transition have minimal spatial overlap, as is the case in [1_R_]^+^. In this way, the *D* index qualitatively supports the existence of charge transfer across a molecule.^83,84^ A comparison across the series of [1_R_]^+^ (R′ = Ph), reveals *D* indices of ≥2.3 Å, in line with a HOMO–LUMO charge transfer event from the non-bonding linear combination of n(P) orbitals to the N(H)CPh moiety in these species. This intramolecular charge transfer is nicely illustrated by plotting the charge density difference between S^0^ and S^1^ state (Fig. 7). We also observe large *D* values for the free bases 2_Tipp_ (R′ = Ph) and 2*_t_*_Bu_ (R′ = Ph), while [1_Tipp_]^+^ (R′ = Me), 2_Tipp_ (R′ = Me), and 2*_t_*_Bu_ (R′ = Me) all showed *D* values of less than 1.4 Å. This is in line with the colouration of benzonitrile-derived free bases, while acetonitrile-derived free bases appear weakly coloured, and computationally bear no significant HOMO–LUMO charge-transfer phenomena. However, there is no clear relation between the *D* indices and the bending angles of the P_3_CN-rings (Fig. S-189†).

### Preparation of a polymer-based Brønsted acid–base sensor

**Scheme 3:**
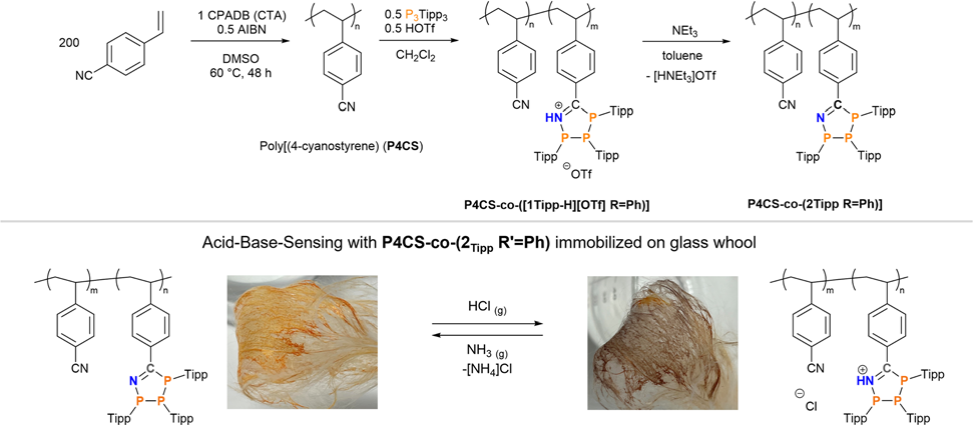
Top: Synthesis of the co-polymers P4CS-*co*-([1_Tipp-H_][OTf] R = Ph) and its neutral congener P4CS-*co*-(2_Tipp_ R = Ph). Bottom: Acid–base sensing with P4CS-*co*-(2_Tipp_ R = Ph) immobilized on glass wool.

Having shown reversible H^+^- and base-induced switching between the neutral triphosphaazolene and cationic triphosphaazolenium states, we were intrigued by the idea of incorporating these P_3_CN-species into a polymer through post-polymerization functionalization. Immobilization of such polymer should then enable to manufacture a chemical H^+^ sensor. First, low-dispersity poly(4-cyanostyrene) (P4CS) (*M*_n_ = 109.4 kDa, *Đ* = 1.14) was synthesised through an established reversible addition fragmentation chain-transfer polymerization (RAFT) protocol using 4-cyano-4-(phenylcarbothioylthio)pentanoic acid (CPADB) as RAFT-agent and azobisiobutyronitrile (AIBN) as initiator.^85^ Post-polymerization functionalization to synthesise a co-polymer was effected by suspending the as-synthesised poly(4-cyanostyrene) in CH_2_Cl_2_ and stepwise addition of 0.5 eq. of both (PTipp)_3_ and HOTf, which immediately afforded a purple suspension. After stirring for 12 h and precipitation with *n*-hexane P4CS-*co*-([1_Tipp_H][OTf] R′ = Ph) was afforded in 74% yield as purple beads. This insoluble purple polymer was suspended in toluene and an excess of neat NEt_3_ was added, resulting in the purple beads developing an orange colouration; continued stirring for 24 h gave an orange solution. The neutral, soluble polymer P4CS-*co*-(2_Tipp_ R′ = Ph) was afforded in good yields as an orange powder after workup (Scheme 3, top), which according to ^31^P NMR spectroscopy contained, in addition to the expected 1 : 1 : 1 resonances of the 1-aza-2,3,4-triphospholene, minimal amounts of an unidentified impurity (*δ*(^31^P) = 59.6 ppm), which could not be removed by further purification and therefore seems likely to be trapped in the polymer matrix (*cf.* ESI p. S-15 ff.†).

Compared to the starting poly(4-cyanostyrene) the dispersity (*Đ* = 3.8) of P4CS-*co*-(2_Tipp_ R′ = Ph) is considerably higher, which may be a result of phosphorus lone pairs in the polymer interacting with the GPC column. To immobilise P4CS-*co*-(2_Tipp_ R′ = Ph), oven-dried glass wool was dipped into a toluene solution of the neutral polymer and after drying for 24 h pale orange glass fibers were obtained. Sensing was tested by placing the bundled fibers in the headspace of a beaker containing concentrated aqueous (35%) HCl, which resulted in a noticeable colour change to purple (Scheme 3, bottom right), characteristic of P4CS-*co*-([1_Tipp_H][Cl] R′ = Ph). When these purple fibers were exposed to NH_3_ vapours by suspending the sample above saturated aqueous ammonium hydroxide, the purple colour vanished and the characteristic orange of P4CS-*co*-(2_Tipp_ R′ = Ph) was again observed (Scheme 3, bottom left). This procedure could be repeated at least five times, without noticeable build-up of [NH_4_]Cl on the glass fibers. Future studies will focus on obtaining P_3_CN-containing polymers that can be more easily processed to harness the proton-responsiveness in simple sensors more effectively.

## Discussion

**Fig. 8:**
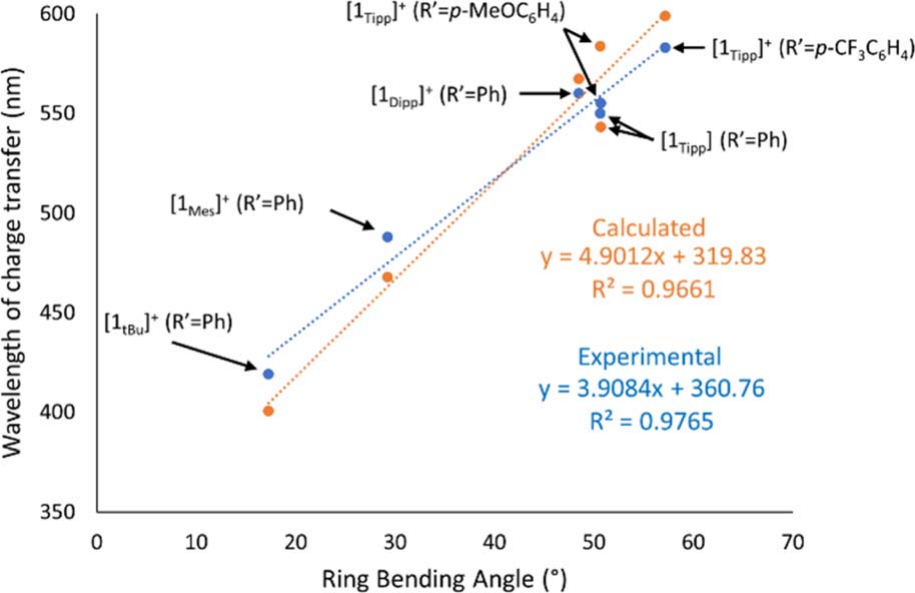
Graph depicting wavelength of charge transfer as a function of crystallographic ring-bending angle in [1_R_]^+^ species both experimentally (blue) and with calculated absorption maxima by TD-DFT (orange).

A rare example of tunable colouration in phosphorus-rich main group species is described *via* intramolecular charge transfer, as demonstrated prototypically in compound [1_Tipp_]^+^. While colour in poly-phosphorus species is not uncommon and can even be tuned electronically, colour typically arises from π–π* transitions, and this, to the best of our knowledge, is the first report on sterically-induced tuning of the HOMO energy, to enable facile modification of the colour in P_3_CN ring systems. The low-energy charge transfer arises from perturbation of the HOMO–LUMO energy levels, which can be related to the structural bending (*θ*) upon N-protonation of the P_3_CN heterocycle and is further enhanced by increased steric demand of the phosphorus substituents. This is corroborated by a strong correlation between the wavelength of charge-transfer (*λ*_CT_, previously labelled as *λ*_1_) as a function of the bending angle as observed in Table 1 and Fig. 8. Furthermore, solid-state UV-Vis spectra of a representative set of samples show near-identical *λ*_CT_ values to that of the analogous solution UV-Vis spectra (ESI Section 3, *cf.*[1_Tipp_]^+^ (R′ = Me) Fig. S-95 and S-96 ff.†), indicating that the bending angles are similar in solution and the solid-state. Additionally, modification of the R′ substituent affects the *λ*_CT_ as shown in Fig. 5, presumably by modulation of the LUMO energy level. *In silico* investigations into this charge transfer phenomenon reveal that the sterically-induced bending found in [1_R_]^+^ (R = Dipp, Tipp) raises the energy of the HOMO to allow for intramolecular charge transfer to occur in the visible region (ESI Section 6†). Further, these systems exhibit notable photostability in the solid state (*vide supra*), and further tuning of the HOMO–LUMO gap can be readily effected by varying the nature of the nitrile used in the synthesis.

## Conclusions

This contribution presents a thorough investigation of the structural and photophysical properties of 1-aza-2,3,4-triphospholenes and related 1-aza-2,3,4-triphospholenium cations, some of which exhibit strong colouration due to a hitherto unrealized charge transfer phenomenon, resulting in an unprecedented potential for control over colouration in phosphorus-rich molecules. Complexes [1_R_]^+^ show colouration associated with low-energy absorption bands that become increasingly red-shifted as steric bulk at the cyclic phosphane substituent is increased. Use of benzonitrile as a substrate allows access to yellow, red, and blue colours as steric bulk at phosphorus is increased in [1_R_]^+^, and colouration may be further tuned by the variation of the aryl nitrile substrate, for example use of *para*-substituted phenyl nitriles, as was shown for selected examples. UV-Visible spectroscopy revealed that [1_Tipp_]^+^ and [1_Dipp_]^+^ have broad, red-shifted absorption bands characteristic of intramolecular charge transfers. Single crystal X-ray crystallography revealed increasing ring bending of [1_R_]^+^ as steric encumbrance at the phosphorus centres are increased, while 2_R_ species conversely remain closer to planarity. A linear correlation was found between the angle of ring bending and the wavelength of the low-energy absorption bands across all benzonitrile-derived species (R′ = Ph), indicating a relationship between ground-state structure and the photophysical properties in this family of compounds. TD-DFT calculations support an intramolecular HOMO–LUMO (n(P) → π*) charge-transfer to which the observed colouration is ascribed. This unusual structure–property relationship provides insight for the rational design of pnictogen-rich charge transfer materials, while the facile and modular synthesis, along with photostability, tunable colour and proton responsiveness of 1-aza-2,3,4-triphospholenes, suggest these heterocycles may have considerable utility in numerous applications. As proof-of-concept, covalent incorporation of these moieties into a 4-cyanostyrene polymer backbone through post-polymerization modification allowed for expedient generation of an air-stable polymer capable of reversible Brønsted acid–base colorimetric chemosensing.

## Data availability

The data supporting this article have been included as part of the ESI.† Crystallographic data for compounds [1Tipp]^+^ (R′ = Me) (CCDC No. 2293093), [1Tipp]^+^ (R′ = Ph) (2293094), [1Dipp]^+^ (R′ = Me) (2293086), [1Dipp]^+^ (R′ = Ph) (2293087), [1Mes]^+^ (R′ = Me) (2293088), [1Mes]^+^ (R′ = Ph) (2293089), **[1^*t*^Bu]^+^ (R**′ **= Ph)** (2293090), 2Tipp (R′ = Me) (2293100), 2Tipp (R′ = Ph) (2293101), 2Dipp (R′ = Me) (2293095), 2Dipp (R′ = Ph) (2293096), 2Mes (R′ = Me) (2293097), 2Mes (R′ = Ph) (2293098), 2*^t^*Bu (R′ = Me) (2293099), [1Tipp]^+^ (R′ = *p*-MeOC_6_H_4_) (2293092), [1Tipp]^+^ (R′ = *p*-CF_3_C_6_H_4_) (2293091) and P_6_Mes_6_ (2293102) have been deposited at the CCDC under 2293086–2293102 and can be obtained free of charge *via*https://www.ccdc.cam.ac.uk/structures/. The geometries of all theoretically studied molecules have been uploaded as a separate xyz-file to provide a more intuitive view of the calculated 3D structures. A video of the acid–base-sensing has been uploaded as a separate file.

## Author contributions

M. A. N., E. A. L., J. E. T. W. and C. H.-J. carried out the experimental work. B. O. P. was responsible for SC-XRD experiments. L. W. and J. E. R. carried out the EPR measurements. The experimental work was designed by M. A. N., E. A. L., I. M. and C. H.-J., while supervision was shared between E. A. L., I. M. and C. H.-J. DFT studies were performed and evaluated by C. H.-J. The ESI was written through contributions from all authors. The manuscript was drafted by M. A. N. and finalized through contributions from all authors.

## Conflicts of interest

There are no conflicts to declare.

## Supplementary Material

SC-015-D4SC02497D-s001

SC-015-D4SC02497D-s002

SC-015-D4SC02497D-s003

SC-015-D4SC02497D-s004

## References

[cit1] Natta G. , From the Stereospecific Polymerization to the Asymmetric Autocatalytic Synthesis of Macromolecules, in Nobel Lectures, Chemistry 1963-1970, Elsevier Publishing Company, Amsterdam, 1972 10.1126/science.147.3655.261 17788204

[cit2] Ziegler K. , Consequences and Development of an Invention, in Nobel Lectures, Chemistry 1963-1970, Elsevier Publishing Company, Amsterdam, 1972

[cit3] Obligacion J. V., Chirik P. J. (2018). Nat. Rev. Chem..

[cit4] Harder S. , Early Main Group Metal Catalysis, 2020, pp. 151–173

[cit5] Novas B. T., Waterman R. (2022). ChemCatChem.

[cit6] Greenberg S., Stephan D. W. (2008). Chem. Soc. Rev..

[cit7] Leitao E. M., Jurca T., Manners I. (2013). Nat. Chem..

[cit8] Less R. J., Melen R. L., Naseri V., Wright D. S. (2009). Chem. Commun..

[cit9] Waterman R. (2013). Chem. Soc. Rev..

[cit10] Melen R. L. (2016). Chem. Soc. Rev..

[cit11] Priegert A. M., Rawe B. W., Serin S. C., Gates D. P. (2016). Chem. Soc. Rev..

[cit12] Vidal F., Jäkle F. (2019). Angew. Chem., Int. Ed..

[cit13] Manners I. (1996). Angew. Chem., Int. Ed..

[cit14] Knights A. W., Nascimento M. A., Manners I. (2022). Polymer.

[cit15] Manners I. (2002). J. Polym. Sci., Part A: Polym. Chem..

[cit16] Huisgen R., Szeimies G., Möbius L. (1967). Chem. Ber..

[cit17] Tornøe C. W., Christensen C., Meldal M. (2002). J. Org. Chem..

[cit18] Rostovtsev V. V., Green L. G., Fokin V. V., Sharpless B. (2002). Angew. Chem., Int. Ed..

[cit19] Rösch W., Facklam T., Regitz M. (1987). Tetrahedron.

[cit20] Sklorz J. A. W., Hoof S., Sommer M. G., Weißer F., Weber M., Wiecko J., Sarkar B., Müller C. (2014). Organometallics.

[cit21] Sklorz J. A. W., Müller C. (2016). Eur. J. Inorg. Chem..

[cit22] Papke M., Dettling L., Sklorz J. A. W., Szieberth D., Nyulászi L., Müller C. (2017). Angew. Chem., Int. Ed..

[cit23] Görlich T., Frost D. S., Boback N., Coles N. T., Dittrich B., Müller P., Jones W. D., Müller C. (2021). J. Am. Chem. Soc..

[cit24] Yang E. S., Mapp A., Taylor A., Beer P. D., Goicoechea J. M. (2023). Chem.–Eur. J..

[cit25] Pfeifer G., Papke M., Frost D., Sklorz J. A. W., Habicht M., Müller C. (2016). Angew. Chem., Int. Ed..

[cit26] O'Connor N. R., Wood J. L., Stoltz B. M. (2016). Isr. J. Chem..

[cit27] Singh P., Varshnaya R. K., Dey R., Banerjee P. (2020). Adv. Synth. Catal..

[cit28] Villinger A., Mayer P., Schulz A. (2006). Chem. Commun..

[cit29] Schulz A., Villinger A. (2008). Angew. Chem., Int. Ed..

[cit30] Velian A., Cummins C. C. (2015). Science.

[cit31] Jupp A. R., Goicoechea J. M. (2013). Angew. Chem., Int. Ed..

[cit32] Chen X., Alidori S., Puschmann F. F., Santiso-Quinones G., Benkő Z., Li Z., Becker G., Grützmacher H.-F., Grützmacher H. (2014). Angew. Chem., Int. Ed..

[cit33] Hinz A., Goicoechea J. M. (2016). Angew. Chem., Int. Ed..

[cit34] Goicoechea J. M., Grützmacher H. (2018). Angew. Chem., Int. Ed..

[cit35] Hoffmann R. (1982). Angew. Chem., Int. Ed. Engl..

[cit36] Wellnitz T., Hering-Junghans C. (2021). Eur. J. Inorg. Chem..

[cit37] Dyker C. A., Burford N., Menard G., Lumsden M. D., Decken A. (2007). Inorg. Chem..

[cit38] Holthausen M. H., Knackstedt D., Burford N., Weigand J. J. (2013). Aust. J. Chem..

[cit39] Gorbachuk E., Grell T., Hey-Hawkins E., Yakhvarov D. (2024). Eur. J. Inorg. Chem..

[cit40] Chitnis S. S., Sparkes H. A., Annibale V. T., Pridmore N. E., Oliver A. M., Manners I. (2017). Angew. Chem., Int. Ed..

[cit41] Hoffmann N., Wismach C., Jones P. G., Streubel R., Huy N. H. T., Mathey F. O. (2002). Chem. Commun..

[cit42] Sapochak L. S., Padmaperuma A. B., Vecchi P. A., Cai X., Burrows P. E. (2007). Proc. SPIE.

[cit43] Jeon S. O., Lee J. Y. (2012). J. Mater. Chem..

[cit44] Joly D., Tondelier D., Deborde V., Delaunay W., Thomas A., Bhanuprakash K., Geffroy B., Hissler M., Réau R. (2012). Adv. Funct. Mater..

[cit45] Stolar M. and Baumgartner T., P-Containing Heteroarenes: Synthesis, Properties, Applications, in Polycyclic Arenes and Heteroarenes, Wiley-VCH Verlag GmbH & Co. KGaA, 2016, pp. 309–330

[cit46] Duffy M. P., Delaunay W., Bouit P. A., Hissler M. (2016). Chem. Soc. Rev..

[cit47] Belyaev A., Chou P. T., Koshevoy I. O. (2021). Chem.–Eur. J..

[cit48] Wu N. M.-W., Ng M., Lam W. H., Wong H.-L., Yam V. W.-W. (2017). J. Am. Chem. Soc..

[cit49] Jiang X.-D., Zhao J., Xi D., Yu H., Guan J., Li S., Sun C.-L., Xiao L.-J. (2015). Chem.–Eur. J..

[cit50] Christianson A. M., Gabbaï F. P. (2016). Inorg. Chem..

[cit51] Qiu Y., Worch J. C., Chirdon D. N., Kaur A., Maurer A. B., Amsterdam S., Collins C. R., Pintauer T., Yaron D., Bernhard S., Noonan K. J. T. (2014). Chem.–Eur. J..

[cit52] Dimroth K., Hoffmann P. (1964). Angew. Chem., Int. Ed. Engl..

[cit53] Allmann R. (1965). Angew. Chem., Int. Ed..

[cit54] Dimroth K. , Delocalized phosphorus-carbon double bonds, in Phosphorus-Carbon Double Bonds, Springer-Verlag, 1964, vol. 38, pp. 1–147

[cit55] Jutzi P. (1975). Angew. Chem., Int. Ed. Engl..

[cit56] Ellis B. D., Dyker C. A., Decken A., Macdonald C. L. B. (2005). Chem. Commun..

[cit57] Binder J. F., Corrente A. M., Macdonald C. L. B. (2016). Dalton Trans..

[cit58] Macdonald C. L. B., Binder J. F., Swidan A. A., Nguyen J. H., Kosnik S. C., Ellis B. D. (2016). Inorg. Chem..

[cit59] Schmidpeter A., Lochschmidt S., Willhalm A. (1983). Angew. Chem., Int. Ed..

[cit60] Roy S., Stollberg P., Herbst-Irmer R., Stalke D., Andrada D. M., Frenking G., Roesky H. W. (2015). J. Am. Chem. Soc..

[cit61] Wu N. M.-W., Wong H.-L., Yam V. W.-W. (2017). Chem. Sci..

[cit62] Chan J. C.-H., Lam W. H., Wong H.-L., Wong W.-T., Yam V. W.-W. (2013). Angew. Chem., Int. Ed..

[cit63] He X., Borau-Garcia J., Woo A. Y. Y., Trudel S., Baumgartner T. (2013). J. Am. Chem. Soc..

[cit64] Riobé F., Szűcs R., Bouit P.-A., Tondelier D., Geffroy B., Aparicio F., Buendía J., Sánchez L., Réau R., Nyulászi L., Hissler M. (2015). Chem.–Eur. J..

[cit65] Chan J. C.-H., Wong H.-L., Wong W.-T., Yam V. W.-W. (2015). Chem.–Eur. J..

[cit66] Higashino T., Yamada T., Sakurai T., Seki S., Imahori H. (2016). Angew. Chem., Int. Ed..

[cit67] Delaunay W., Szűcs R., Pascal S., Mocanu A., Bouit P. A., Nyulászi L., Hissler M. (2016). Dalton Trans..

[cit68] Omori H., Hiroto S., Takeda Y., Fliegl H., Minakata S., Shinokubo H. (2019). J. Am. Chem. Soc..

[cit69] Baumgartner T. (2014). Acc. Chem. Res..

[cit70] Stolar M., Borau-Garcia J., Toonen M., Baumgartner T. (2015). J. Am. Chem. Soc..

[cit71] Bridges C. R., Borys A. M., Béland V. A., Gaffen J. R., Baumgartner T. (2020). Chem. Sci..

[cit72] Reus C., Stolar M., Vanderkley J., Nebauer J., Baumgartner T. (2015). J. Am. Chem. Soc..

[cit73] Das B., Makol A., Kundu S. (2022). Dalton Trans..

[cit74] Petrov A., Conrad L., Coles N. T., Weber M., Andrae D., Zagidullin A., Miluykov V., Müller C. (2022). Chem.–Eur. J..

[cit75] Sharma M. K., Weinert H. M., Li B., Wölper C., Henthorn J. T., Cutsail Iii G. E., Haberhauer G., Schulz S. (2023). Angew. Chem., Int. Ed..

[cit76] Weber L. (1992). Chem. Rev..

[cit77] Rottschäfer D., Sharma M. K., Neumann B., Stammler H.-G., Andrada D. M., Ghadwal R. S. (2019). Chem.–Eur. J..

[cit78] Liu L. L., Cao L. L., Zhou J., Stephan D. W. (2019). Angew. Chem., Int. Ed..

[cit79] Schumann A., Reiß F., Jiao H., Rabeah J., Siewert J.-E., Krummenacher I., Braunschweig H., Hering-Junghans C. (2019). Chem. Sci..

[cit80] Hering C., Schulz A., Villinger A. (2014). Chem. Sci..

[cit81] Gómez-Ruiz S., Frank R., Gallego B., Zahn S., Kirchner B., Hey-Hawkins E. (2011). Eur. J. Inorg. Chem..

[cit82] Pyykkö P., Atsumi M. (2009). Chem.–Eur. J..

[cit83] Le Bahers T., Adamo C., Ciofini I. (2011). J. Chem. Theory Comput..

[cit84] Huet L., Perfetto A., Muniz-Miranda F., Campetella M., Adamo C., Ciofini I. (2020). J. Chem. Theory Comput..

[cit85] Jin J., Li Y., Cao D., Wang S., Yan X. (2023). Angew. Chem., Int. Ed..

