# Supplementary material for: 1,3-Dipolar cyclisation reactions of nitriles with sterically encumbered cyclic triphosphanes: synthesis and electronic structure of phosphorus-rich heterocycles with tunable colour
Source: Chem Sci. 2024 Jun 19;15(30):12006–16. doi: 10.1039/d4sc02497d (PMC11290424; doi:10.1039/d4sc02497d)
Supplement: SC-015-D4SC02497D-s002 [file SC-015-D4SC02497D-s002.pdf]

Electronic Supplementary Material (ESI) for Chemical Science.
This journal is © The Royal Society of Chemistry 2024

Electronic Supplementary Information

1,3-Dipolar Cyclisation Reactions of Nitriles with Sterically Encumbered Cyclic Triphosphanes: Synthesis and Electronic Structure of Phosphorus-rich Heterocycles with Tunable Color

Mitchell A. Nascimento,[1] Etienne LaPierre,[1*] Brian O. Patrick,[2] Jade E. T. Watson,[1] Lara Watanabe,[3] Jeremy Rawson,[3] Christian Hering-Junghans,[4,5*] and Ian Manners.[1]

[1] Department of Chemistry, University of Victoria, 3800 Finnerty Rd, Victoria, British Columbia, V8P 5C2, Canada

[2] Department of Chemistry, University of British Columbia, 2036 Main Mall, Vancouver, British Columbia V6T 1Z1, Canada

[3] Department of Chemistry and Biochemistry, University of Windsor, 401 Sunset Avenue, Windsor, Ontario N9B 3P4, Canada

[4] Leibniz Institut für Katalyse e.V. (LIKAT), A.-Einstein-Str.3a, 18059 Rostock, Germany

[5] Institut für Chemie, Otto-von-Guericke-Universität Magdeburg, Universitätsplatz 2, 39106 Magdeburg, Germany

_Toc163916641

1. Experimental Details 2
2. NMR Spectra..18
3. UV-Visible Spectroscopy and Photostability Tests 112
4. EPR Analysis..125
5. Crystallographic Details..126
6. Computational Details..150
7. References 185

1. Experimental Details

1.1 General Considerations

Storage and manipulation of all compounds were performed under an inert atmosphere in a dinitrogen-filled MBraun 200B glovebox equipped with a cold-well, or using a dinitrogen Schlenk line using standard techniques. $P_3{}^tBu_3$, P_3Mes_3, P_3Dipp_3, and P_3Tipp_3 were synthesized as described in the literature.[1,2] The compounds **$[1_{tBu}]^+$ (R' = Me)**, **2_{tBu} (R' = Me)**, **$[1_{tBu}]^+$ (R' = Ph)**, and **2_{tBu} (R' = Ph)** were synthesized as per literature procedures.[3]

Toluene and *n*-hexane were dried and purified using an MBraun Grubbs/Dow solvent purification system[4] and were stored over activated 4 Å molecular sieves. C_6D_6 was dried over sodium/benzophenone, distilled and degassed prior to use. $CDCl_3$, MeCN, PhCN, and NEt_3 were dried over CaH_2 for 18 h, vacuum distilled and degassed prior to use. Fluorobenzene was dried over P_2O_5 for 18 h, then fractionally distilled and degassed prior to use. Triflic acid (HOTf) was purchased from Sigma Aldrich, was carefully transferred from a sealed ampule to a 100 mL Schlenk flask under a stream of dinitrogen, and subsequently cannula transferred to a 10 mL J Young ampoule for storage. All other reagents were purchased from Sigma-Aldrich and used as received.

^1H and ^{13}C NMR spectrometry chemical shifts were referenced to residual proteo-solvent resonances ($CD(H)Cl_3$ $\delta(^1H)$ = 7.26 ppm) and naturally abundant ^{13}C resonances for all deuterated solvents ($CD(H)Cl_3$ $\delta(^{13}C)$ = 77.16 ppm). All heteronuclear NMR spectra were referenced externally to IUPAC standards (^{31}P: 85% H_3PO_4; ^{19}F: $CFCl_3$). Chemical shift assignments are based on NMR experiments performed on Bruker Avance NEO 500 MHz or AV III 300 MHz spectrometers.

Elemental analyses were obtained using a Leco Tru Spec elemental analyzer device. Although some of the results are outside the range viewed as establishing analytical purity, they are provided to illustrate the best values obtained to date. ^1H NMR data confirmed in most cases the absence of (organic) side products (see experimental procedures for specific details).

Mass spectra were obtained using an Agilent 1260/6130 Quadrupol LC-MS (ESI) in ESI- mode for the detection of the triflate anion in **$[1_R]^+$**. High-resolution mass spectra for **$[1_R]^+$** and **2_R** were obtained on a Waters Acquity UPLC H-Class/Xevo G2-XS Time-of-Flight LC-MS (ESI) instrument.

1.2 Synthesis of 1-aza-2,3,4,-triphospholene heterocycles

[1$_{Tipp}$]$^+$ (R' = Me), **[1$_{Tipp}$]$^+$ (R' = Ph)**, **[1$_{Tipp}$]$^+$ (R' = p-MeOC$_6$H$_4$)**, **[1$_{Tipp}$]$^+$ (R' = p-CF$_3$C$_6$H$_4$)**, **[1$_{Dipp}$]$^+$ (R' = Me)**, **[1$_{Dipp}$]$^+$ (R' = Ph)**, **2$_{Tipp}$ (R' = Me)**, **2$_{Tipp}$ (R' = Ph)**, **2$_{Dipp}$ (R' = Me)**, and **2$_{Dipp}$ (R' = Ph)** were all synthesized in similar fashion. **[1$_{Mes}$]$^+$ (R' = Me)** was synthesized akin to **[1$_{Tipp}$]$^+$ (R' = Me)** but is unstable in solution and accordingly has not been isolated as spectroscopically pure material. **[1$_{Mes}$]$^+$ (R' = Ph)** can be synthesized in 94% spectroscopic yield but is consistently contaminated by an unknown protonated phosphorus-containing species at δ -65.6 ppm. **2$_{Mes}$ (R' = Me)** is highly soluble in all common solvents, so single crystals had to be grown out of a concentrated pentane solution stored at -30 °C for nearly a month to acquire sufficient spectroscopically pure material for NMR spectrum acquisition. The syntheses of **[1$_{Tipp}$]$^+$** and **2$_{Tipp}$ (R' = Me)** are outlined below as representative procedures, and their NMR spectra are fully assigned as a representative example of peak assignment in these systems.

[1$_{Tipp}$]$^+$ (R' = Me)

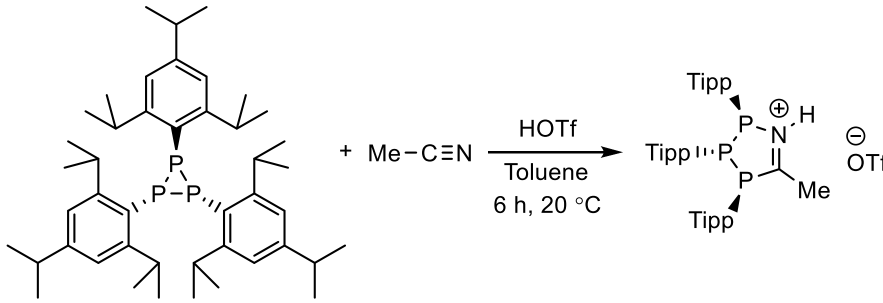

A 50 mL ampoule equipped with a J. Young valve and a hose barb was charged with 1 mmol of P$_3$Tipp$_3$ (578 mg), 20 mmol (2 mL) of MeCN, and 2 mL of toluene. 1 mmol (88 µL) of HOTf was added to the solution, which immediately turned deep red. The solution was allowed to stir for 6 h at 20 °C. The volatiles were then removed *in vacuo* at 40 °C. The residue was washed with *n*-hexane until the washings were no longer coloured, and the red solid was finally sonicated in *n*-hexane to remove any further impurities from the microcrystalline solid. Raw yield of microcrystalline powder: 840 mg (94%). The solid was then taken up in minimal PhF, layered with *n*-hexane, and was then placed at -30 °C for 13 days to yield single crystals of **[1$_{Tipp}$] (R' = Me)** (688 mg, 77%).

^{1}H NMR (500 MHz, CDCl$_3$): δ 12.61 (s, 1H, **N*H***), 7.19 (s, 1H, **Tipp-*ArH***), 7.13 (d, J = 6.4 Hz, 1H, **Tipp-*ArH***), 7.04 (d, J = 5.0 Hz, 4H, **Tipp-*ArH***), 4.58 (s, 2H, ***i*Pr-C*H***), 4.19 (dt, J = 13.0, 6.3 Hz, 1H, ***i*Pr-C*H***), 4.09 (dq, J = 13.6, 6.7 Hz, 1H, ***i*Pr-C*H***), 4.02 (dt, J = 12.0, 5.6 Hz, 2H, ***i*Pr-C*H***), 2.92 (dt, J = 13.8, 6.9 Hz, 1H, ***i*Pr-C*H***), 2.83 (ddt, J = 17.7, 13.8, 6.9 Hz, 2H, ***i*Pr-C*H***), 2.43 (dd, J = 10.2, 4.6 Hz, 3H, **N=C-C*H$_3$***), 1.48 (d, J = 6.7 Hz, 3H, ***i*Pr-C*H$_3$***), 1.45 (d, J = 6.4 Hz, 3H, ***i*Pr-C*H$_3$***), 1.35 (d, J = 6.6 Hz, 6H, ***i*Pr-C*H$_3$***), 1.26 (d, J = 6.9 Hz, 12H, ***i*Pr-C*H$_3$***), 1.24 – 1.20 (m, 9H, ***i*Pr-C*H$_3$***), 1.18 (d, J = 6.9 Hz, 9H, ***i*Pr-C*H$_3$***), 0.97 (s, 3H, ***i*Pr-C*H$_3$***), 0.95 (d, J = 6.7 Hz, 9H, ***i*Pr-C*H$_3$***).

^{19}F NMR (471 MHz, $CDCl_3$): δ -78.7 (***OTf***).

$^{31}P\{^1H\}$ NMR (203 MHz, $CDCl_3$): δ 80.5 (dd, $^1J_{PP}$ = 155.1, $^2J_{PP}$ 14.7 Hz, ***P**_N*), 53.6 (dd, $^1J_{PP}$ = 201.4, $^1J_{PP}$ = 154.6 Hz, ***P**_P*), 3.8 (dd, $^1J_{PP}$ = 201.3, $^2J_{PP}$ = 16.6 Hz, ***P**_C*).

^{13}C NMR (126 MHz, $CDCl_3$): δ 204.4 (d, J = 37.1 Hz, Me-**C**=N), 158.9 (d, J = 38.2 Hz, Tipp-*i*Pr-**C**$_{Ar}$), 158.0 (Tipp-*i*Pr-**C**$_{Ar}$), 157.9 (Tipp-*i*Pr-**C**$_{Ar}$), 156.7 (Tipp-*i*Pr-**C**$_{Ar}$), 156.5(Tipp-*i*Pr-**C**$_{Ar}$), 155.0 (Tipp-*i*Pr-**C**$_{Ar}$), 154.8 (Tipp-*i*Pr-**C**$_{Ar}$), 154.5 (d, J = 4.5 Hz, Tipp-*i*Pr-**C**$_{Ar}$), 153.0 (Tipp-*i*Pr-**C**$_{Ar}$), 124.9 (Tipp-***m***-**C**$_{Ar}$), 123.9 (Tipp-***m***-**C**$_{Ar}$), 123.4 (Tipp-***m***-**C**$_{Ar}$), 123.4 (Tipp-***m***-**C**$_{Ar}$), 123.2 (d, J = 9.3 Hz, Tipp-***m***-**C**$_{Ar}$), 121.9 (br d, J = 43.3 Hz, ***ipso***-**C**$_{Ar}$), 121.37 (Tipp-***m***-**C**$_{Ar}$), 120.3 (br d, J = 27.6 Hz, ***ipso***-**C**$_{Ar}$), 115.8 (br d, J = 36.4 Hz, ***ipso***-**C**$_{Ar}$), 35.4 (d, J = 11.5 Hz, *o*-*i*Pr_2**C**H), 34.61 (*p*-*i*Pr_2**C**H), 34.56 (*p*-*i*Pr_2**C**H), 34.4 (*p*-*i*Pr_2**C**H), 34.0 (d, J = 34.8 Hz, *o*-*i*Pr_2**C**H), 33.3 (m, *o*-*i*Pr_2**C**H)), 32.2 (d, J = 22.7 Hz, *o*-*i*Pr_2**C**H), 27.1 (Ar-CH-**C**H_3), 26.4 (Ar-CH-**C**H_3), 25.4 (Ar-CH-**C**H_3), 24.9 (Ar-CH-**C**H_3), 24.8 (Ar-CH-**C**H_3), 24.6 (dd, J = 8.4, 5.0 Hz, H_3**C**-C=N), 24.2 (Ar-CH-**C**H_3), 23.8 (Ar-CH-**C**H_3), 23.7 (Ar-CH-**C**H_3), 23.6 (Ar-CH-**C**H_3), 23.4 (Ar-CH-**C**H_3).

MS: $C_{47}H_{73}NP_3^+$ found: 744.4969 (expected: 744.4951) (-ESI 149 $CF_3SO_3^-$)

CHN: (expected) C, 64.48; H, 8.23; N, 1.57; S, 3.59; (found) C, 63.14; H, 7.42; N, 1.26; S, 3.54.

Based on ^{1}H NMR experiments the presence of significant (soluble organic) impurities can be excluded (cf. Figure S1).

2$_{Tipp}$ (R' = Me)

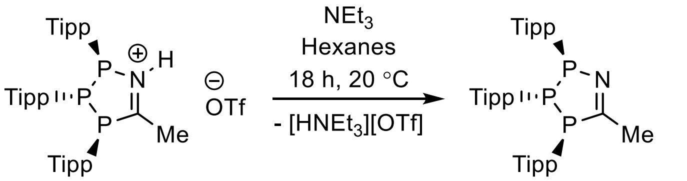

In a nitrogen-filled glovebox, a 1dr vial was charged with 200 mg (0.2237 mmol) of **[1$_{Tipp}$] (R' = Me)** and 2 mL of *n*-hexane. 1.1 equivalents of NEt_3 (25 mg, 0.24 mmol) were added, and the reaction mixture was stirred for 18 h at 20 °C. The reaction was then placed at -30 °C for 24 h to allow for the precipitation of [$HNEt_3$][OTf] from *n*-hexane, and the supernatant was filtered through a 0.2 μm PTFE syringe filter. The *n*-hexane was pumped down to concentrate the solution (no incipient precipitation is seen, as the free base tends to oil out), and the solution was placed at -30 °C for 17 days to afford large single crystals of **2$_{Tipp}$ (R' = Me)** (128 mg, 56%).

^{1}H NMR (500 MHz, $CDCl_3$): δ 7.04 (s, 1H, **Tipp-*ArH***), 7.02 (s, 1H, **Tipp-*ArH***), 6.96 (s, 1H, **Tipp-*ArH***), 6.95 (s, 1H, **Tipp-*ArH***), 4.53 – 4.41 (m, 2H, ***i*Pr-C*H***), 4.41 – 4.29 (m, 1H, ***i*Pr-C*H***), 4.13 – 4.00 (m, 1H, ***i*Pr-C*H***), 3.87 – 3.74 (m, J = 6.5 Hz, 2H, ***i*Pr-C*H***), 2.93 – 2.75 (m, 3H, ***i*Pr-C*H***), 2.10 (t, J = 5.6 Hz, 3H, **N=C-C*H₃***), 1.33 (d, J = 5.7 Hz, 3H, ***i*Pr-C*H₃***), 1.29 (d, 3H, ***i*Pr-C*H₃***), 1.24 (dd, J = 6.8, 1.7 Hz, 15H, ***i*Pr-C*H₃***), 1.22 – 1.18 (m, 15H, ***i*Pr-C*H₃***), 1.16 (d, J = 6.7

Hz, 7H, ***i*Pr-C*H*$_3$**), 1.07 (d, J = 6.5 Hz, 3H, ***i*Pr-C*H*$_3$**), 1.00 (d, J = 6.7 Hz, 7H, ***i*Pr-C*H*$_3$**), 0.93 (d, J = 6.7 Hz, 6H, ***i*Pr-C*H*$_3$**).

^{31}P{^{1}H} NMR (203 MHz, $CDCl_3$): δ 82.3 (dd, $^1J_{PP}$ = 185.4, $^2J_{PP}$ = 37.5 Hz, $\boldsymbol{P_N}$), 34.2 (dd, $^1J_{PP}$ = 220.5, $^1J_{PP}$ = 185.5 Hz, $\boldsymbol{P_P}$), 19.0 (dd, $^1J_{PP}$ = 220.4, $^2J_{PP}$ = 37.6 Hz, $\boldsymbol{P_C}$).

^{13}C NMR (126 MHz, $CDCl_3$): δ 181.5 (d, J = 41.9 Hz, Me-**C**=N), 157.3 (br d, J = 33.5 Hz, Tipp-*i*Pr-**C**$_{Ar}$), 156.9 (Tipp-*i*Pr-**C**$_{Ar}$), 156.8 (Tipp-*i*Pr-**C**$_{Ar}$), 154.4 (br s, Tipp-*i*Pr-**C**$_{Ar}$), 154.3 (Tipp-*i*Pr-**C**$_{Ar}$), 154.2 (Tipp-*i*Pr-**C**$_{Ar}$), 151.4 (Tipp-*i*Pr-**C**$_{Ar}$), 150.2 (Tipp-*i*Pr-**C**$_{Ar}$), 150.0 (Tipp-*i*Pr-**C**$_{Ar}$), 130.3 (br dd, J = 13.3, 3.9 Hz, ***ipso*-C**$_{Ar}$), 130.0 (br dd, J = 12.5, 2.9 Hz ***ipso*-C**$_{Ar}$), 123.9 – 123.6 (br m, ***ipso*-C**$_{Ar}$), 123.4 (br s, Tipp-***m*-C**$_{Ar}$), 122.3 (s, Tipp-***m*-C**$_{Ar}$), 121.71 (s, Tipp-***m*-C**$_{Ar}$), 121.73 (s, Tipp-***m*-C**$_{Ar}$), 121.5 (br s, Tipp-***m*-C**$_{Ar}$), 34.21 (s, two *p*-*i*Pr_2**C**H), 34.19 (s, *p*-*i*Pr_2**C**H), 33.4 (d, J = 13.3 Hz, *o*-*i*Pr_2**C**H), 32.8 – 32.4 (m, two *o*-*i*Pr_2**C**H), 32.2 (d, J = 38.9 Hz, *o*-*i*Pr_2**C**H), 31.2 (d, J = 19.0 Hz, two *o*-*i*Pr_2**C**H), 26.8 (dd, J = 26.6, 7.5 Hz), 26.3 (Ar-CH-**C**H_3), 25.6 (Ar-CH-**C**H_3), 25.0 (Ar-CH-**C**H_3), 24.7 (Ar-CH-**C**H_3), 24.7 (Ar-CH-**C**H_3), 24.4 (Ar-CH-**C**H_3), 24.2 (Ar-CH-**C**H_3), 23.82 (Ar-CH-**C**H_3), 23.79 (Ar-CH-**C**H_3), 23.8 (Ar-CH-**C**H_3), 23.7 (Ar-CH-**C**H_3), 23.6 (Ar-CH-**C**H_3).

No MS and CHN data was collected for this sample.

[1$_{Tipp}$]$^+$ (R' = Ph)

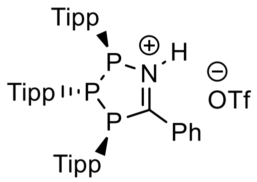

Reaction conducted using 300 mg (0.427 mmol) of P_3Tipp_3.Yield after recrystallization (PhF/*n*-hexane): 272 mg (0.337 mmol, 79%).

^{1}H NMR (500 MHz, $CDCl_3$): δ 12.44 (d, J = 14.6 Hz, 1H, **N*H***), 7.61 (d, J = 7.9 Hz, 2H, **Ph *o-H***), 7.50 (t, J = 7.5 Hz, 1H, **Ph *p-H***), 7.37 (t, J = 7.7 Hz, 2H, **Ph *m-H***), 7.15 (s, 2H, **Tipp-*ArH***), 7.06 (s, 2H, **Tipp-*ArH***), 6.98 (d, J = 6.1 Hz, 1H, **Tipp-*ArH***), 6.92 (s, 1H, **Tipp-*ArH***), 4.59 (s, 2H, ***i*Pr-C*H***), 4.41 – 4.27 (m, 2H, ***i*Pr-C*H***), 3.91 (hept, J = 13.5, 6.6 Hz, 1H, ***i*Pr-C*H***), 3.73 (p, J = 6.4 Hz, 1H, ***i*Pr-C*H***), 2.91 (p, J = 13.8, 6.9 Hz, 1H, ***i*Pr-C*H***), 2.83 (hept, J = 13.8, 6.8 Hz, 2H, ***i*Pr-C*H***), 1.45 – 1.38 (m, 9H, ***i*Pr-C*H*$_3$**), 1.34 (d, J = 6.6 Hz, 3H, ***i*Pr-C*H*$_3$**), 1.26 (d, J = 6.9 Hz, 12H, ***i*Pr-C*H*$_3$**), 1.21 (t, J = 7.3 Hz, 12H, ***i*Pr-C*H*$_3$**), 1.17 (dd, J = 6.8, 4.6 Hz, 6H, ***i*Pr-C*H*$_3$**), 1.02 (d, J = 6.4 Hz, 6H, ***i*Pr-C*H*$_3$**), 0.69 (d, J = 6.7 Hz, 3H, ***i*Pr-C*H*$_3$**), 0.47 (d, J = 6.6 Hz, 3H, ***i*Pr-C*H*$_3$**).

^{19}F NMR (471 MHz, $CDCl_3$): δ -78.55 (***OTf***).

^{31}P{^{1}H} NMR (203 MHz, $CDCl_3$): δ 87.7 (dd, $^1J_{PP}$ = 183.1, $^2J_{PP}$ = 15.2 Hz, $\boldsymbol{P_N}$), 42.9 (dd, $^1J_{PP}$ = 183.2, $^1J_{PP}$ = 167.1 Hz, $\boldsymbol{P_P}$), -5.8 (dd, $^1J_{PP}$ = 166.9, $^2J_{PP}$ = 16.1 Hz, $\boldsymbol{P_C}$).

^{13}C NMR (126 MHz, $CDCl_3$): δ 195.9 (d, J = 42.5 Hz, **Ph-*C*=N**), 157.9 (Tipp-*i*Pr-**C**$_{Ar}$), 157.8 (Tipp-*i*Pr-**C**$_{Ar}$), 156.8 (Tipp-*i*Pr-**C**$_{Ar}$), 156.7 (Tipp-*i*Pr-**C**$_{Ar}$), 156.4 (Tipp-*i*Pr-**C**$_{Ar}$), 154.7 (Tipp-*i*Pr-**C**$_{Ar}$), 154.2 (Tipp-*i*Pr-**C**$_{Ar}$), 153.3 (d, J = 5.4 Hz, Tipp-*i*Pr-**C**$_{Ar}$), 152.7 (Tipp-*i*Pr-**C**$_{Ar}$), 135.1 (**Ph *p-C***), 131.9 (t, J = 6.3 Hz, ipso ***C*-C=N**), 129.4 (**Ph *m-C***), 129.2 (d, J = 7.4 Hz, **Ph *o-C***), 124.6 (Tipp-***m*-C**$_{Ar}$), 123.7 (Tipp-***m*-C**$_{Ar}$), 123.6 (dd, J = 54.0, 6.5 Hz, ***ipso*-C**$_{Ar}$), 123.54 (Tipp-***m*-C**$_{Ar}$),

123.51 (Tipp-***m***-**C**$_{Ar}$), 122.5 (d, J = 9.3 Hz, Tipp-***m***-**C**$_{Ar}$), 122.5 (dd, J = 49.7, 7.6 Hz, ***ipso***-**C**$_{Ar}$), 116.3 (d, J = 44.0 Hz, ***ipso***-**C**$_{Ar}$), 35.4 (*i*Pr$_2$**C**H), 34.5 (*i*Pr$_2$**C**H), 34.3 (*i*Pr$_2$**C**H), 34.2 (*i*Pr$_2$**C**H), 33.9 (d, J = 35.9 Hz, *i*Pr$_2$**C**H), 33.3 (dt, J = 19.0, 7.5 Hz, two *i*Pr$_2$**C**H), 32.3 (dd, J = 22.5, 2.9 Hz, two *i*Pr$_2$**C**H), 25.3 (Ar-CH-**C**H$_3$), 25.25 (Ar-CH-**C**H$_3$), 24.71 (Ar-CH-**C**H$_3$), 24.65 (Ar-CH-**C**H$_3$), 24.2 (Ar-CH-**C**H$_3$), 24.1 (Ar-CH-**C**H$_3$), 23.62 (Ar-CH-**C**H$_3$), 23.55 (Ar-CH-**C**H$_3$), 23.54 (Ar-CH-**C**H$_3$), 23.50 (Ar-CH-**C**H$_3$), 23.43 (Ar-CH-**C**H$_3$), 23.42 (Ar-CH-**C**H$_3$).

MS: $C_{52}H_{75}NP_3^+$ found: 806.5117 (expected: 806.5107) (-ESI 149 $CF_3SO_3^-$)

CHN: (expected) C, 65.77; H, 7.87; N, 1.42; S, 3.25; (found) C, 65.79; H, 7.27; N, 1.22; S, 3.512$_{Tipp}$ (R' = Ph)

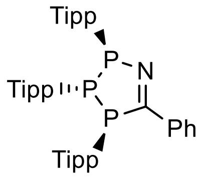

Reaction conducted using 103 mg (0.108 mmol) of **[1$_{Tipp}$]$^+$ (R = Ph)**.Yield after recrystallization (pentane): 52 mg (0.0637 mmol, 59%)

^{1}H NMR (500 MHz, CDCl$_3$): δ 7.70 (d, J = 7.5 Hz, 2H, **Ph *o-H***), 7.22 (dt, J = 14.5, 6.9 Hz, 3H, **Ph *p-H* and Ph *m-H***), 7.02 (d, J = 2.1 Hz, 2H, **z**), 6.96 (s, 2H, **Tipp-*ArH***), 6.94 – 6.90 (m, 1H, **Tipp-*ArH***), 6.83 (s, 1H, **Tipp-*ArH***), 4.29 (dq, J = 19.7, 6.4 Hz, 3H , ***i*Pr-C*H***), 3.73 (dh, J = 19.5, 6.8 Hz, 3H, ***i*Pr-C*H***), 2.93 – 2.71 (m, 3H, , ***i*Pr-C*H***), 1.31 (d, J = 6.6 Hz, 2H, ***i*Pr-C*H₃***), 1.25 (dd, J = 6.7, 3.1 Hz, 12H, ***i*Pr-C*H$_3$***), 1.21 (d, J = 6.9 Hz, 6H, ***i*Pr-C*H$_3$***), 1.16 (dt, J = 7.1, 3.8 Hz, 12H, ***i*Pr-C*H$_3$***), 1.08 (d, J = 6.6 Hz, 6H, ***i*Pr-C*H$_3$***), 0.95 (d, J = 6.4 Hz, 6H, ***i*Pr-C*H$_3$***), 0.74 (d, J = 6.6 Hz, 3H, ***i*Pr-C*H$_3$***), 0.59 (d, J = 6.4 Hz, 3H, ***i*Pr-C*H$_3$***).

^{31}P{^{1}H} NMR (203 MHz, CDCl$_3$): δ 90.6 (dd, $^1J_{PP}$ = 210.2, $^2J_{PP}$ = 20.5 Hz, $\boldsymbol{P_N}$), 28.26 (dd, $^1J_{PP}$ = 210.3, $^1J_{PP}$ = 205.5 Hz, $\boldsymbol{P_P}$), 18.29 (dd, $^1J_{PP}$ = 205.0, $^2J_{PP}$ = 20.4 Hz, $\boldsymbol{P_C}$).

^{13}C NMR (126 MHz, CDCl$_3$): δ 177.7 (d, J = 40.8 Hz, **Ph-*C*=N**), 156.8 (Tipp-*i*Pr-**C**$_{Ar}$), 156.7 (Tipp-*i*Pr-**C**$_{Ar}$), 155.3 (d, J = 33.8 Hz, Tipp-*i*Pr-**C**$_{Ar}$), 154.2 (Tipp-*i*Pr-**C**$_{Ar}$), 153.1 (Tipp-*i*Pr-**C**$_{Ar}$), 152.9 (Tipp-*i*Pr-**C**$_{Ar}$), 151.0 (Tipp-*i*Pr-**C**$_{Ar}$), 150.0 (Tipp-*i*Pr-**C**$_{Ar}$), 149.6 (Tipp-*i*Pr-**C**$_{Ar}$), 139.0 (d, J = 7.4 Hz, ipso ***C*-C=N**), 131.7 (dd, J = 44.4, 14.0 Hz, ***ipso***-**C**$_{Ar}$), 129.7 (**Ph *p-C***), 128.34 (**Ph *o-C***), 128.30 (**Ph *o-C***), 127.9 (**Ph *m-C***), 123.9 (dd, J = 53.2, 11.0 Hz, ***ipso***-**C**$_{Ar}$), 123.4 (Tipp-***m***-**C**$_{Ar}$), 122.3 (Tipp-***m***-**C**$_{Ar}$), 121.72 (Tipp-***m***-**C**$_{Ar}$), 121.70 (Tipp-***m***-**C**$_{Ar}$), 121.3 (d, J = 6.9 Hz, Tipp-***m***-**C**$_{Ar}$), 34.2 (***i*Pr-C*H***), 34.1 (***i*Pr-C*H***), 33.4 (d, J = 8.4 Hz, ***i*Pr-C*H***), 32.9 (dt, J = 18.0, 8.7 Hz, two ***i*Pr-C*H***), 32.2 (d, J = 37.8 Hz, (***i*Pr-C*H***), 31.2 (***i*Pr-C*H***), 31.0 (***i*Pr-C*H***), 25.5 (***i*Pr-C*H$_3$***), 25.4 (***i*Pr-C*H$_3$***), 25.1 (***i*Pr-C*H$_3$***), 24.9 (***i*Pr-C*H$_3$***), 24.7 (***i*Pr-C*H$_3$***), 24.3 (***i*Pr-C*H$_3$***), 24.1 (***i*Pr-C*H$_3$***), 24.0 (***i*Pr-C*H$_3$***), 23.9 (***i*Pr-C*H$_3$***), 23.83 (***i*Pr-C*H$_3$***), 23.77 (***i*Pr-C*H$_3$***), 23.73 (***i*Pr-C*H$_3$***), 23.68 (***i*Pr-C*H$_3$***), 23.63 (***i*Pr-C*H$_3$***).

MS: $[C_{52}H_{73}NP_3+H]^+$ found: 806.5112 (expected: 806.5107).

CHN: (expected) C, 77.48; H, 9.25; N, 1.74; (found) C, 77.01; H, 8.99; N, 1.29.

[1_{Dipp}]$^+$ (R' = Me)

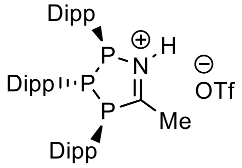

Reaction conducted using 300 mg (0.520 mmol) of P_3Dipp_3. Yield after recrystallization (PhF/*n*-hexane): 312 mg (0.406 mmol, 78%)^{1}H NMR (500 MHz, $CDCl_3$): δ 12.77 (s, 1H, ***NH***), 7.57 (t, J = 7.8 Hz, 1H, *Dipp* ***p-H***), 7.46 (t, J = 7.8 Hz, 1H, *Dipp* ***p-H***), 7.39 (q, J = 7.7 Hz, 2H, *Dipp* ***p-H*** and *Dipp* ***m-H***), 7.31 (t, J = 7.2 Hz, 1H, *Dipp* ***m-H***), 7.26 – 7.20 (m, 4H, *Dipp* ***m-H***), 4.58 (s, 2H, ***i*Pr-C*H***), 4.16 (p, J = 12.9, 6.5 Hz, 1H, ***i*Pr-C*H***), 4.08 (hept, J = 13.6, 6.7 Hz, 1H, ***i*Pr-C*H***), 4.03 – 3.95 (m, 2H, ***i*Pr-C*H***), 2.45 (dd, J = 10.3, 5.0 Hz, 3H, **N=C-C*H₃***), 1.49 (d, J = 6.7 Hz, 3H, ***i*Pr-C*H₃***), 1.45 (d, J = 6.5 Hz, 3H, ***i*Pr-C*H₃***), 1.36 (d, J = 6.7 Hz, 6H, ***i*Pr-C*H₃***), 1.28 (d, J = 6.7 Hz, 6H, ***i*Pr-C*H₃***), 1.24 (d, J = 6.8 Hz, 3H, ***i*Pr-C*H₃***), 1.16 (d, J = 6.8 Hz, 3H, ***i*Pr-C*H₃***), 1.02 (d, J = 6.7 Hz, 6H, ***i*Pr-C*H₃***), 1.01 (d, J = 6.7 Hz, 6H, ***i*Pr-C*H₃***).

^{19}F NMR (471 MHz, $CDCl_3$): δ -78.60 (***OTf***).

^{31}P{^{1}H} NMR (203 MHz, $CDCl_3$): δ 84.48 (d, $^1J_{PP}$ = 159.2 Hz, $\boldsymbol{P_N}$), 53.09 (dd, $^1J_{PP}$ = 202.5, $^1J_{PP}$ = 159.9 Hz, $\boldsymbol{P_P}$), 6.15 (dd, $^1J_{PP}$ = 202.6, $^2J_{PP}$ = 14.3 Hz, $\boldsymbol{P_C}$).

^{13}C NMR (126 MHz, $CDCl_3$): δ 204.7 (d, J = 36.1 Hz, **Me-*C*=N**), 158.9 (d, J = 37.7 Hz, Dipp-*i*Pr-$\mathbf{C_{Ar}}$), 158.1 (Dipp-*i*Pr-$\mathbf{C_{Ar}}$), 158.0 (Dipp-*i*Pr-$\mathbf{C_{Ar}}$), 156.5 (Dipp-*i*Pr-$\mathbf{C_{Ar}}$), 156.4 (Dipp-*i*Pr-$\mathbf{C_{Ar}}$), 154.5 (d, J = 4.1 Hz, Dipp-*i*Pr-$\mathbf{C_{Ar}}$), 134.1 (**Dipp-*p-ArH***), 133.9 (**Dipp-*p-ArH***), 132.3 (**Dipp-*p-ArH***), 126.6 (**Dipp-*p-ArH***), 125.7 (two **Dipp-*p-ArH***), 125.3 (**Dipp-*p-ArH***), 125.2 (**Dipp-*p-ArH***), 125.1 (d, J = 9.1 Hz, **Dipp-*p-ArH***), 124.9 (dd, J = 32.2, 10.4 Hz, ***ipso*-C_{Ar}**), 123.3 (dd, J = 38.0, 15.4 Hz, ***ipso*-C_{Ar}**), 119.4 (dd, J = 56.6, 10.7 Hz, ***ipso*-C_{Ar}**), 35.6 (d, J = 3.2 Hz, ***i*Pr-C*H***), 35.5 (d, J = 1.7 Hz, ***i*Pr-C*H***), 34.2 (d, J = 35.4 Hz, ***i*Pr-C*H***), 33.5 (dt, J = 18.1, 9.7 Hz, two ***i*Pr-C*H***), 32.4 (***i*Pr-C*H***), 32.2 (d, J = 3.0 Hz, ***i*Pr-C*H***), 27.1 (***i*Pr-C*H₃***), 26.2 (***i*Pr-C*H₃***), 25.4 (two ***i*Pr-C*H₃***), 24.9 (two ***i*Pr-C*H₃***), 24.79 (two ***i*Pr-C*H₃***), 24.68 (dd, J = 8.8, 5.3 Hz, ***H₃C*-C=N**), 24.31 (two ***i*Pr-C*H₃***), 24.15 (***i*Pr-C*H₃***), 23.58 (***i*Pr-C*H₃***).

MS: $C_{38}H_{55}NP_3^+$ found: 618.3563 (expected: 618.3542) (-ESI 149 $CF_3SO_3^-$)

CHN: (expected) C, 61.01; H, 7.22; N, 1.82; S, 4.18; (found) C, 60.36; H, 6.72; N, 1.54; S, 4.78.

2_{Dipp} (R' = Me)

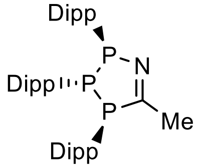

Reaction conducted using 99 mg (0.129 mmol) of **[1_{Dipp}]$^+$ (R = Me)**. Yield after recrystallization (pentane): 52 mg (0.0839 mmol, 65%).

^{1}H NMR (500 MHz, $CDCl_3$): δ 7.36 (t, J = 7.7 Hz, 1H, *Dipp* ***p-H***), 7.27 (dt, J = 15.3, 7.8 Hz, 2H, *Dipp* ***p-H***), 7.17 (d, J = 7.1 Hz, 2H, *Dipp* ***m-H***), 7.16 – 7.10 (m, 4H, *Dipp* ***m-H***), 4.24 (bs, 1H, ***i*Pr-C*H***), 4.19 (bdq, J = 12.0, 6.4 Hz, 2H, ***i*Pr-C*H***), 3.71 (dq, J = 13.1, 6.6 Hz, 2H, ***i*Pr-C*H***), 3.66 (bs, 1H, ***i*Pr-C*H***), 2.18 (t, J = 5.7 Hz, 3H, **N=C-C*H₃***), 1.27 – 1.23 (bm, 3H, ***i*Pr-C*H₃***), 1.22 (d, J = 6.7 Hz, 9H, ***i*Pr-C*H₃***), 1.14 (d, J = 6.7 Hz, 9H, ***i*Pr-C*H₃***),

1.10 (d, J = 6.7 Hz, 6H, ***i*Pr-C*H*$_3$**), 1.01 (d, J = 6.7 Hz, 6H, ***i*Pr-C*H*$_3$**), 0.99 – 0.95 (bm, 3H, ***i*Pr-C*H*$_3$**).

$^{31}P\{^1H\}$ NMR (203 MHz, $CDCl_3$): ABX spin system. δ 98.56 (dd, J = 196.9, 10.4 Hz), 33.31 – 20.11 (m).

^{13}C NMR (126 MHz, $CDCl_3$): δ 182.4 (d, J = 43.4 Hz, **Me-*C*=N**), 157.1 (Dipp-*i*Pr-**C**$_{Ar}$), 156.8 (Dipp-*i*Pr-**C**$_{Ar}$), 156.7 (Dipp-*i*Pr-**C**$_{Ar}$), 154.6 (Dipp-*i*Pr-**C**$_{Ar}$), 153.3 (Dipp-*i*Pr-**C**$_{Ar}$), 153.2 (Dipp-*i*Pr-**C**$_{Ar}$), 134.5 (d, J = 31.9 Hz, ***ipso*-C**$_{Ar}$), 131.0 (**Dipp-*p-ArH***), 129.8 (**Dipp-*p-ArH***), 129.3 (**Dipp-*p-ArH***), 128.1 (d, J = 39.8 Hz, ***ipso*-C**$_{Ar}$), 125.5 – 125.0 (m, **Dipp-*m-ArH***), 124.12 (**Dipp-*m-ArH***), 124.09 (**Dipp-*m-ArH***), 123.38 (**Dipp-*m-ArH***), 123.37 (**Dipp-*m-ArH***), 123.2 (dd, J = 11.2, 5.2 Hz, (**Dipp-*m-ArH***)), 33.5 – 33.2 (bm, ***i*Pr-C*H***), 33.0 (dt, J = 18.3, 9.3 Hz, two ***i*Pr-C*H***), 32.3 (bd, J = 33.6 Hz, ***i*Pr-C*H***), 31.3 (***i*Pr-C*H***), 31.2 (***i*Pr-C*H***), 26.5 (dd, J = 28.1, 8.1 Hz, ***i*Pr-C*H*$_3$**), 25.9 (dt, J = 11.2, 6.2 Hz, ***i*Pr-C*H*$_3$**), 25.0 (d, J = 1.9 Hz, ***i*Pr-C*H*$_3$**), 24.8 (***i*Pr-C*H*$_3$**), 24.54 (***i*Pr-C*H*$_3$**), 24.48 (***i*Pr-C*H*$_3$**), 24.25 (***i*Pr-C*H*$_3$**), 24.04 (***i*Pr-C*H*$_3$**).

MS: $[C_{38}H_{54}NP_3+H]^+$ found: 618.3542 (expected: 618.3544)

CHN: (expected) C, 73.88; H, 8.81; N, 2.27; (found) C, 74.52; H, 8.44; N, 1.90.

Even though the C-Value was found to be too high on multiple tries, no significant (soluble organic) impurities were detected by 1H NMR spectroscopy (cf. Figure S48).

[1$_{Dipp}$]$^+$ (R' = Ph)

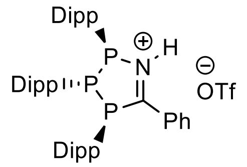

Reaction conducted using 300 mg (0.520 mmol) of P_3Dipp_3. Yield after recrystallization (PhF/*n*-hexane): 306 mg (0.369 mmol, 71%). ^1H NMR (500 MHz, $CDCl_3$): δ 12.61 (d, J = 12.8 Hz, 1H, ***NH***), 7.60 (d, J = 7.9 Hz, 2H, **Ph *o-H***), 7.52 (t, J = 7.7 Hz, 2H, **Ph *p-H*** and **Dipp *p-H***), 7.39 (dt, J = 20.5, 7.8 Hz, 4H, **Ph *p-H*** and two **Dipp *p-H***), 7.31 (dd, J = 7.8, 2.5 Hz, 2H, **Dipp *m-H***), 7.25 (d, J = 7.8 Hz, 2H, **Dipp *m-H***), 7.18 (t, J = 6.9 Hz, 1H, **Dipp *m-H***), 7.09 (d, J = 7.6 Hz, 1H, **Dipp *m-H***), 4.58 (s, 2H, ***i*Pr-C*H***), 4.31 – 4.17 (m, 2H, ***i*Pr-C*H***), 3.95 (tt, J = 13.5, 6.7 Hz, 1H, ***i*Pr-C*H***), 3.71 (hept, J = 5.6 Hz, 1H, ***i*Pr-C*H***), 1.40 (m, 9H, ***i*Pr-C*H*$_3$**), 1.36 (d, J = 6.7 Hz, 3H, ***i*Pr-C*H*$_3$**), 1.09 (d, J = 6.6 Hz, 6H, ***i*Pr-C*H*$_3$**), 0.73 (d, J = 6.7 Hz, 3H, ***i*Pr-C*H*$_3$**), 0.52 (d, J = 6.5 Hz, 3H, ***i*Pr-C*H*$_3$**). **persistent impurity at δ 1.87 (dd, J = 15.7, 5.5 Hz)**

^{19}F NMR (471 MHz, $CDCl_3$): δ -78.53 (***OTf***). **persistent impurity at δ -113.10 ppm**

$^{31}P\{1H\}$ NMR (203 MHz, $CDCl_3$): δ 91.13 (dd, $^1J_{PP}$ = 182.6, $^2J_{PP}$ = 10.7 Hz, $\boldsymbol{P_N}$), 39.02 (dd, $^1J_{PP}$ = 182.6, $^1J_{PP}$ = 176.7 Hz, $\boldsymbol{P_P}$), -2.63 (dd, $^1J_{PP}$ = 176.0, $^2J_{PP}$ = 13.2 Hz, $\boldsymbol{P_C}$). **persistent impurity at δ -3.6 ppm, present after multiple recrystallizations out of PhF/*n*-hexane**

^{13}C NMR (126 MHz, $CDCl_3$): δ 196.4 (d, J = 43.9 Hz, **Ph-*C*=N**), 158.0 (d, J = 14.5 Hz, Dipp-*i*Pr-**C**$_{Ar}$), 156.6 (d, J = 39.3 Hz, Dipp-*i*Pr-**C**$_{Ar}$), 156.3 (d, J = 18.4 Hz, Dipp-*i*Pr-**C**$_{Ar}$), 153.4 (d, J = 5.2

Hz, Dipp-*i*Pr-**C$_{Ar}$**), 135.4 (**Ph *p-C***), 133.7 (**Dipp-*p-ArH***), 133.3 (**Dipp-*p-ArH***), 132.1 (**Dipp-*p-ArH***), 131.7 (bs, ipso ***C*-C=N**), 129.41 (**Dipp-*m-ArH***), 129.29 (d, J = 7.7 Hz, **Ph *o-C***), 126.47 (d, J = 49.3 Hz, ***ipso*-C$_{Ar}$**), 126.39 (**Dipp-*m-ArH***), 125.83 (d, J = 40.0 Hz, ***ipso*-C$_{Ar}$**), 125.49 (**Dipp-*m-ArH***), 125.26 (**Dipp-*m-ArH***), 124.51 (d, J = 9.1 Hz, **Dipp-*m-ArH***), 119.48 (d, J = 43.4 Hz, ***ipso*-C$_{Ar}$**), 35.52 (bs, ***i*Pr-C*H***), 34.04 (d, J = 35.9 Hz, ***i*Pr-C*H***), 33.47 (dt, J = 18.8, 9.5 Hz, ***i*Pr-C*H***), 32.35 (dd, J = 22.9, 4.2 Hz, ***i*Pr-C*H***), 27.16 (***i*Pr-C*H*$_3$**), 25.25 (***i*Pr-C*H*$_3$**), 25.18 (***i*Pr-C*H*$_3$**), 24.78 (***i*Pr-C*H*$_3$**), 24.50 (***i*Pr-C*H*$_3$**), 24.42 (***i*Pr-C*H*$_3$**), 24.26 (***i*Pr-C*H*$_3$**), 23.31 (***i*Pr-C*H*$_3$**).

MS: $C_{43}H_{57}NP_3^+$ found: 680.3714 (expected: 680.3704) (-ESI 149 $CF_3SO_3^-$)

CHN: (expected) C, 63.68; H, 6.92; N, 1.69; S, 3.86; (found) C, 61.75; H, 6.78; N, 1.23; S, 5.05.

Even though the C-Values were found to be too low on multiple tries, no significant (soluble organic) impurities were detected by ^{1}H NMR spectroscopy (cf. Figure S54).

2$_{Dipp}$ (R' = Ph)

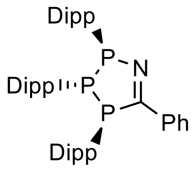

Reaction conducted using 184 mg (0.222 mmol) of **[1$_{Dipp}$]$^+$ (R' = Ph)**. Yield after recrystallization (pentane): 73 mg (0.0881 mmol, 40%).

^{1}H NMR (500 MHz, $CDCl_3$): δ 7.78 (d, J = 7.3 Hz, 2H, **Ph *o-H***), 7.38 – 7.25 (m, 6H, **Ph *m-H***, **Ph *p-H*** and **Dipp *p-H***), 7.21 (dd, J = 7.7, 2.5 Hz, 2H, **Dipp *m-H***), 7.18 (d, J = 7.7 Hz, 2H, **Dipp *m-H***), 7.12 (s, 1H, **Dipp *m-H***), 7.02 (d, J = 7.6 Hz, 1H, **Dipp *m-H***), 4.35 (bs, 1H, ***i*Pr-C*H***), 4.11 (bs, 2H, ***i*Pr-C*H***), 3.66 (dq, J = 12.8, 6.5 Hz, 2H, ***i*Pr-C*H***), 3.62 – 3.55 (m, 1H, ***i*Pr-C*H***), 1.36 (d, J = 6.6 Hz, 3H, ***i*Pr-C*H*$_3$**), 1.27 (dd, J = 6.6, 2.1 Hz, 12H, ***i*Pr-C*H*$_3$**), 1.15 (d, J = 6.6 Hz, 6H, ***i*Pr-C*H*$_3$**), 1.12 (d, J = 6.4 Hz, 3H, ***i*Pr-C*H*$_3$**), 0.99 (d, J = 6.4 Hz, 6H, ***i*Pr-C*H*$_3$**), 0.78 (d, J = 6.6 Hz, 3H, ***i*Pr-C*H*$_3$**), 0.66 (d, J = 6.4 Hz, 3H, ***i*Pr-C*H*$_3$**).

^{31}P{^{1}H} NMR (203 MHz, $CDCl_3$): ABX spin system. δ 100.98 (dd), 25.62 – 21.39 (m).

^{13}C NMR (126 MHz, $CDCl_3$): δ 178.3 (d, J = 45.6 Hz, **Ph-*C*=N**), 156.8 (dd, J = 11.5, 3.6 Hz, two Dipp-*i*Pr-**C$_{Ar}$**), 155.3 (d, J = 37.0 Hz, Dipp-*i*Pr-**C$_{Ar}$**), 154.5 (Dipp-*i*Pr-**C$_{Ar}$**), 152.3 (d, J = 14.5 Hz, two Dipp-*i*Pr-**C$_{Ar}$**), 138.7 (dd, J = 23.7, 7.6 Hz, ***ipso*-C$_{Ar}$**), 135.9 (d, J = 14.4 Hz, ***ipso*-C$_{Ar}$**), 135.5 (dd, J = 11.4, 4.7 Hz, ipso ***C*-C=N**), 132.4 (dd, J = 33.9, 12.9 Hz, ***ipso*-C$_{Ar}$**), 130.7 (**Dipp-*p-ArH***), 130.1 (**Ph *p-C***), 129.9 (**Dipp-*p-ArH***), 129.1 (**Dipp-*p-ArH***), 128.59 (**Ph *o-C***), 128.55 (d, J = 1.9 Hz, **Ph *o-C***), 128.2 (**Ph *m-C***), 125.3 (**Dipp-*m-ArH***), 124.2 (**Dipp-*m-ArH***), 123.4 (d, J = 2.0 Hz, **Dipp-*m-ArH***), 123.2 (d, J = 5.1 Hz, **Dipp-*m-ArH***), 33.2 (ddd, J = 25.1, 16.1, 7.7 Hz, three ***i*Pr-C*H***), 32.4 (dd, J = 35.0, 9.1 Hz, ***i*Pr-C*H***), 31.1 (d, J = 17.2 Hz, two ***i*Pr-C*H***), 25.50 (***i*Pr-C*H*$_3$**), 25.49 (***i*Pr-C*H*$_3$**), 25.4 (***i*Pr-C*H*$_3$**), 25.24 (***i*Pr-C*H*$_3$**), 25.15 (***i*Pr-C*H*$_3$**), 24.4 (***i*Pr-C*H*$_3$**), 24.2 (***i*Pr-C*H*$_3$**), 24.0 (***i*Pr-C*H*$_3$**), 23.6 (***i*Pr-C*H*$_3$**).

MS: $[C_{43}H_{56}NP_3+H]^+$ found: 680.3689 (expected: 680.3699)

CHN: (expected) C, 75.97; H, 8.30; N, 2.06; (found) C, 74.96; H, 8.05; N, 1.72.

No significant impurities were detected by ^{1}H NMR spectroscopy (cf. Figure S61).

[1_{Mes}]$^{+}$ (R' = Me)

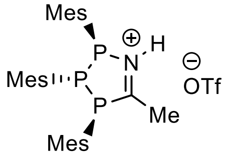

Reaction conducted using 501 mg (1.11 mmol) of P_3Mes_3. Yield after recrystallization (PhF/pentane): 178 mg (0.278 mmol, 25%).

^{1}H NMR (500 MHz, $CDCl_3$): δ 11.77 (s, 1H, ***NH***), 7.02 (s, 1H, **Mes-*ArH***), 7.00 – 6.97 (m, 1H, **Mes-*ArH***), 6.93 (s, 2H, **Mes-*ArH***), 6.89 – 6.86 (m, 2H, **Mes-*ArH***), 2.65 (s, 6H, **Mes-C*H*$_3$**), 2.60 (dd, J = 10.3, 4.4 Hz, 6H, **Mes-C*H*$_3$** and ***H$_3$C*-C=N**), 2.55 (s, 3H, **Mes-C*H*$_3$**), 2.50 (s, 6H, **Mes-C*H*$_3$**), 2.31 (s, 3H, **Mes-C*H*$_3$**), 2.28 (s, 6H, **Mes-C*H*$_3$**). ** impurities at δ 5.73 (d, J = 261.7 Hz), 5.13 (dd, J = 220.2, 4.1 Hz), 2.75 (d, J = 11.5 Hz), 2.44 (s), 1.97 (d, J = 14.0 Hz)**

^{19}F NMR (471 MHz, $CDCl_3$): δ -78.51 (***OTf***).

^{31}P{^{1}H} NMR (203 MHz, $CDCl_3$): δ 91.4 (d, $^{1}J_{PP}$ = 193.4 Hz, $\boldsymbol{P_N}$), 12.1 (dd, $^{1}J_{PP}$ = 216.2, $^{1}J_{PP}$ = 194.2 Hz, $\boldsymbol{P_P}$), 4.0 (dd, $^{1}J_{PP}$ = 216.2, $^{2}J_{PP}$ = 8.4 Hz, $\boldsymbol{P_C}$). **persistent impurities at δ -18.94 (d, J = 261.0 Hz), -59.53 (dt, J = 261.5, 6.7, 3.6 Hz)**

^{13}C NMR (126 MHz, $CDCl_3$): δ 207.9 (d, J = 37.7 Hz, **Me-*C*=N**), 147.1 (d, J = 42.8 Hz, **Mes-C$_{Ar}$**), 146.2 (d, J = 17.2 Hz, **Mes-C$_{Ar}$**), 143.9 (**Mes-C$_{Ar}$**), 143.3 (**Mes-C$_{Ar}$**), 143.0 (d, J = 8.2 Hz, **Mes-C$_{Ar}$**), 142.3 (**Mes-C$_{Ar}$**), 142.2 (**Mes-C$_{Ar}$**), 142.1 (**Mes-C$_{Ar}$**), 141.8 (**Mes-C$_{Ar}$**), 131.1 (d, J = 3.4 Hz, two **Mes-*ArH***), 131.0 (d, J = 4.0 Hz, two **Mes-*ArH***), 130.5 (d, J = 68.0 Hz, two **Mes-*ArH***), 126.5 – 125.7 (m, ***ipso*-C$_{Ar}$**), 122.1 (dd, J = 21.8, 12.3 Hz, ***ipso*-C$_{Ar}$**), 119.4 (d, J = 23.2 Hz, ***ipso*-C$_{Ar}$**), 25.2 – 24.9 (m, **Mes-C*H*$_3$**), 24.9 – 24.7 (m, **Mes-C*H*$_3$**), 24.3 (dd, J = 12.5, 5.3 Hz, ***H$_3$C*-C=N**), 24.1 (**Mes-C*H*$_3$**), 22.9 (dd, J = 18.6, 3.3 Hz, **Mes-C*H*$_3$**), 21.2 (**Mes-C*H*$_3$**), 21.1 (**Mes-C*H*$_3$**), 21.0 (**Mes-C*H*$_3$**).

MS: $C_{29}H_{37}NP_3^{+}$ found: 492.2129 (expected: 429.2134) (-ESI 149 $CF_3SO_3^{-}$)

Satisfactory CHN data for this sample was not acquired.

2_{Mes} (R' = Me)

In a nitrogen-filled glovebox, a 3 mL vial was charged with 120 mg (0.187 mmol) of **[1_{Mes}] (R' = Me)** and 2 mL of *n*-hexane. 1.1 equivalents of NEt_3 (21 mg, 0.21 mmol) were added, and the reaction mixture was stirred for 18 h. The reaction was then stored at -30 °C for 24 h to allow for the precipitation of [$HNEt_3$][OTf] from *n*-hexane, and the supernatant was filtered through a 0.2 μm Teflon syringe filter. The *n*-hexane was pumped down to concentrate the solution and the solution was placed at -30 °C for 15 days, however no crystallization was initiated and instead a pale yellow oil was seen along the sides of the vial. This material contaminated with as-of-yet

unknown phosphorus impurities and [$HNEt_3$][OTf] and therefore was not suitable for analytical characterization. Volatiles were removed *in vacuo* and the oil was extracted with minimal *n*-pentane; however, storage of this solution for an extended time at -30 °C resulted in no crystal formation. Finally, volatiles were again removed *in vacuo,* and the resultant oil was dissolved in minimal acetonitrile and stored at -30 °C for 24 days, after which a small crop of colourless, transparent crystals were seen growing along the edge of the solvent line, which were suitable for single crystal X-Ray diffraction studies.

Yield after crystallization: 92 mg (0.110 mmol, 59%).

^{1}H NMR (500 MHz, $CDCl_3$) δ 6.93 (s, 3H, **Mes-*ArH***), 6.83 (d, 3H, **Mes-*ArH***), 2.67 (s, 3H, **Mes-C*H*$_3$**), 2.61 (s, 6H, **Mes-C*H*$_3$**), 2.44 (s, 6H, **Mes-C*H*$_3$**), 2.35 (dd, J = 7.3, 4.5 Hz, 3H, ***H*$_3$*C*-C=N**), 2.30 (s, 3H, **Mes-C*H*$_3$**), 2.29 (s, 3H, **Mes-C*H*$_3$**), 2.27 (s, 3H, **Mes-C*H*$_3$**), 2.14 (s, 3H, **Mes-C*H*$_3$**).

^{31}P NMR (203 MHz, $CDCl_3$) δ 113.9 (d, $^{1}J_{PP}$ = 242.6 Hz, $\boldsymbol{P_N}$), 22.1 (d, $^{1}J_{PP}$ = 278.4 Hz, $\boldsymbol{P_C}$), -6.4 (dd, $^{1}J_{PP}$ = 278.5, $^{1}J_{PP}$ = 242.4 Hz, $\boldsymbol{P_P}$).

^{13}C NMR (126 MHz, $CDCl_3$) δ 185.44 (ddd, J = 47.8, 9.1, 2.5 Hz, **H$_3$C-*C*z=N**), 145.88 (d, J = 40.8 Hz, **Mes-C$_{Ar}$**), 145.43 (dd, J = 15.1, 3.2 Hz, **Mes-C$_{Ar}$**), 143.62 (**Mes-C$_{Ar}$**), 140.21 (**Mes-C$_{Ar}$**), 140.09 (dd, J = 14.4, 3.0 Hz, **Mes-C$_{Ar}$**), 138.96 (**Mes-C$_{Ar}$**), 137.53 (d, J = 2.0 Hz, **Mes-C$_{Ar}$**), 133.16 (PN-Cipso, dd, J = 40.5, 12.0 Hz), 130.24, 130.00 (d, J = 4.2 Hz), 129.48 (d, J = 53.8 Hz), 128.27 (dd, J = 29.5, 14.8 Hz, ***ipso*-C$_{Ar}$**), 127.86 (ddd, J = 32.6, 21.2, 18.0 Hz, two ***ipso*-C$_{Ar}$**), 25.37 (dd, J = 35.6, 10.2 Hz, ***H*$_3$*C*-C=N**), 24.52 (d, J = 32.5 Hz, **Mes-C*H*$_3$**), 23.82 (dt, J = 15.6, 10.2 Hz, two **Mes-C*H*$_3$**), 23.36 (dd, J = 14.0, 3.7 Hz, two **Mes-C*H*$_3$**), 23.22 (**Mes-C*H*$_3$**), 21.09 (**Mes-C*H*$_3$**), 21.02 (**Mes-C*H*$_3$**), 20.84 (**Mes-C*H*$_3$**).

No MS and CHN data were collected due to the high sensitivity of the sample.

[1$_{Mes}$]$^+$ (R' = Ph)

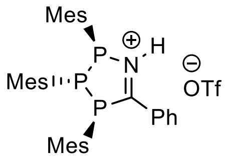

Reaction conducted using 500 mg (1.11 mmol) of P_3Mes_3. Yield after recrystallization (PhF/*n*-hexane): 141 mg (0.200 mmol, 18%).

^{1}H NMR (500 MHz, $CDCl_3$) δ 12.75 (s, 1H, ***NH***), 7.76 (d, J = 7.8 Hz, 2H, **Ph *o-H***), 7.58 (t, J = 7.4 Hz, 1H, **Ph *p-H***), 7.44 (t, J = 7.8 Hz, 2H, **Ph *m-H***), 6.95 (s, 2H, **Mes-*ArH***), 6.89 (s, 3H, **Mes-*ArH***), 6.77 (s, 1H, **Mes-*ArH***), 2.67 (s, 6H, **Mes-C*H*$_3$**), 2.59 (s, 9H, **Mes-C*H*$_3$**), 2.34 (d, J = 3.8 Hz, 3H, **Mes-C*H*$_3$**), 2.29 (d, J = 3.0 Hz, 6H, **Mes-C*H*$_3$**), 2.21 (s, 3H, **Mes-C*H*$_3$**).

^{19}F NMR (471 MHz, $CDCl_3$) δ -78.46 (***OTf***).

^{31}P NMR (203 MHz, $CDCl_3$) δ 92.9 (d, $^{1}J_{PP}$ = 197.3 Hz, $\boldsymbol{P_N}$), 1.6 (dd, $^{1}J_{PP}$ = 215.3, $^{1}J_{PP}$ = 200.6 Hz, $\boldsymbol{P_P}$), -2.5 (d, $^{1}J_{PP}$ = 217.1 Hz, $\boldsymbol{P_C}$).

^{13}C NMR (126 MHz, $CDCl_3$) δ 200.20 (**Me-*C*=N**), 146.32 (d, J = 19.0 Hz, **Mes-C_{Ar}**), 143.27, 141.89 (d, J = 4.8 Hz, **Mes-C_{Ar}**), 141.75 (d, J = 19.1 Hz, **Mes-C_{Ar}**), 135.70 (**Mes-C_{Ar}**), 132.28 (d, J = 11.5 Hz, ***ipso*-Ph**), 131.11 (**Mes-*ArH***), 130.07 (d, J = 7.9 Hz, **Mes-*ArH***), 129.86 (**Mes-*ArH***), 129.51 (d, J = 9.2 Hz, **Mes-*ArH***), 128.00 (dd, J = 48.0, 11.2 Hz, ***ipso*-C_{Ar}**), 125.77 (d, J = 33.5 Hz, ***ipso*-C_{Ar}**), 119.85 (dd, J = 35.4, 15.8 Hz, ***ipso*-C_{Ar}**), 25.07 (dt, J = 16.4, 10.7 Hz, **Mes-C*H_3***), 24.56 – 23.93 (m, **Mes-C*H_3***), 23.13 (dd, J = 18.4, 2.3 Hz, **Mes-C*H_3***), 21.30 (**Mes-C*H_3***), 21.20 (**Mes-C*H_3***).

MS: Ion peak was not detected by ESI-MS.

CHN: (expected) C, 59.74; H, 5.59; N, 1.99; S, 4.56; (found) C, 55.36; H, 5.57; N, 1.40; S, 5.38.

In solution **$[1_{Mes}]^+$ (R' = Ph)** is prone to form P_4Mes_4 and its presence in the solid state cannot be excluded, which is in line with C-values that were too low even on repeated measurements.

$[1_{tBu}]^+$ (R' = Ph)

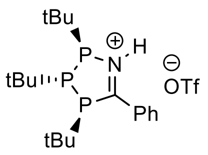

Reaction conducted using 265 mg (0.100 mmol) of $P_3{}^tBu_3$. Yield after recrystallization (DCM/*n*-hexane): 483 mg (0.000933 mmol, 93%).

^{1}H NMR (500 MHz, $CDCl_3$) δ 12.82 (s, 1H, ***NH***), 7.75 (d, J = 8.2 Hz, 2H, **Ph *o-H***), 7.70 (t, J = 7.4 Hz, 1H, **Ph *p-H***), 7.57 (t, J = 7.8 Hz, 2H, **Ph *m-H***), 1.36 (d, J = 15.2 Hz, 9H, ***^{t}Bu***), 1.31 (d, J = 14.5 Hz, 9H, ***^{t}Bu***), 1.12 (d, J = 15.6 Hz, 9H, ***^{t}Bu***).

^{19}F NMR (471 MHz, $CDCl_3$) δ -78.52 (***OTf***).

^{31}P NMR (203 MHz, $CDCl_3$) δ 133.39 (d, $^1J_{PP}$ = 278.9 Hz, $\boldsymbol{P_N}$), 56.81 (br d, $\boldsymbol{P_P}$), -10.53 (br s, $\boldsymbol{P_C}$).

^{13}C NMR (126 MHz, $CDCl_3$) δ 135.06 (d, J = 16.2 Hz, **Ph-*p*-C_{Ar}**), 130.32 (d, J = 9.2 Hz, **Ph-*o*-C_{Ar}**), 129.35 (s, **Ph-*p*-C_{Ar})**, 37.52 (d, J = 18.5 Hz, ***^{t}Bu***), 35.89 (d, J = 33.8 Hz, ***^{t}Bu***), 30.79 (dd, J = 12.3, 5.0 Hz, ***^{t}Bu***), 30.52 – 30.12 (m, ***^{t}Bu***), 27.02 (dd, J = 15.7, 5.5 Hz, ***^{t}Bu***). * quaternary **Ph-*C*=N** carbon was not observed in either direct observation or 2-D HMBC spectra*

MS: $C_{19}H_{33}NP_3{}^+$ found: 368.1820 (expected: 368.1821) (-ESI 149 $CF_3SO_3{}^-$)

CHN: (expected) C, 46.42; H, 6.43; N, 2.71; S, 6.20; (found) C, 47.41; H, 6.10; N, 2.44; S, 6.53.

No significant impurities were detected by ^{1}H NMR spectroscopy (cf. Figure S88).

[1_{Tipp}]$^+$ (R' = *p*-MeOC_6H_4)

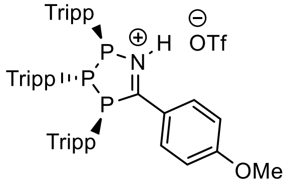

Reaction conducted using 350 mg (0.498 mmol) of P_3Tipp_3. Yield after recrystallization (PhF/*n*-hexane): 452 mg (0.458 mmol, 92%).

^{1}H NMR (500 MHz, $CDCl_3$) δ 11.94 (d, J = 13.4 Hz, 1H, ***NH***), 7.63 (d, J = 7.8 Hz, 2H, **MeOPh *o-H***), 7.14 (s, 2H, **Tipp-*ArH***), 7.04 (s, 2H, **Tipp-*ArH***), 6.97 (d, J = 5.0 Hz, 1H, **Tipp-*ArH***), 6.94 (s, 1H, **Tipp-*ArH***), 6.84 (d, J = 7.9 Hz, 2H, **Ph *m-H***), 4.57 (s, 2H, ***i*Pr-C*H***), 4.39 – 4.26 (m, 2H, ***i*Pr-C*H***), 3.96 – 3.85 (m, 1H, ***i*Pr-C*H***), 3.81 (s, 3H, **OC*H₃***), 3.77 – 3.69 (m, 1H, ***i*Pr-C*H***), 2.96 – 2.86 (m, 1H, ***i*Pr-C*H***), 2.86 – 2.76 (m, 2H, ***i*Pr-C*H***), 1.43 – 1.35 (m, 9H, ***i*Pr-C*H₃***), 1.31 (d, J = 5.9 Hz, 3H, ***i*Pr-C*H₃***), 1.29 – 1.23 (m, 12H, ***i*Pr-C*H₃***), 1.23 – 1.14 (m, 18H, ***i*Pr-C*H₃***), 0.99 (d, 6H, ***i*Pr-C*H₃***), 0.66 (d, J = 5.9 Hz, 3H, ***i*Pr-C*H₃***), 0.51 (d, J = 5.8 Hz, 3H, ***i*Pr-C*H₃***).

^{19}F NMR (471 MHz, $CDCl_3$) δ -78.52 (***OTf***).

^{31}P NMR (203 MHz, $CDCl_3$) δ 86.6 (dd, $^1J_{PP}$ = 185.2, $^2J_{PP}$ = 16.3 Hz, $\boldsymbol{P_N}$), 34.10 (dd, $^1J_{PP}$ = 185.0, $^1J_{PP}$ = 163.3 Hz, $\boldsymbol{P_P}$), -9.26 (dd, $^1J_{PP}$ = 163.0, $^2J_{PP}$ = 16.6 Hz, $\boldsymbol{P_C}$).

^{13}C NMR (126 MHz, $CDCl_3$) δ 193.27 (d, J = 43.8 Hz, **Ph-*C*=N**), 165.57 (**C**-OMe), 158.01 (d, J = 12.2 Hz, Tipp-*i*Pr-**C**$_{Ar}$), 156.85 (d, J = 18.9 Hz, Tipp-*i*Pr-**C**$_{Ar}$), 156.46 (d, J = 38.1 Hz, Tipp-*i*Pr-**C**$_{Ar}$), 154.58 (Tipp-*i*Pr-**C**$_{Ar}$), 154.10 (Tipp-*i*Pr-**C**$_{Ar}$), 153.54 (d, J = 5.5 Hz, Tipp-*i*Pr-**C**$_{Ar}$), 152.64 (Tipp-*i*Pr-**C**$_{Ar}$), 148.74 (d, J = 15.4 Hz, Tipp-*i*Pr-**C**$_{Ar}$), 132.18 (d, J = 7.2 Hz, **Ph *o*-C**), 124.81 (**Tipp-*m*-C**$_{Ar}$), 124.15 (d, J = 38.1 Hz, ***ipso*-C**$_{Ar}$), 123.74 (**Tipp-*m*-C**$_{Ar}$), 123.63 (**Tipp-*m*-C**$_{Ar}$), 122.66 (dd, J = 52.3, 9.3 Hz, ***ipso*-C**$_{Ar}$), 122.59 (d, J = 9.1 Hz, **Tipp-*m*-C**$_{Ar}$), 116.84 (d, J = 45.1 Hz, ***ipso*-C**$_{Ar}$), 115.02 (**Ph *o*-Cf**), 56.02 (O**C**H_3), 35.43 (*i*Pr_2CH), 34.67 (*i*Pr_2**C**H), 34.41 (*i*Pr_2**C**H), 33.84 (d, J = 36.4 Hz, *i*Pr_2CH), 33.69 (*i*Pr_2CH), 33.35 (dt, J = 18.1, 8.4 Hz, *i*Pr_2**C**H), 32.32 (dd, J = 21.7, 3.9 Hz, *i*Pr_2**C**H), 27.19 (Ar-CH-**C**H_3), 25.47 (Ar-CH-**C**H_3), 24.87 (Ar-CH-**C**H_3), 24.76 (Ar-CH-**C**H_3), 24.26 (Ar-CH-**C**H_3), 23.80 (Ar-CH-**C**H_3), 23.71 (Ar-CH-**C**H_3), 23.61 (Ar-CH-**C**H_3).

MS: $C_{53}H_{77}NP_3^+$ found: 836.5222 (expected: 836.5213.1821) (-ESI 149 $CF_3SO_3^-$)

CHN: (expected) C, 65.77; H, 7.87; N, 1.42; S, 3.25; (found) C, 65.79; H, 7.27; N, 1.22; S, 3.51.

[1_{Tipp}]$^+$ (R' = *p*-$CF_3C_6H_4$)

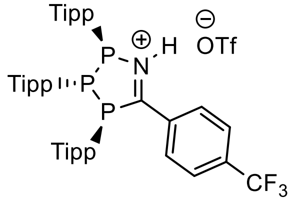

Reaction conducted using 350 mg (0.498 mmol) of P_3Tipp_3.Yield after recrystallization (PhF/*n*-hexane): 433 mg (0.423 mmol, 85%).

It should be noted that **[1_{Tipp}] (R' = *p*-$CF_3C_6H_4$)** was significantly more lipophilic, and therefore, difficult to fully dry, than other Tipp species, and showed significant decomposition if left in dilute solution, at reduced or ambient temperatures, for longer than 24 h.

^{1}H NMR (500 MHz, $CDCl_3$) δ 12.84 (d, J = 10.9 Hz, 1H, ***NH***), 7.72 (d, J = 7.3 Hz, 2H, **CF_3Ph *o-H***), 7.64 (d, J = 7.5 Hz, 2H, **Ph *m-H***), 7.16 (s, 2H**, Tipp-ArH**), 7.07 (s, 2H**, Tipp-ArH**), 7.00 (d, 1H**, Tipp-ArH**), 6.93 (s, 1H**, Tipp-ArH**), 4.59 (s, 2H, ***i*Pr-C*H***), 4.43 – 4.26 (m, 2H, ***i*Pr-C*H***), 3.96 – 3.82 (m, 1H, ***i*Pr-C*H***), 3.81 – 3.70 (m, 1H, ***i*Pr-C*H***), 2.98 – 2.88 (m, 1H, ***i*Pr-C*H***), 2.87 – 2.77 (m, 2H, ***i*Pr-C*H***), 1.45 – 1.37 (m, 9H, ***i*Pr-C*H₃***), 1.34 (d, J = 5.9 Hz, 3H, ***i*Pr-C*H₃***), 1.26 (d, J = 6.1 Hz, 12H, ***i*Pr-C*H₃***), 1.21 (s, 9H, ***i*Pr-C*H₃***), 1.18 (dd, J = 12.0, 5.4 Hz, 9H, ***i*Pr-C*H₃***), 1.09 – 1.01 (m, 6H, ***i*Pr-C*H₃***), 0.72 (d, J = 5.9 Hz, 3H, ***i*Pr-C*H₃***), 0.52 (d, J = 5.8 Hz, 3H, ***i*Pr-C*H₃***).

^{19}F NMR (471 MHz, $CDCl_3$) δ -63.45 (C_6H_4C***F_3***), -78.63 (**OTf**).

^{31}P NMR (203 MHz, $CDCl_3$) δ 89.12 (dd, $^1J_{PP}$ = 181.7, $^2J_{PP}$ = 15.8 Hz, ***P_N***), 50.66 (dd, $^1J_{PP}$ = 180.4, $^1J_{PP}$ = 172.9 Hz, ***P_P***), -2.38 (dd, $^1J_{PP}$ = 171.7, $^2J_{PP}$ = 17.4 Hz, ***P_C***).

^{13}C NMR (126 MHz, $CDCl_3$) δ 194.66 (d as determined by HMBC, J = 36.6 Hz, **Ph-*C*=N**), 157.95 (d, J = 13.8 Hz, Tipp-*i*Pr-**C_{Ar}**), 157.01 (Tipp-*i*Pr-**C_{Ar}**), 156.91 (Tipp-*i*Pr-**C_{Ar}**), 156.73 (d, J = 5.2 Hz, Tipp-*i*Pr-**C_{Ar}**), 155.10 (Tipp-*i*Pr-**C_{Ar}**), 154.72 (Tipp-*i*Pr-**C_{Ar}**), 153.21 (d, J = 5.3 Hz, Tipp-*i*Pr-**C_{Ar}**), 153.00 (Tipp-*i*Pr-**C_{Ar}**), 135.70 (d, J = 34.2 Hz, ***C*-CF_3**), 134.72 (**C-*C*F_3**)f, 129.42 (d, **Ph *o*-C**), 126.32 (d, **Ph *m*-C**), 124.74 (**Tipp-*m*-C_{Ar}**), 123.84 (**Tipp-*m*-C_{Ar}**), 123.68 (**Tipp-*m*-C_{Ar}**), 122.77 (d, J = 9.1 Hz, **Tipp-*m*-C_{Ar}**), 122.80 (d, J = 44.3 Hz, ***ipso*-C_{Ar}**), 122.23 (d, J = 50.0 Hz, ***ipso*-C_{Ar}**), 115.76 (d, 57.6 Hz, ***ipso*-C_{Ar}**), 35.45 (*i*Pr_2**C**H), 34.57 (*i*Pr_2**C**H), 34.07 (d, J = 35.4 Hz, *i*Pr_2**C**H), 33.44 (br m, *i*Pr_2**C**H), 32.50 (br d, J = 22.6 Hz, *i*Pr_2**C**H), 27.10 (Ar-CH-**C**H_3), 25.24 (d, J = 7.9 Hz, Ar-CH-**C**H_3), 24.74 (Ar-CH-**C**H_3), 24.69 (Ar-CH-**C**H_3), 24.30 (Ar-CH-**C**H_3), 24.17 (Ar-CH-**C**H_3), 23.62 (Ar-CH-**C**H_3), 23.56 (Ar-CH-**C**H_3), 23.54 (Ar-CH-**C**H_3), 23.49 (Ar-CH-**C**H_3), 23.43 (Ar-CH-**C**H_3), 23.39 (Ar-CH-**C**H_3).

MS: Ion peak was not detected by ESI-MS.

CHN: (expected) C, 66.58; H, 7.91; N, 1.46; S, 3.35; (found) C, 66.22; H, 7.30; N, 1.20; S, 3.51.

Poly(4-cyanostyrene) (P4CS)

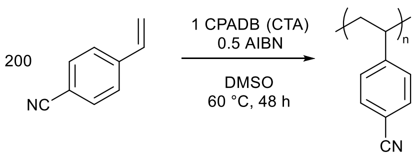

Poly[(4-cyanostyrene) (**P4CS**)

Poly(4-cyanostyrene) was synthesized by a RAFT polymerization according to an adapted literature procedure.[5] 4-cyanostyrene (1.5 g, 11.6 mmol), 4-Cyano-4-(phenylcarbonothioylthio)pentanoic acid (CPADB, chain transfer agent (CTA), 16.2 mg, 0.06 mmol) and azobisisobuyronitrile (AIBN, initiator, 4.8 mg, 0.029 mmol) were dissolved in 10 mL of DMSO ([Monomer]/[CTA]/[Initiator]=200/1/0.25). The reaction mixture was then degassed by three consecutive freeze-pump-thaw cycles and the system was then back-filled with argon. The solution was heated to 60 °C for 2 days. The resulting pink viscous reaction mixture was added dropwise to 200 mL of stirring *n*-hexane, from which a viscous oily material was obtained. This material was taken up in a minimal amount of DMSO and added dropwise to 300 mL of methanol, from which a pale pink precipitate was obtained. The supernatent was removed by decantation off and the material was triturated with 3 × 300 mL methanol, yielding the title polymer. GPC: M_n = 109.4 kDa, $Đ$ = 1.14.

Photograph of the isolated polymer **P4CS**:

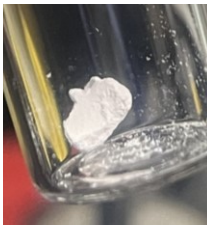

Poly(4-cyanostyrene)-co-([1_{Tipp}H][OTf] R'=Ph)

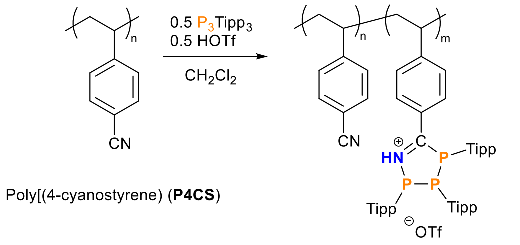

P4CS-co-([1_{Tipp}H][OTf] R=Ph)

Poly(4-cyanostyrene) (129 mg, 9.99 mmol of repeat units) was suspended in a 5 mL CH_2Cl_2 solution of P_3Tipp_3 (351 mg, 4.99 mmol) at 22 °C. To the stirring suspension, 44 µL of HOTf (4.99 mmol) was added in one portion, leading to the immediate formation of a purple suspension. The suspension was allowed to stir for 12 hours, after which 15 mL of *n*-hexane was added. The purple precipitate was isolated by centrifugation and washed with *n*-hexane to afford purple beads of

poly[(4-cyanostyrene)-co-(**[1$_{Tipp}$H][OTf] R'=Ph**) (**P4CS-co-([1$_{Tipp}$H][OTf] R'=Ph**). The polymer was insoluble in conventional solvents and was used as isolated for the synthesis of poly[(4-cyanostyrene)-co-(**2Tipp R=Ph**). Isolated yield: 410 mg, 74%.

Photograph of the isolated polymer **P4CS-co-([1$_{Tipp}$H][OTf] R'=Ph**:

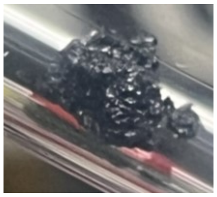

Poly(4-cyanostyrene)-co-([2$_{Tipp}$ R'=Ph)

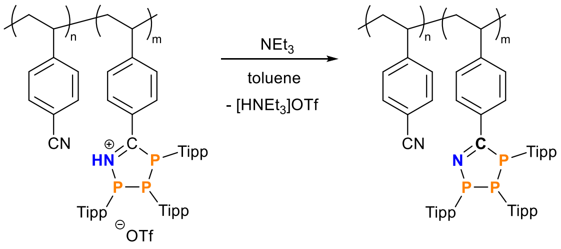

P4CS-co-([1$_{Tipp}$H][OTf] R'=Ph) **P4CS-co-(2$_{Tipp}$ R'=Ph)**

Poly[(4-cyanostyrene)-co-(**[1$_{Tipp}$H][OTf] R'=Ph**)] (150 mg, 0.135 mmol) was suspended in 3 mL of toluene, and NEt_3 (500 µL, 3.5 mmol, 26.6 eq) was added to the suspension in one portion at 22 °C. The polymer beads began to turn orange on the exterior, and the suspension was allowed to stir for 24 hours. This yielded an orange solution, from which the volatiles were removed *in vacuo,* and the resulting material was washed with copious amounts of acetonitrile, to yield an orange powder of poly[(4-cyanostyrene)-co-(**2Tripp R=Ph**)] (115 mg, 88 %). We note a so far unidentified impurity (δ(^{31}P) = 59.6 ppm) in the isolated material, which is likely trapped within the polymer matrix. The polymer also bears many phosphorus lone pairs which may bind to the GPC column, resulting in a broad retention volume and large polydispersity compared to the as-synthesized poly(4-cyanostyrene). GPC: M_n = 185.6 kDa, $Đ$ = 3.87.

^{31}P NMR (203 MHz, $CDCl_3$) δ 87.9 (br, ***P$_N$***), 30.5 (br, ***P$_P$***), 16.0 (br, ***P$_C$***) (see ^{31}P NMR spectrum of **P4CS-co-([2$_{Tipp}$ R'=Ph)** in $CDCl_3$ overlayed with that of isolated **2$_{Tipp}$ (R' = Ph)** (blue) below)

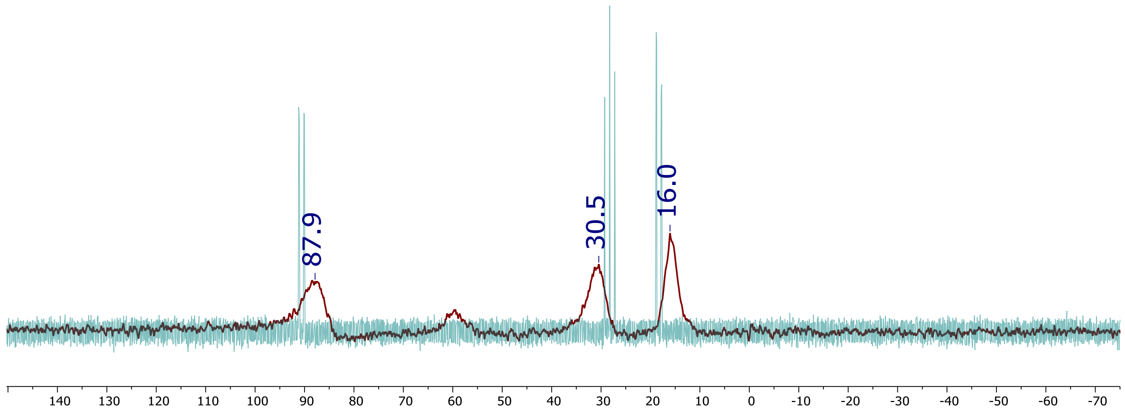

Figure S0. ^{31}P NMR spectrum of **P4CS-co-([2$_{Tipp}$ R'=Ph)** in $CDCl_3$ overlayed with that of isolated **2$_{Tipp}$ (R' = Ph)** (blue) below.

Acid-Base sensing with Poly[(4-cyanostyrene)-co-(2$_{Tipp}$ R'=Ph)

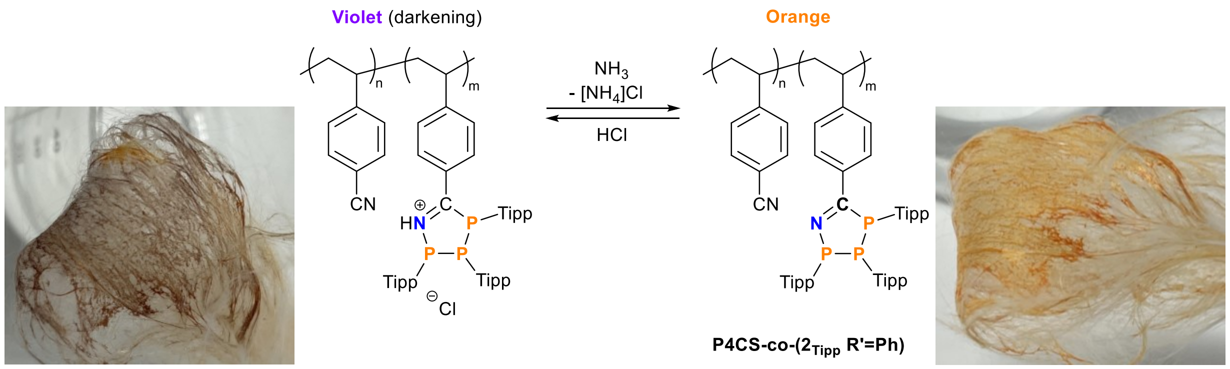

50 mg of poly[(4-cyanostyrene)-co-(**2$_{Tipp}$ R'=Ph**)] was dissolved in 5 mL of toluene in a 20 mL scintillation vial. Glass wool, dried at 200 °C for 24 h, was then dipped into the vial and then the fibers were spread apart to prevent aggregation of fibres upon toluene evaporation. The wool was allowed to dry for 24 h, yielding pale orange fibres with a few agglomerations. This material can be freely handled in air with no evidence of degradation. The fibres were bound together with scotch tape and placed in the headspace of beakers containing either 35% $HCl_{(aq)}$ or saturated aqueous ammonium hydroxide, and colour changes indicative of formation of Poly[(4-cyanostyrene)-co-(**[1$_{Tipp}$-H][Cl] R=Ph**)] (purple) or Poly[(4-cyanostyrene)-co-(**2$_{Tipp}$ R=Ph**)] (orange) were noted. The fibres could be cycled more than 20 times, during which we did not observe a change in intensity of the fibre colour changes or evidence of $[NH_4][Cl]$ build up on the fibres.

A video showing the sensing experiment has been added as additional supporting material.

2. NMR Spectra

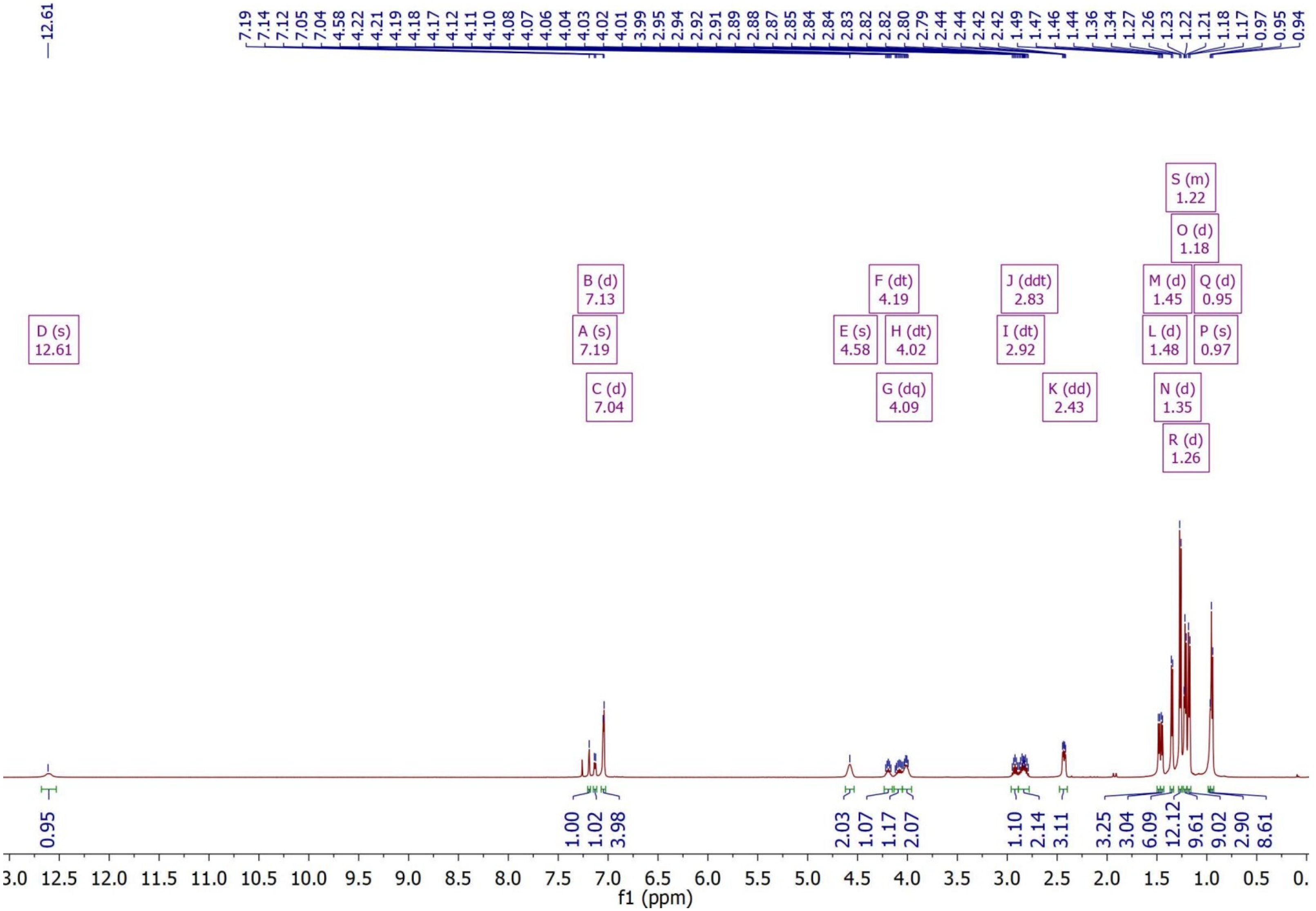

Figure S-1 ^{1}H NMR spectrum ($CDCl_3$) of compound $[1_{Tipp}]^{+}$ (R' = Me)

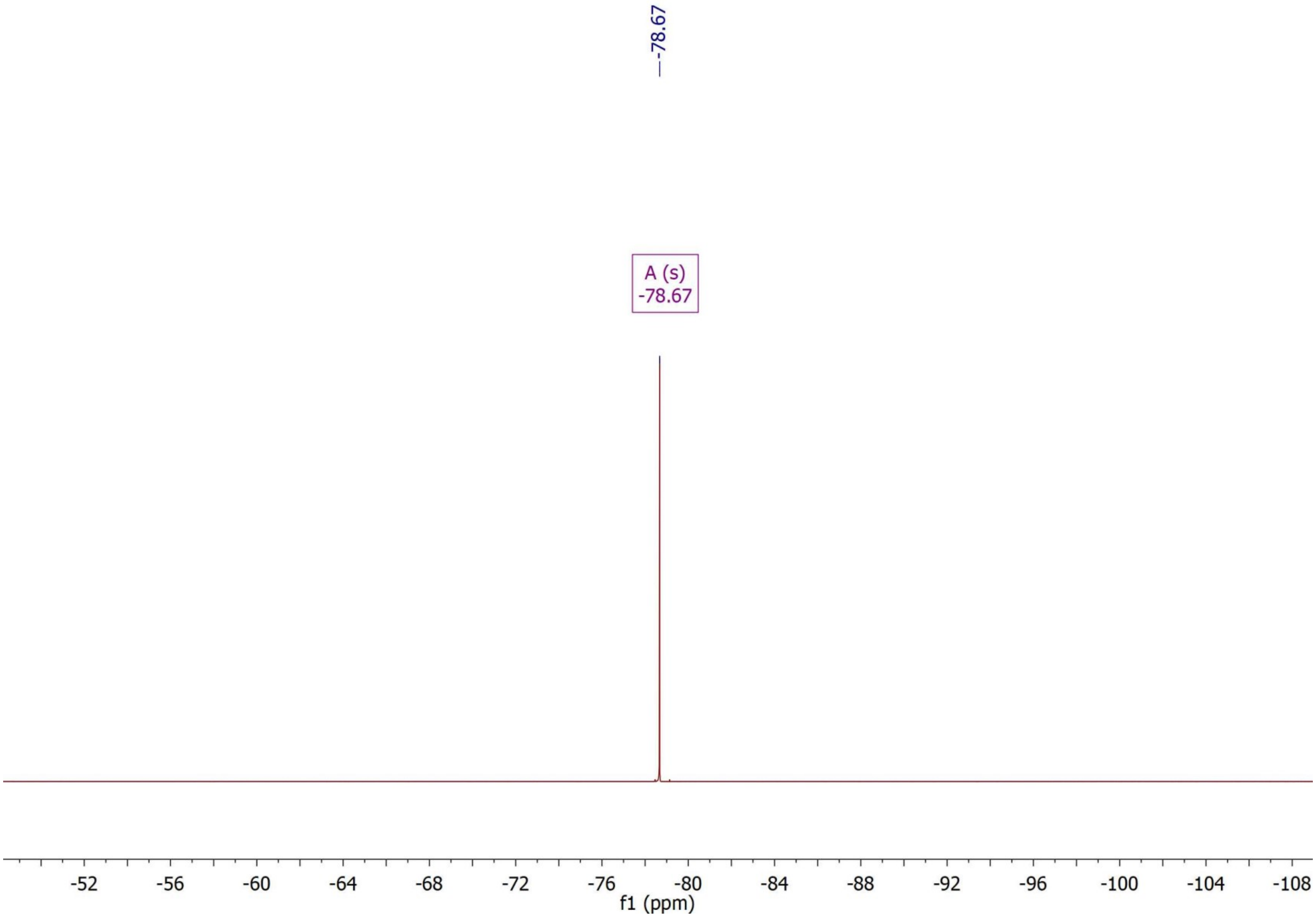

Figure S-2 ^{19}F NMR spectrum ($CDCl_3$) of compound $[1_{Tipp}]^+$ (R' = Me)

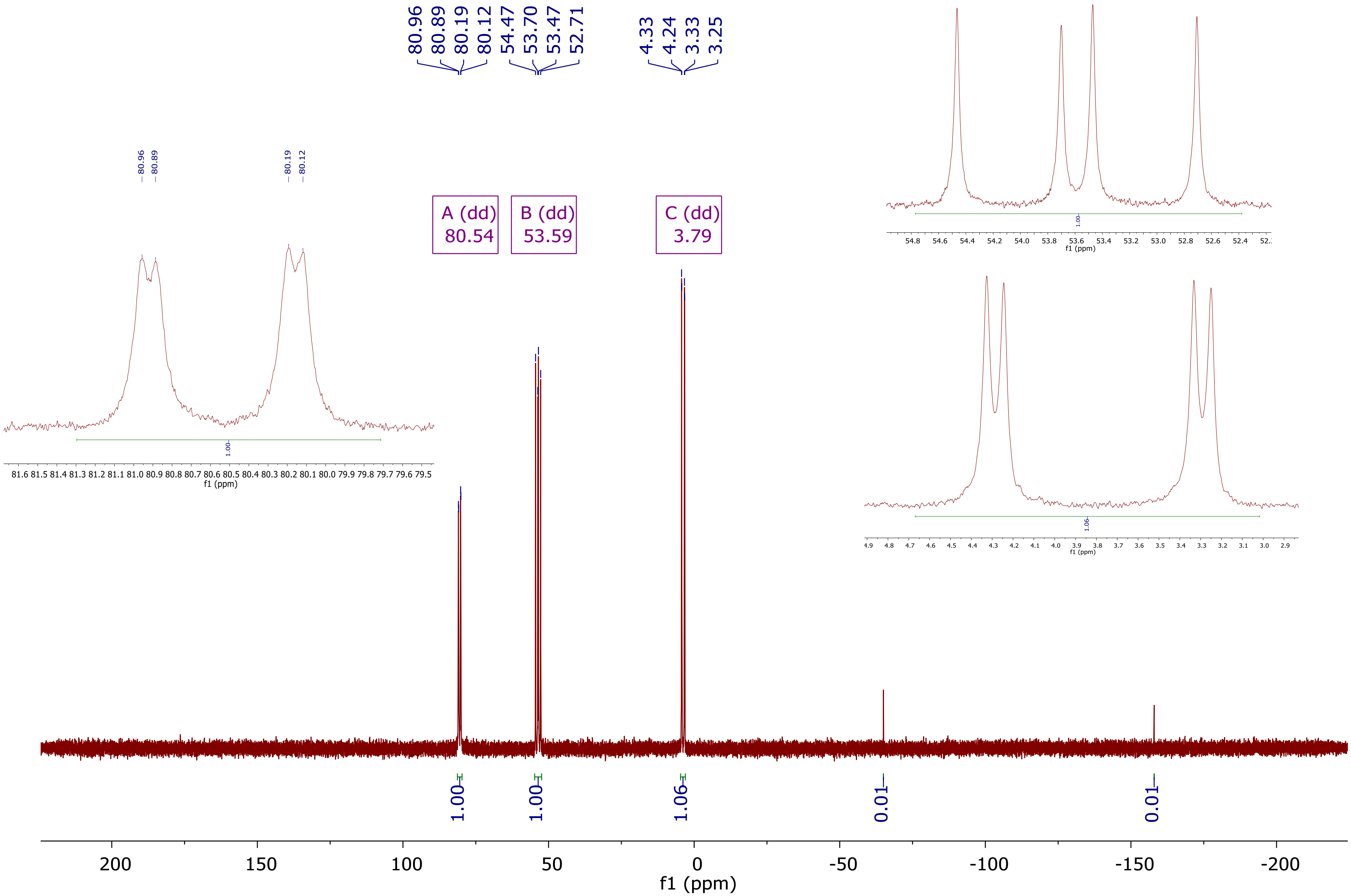

Figure S-3 $^{31}P\{^{1}H\}$ NMR spectrum (CDCl$_3$) of compound $[1_{Tipp}]^{+}$ (R' = Me)

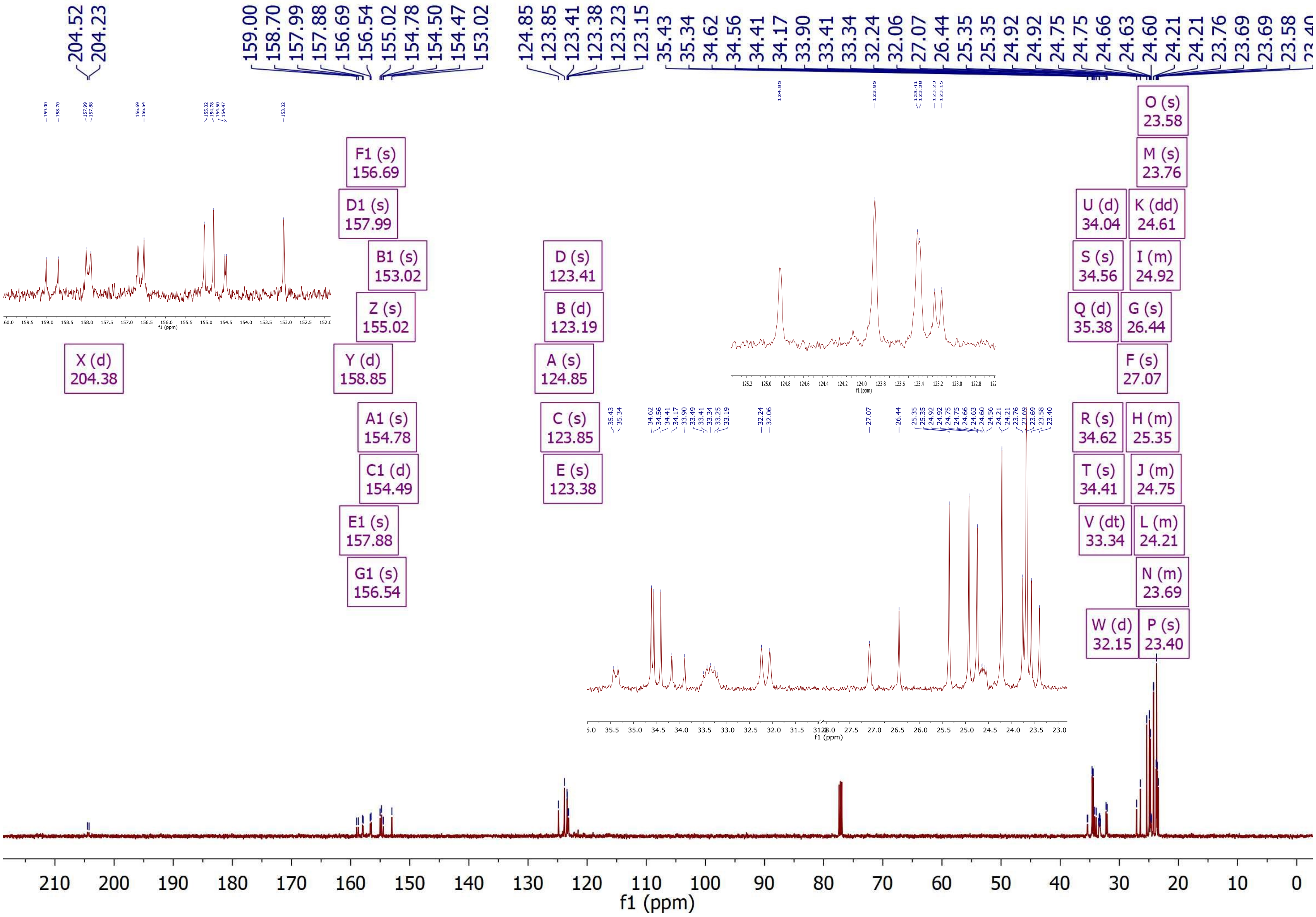

Figure S-4 $^{13}C\{^{1}H\}$ **UDEFT NMR spectrum (CDCl₃) of compound** $[1_{Tipp}]^{+}$ **(R' = Me)**

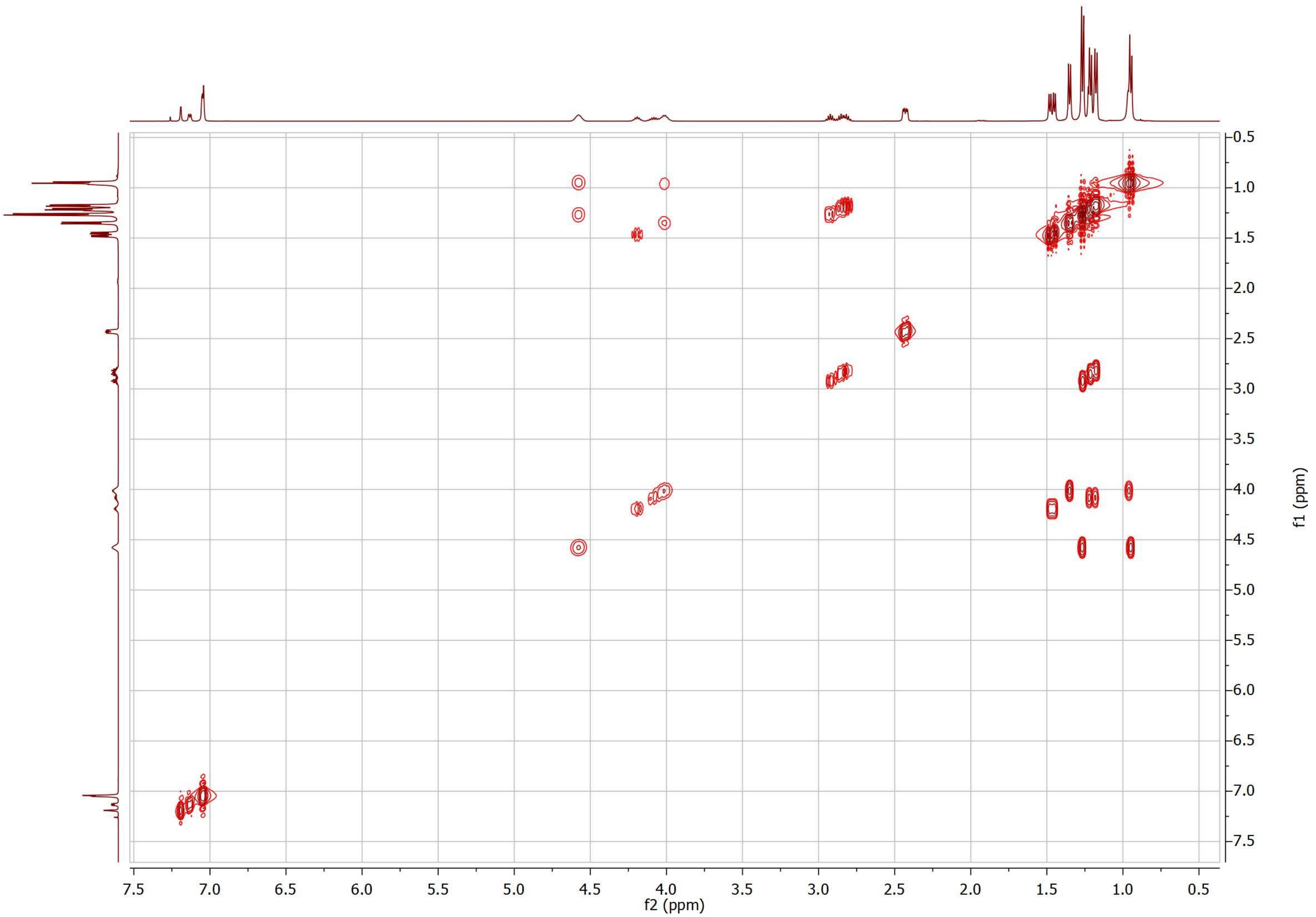

Figure S-5 ^{1}H COSY NMR spectrum ($CDCl_3$) of compound $[1_{Tipp}]^{+}$ (R' = Me)

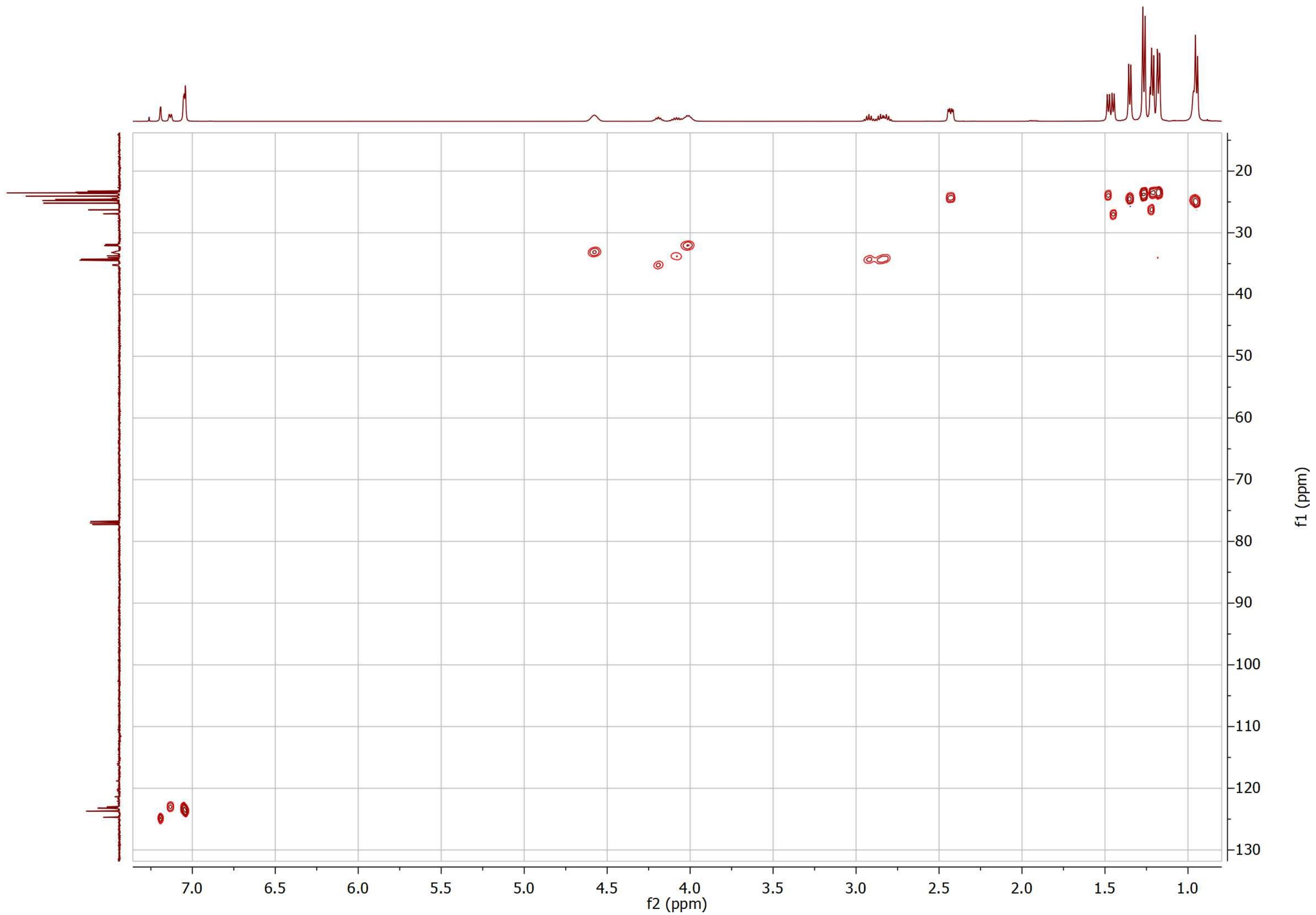

Figure S-6 HSQC NMR spectrum ($CDCl_3$) of compound $[1_{Tipp}]^+$ (R' = Me)

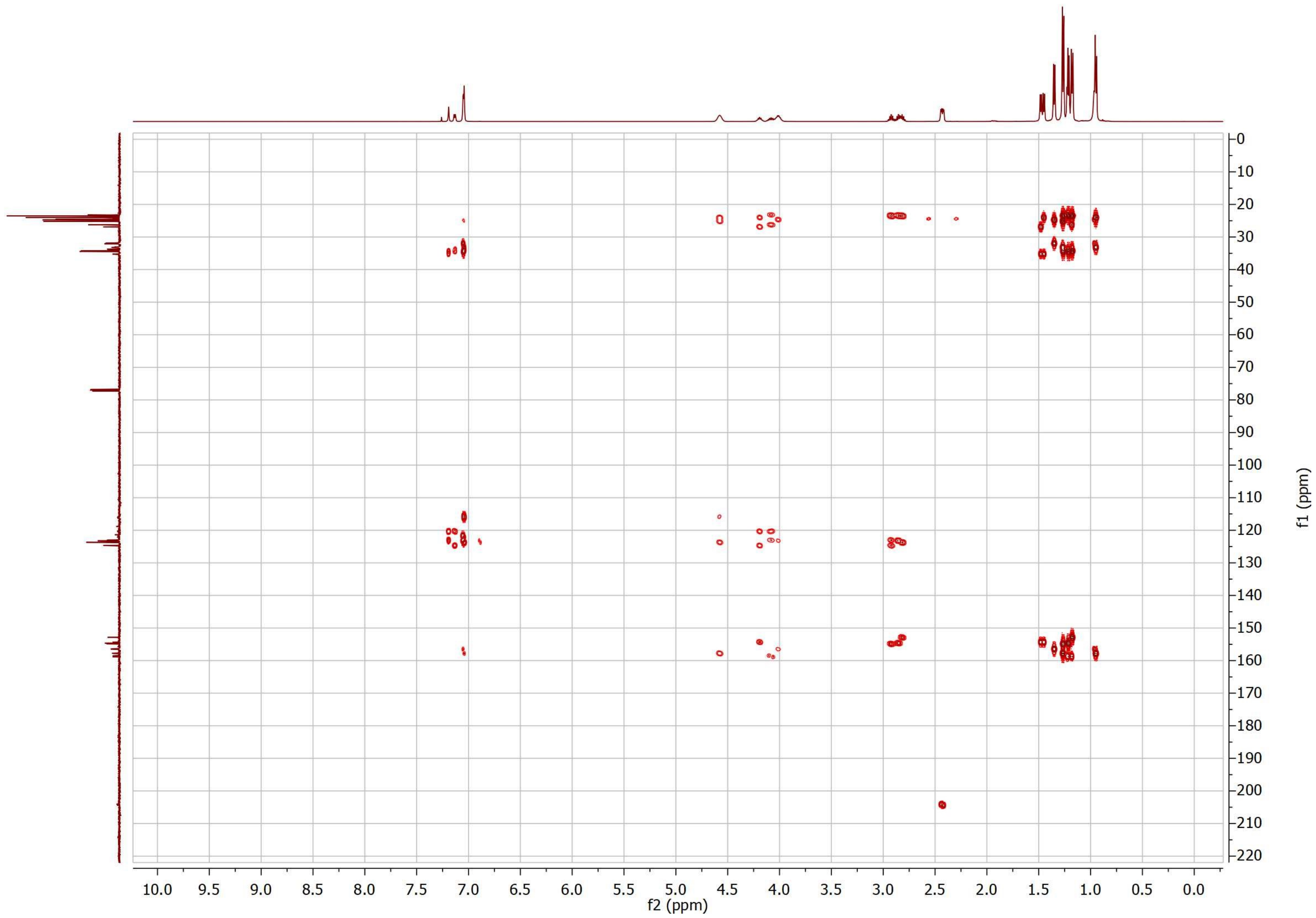

Figure S-7 HMBC NMR spectrum ($CDCl_3$) of compound $[1_{Tipp}]^+$ (R' = Me)

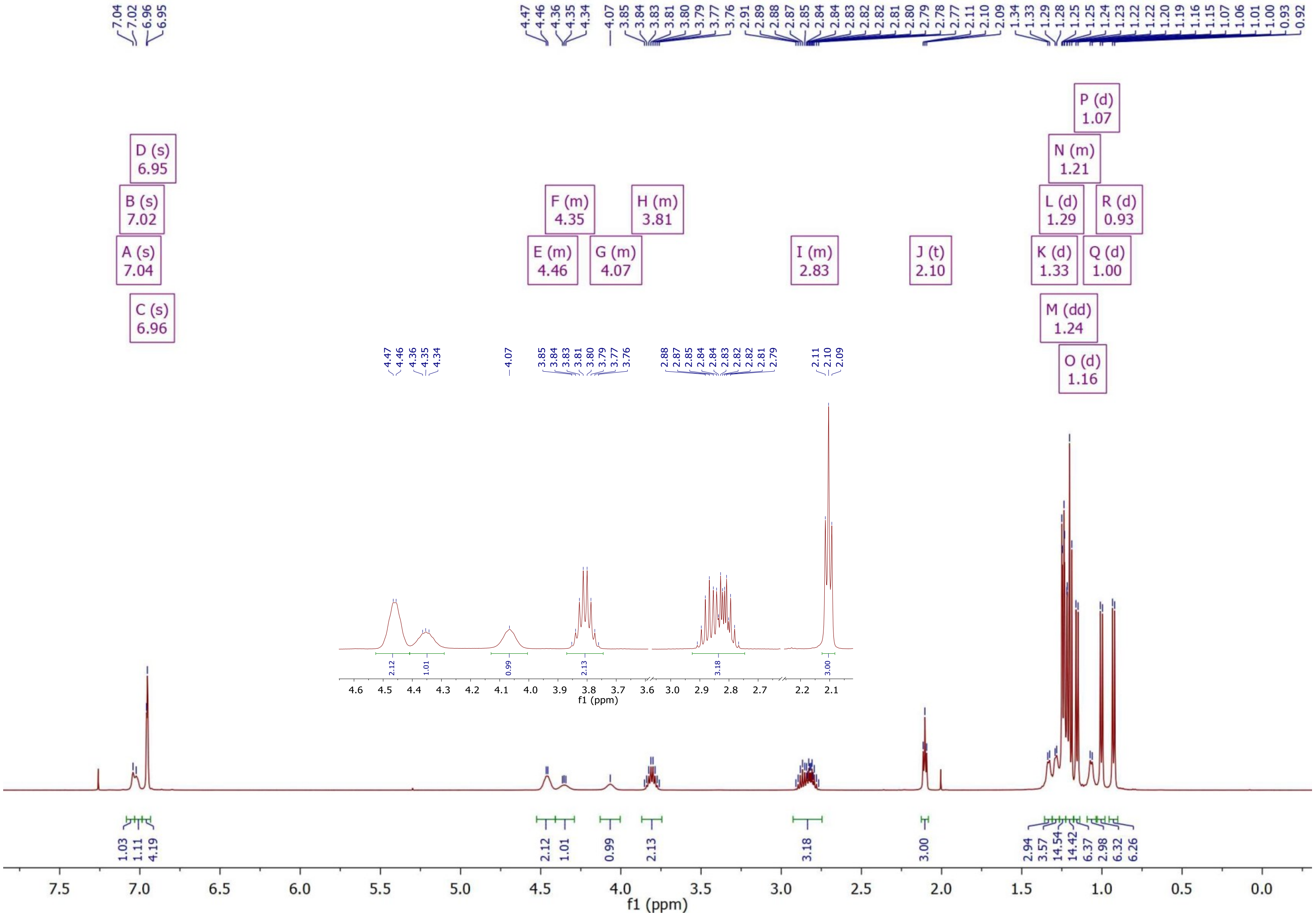

Figure S-8 ^{1}H NMR spectrum ($CDCl_3$) of compound 2_{Tipp} (R' = Me)

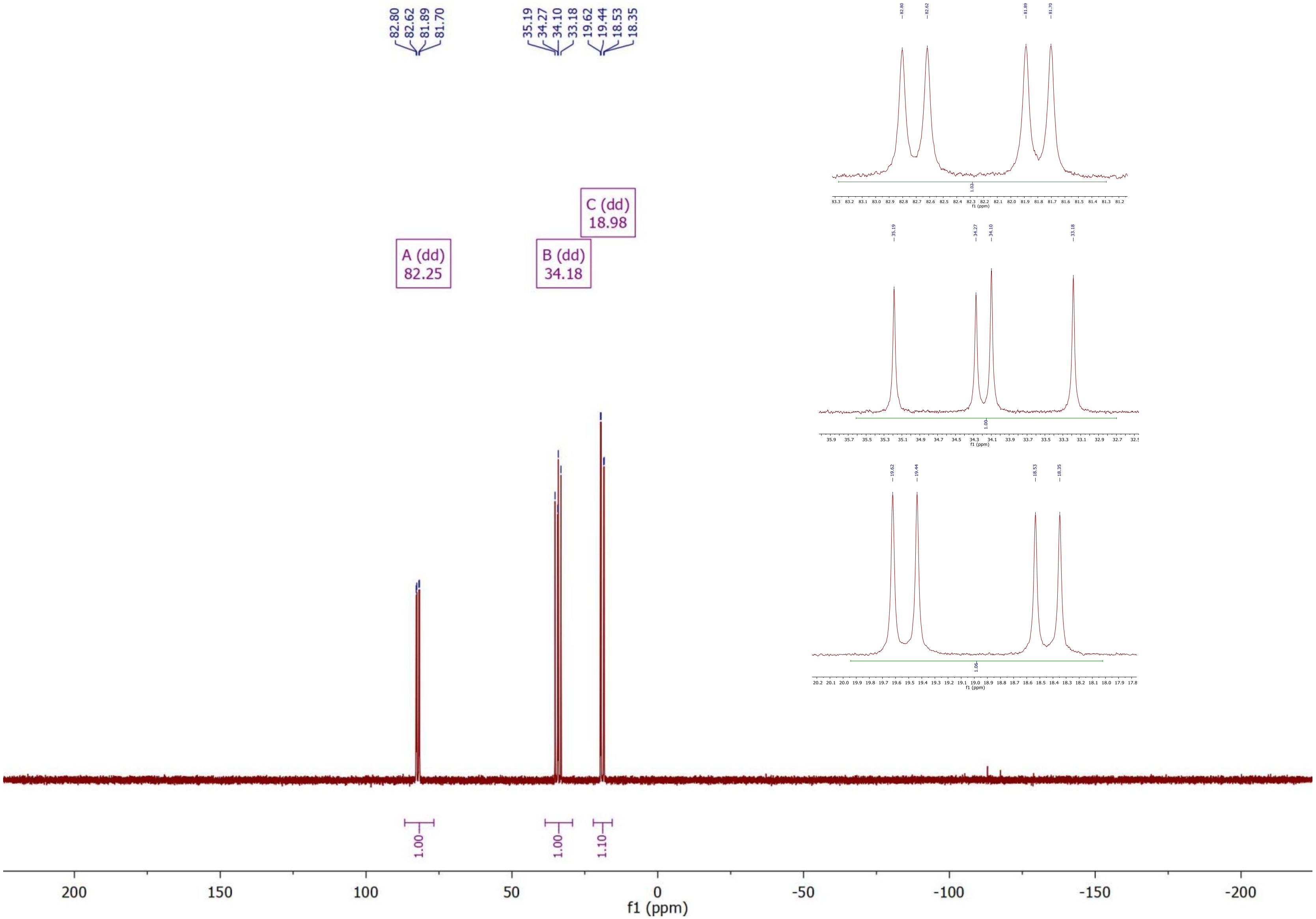

Figure S-9 $^{31}P\{^{1}H\}$ NMR spectrum ($CDCl_3$) of compound 2_{Tipp} (R' = Me)

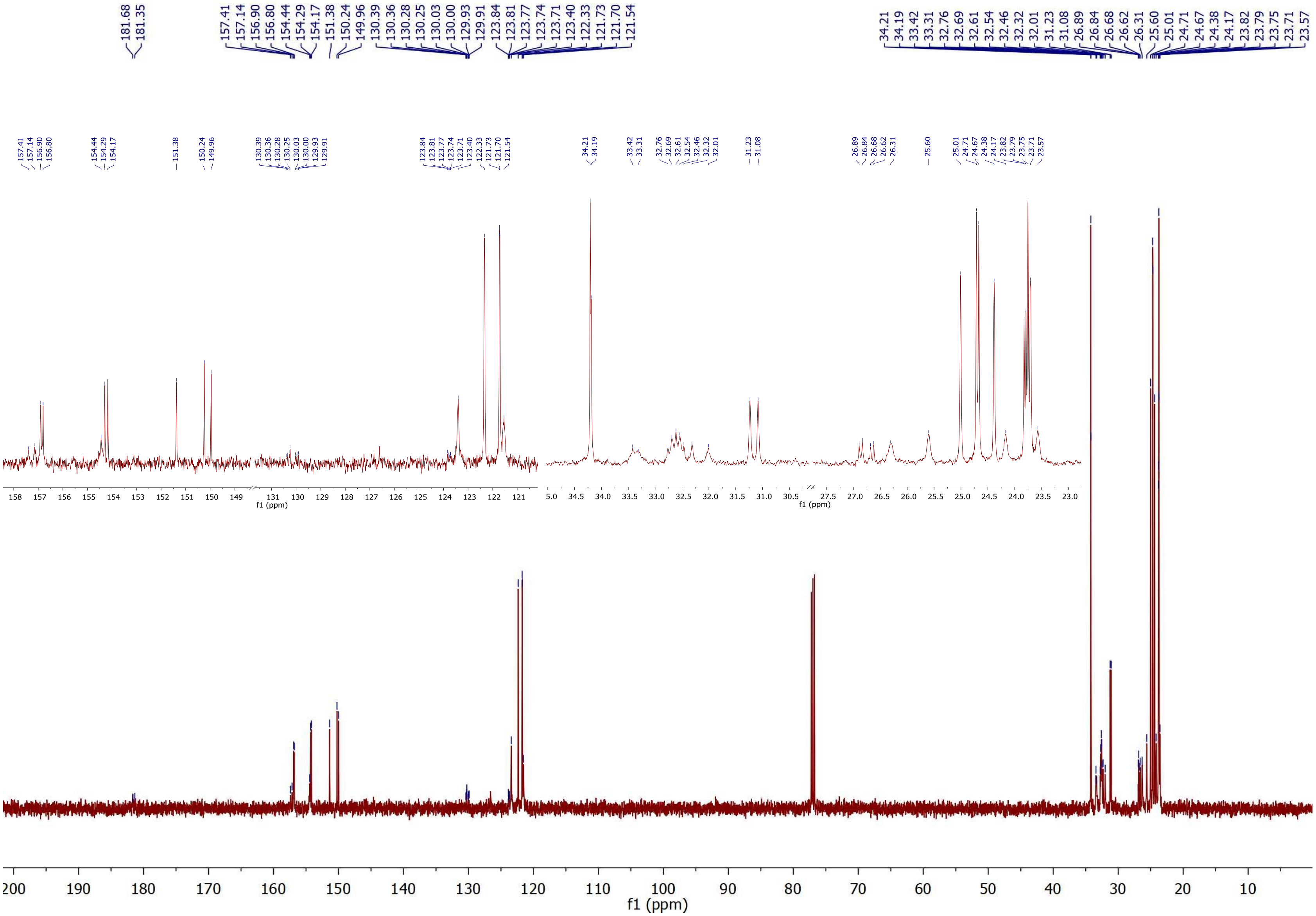

Figure S-10 $^{13}C\{^{1}H\}$ UDEFT NMR spectrum ($CDCl_3$) of compound 2_{Tipp} (R' = Me)

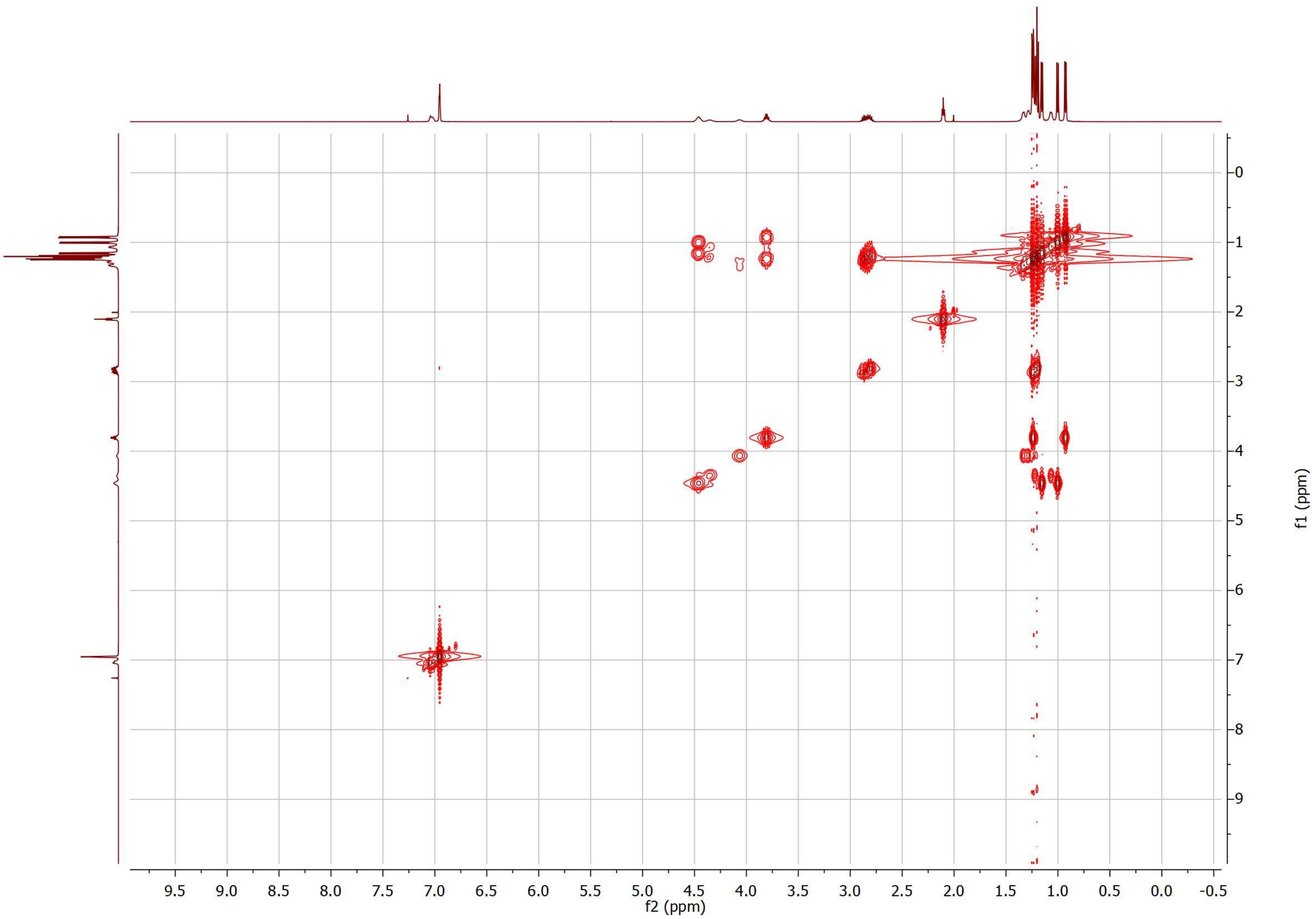

Figure S-11 ^{1}H COSY NMR spectrum ($CDCl_3$) of compound 2_{Tipp} (R’ = Me)

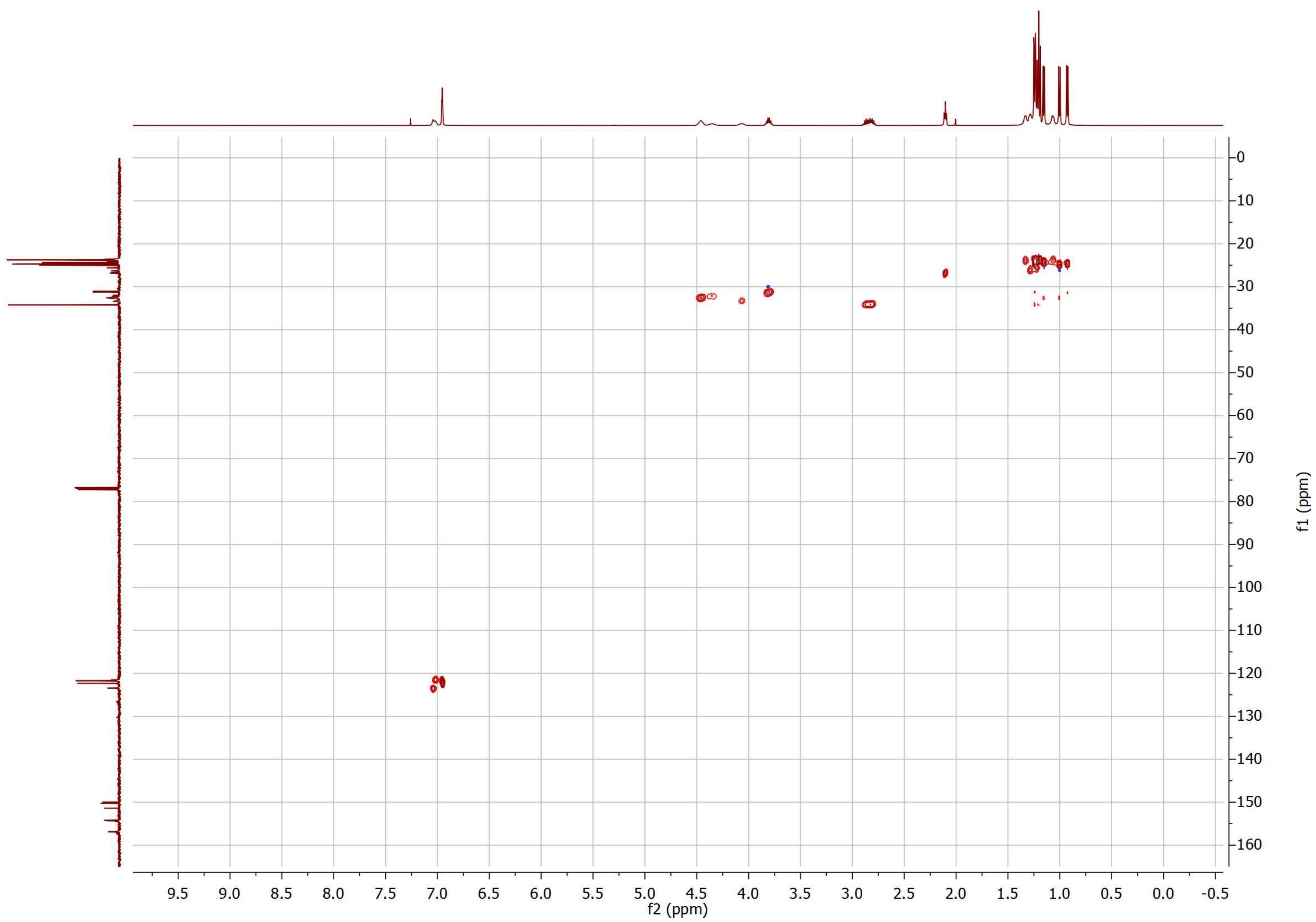

Figure S-12 HSQC NMR spectrum ($CDCl_3$) of compound 2_{Tipp} (R' = Me)

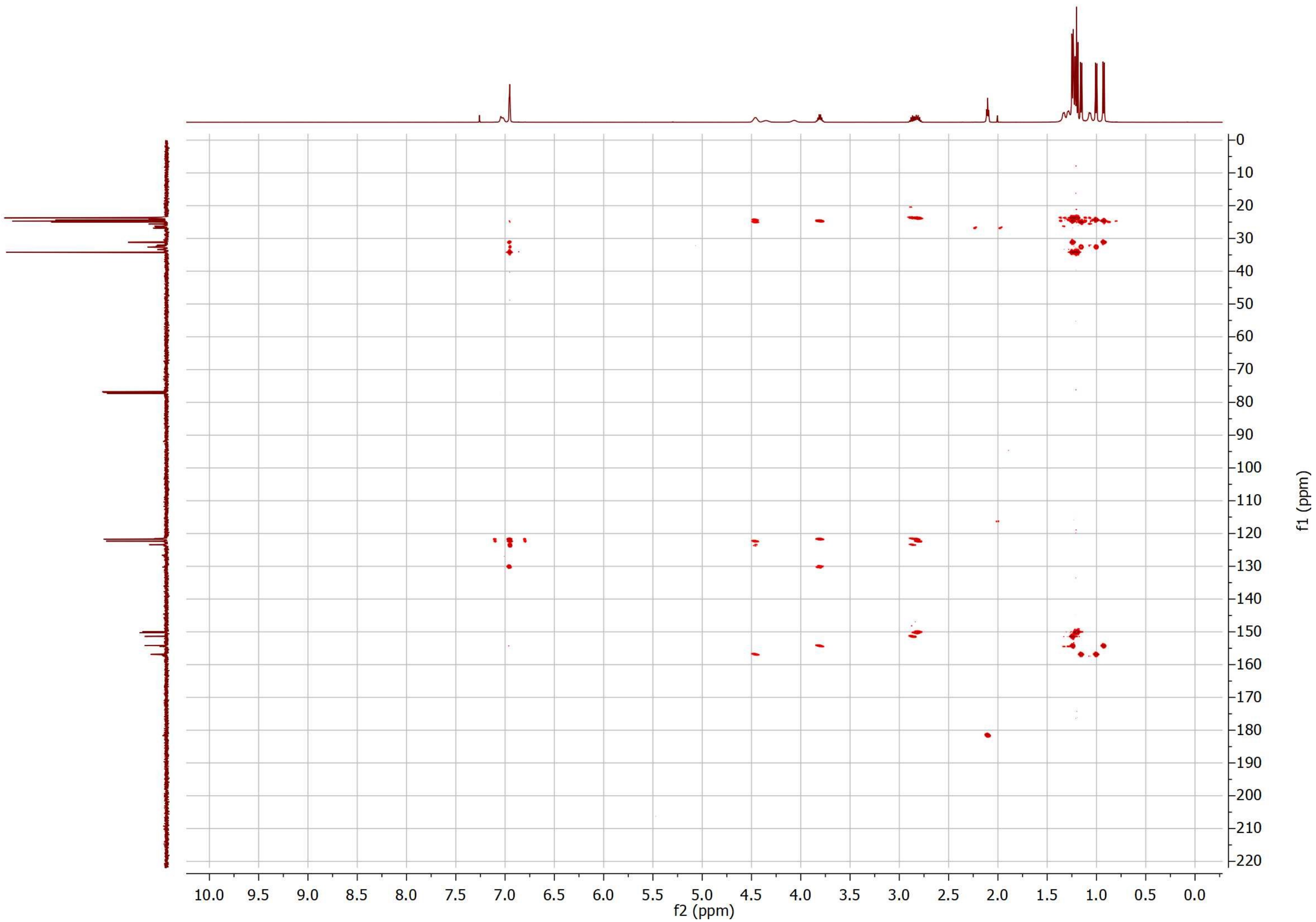

Figure S-13 HMBC NMR spectrum ($CDCl_3$) of compound 2_{Tipp} (R' = Me)

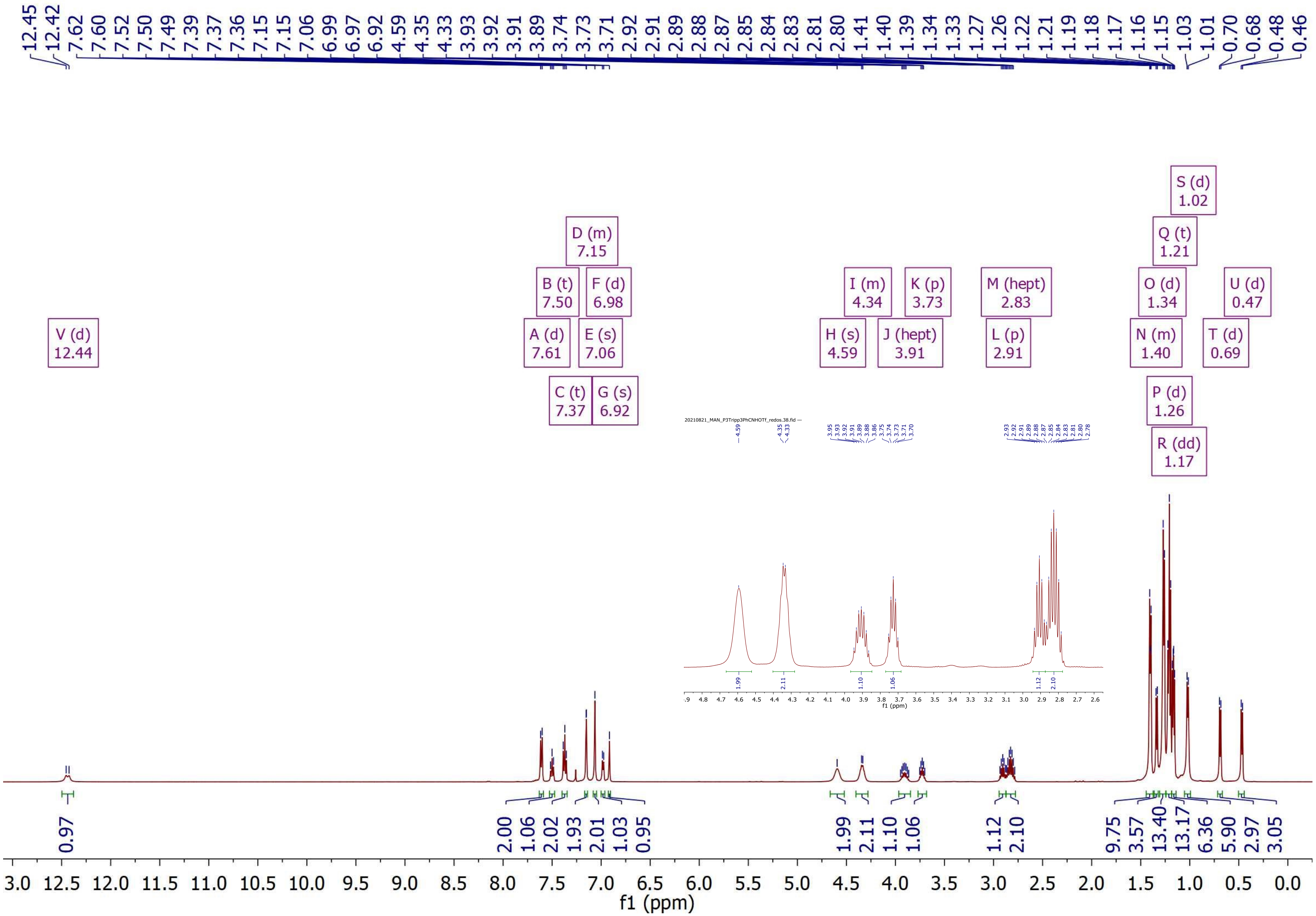

Figure S-14 ^{1}H NMR spectrum ($CDCl_3$) of compound $[1_{Tipp}]^+$ (R' = Ph)

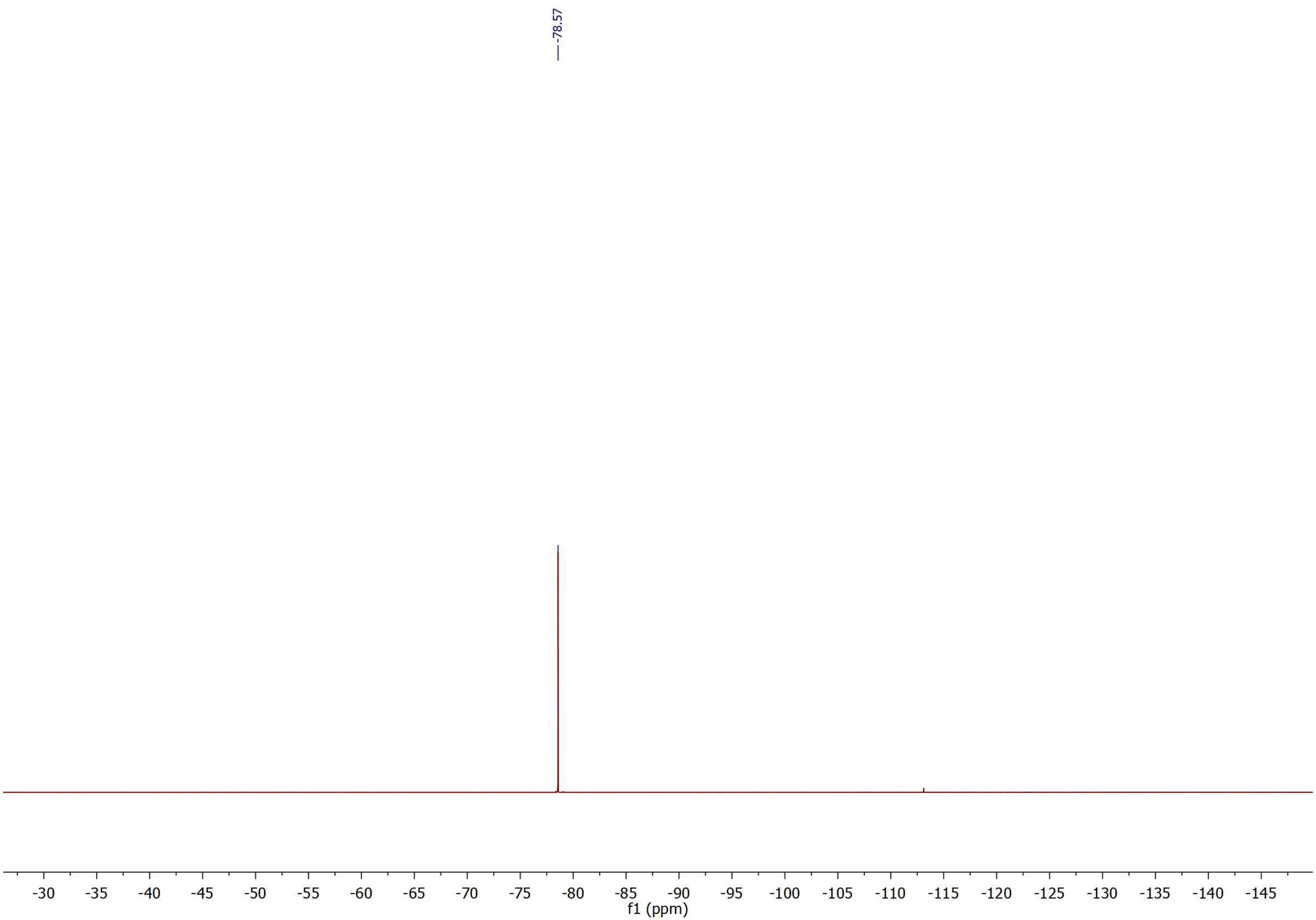

Figure S-15 ^{19}F NMR spectrum ($CDCl_3$) of compound $[1_{Tipp}]^+$ (R' = Ph)

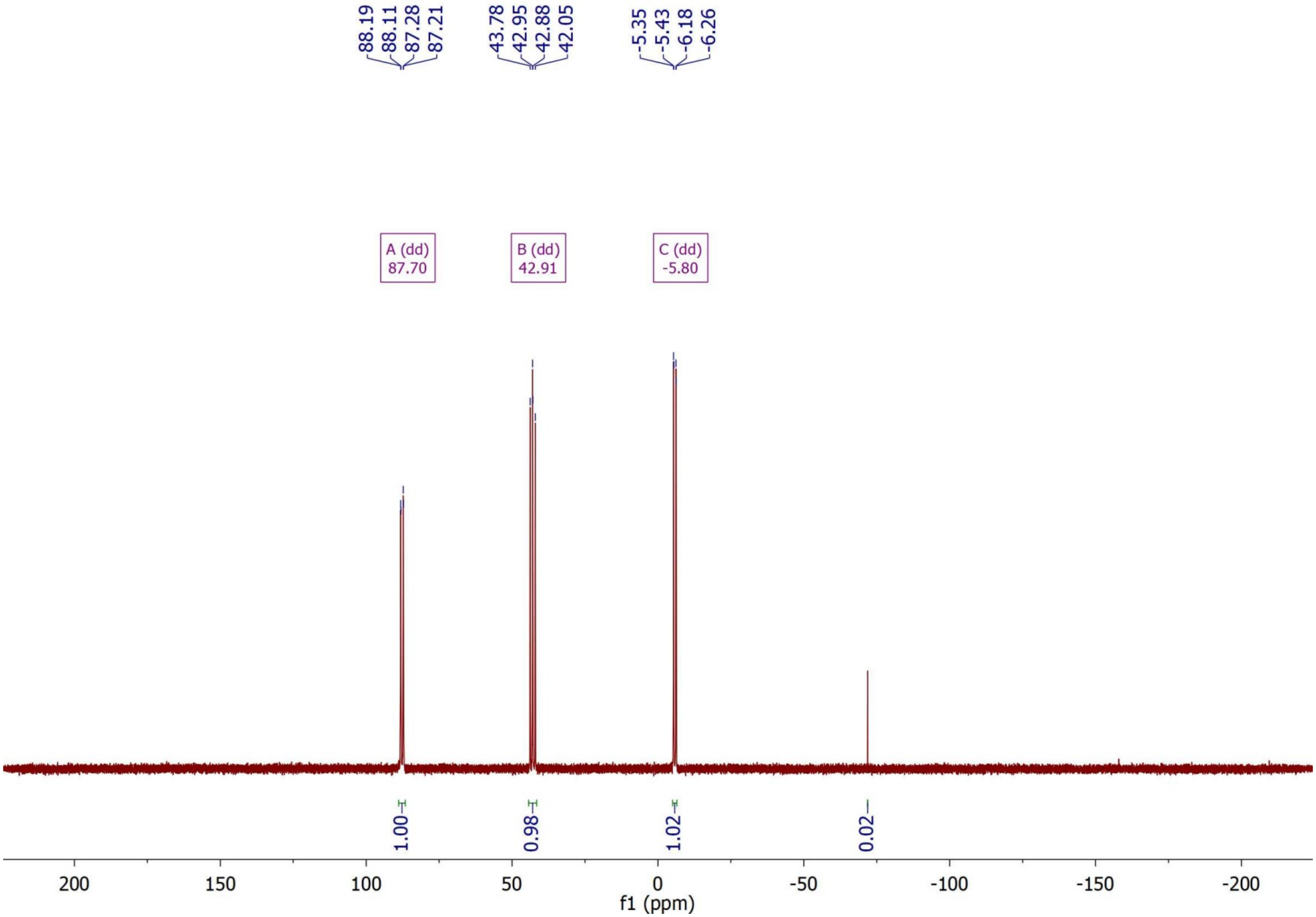

Figure S-16 $^{31}P\{^{1}H\}$ UDEFT NMR spectrum ($CDCl_3$) of compound $[1_{Tipp}]^{+}$ (R' = Ph)

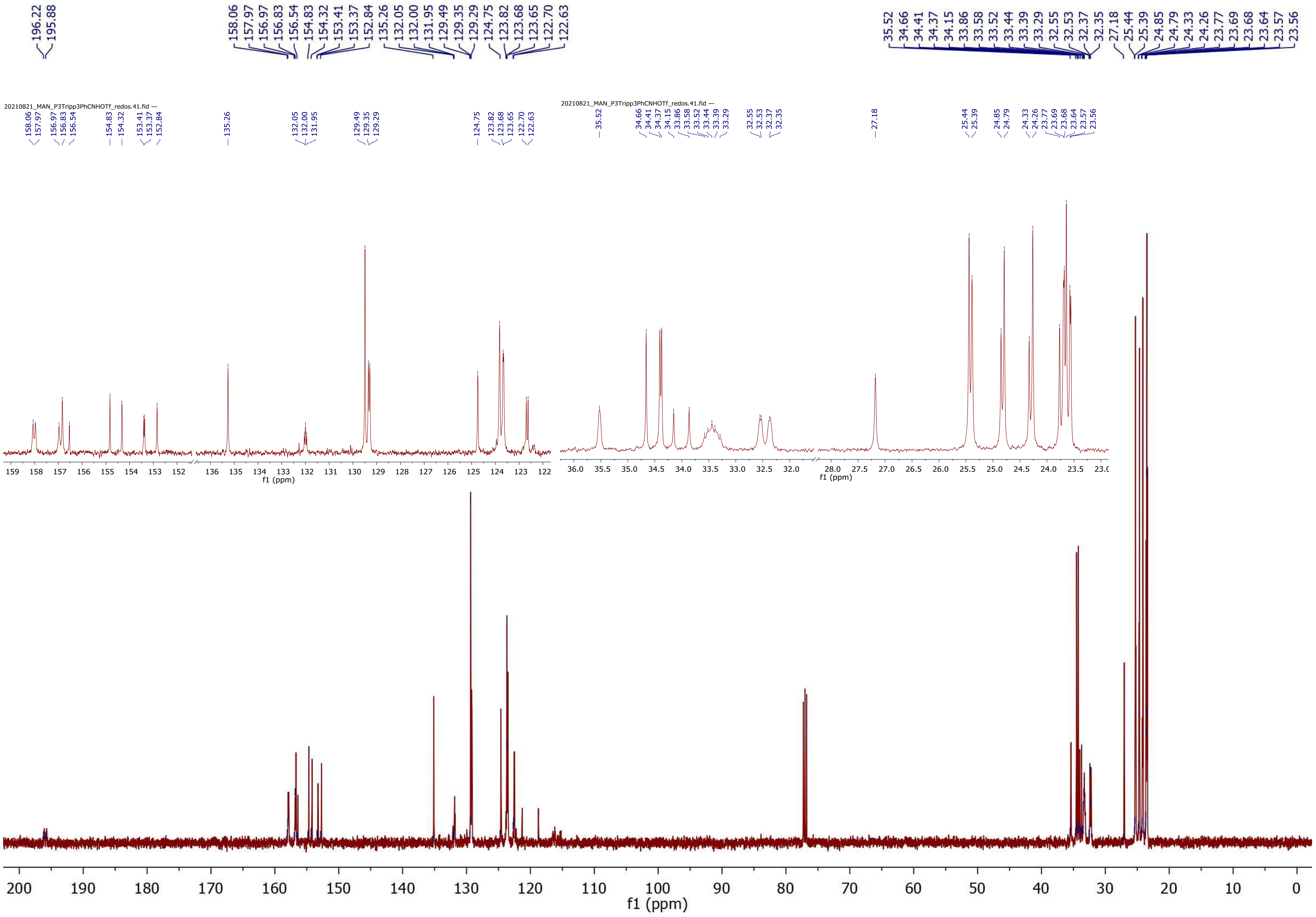

Figure S-17 $^{13}C\{^{1}H\}$ UDEFT NMR spectrum ($CDCl_3$) of compound $[1_{Tipp}]^+$ (R' = Ph)

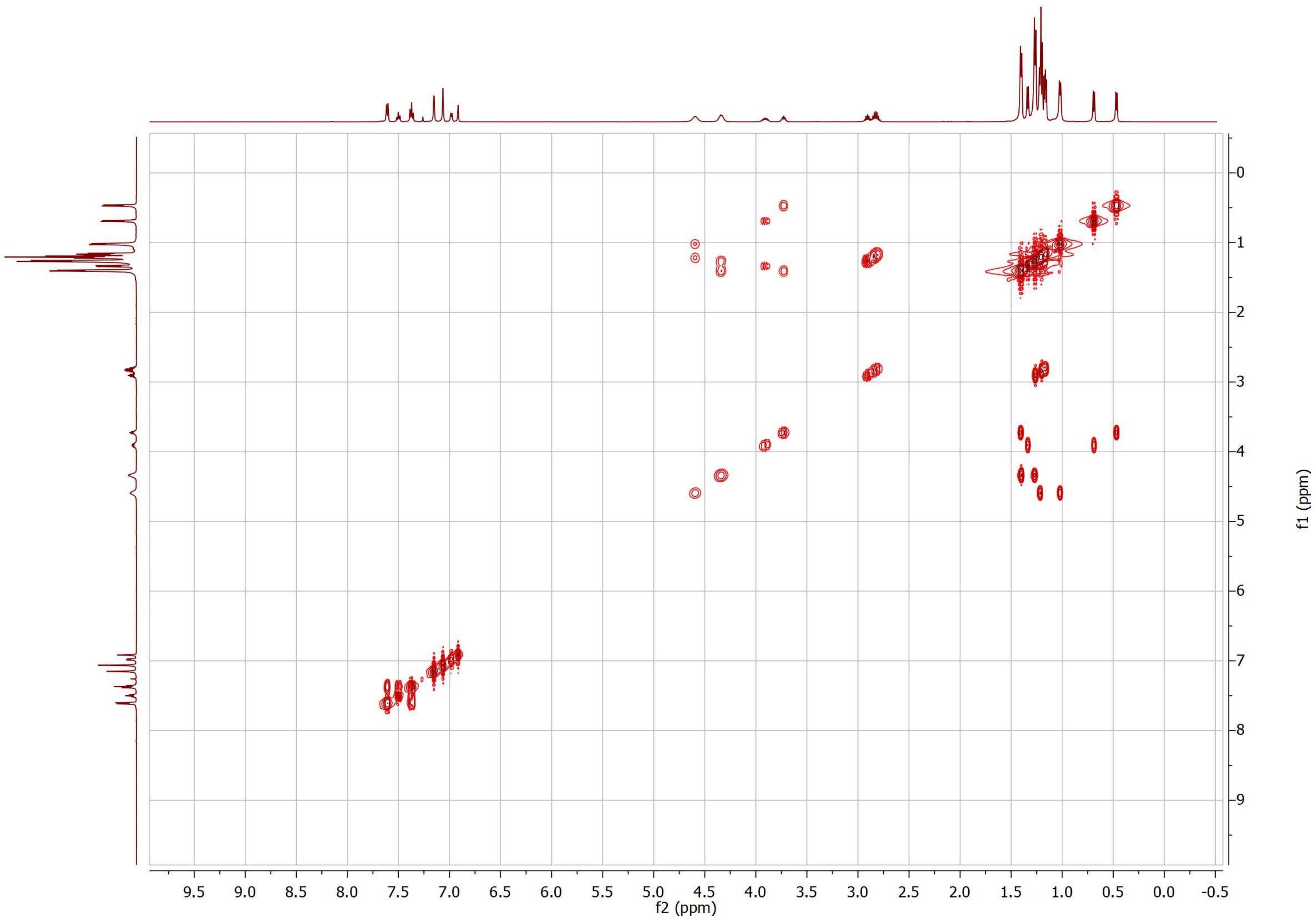

Figure S-18 ^{1}H COSY NMR spectrum ($CDCl_3$) of compound $[1_{Tipp}]^+$ (R' = Ph)

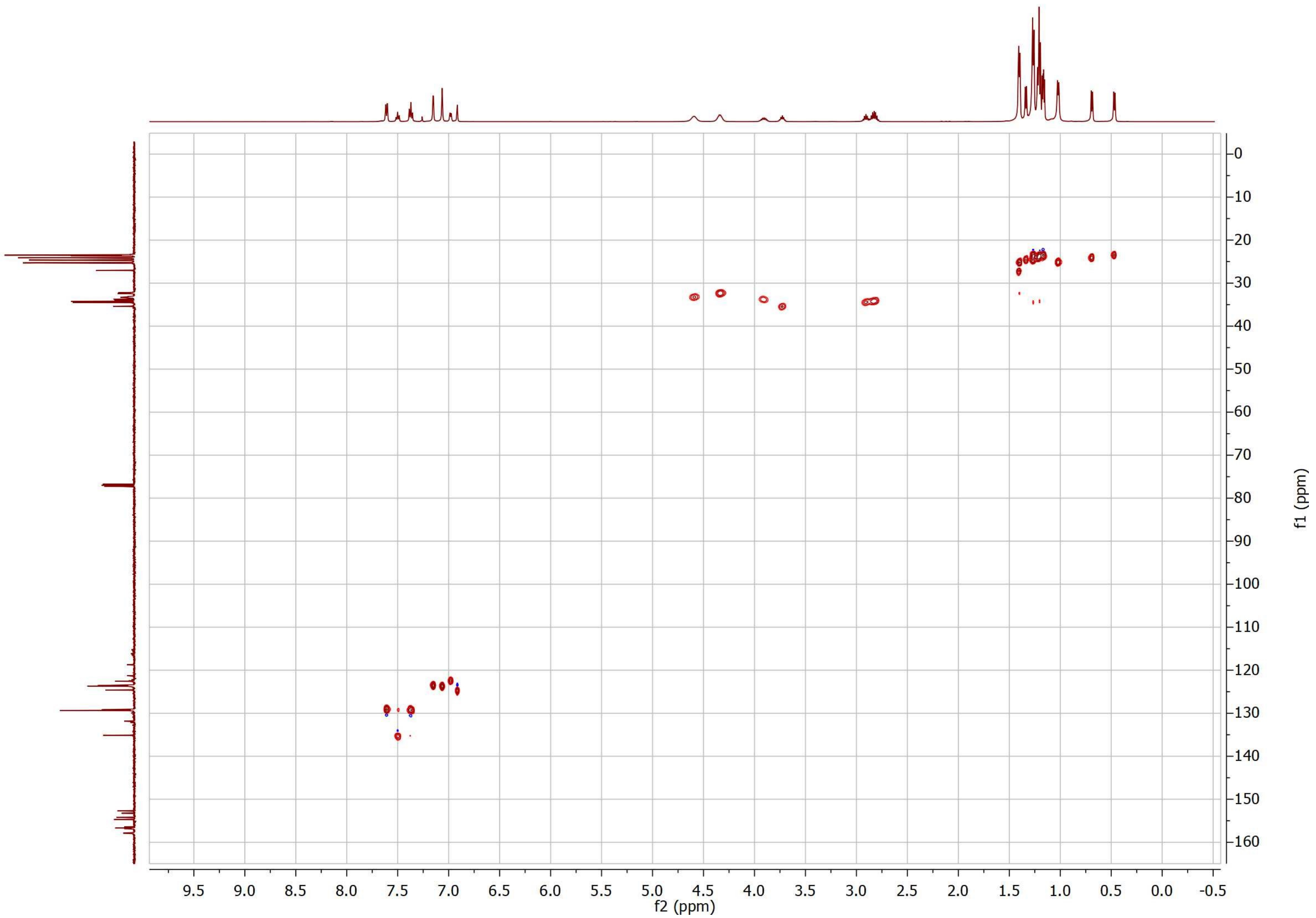

Figure S-19 HSQC NMR spectrum ($CDCl_3$) of compound $[1_{Tipp}]^+$ (R' = Ph)

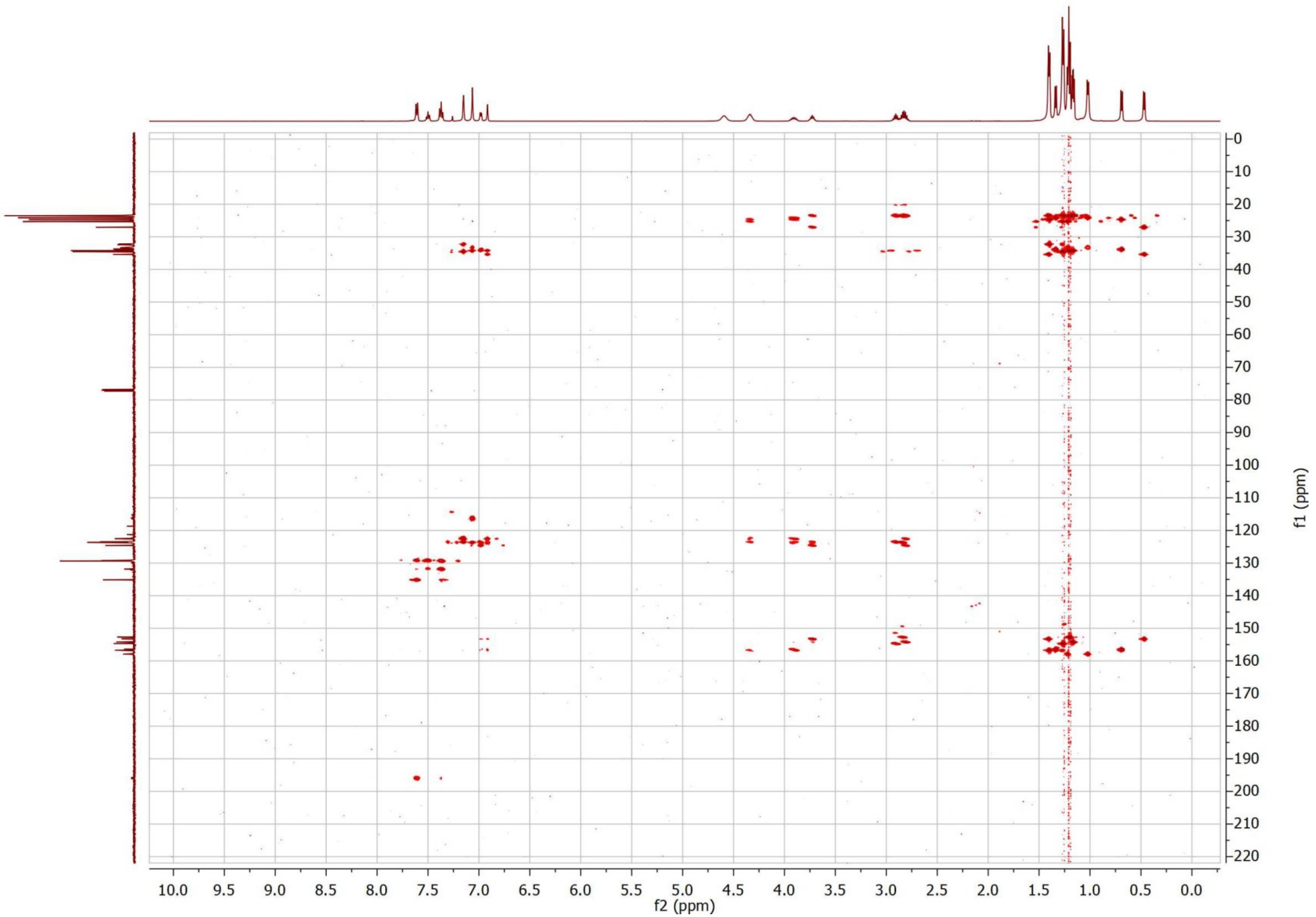

Figure S-20 HMBC NMR spectrum (CDCl$_3$) of compound [1$_{Tipp}$]$^+$ (R' = Ph)

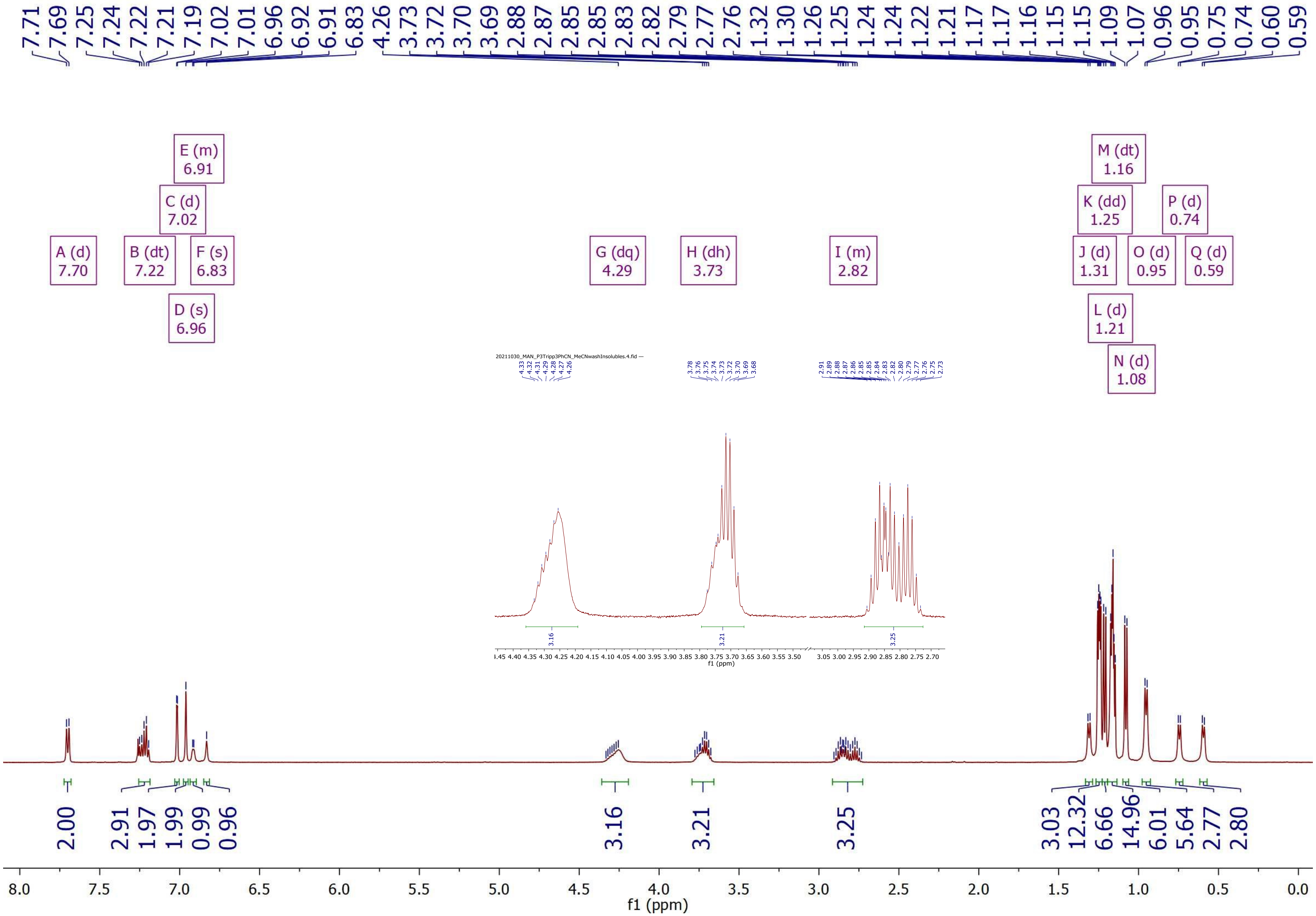

Figure S-21 ^{1}H NMR spectrum ($CDCl_3$) of compound 2_{Tipp} (R' = Ph)

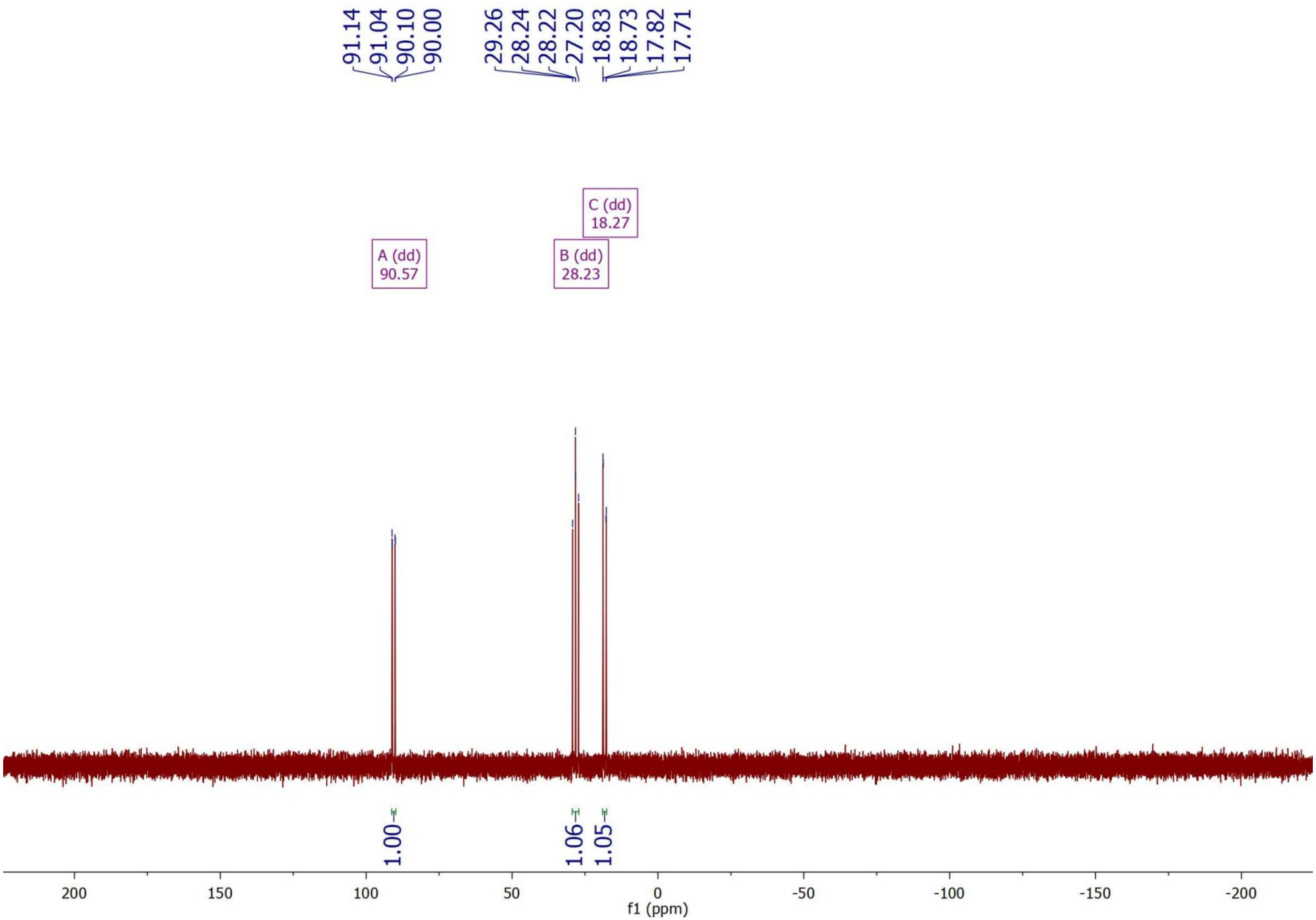

Figure S-22 $^{31}P\{^{1}H\}$ NMR spectrum ($CDCl_3$) of compound 2_{Tipp} (R' = Ph)

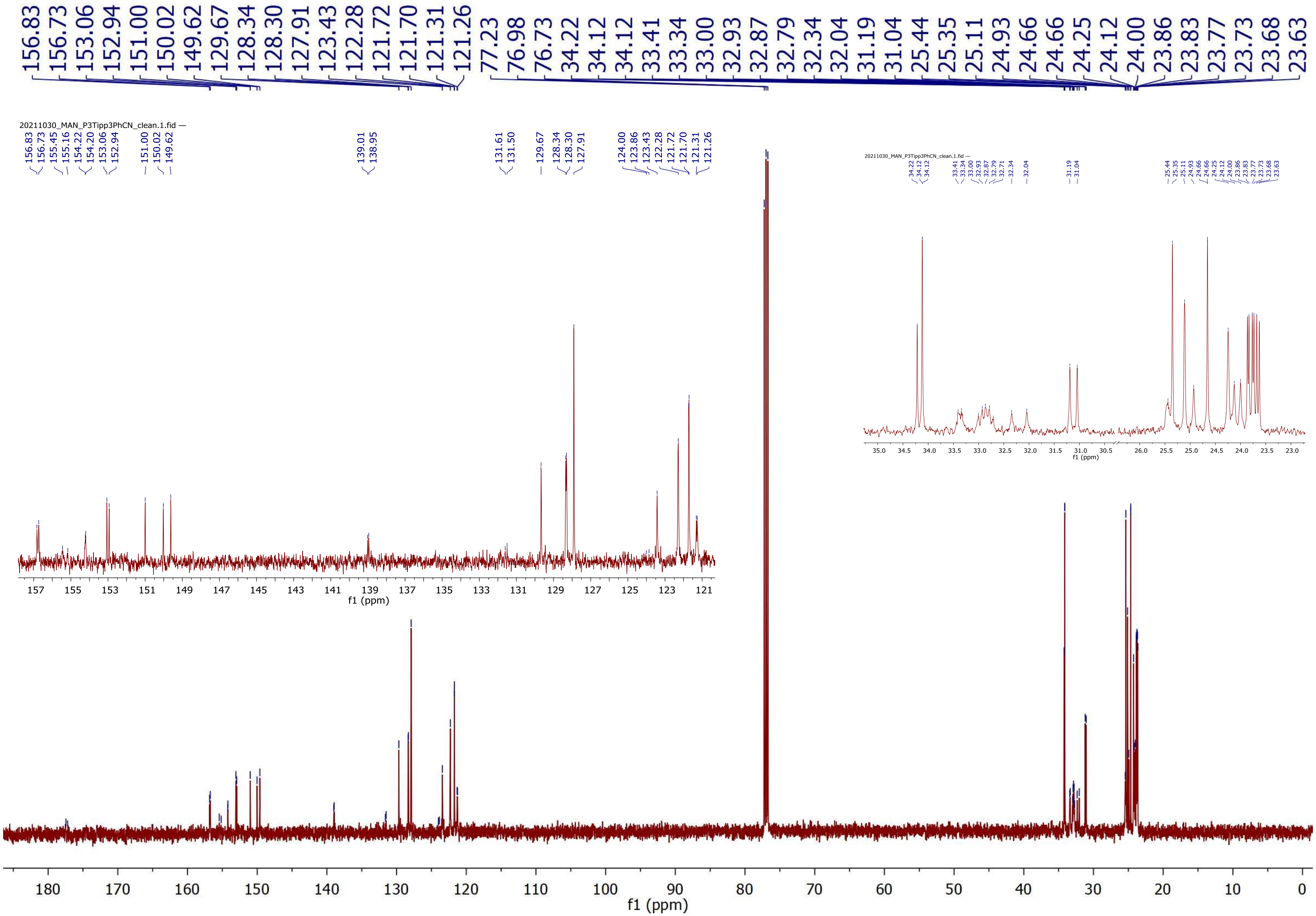

Figure S-23 $^{13}C\{^1H\}$ UDEFT NMR spectrum ($CDCl_3$) of compound 2_{Tipp} (R' = Ph)

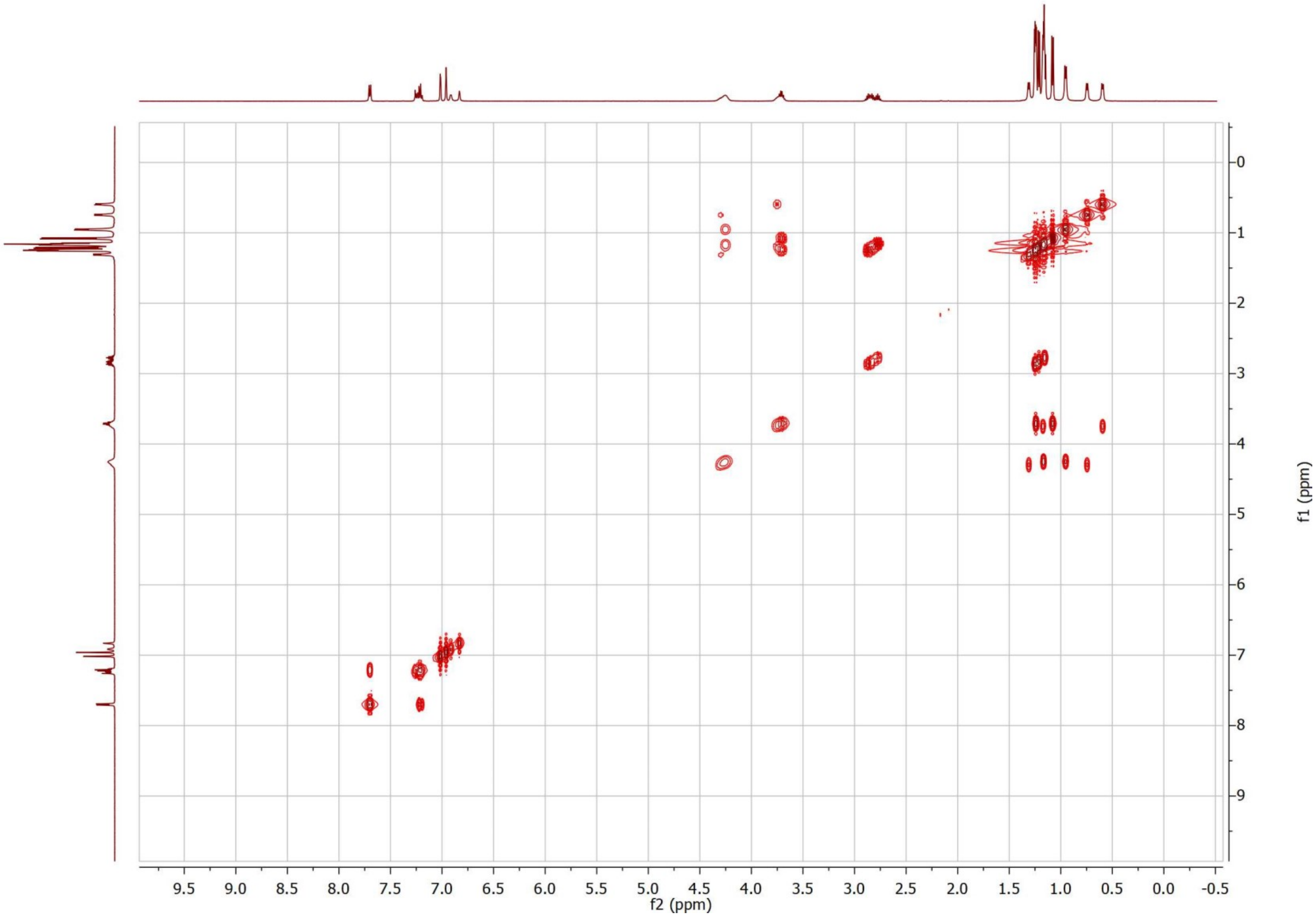

Figure S-24 ^{1}H COSY NMR spectrum ($CDCl_3$) of compound 2_{Tipp} (R' = Ph)

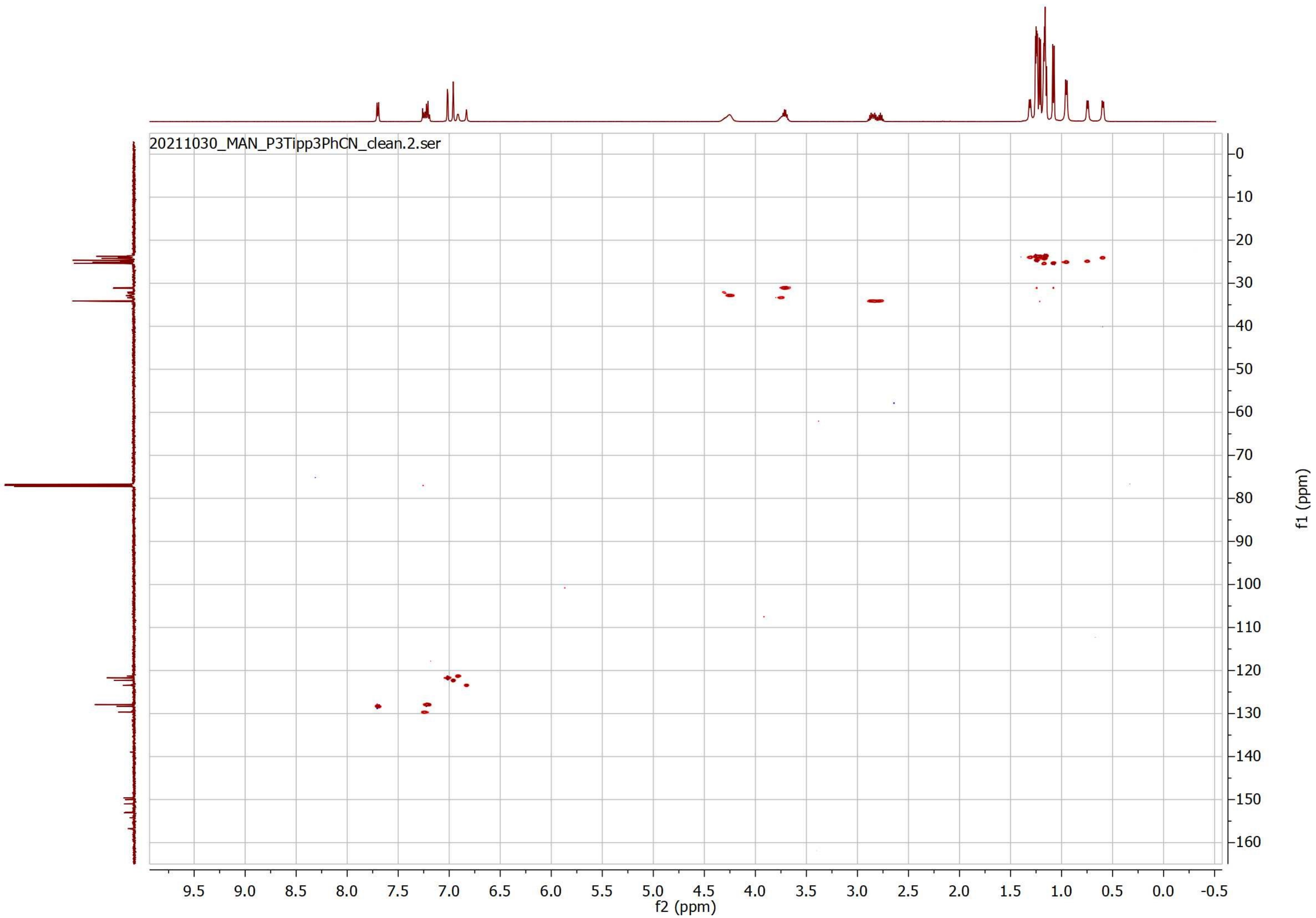

Figure S-25 HSQC NMR spectrum ($CDCl_3$) of compound 2_{Tipp} (R' = Ph)

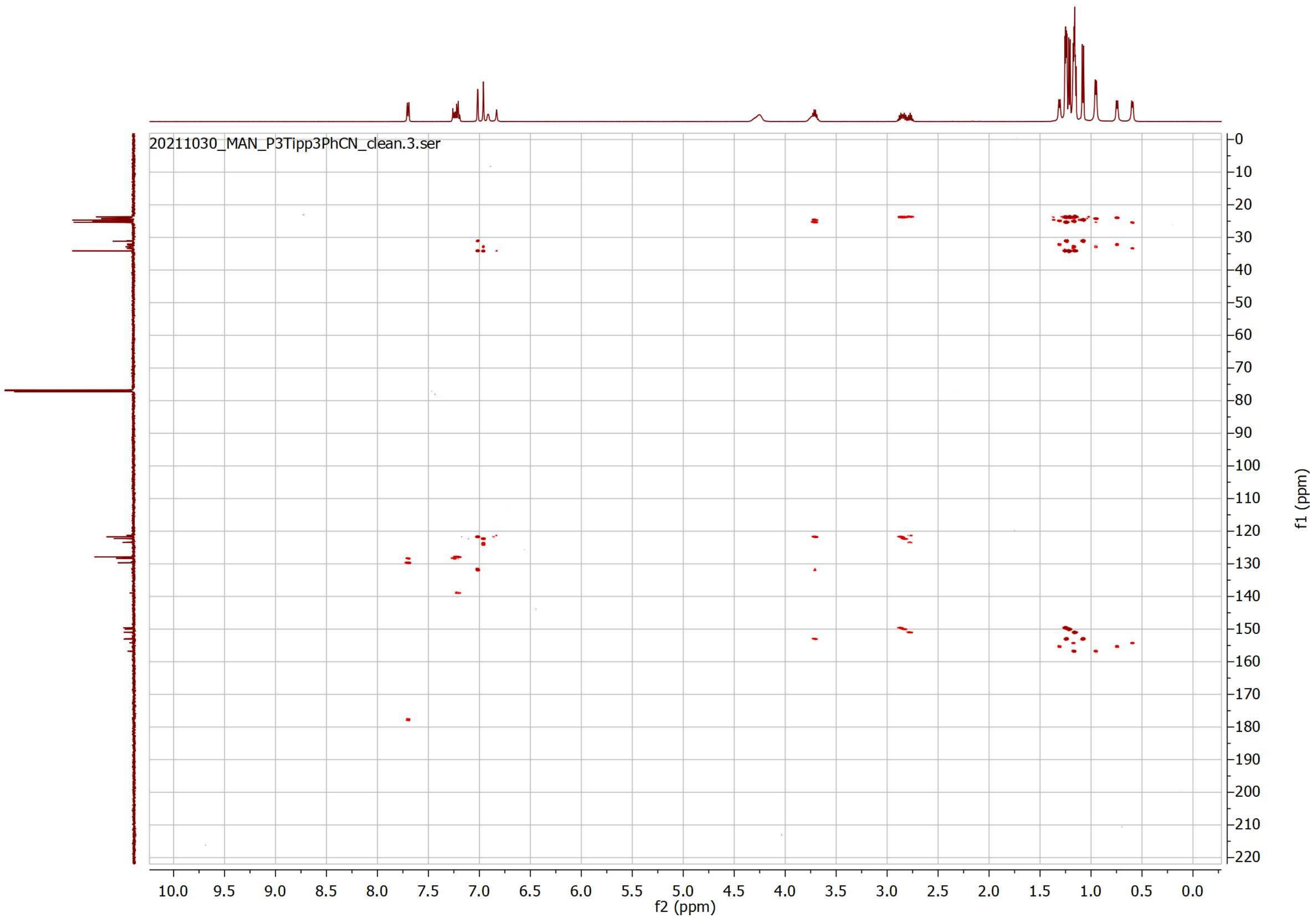

Figure S-26 HMBC NMR spectrum ($CDCl_3$) of compound 2_{Tipp} (R' = Ph)

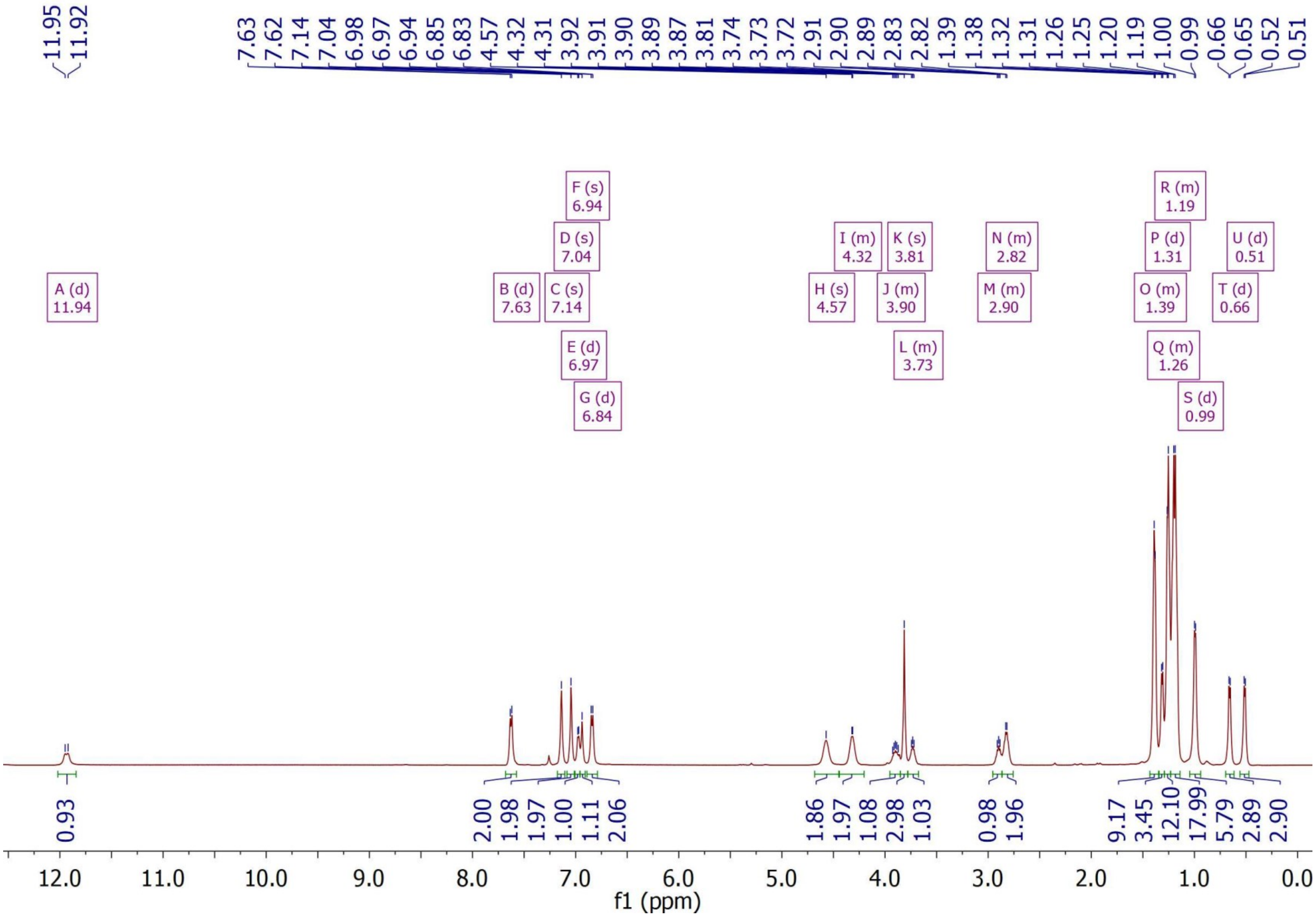

Figure S-27 ^{1}H NMR spectrum ($CDCl_3$) of compound $[1_{Tipp}]^+$ (R' = *p*-$MeOC_6H_4$)

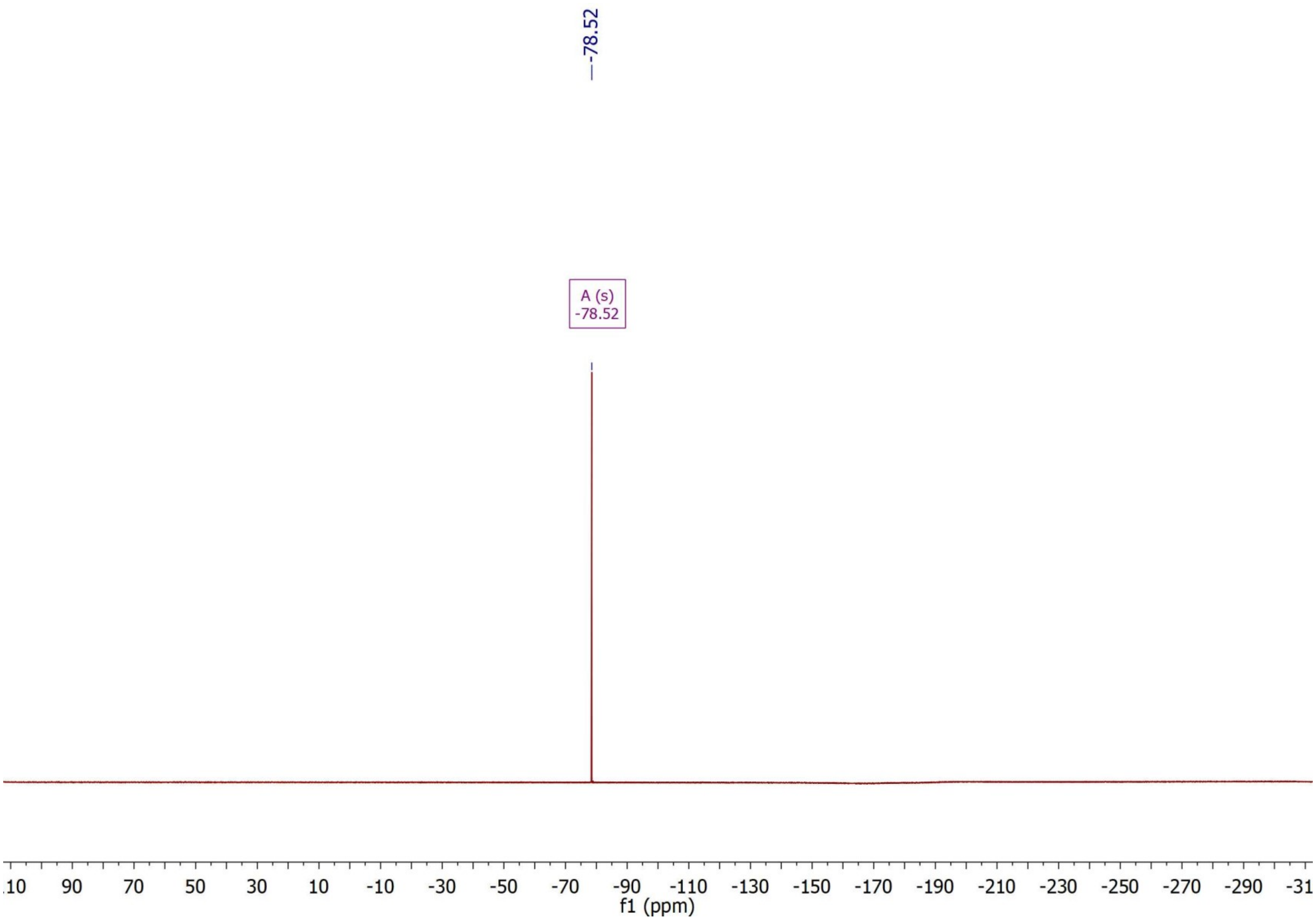

Figure S-28 ^{19}F NMR spectrum ($CDCl_3$) of compound $[1_{Tipp}]^+$ (R' = *p*-MeOC_6H_4)

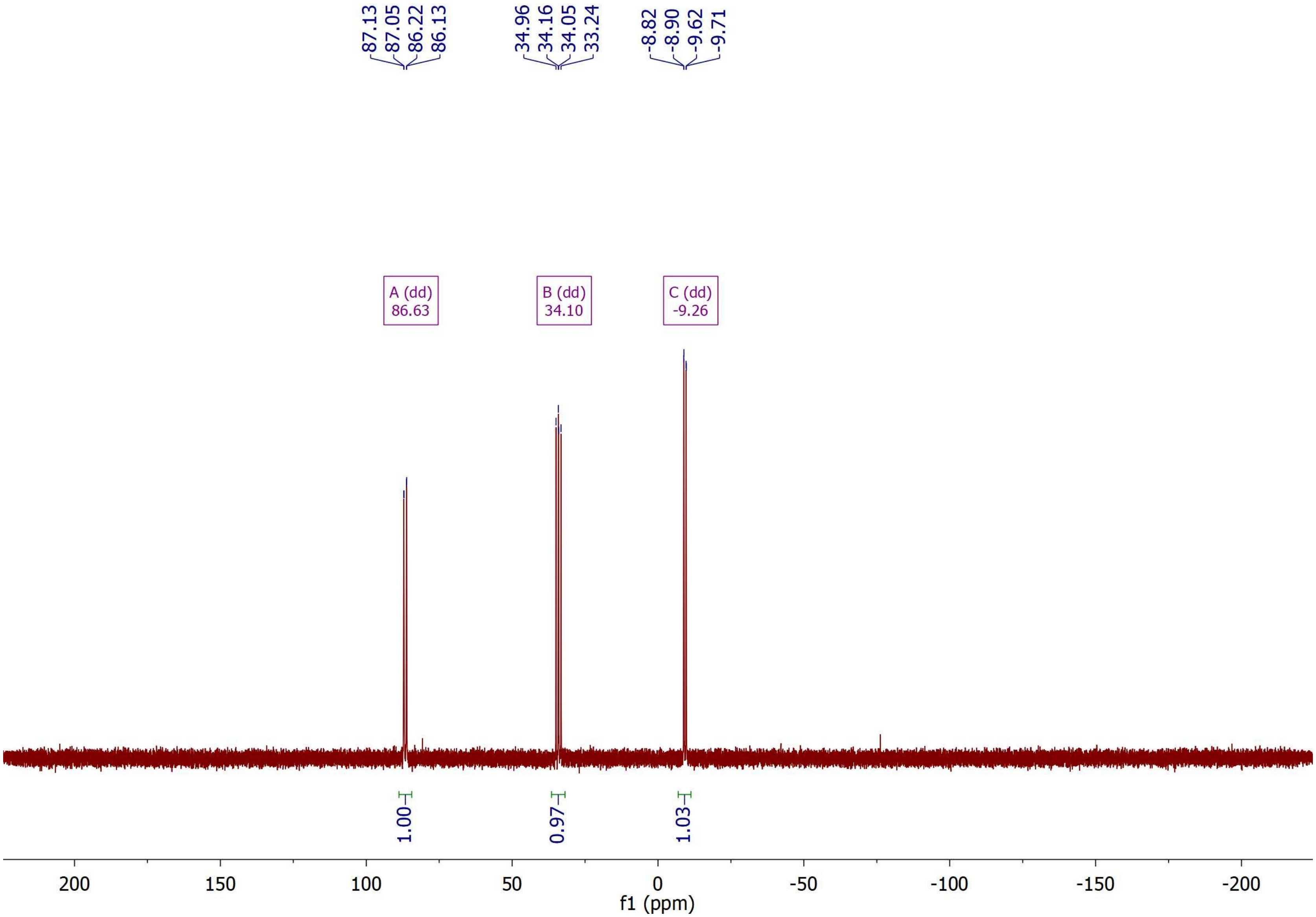

Figure S-29 $^{31}P\{^{1}H\}$ NMR spectrum ($CDCl_3$) of compound $[1_{Tipp}]^+$ (R' = *p*-$MeOC_6H_4$)

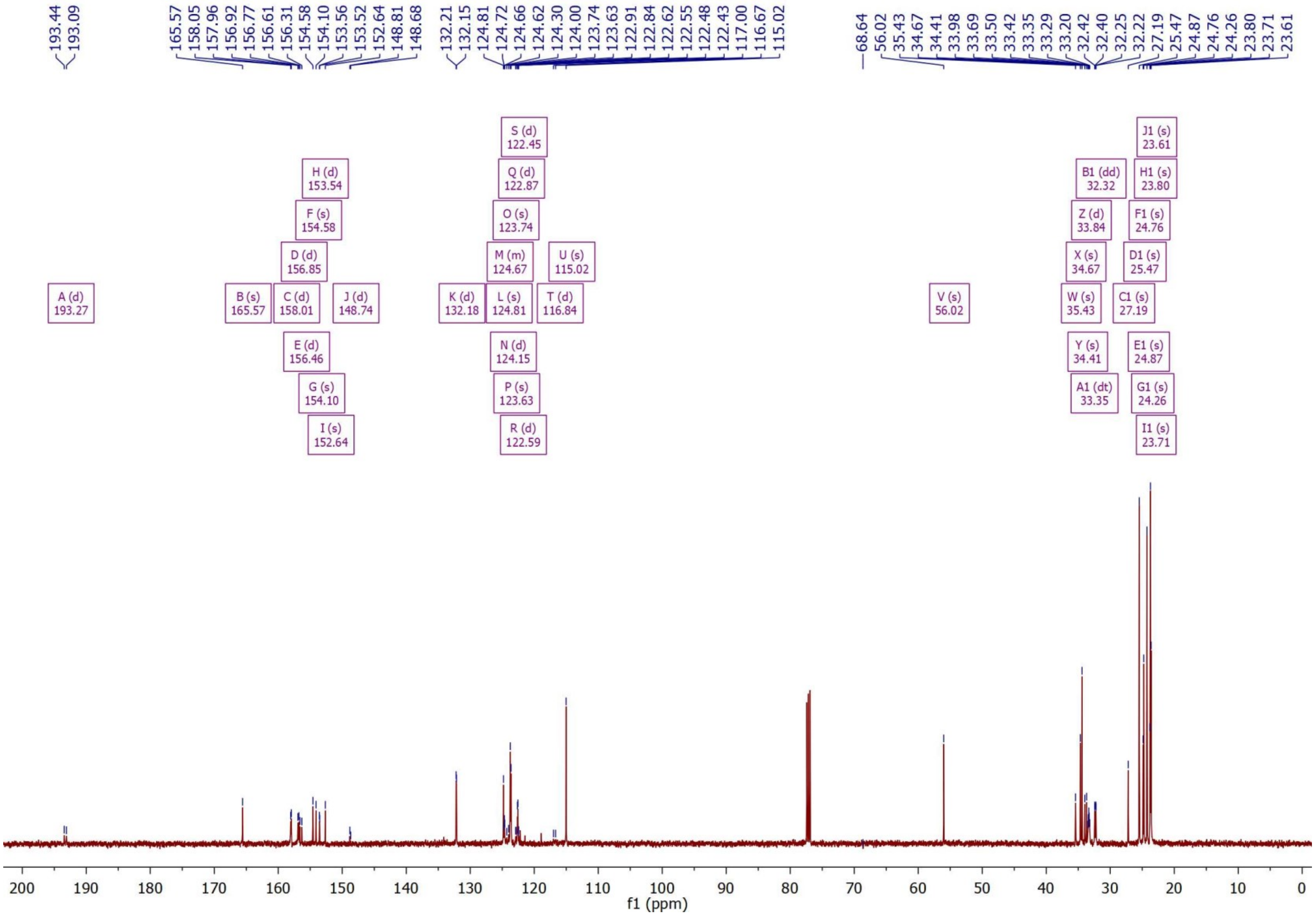

Figure S-30 $^{13}C\{^{1}H\}$ UDEFT NMR spectrum ($CDCl_3$) of compound $[1_{Tipp}]^+$ (R' = *p*-$MeOC_6H_4$)

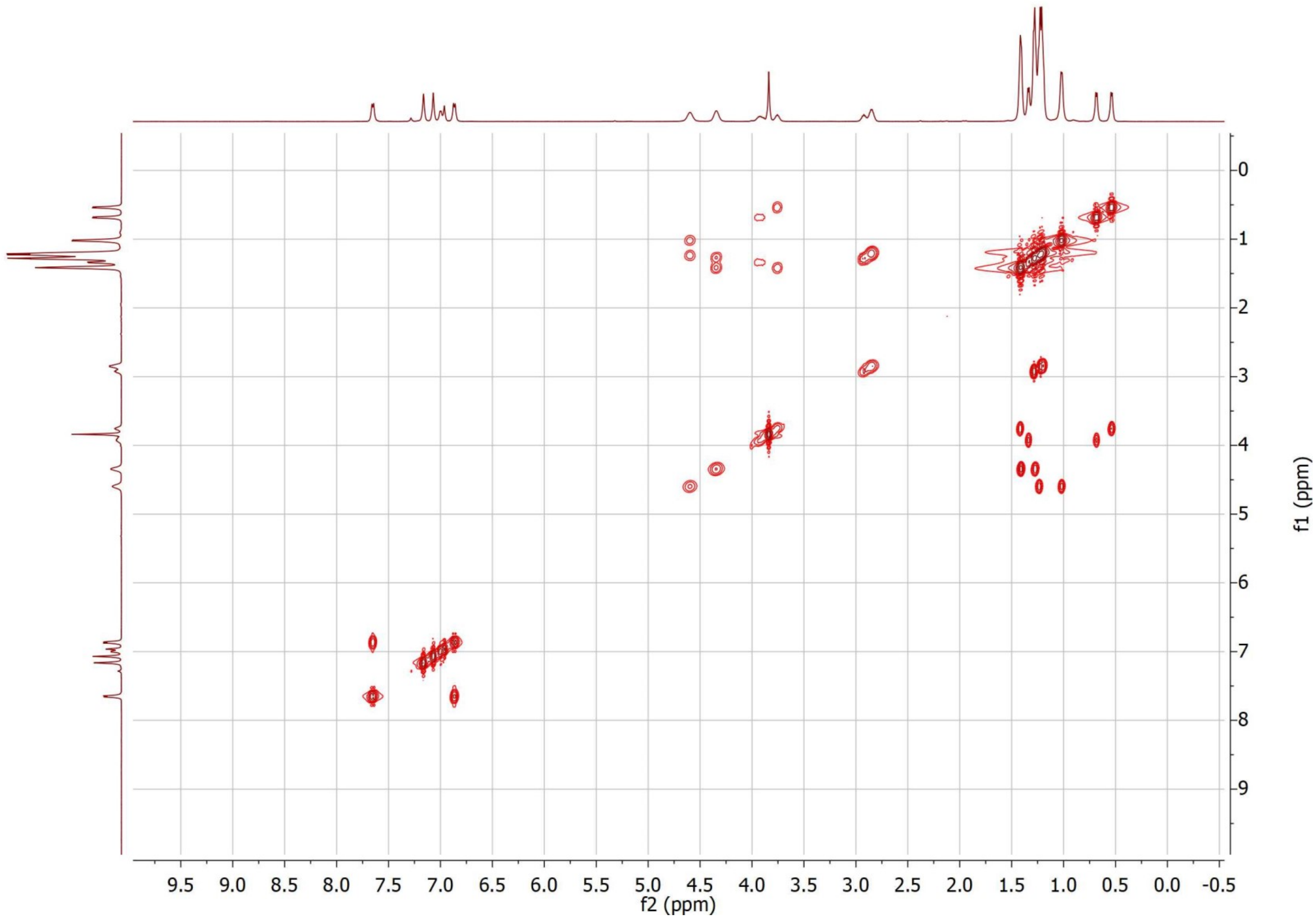

Figure S-31 ^{1}H COSY NMR spectrum ($CDCl_3$) of compound $[1_{Tipp}]^+$ (R' = *p*-$MeOC_6H_4$)

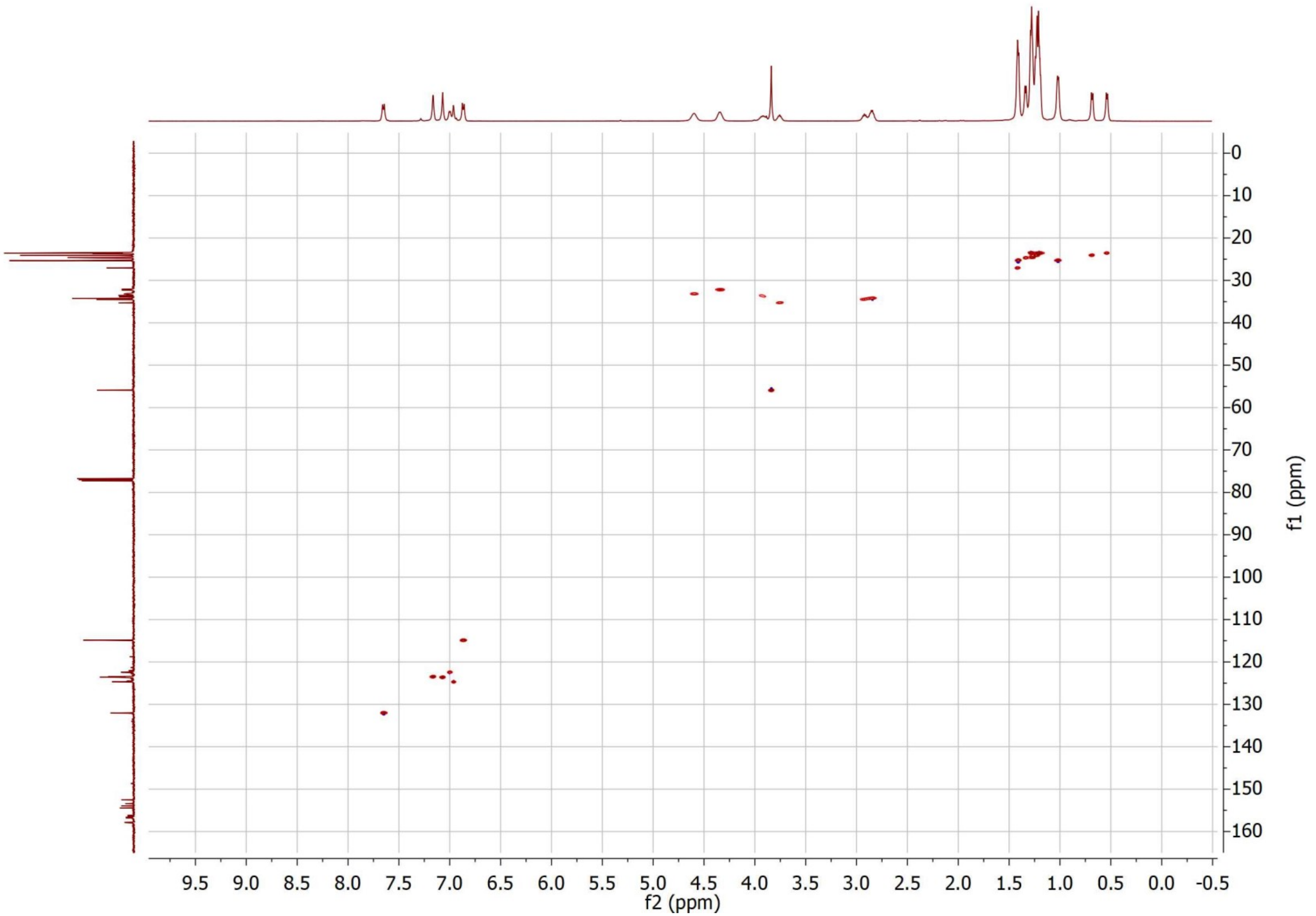

Figure S-32 HSQC NMR spectrum ($CDCl_3$) of compound $[1_{Tipp}]^+$ (R' = *p*-$MeOC_6H_4$)

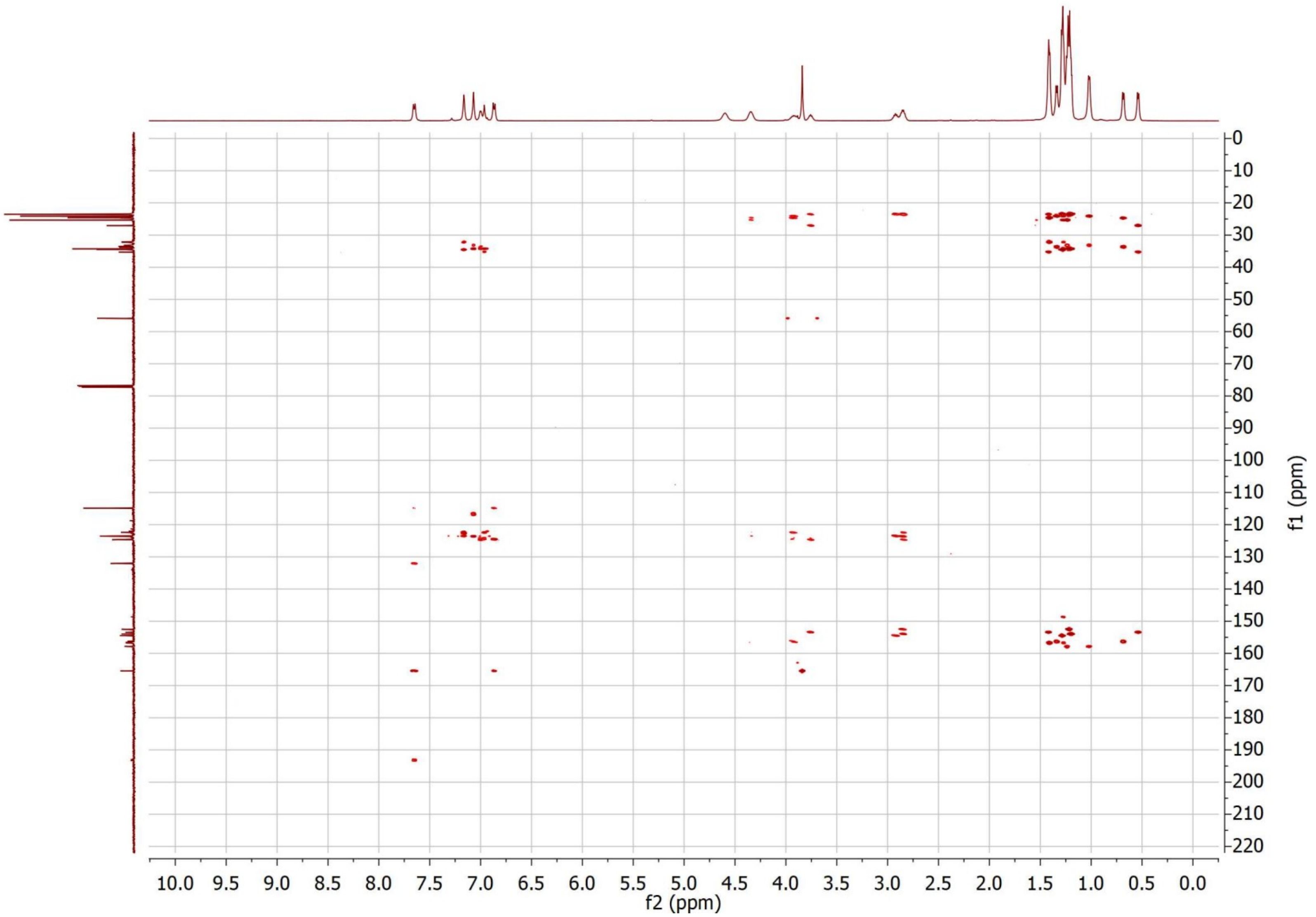

Figure S-33 HMBC NMR spectrum ($CDCl_3$) of compound $[1_{Tipp}]^+$ (R' = *p*-$MeOC_6H_4$)

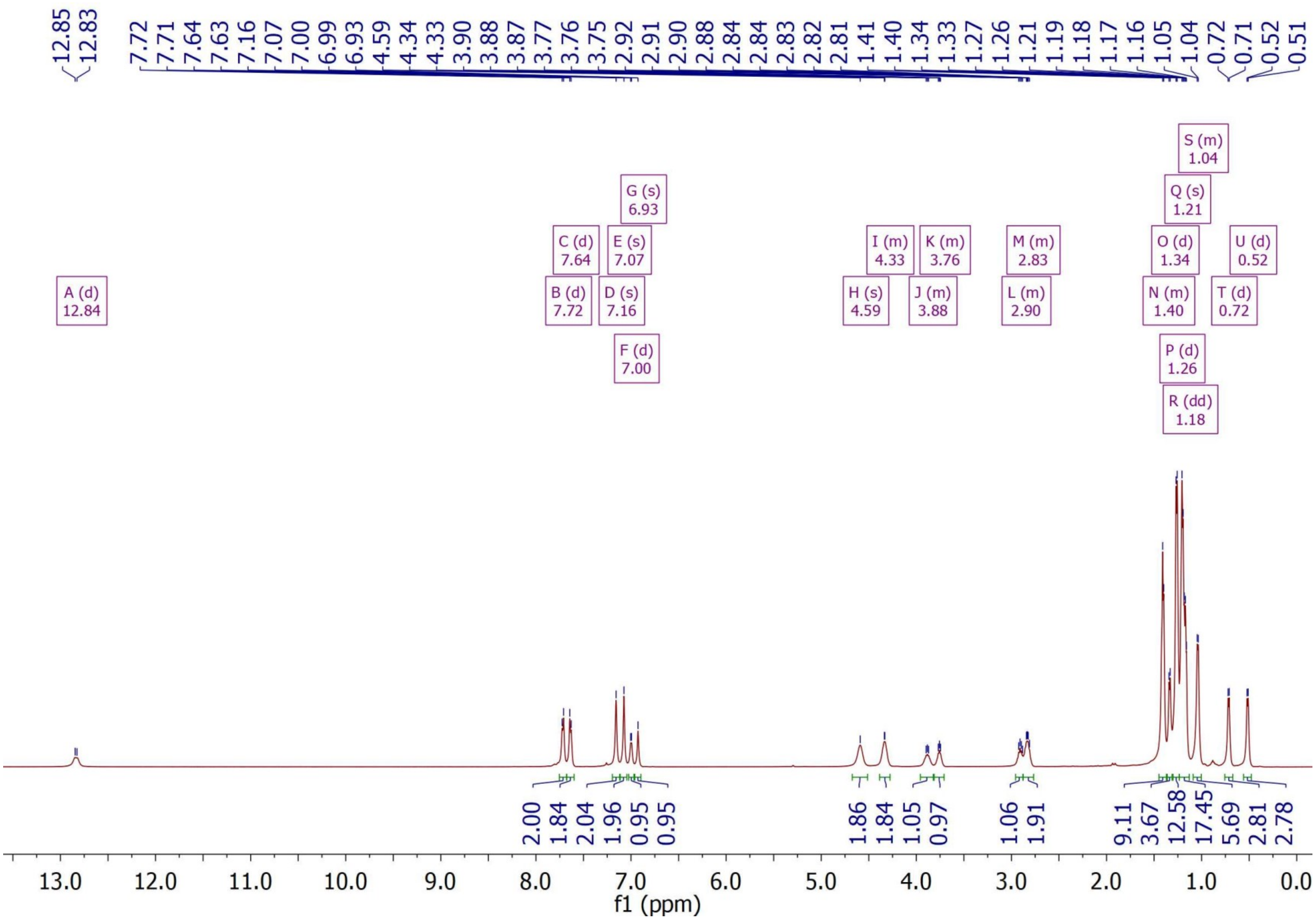

Figure S-34 ¹H NMR spectrum ($CDCl_3$) of compound $[1_{Tipp}]^+$ (R' = *p*-$CF_3C_6H_4$)

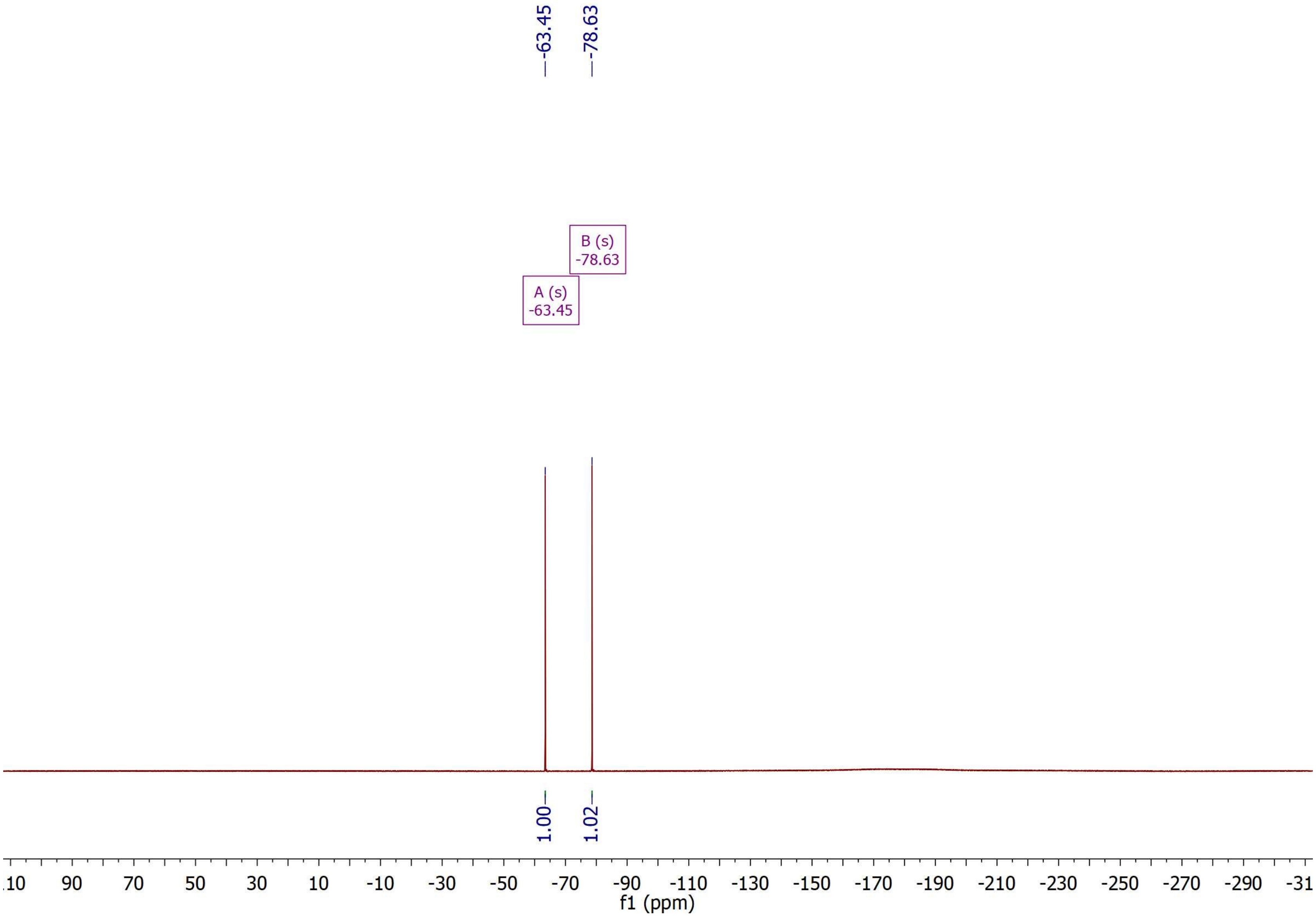

Figure S-35 ^{19}F NMR spectrum ($CDCl_3$) of compound [1_{Tipp}]$^{=}$ (R' = *p*-$CF_3C_6H_4$)

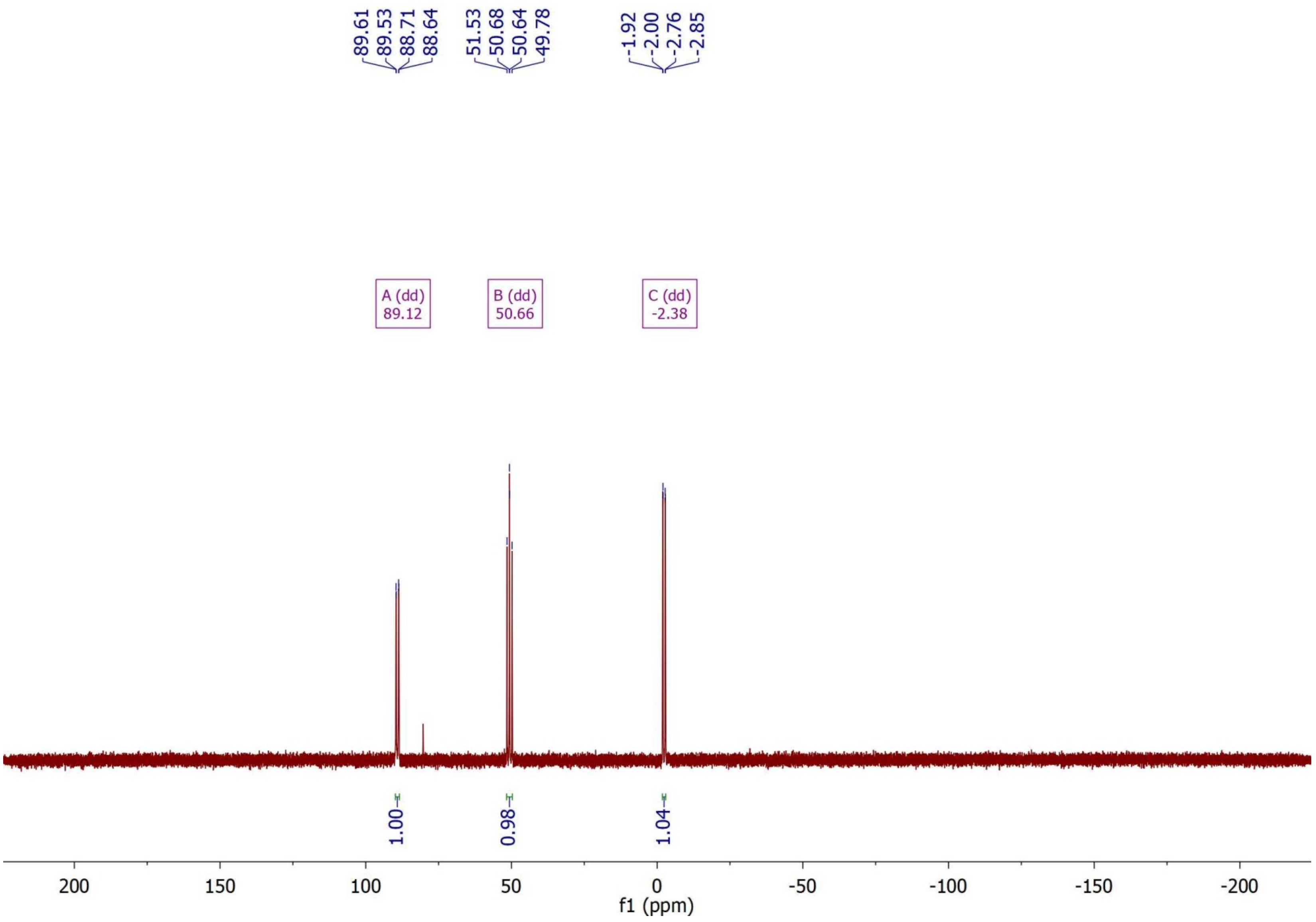

Figure S-36 $^{31}P\{^{1}H\}$ NMR spectrum ($CDCl_3$) of compound $[1_{Tipp}]^+$ (R' = *p*-$CF_3C_6H_4$)

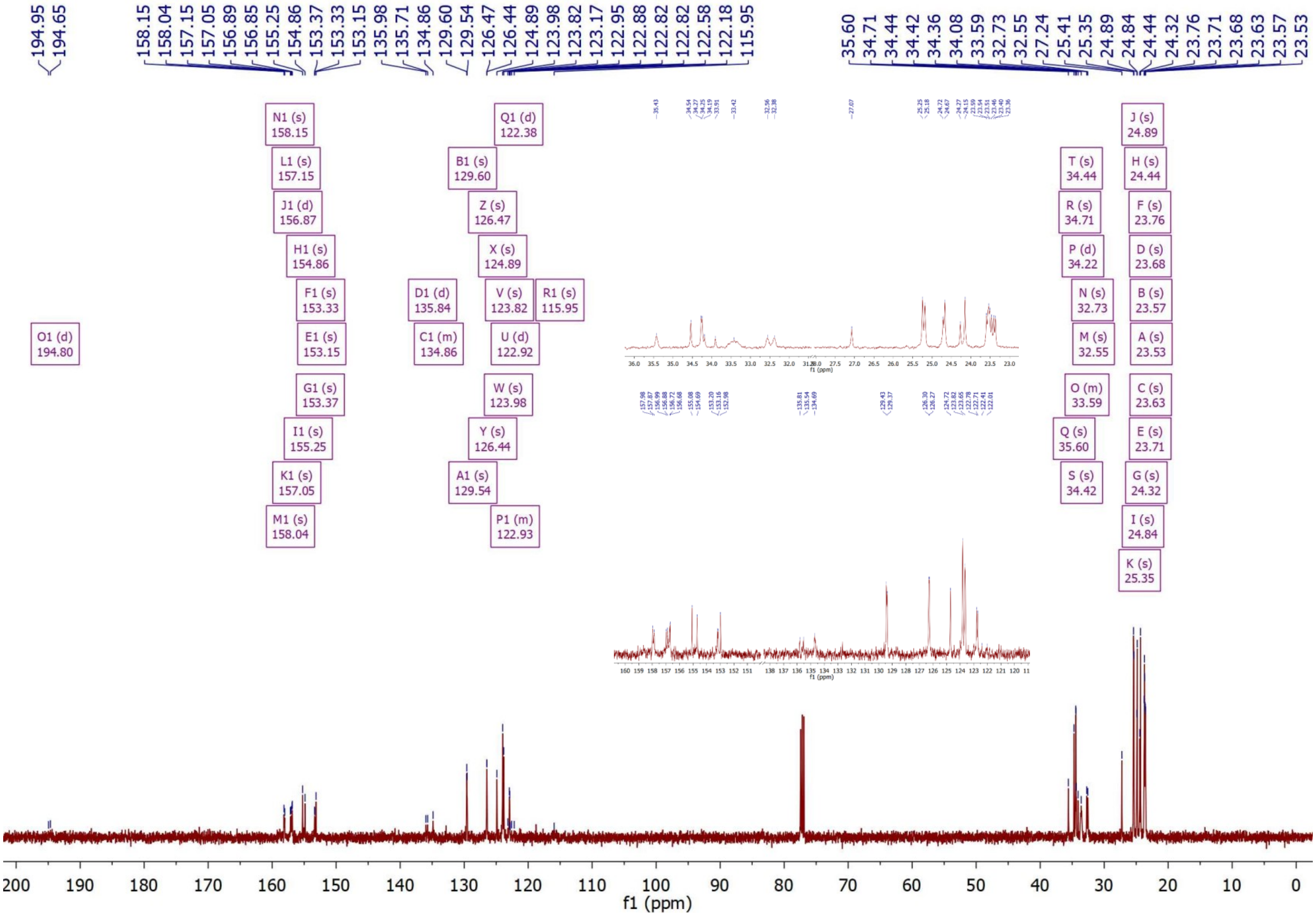

Figure S-37 $^{13}C\{^1H\}$ UDEFT NMR spectrum ($CDCl_3$) of compound $[1_{Tipp}]^+$ (R' = *p*-$CF_3C_6H_4$)

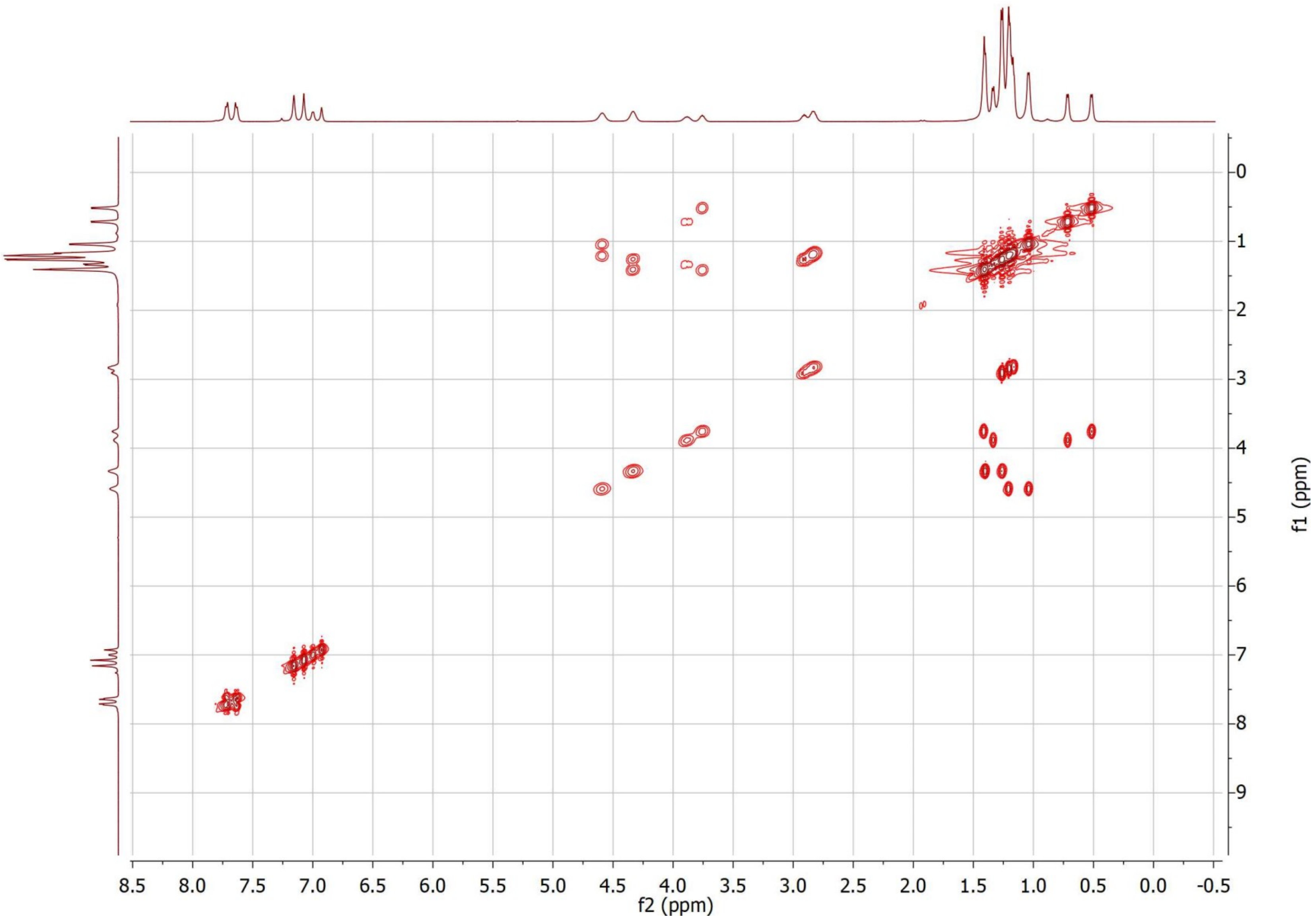

Figure S-38 ^{1}H COSY NMR spectrum ($CDCl_3$) of compound $[1_{Tipp}]^+$ (R' = *p*-$CF_3C_6H_4$)

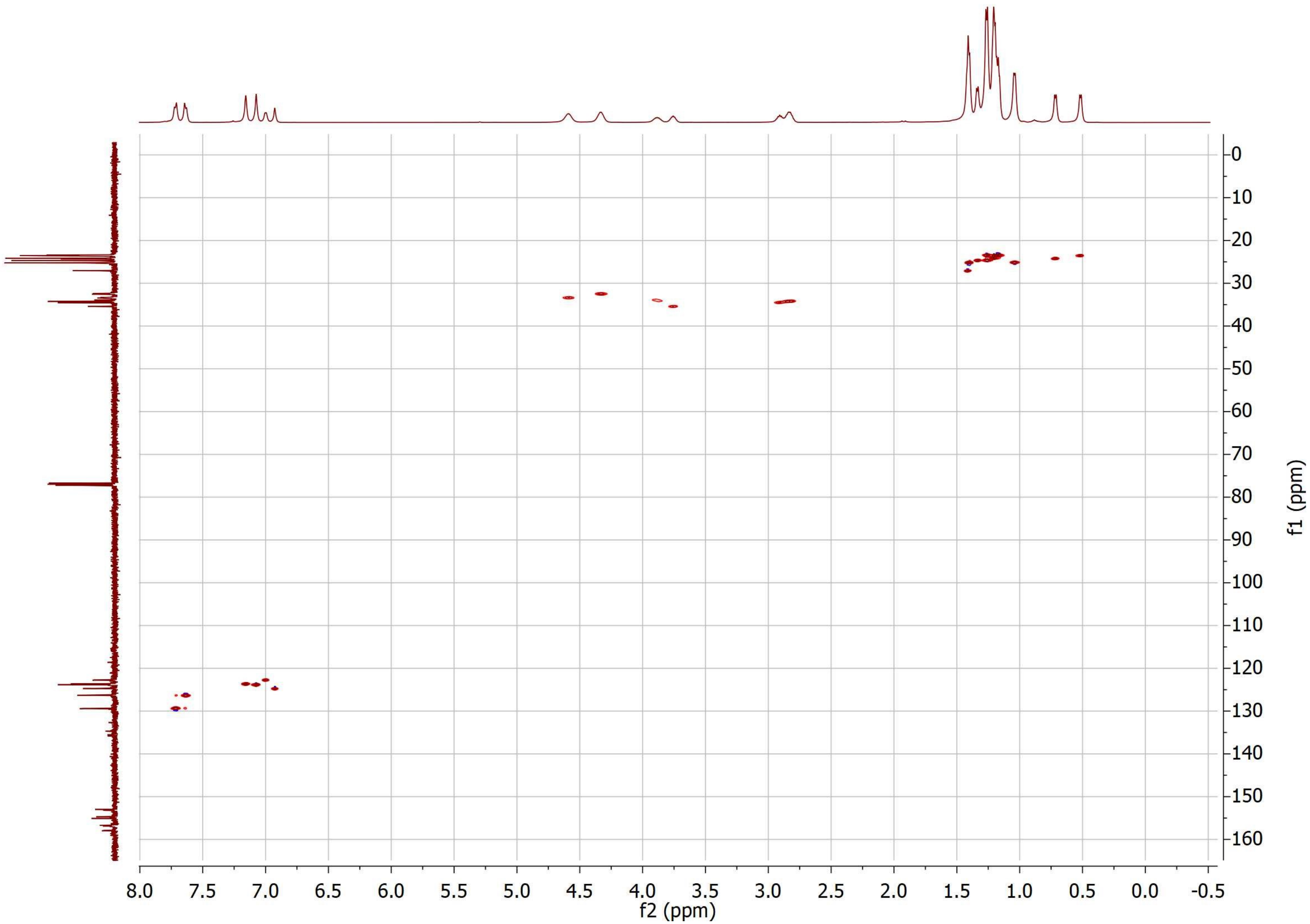

Figure S-39 HSQC NMR spectrum ($CDCl_3$) of compound $[1_{Tipp}]^+$ (R' = *p*-$CF_3C_6H_4$)

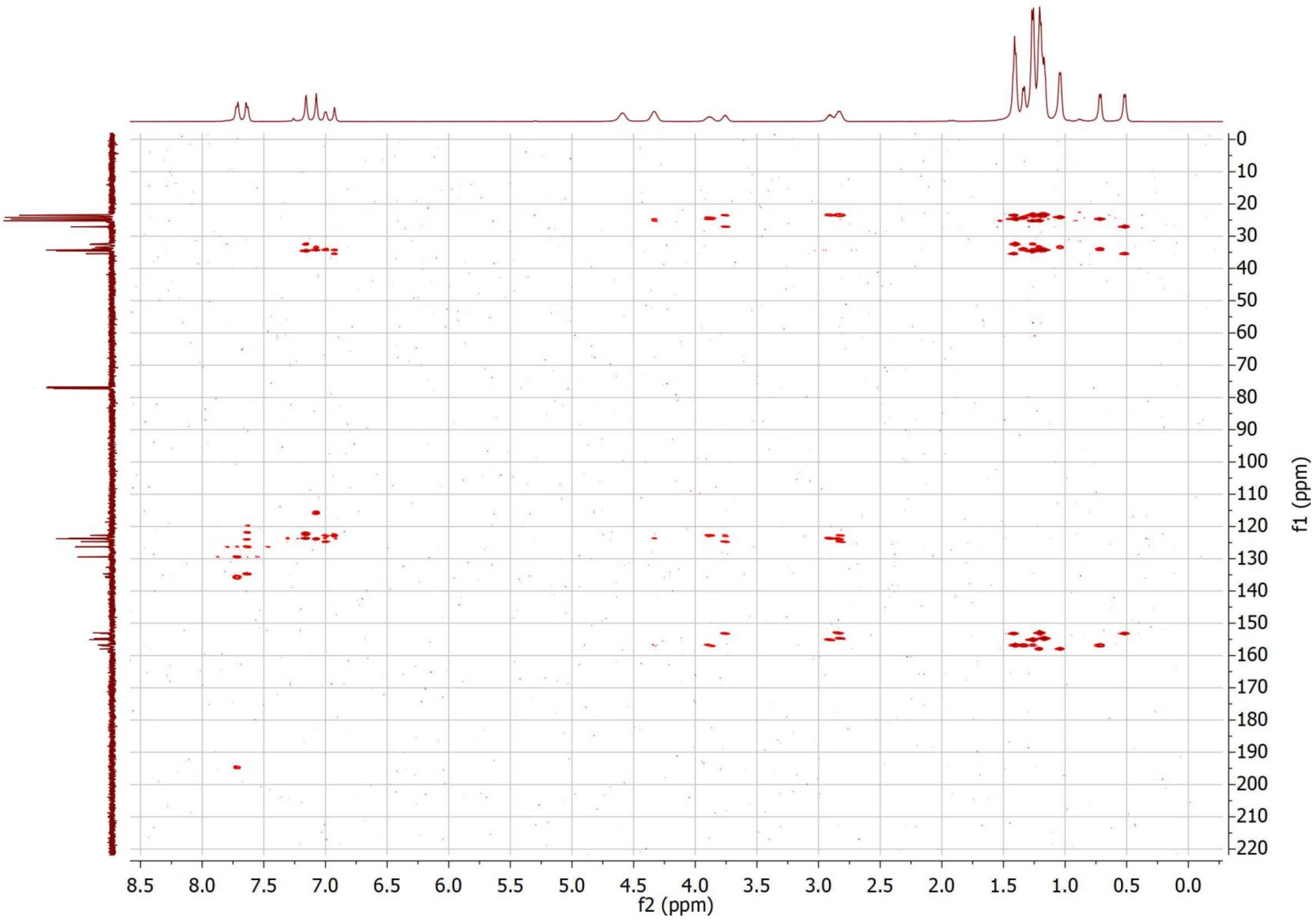

Figure S-40 HMBC NMR spectrum ($CDCl_3$) of compound $[1_{Tipp}]^+$ (R' = *p*-$CF_3C_6H_4$)

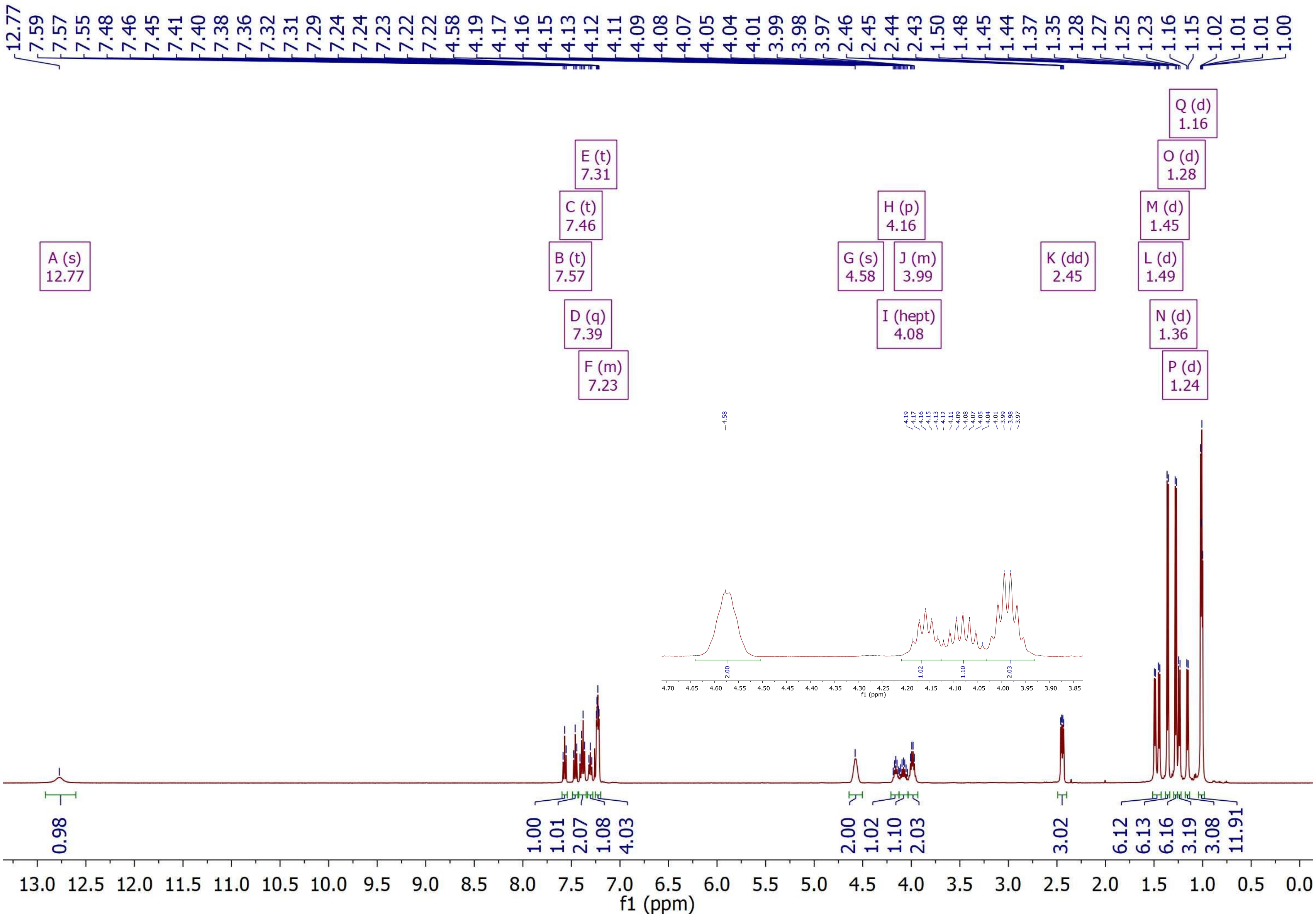

Figure S-41 ^{1}H NMR spectrum ($CDCl_3$) of compound $[1_{Dipp}]^+$ (R' = Me)

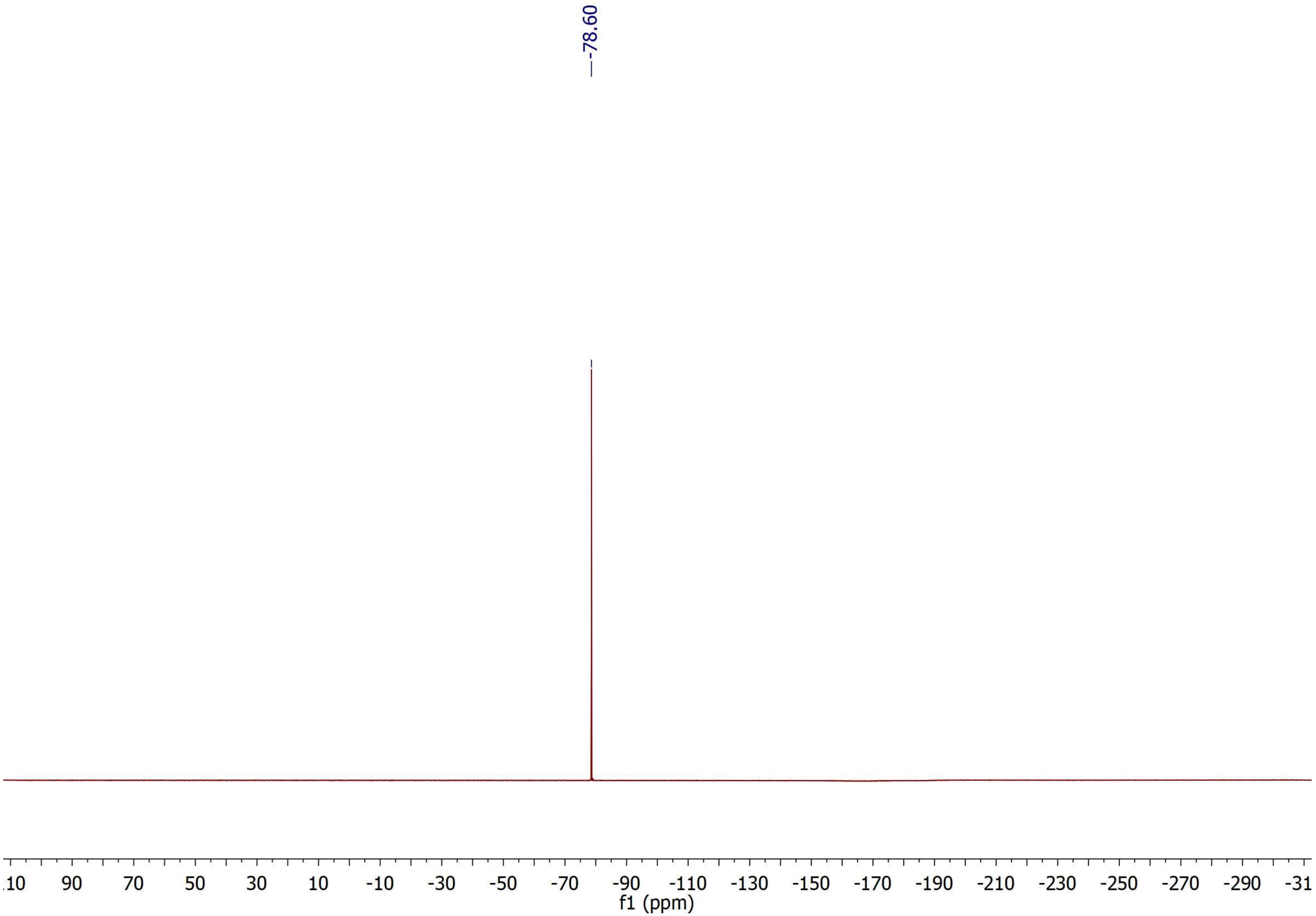

Figure S-42 ^{19}F NMR spectrum ($CDCl_3$) of compound $[1_{Dipp}]^+$ (R' = Me)

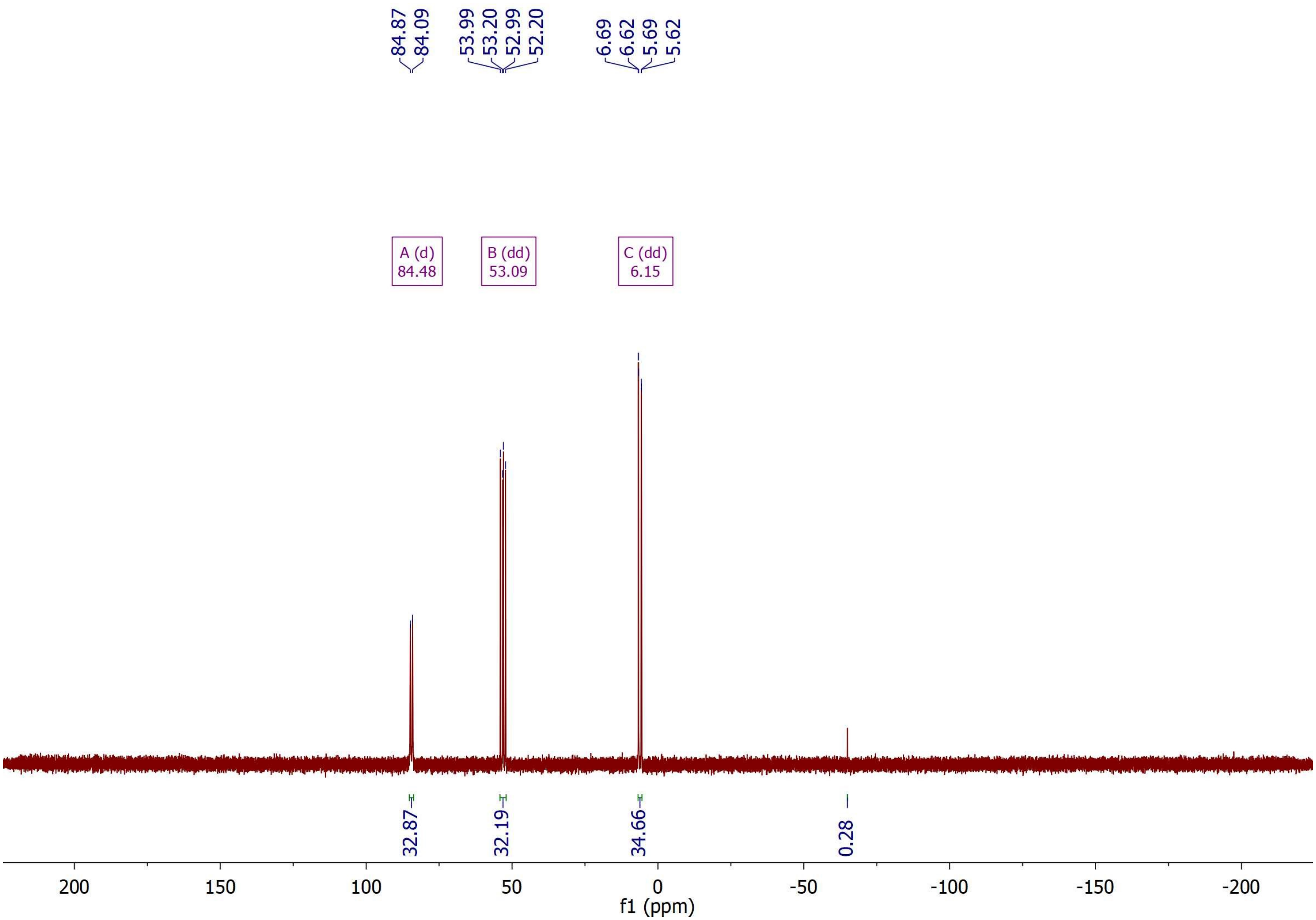

Figure S-43 $^{31}P\{^{1}H\}$ NMR spectrum ($CDCl_3$) of compound $[1_{Dipp}]^{+}$ (R' = Me)

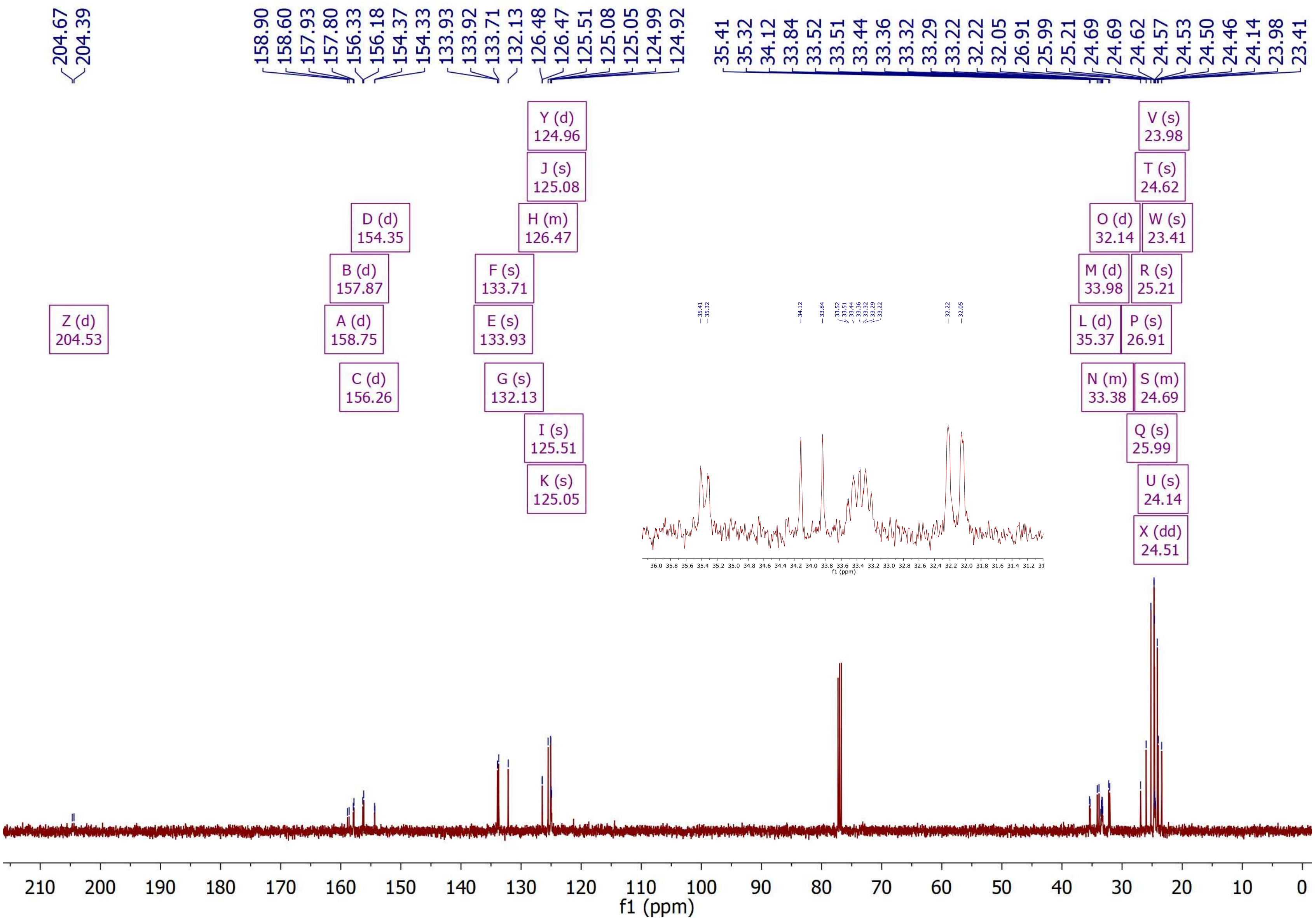

Figure S-44 $^{13}C\{^1H\}$ UDEFT NMR spectrum ($CDCl_3$) of compound $[1_{Dipp}]^+$ (R' = Me)

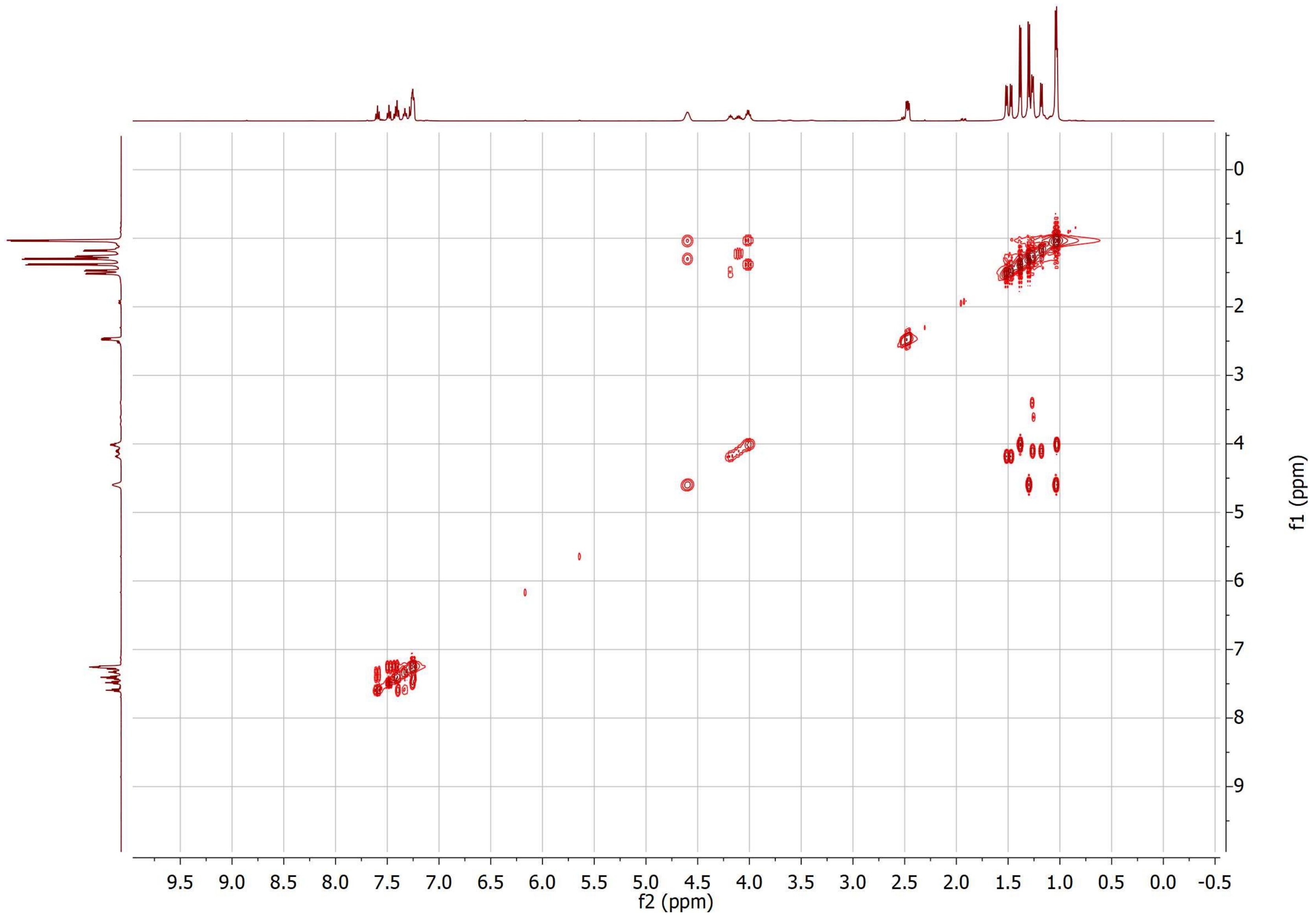

Figure S-45 ^{1}H COSY NMR spectrum (CDCl$_3$) of compound [1_{Dipp}]$^{+}$ (R' = Me)

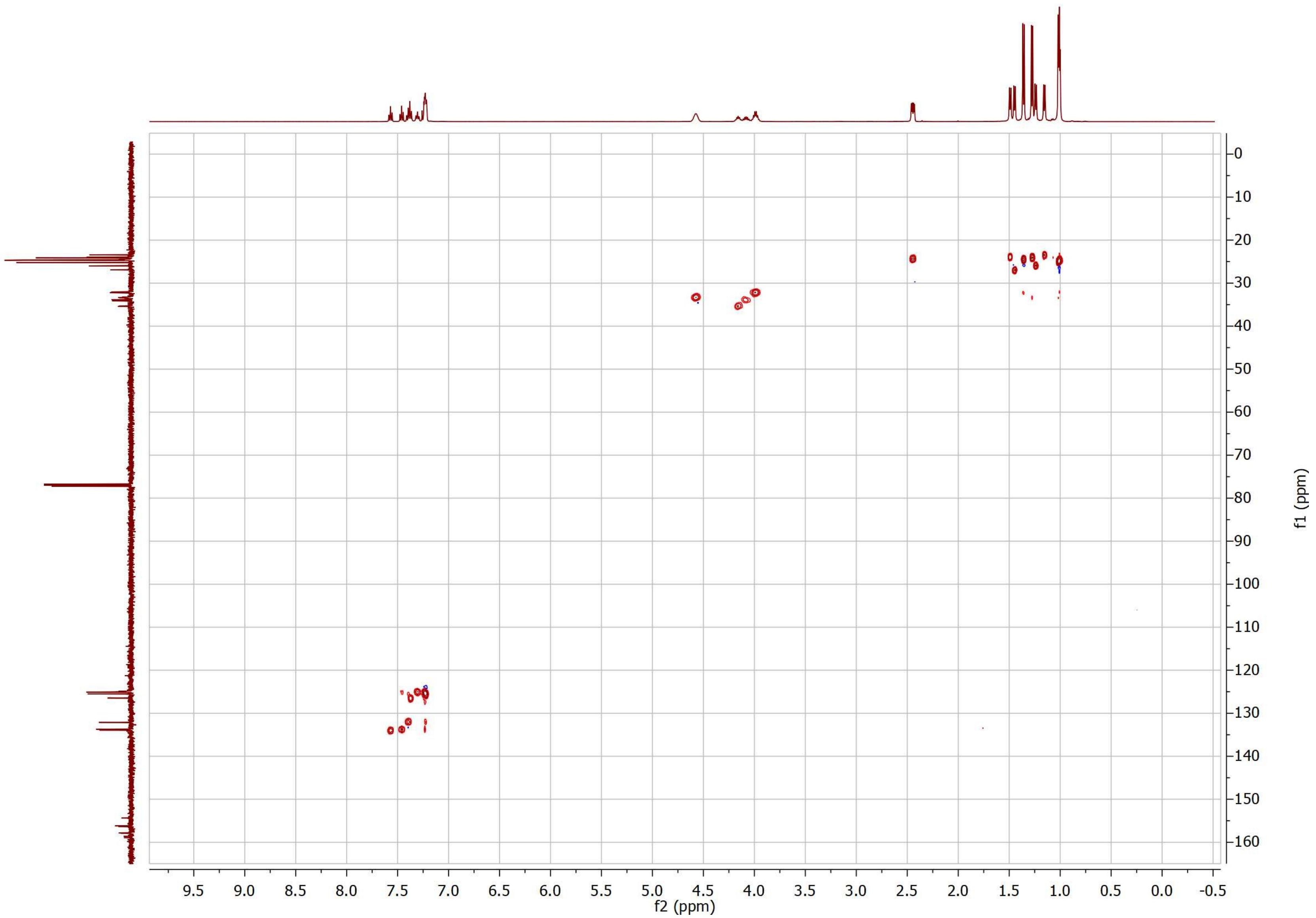

Figure S-46 HSQC NMR spectrum ($CDCl_3$) of compound $[1_{Dipp}]^+$ (R' = Me)

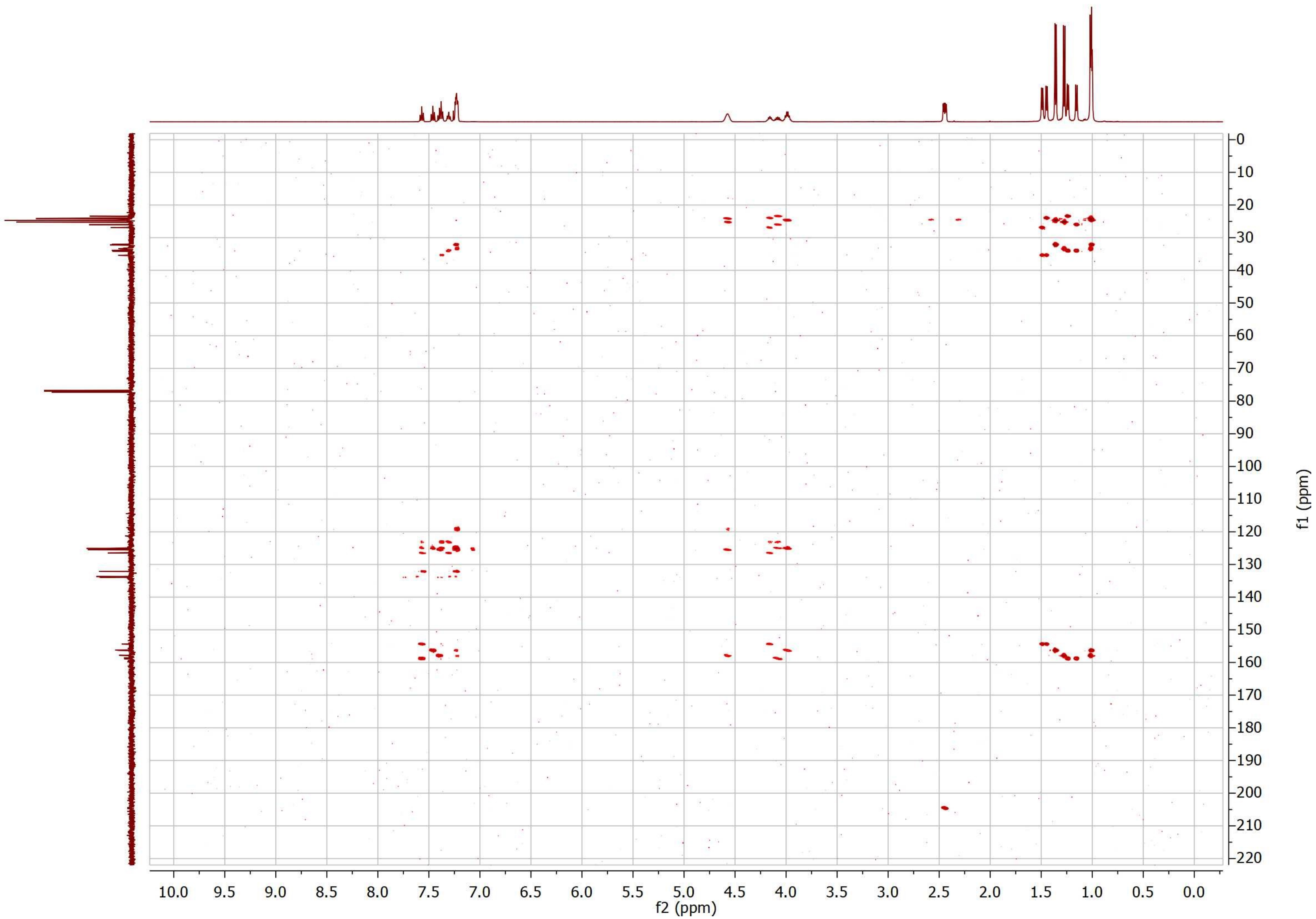

Figure S-47 HMBC NMR spectrum ($CDCl_3$) of compound $[1_{Dipp}]^+$ (R’ = Me)

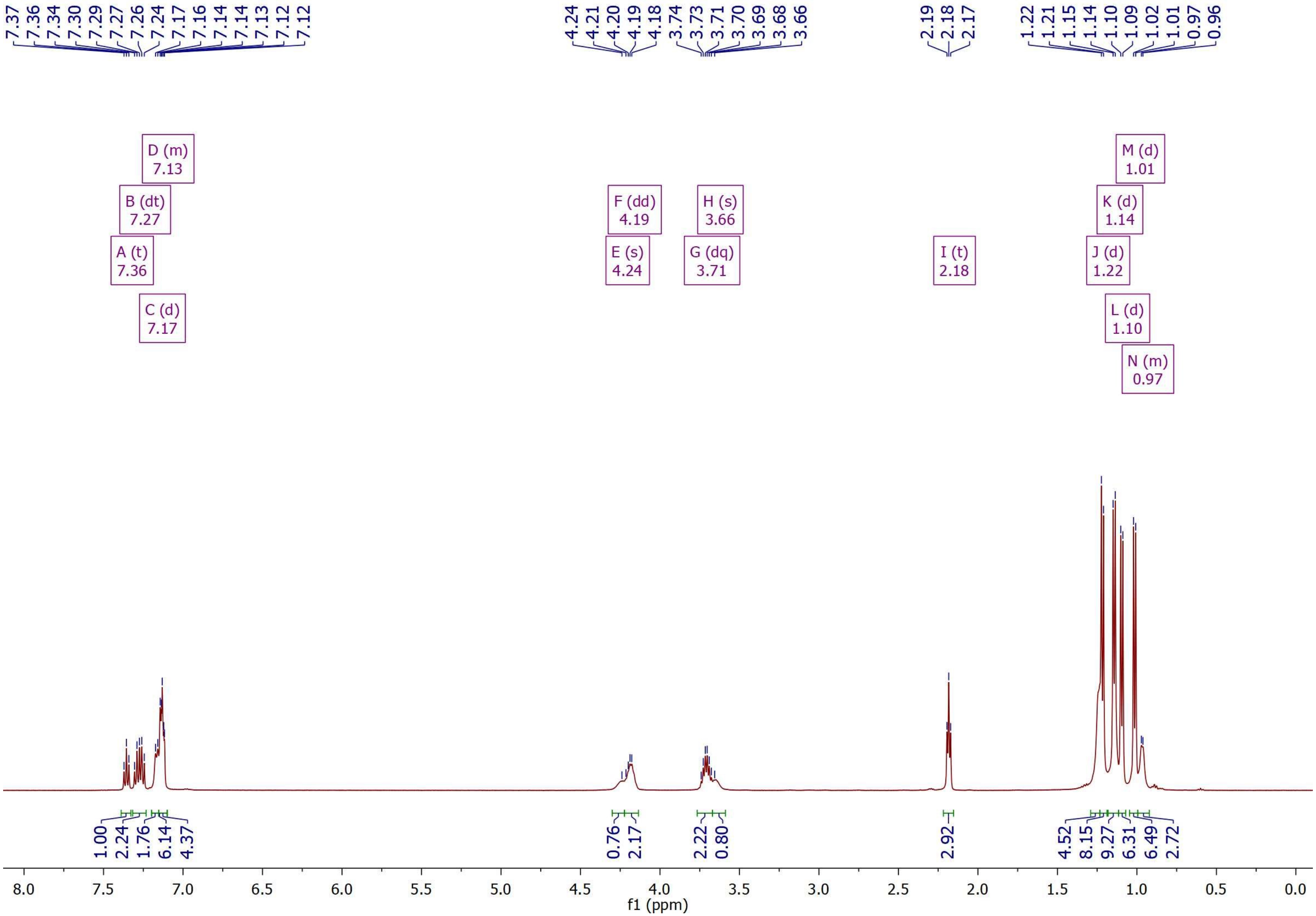

Figure S-48 ^{1}H NMR spectrum ($CDCl_3$) of compound 2_{Dipp} (R' = Me)

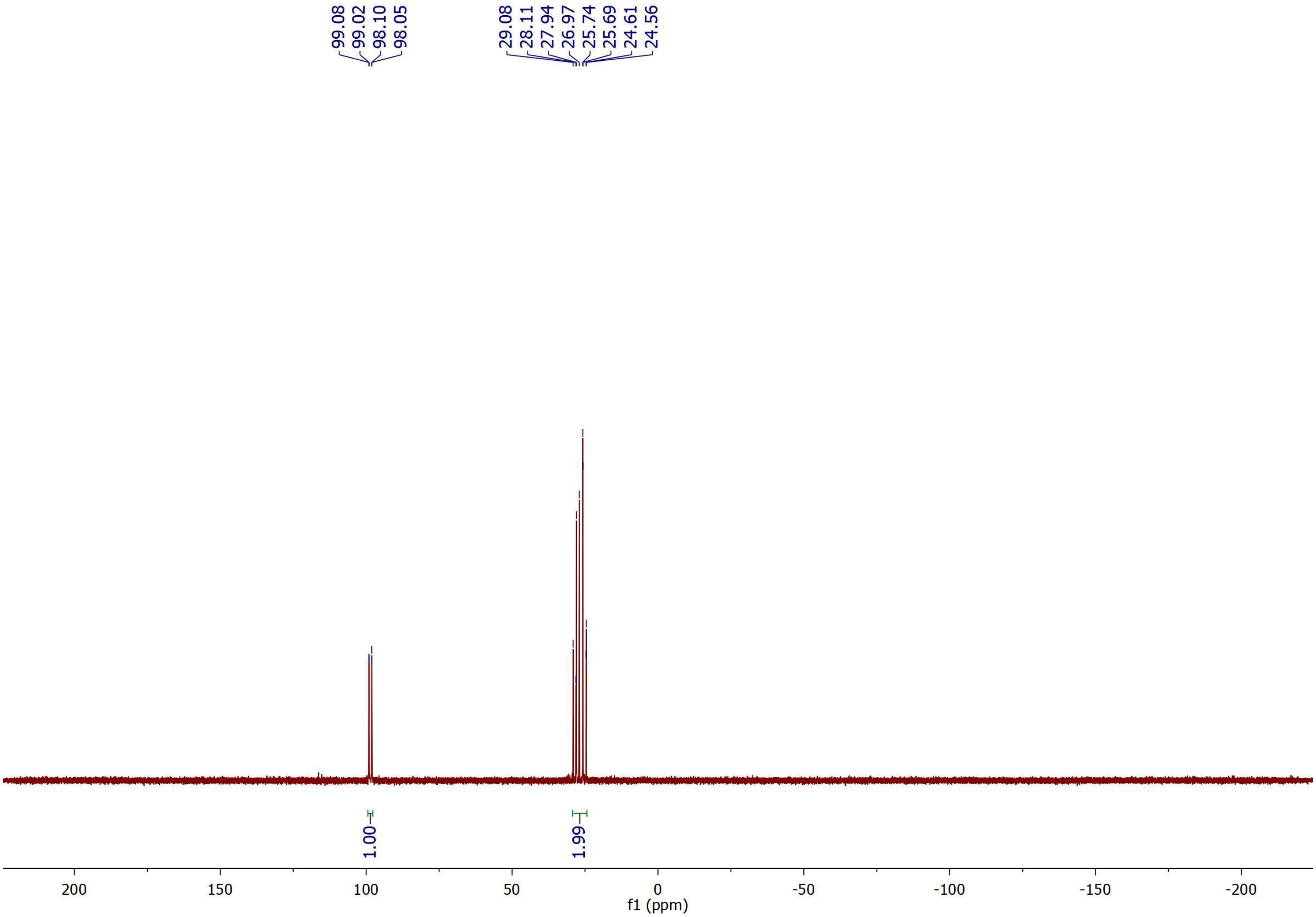

Figure S-49 $^{31}P\{^{1}H\}$ NMR spectrum ($CDCl_3$) of compound 2_{Dipp} (R' = Me)

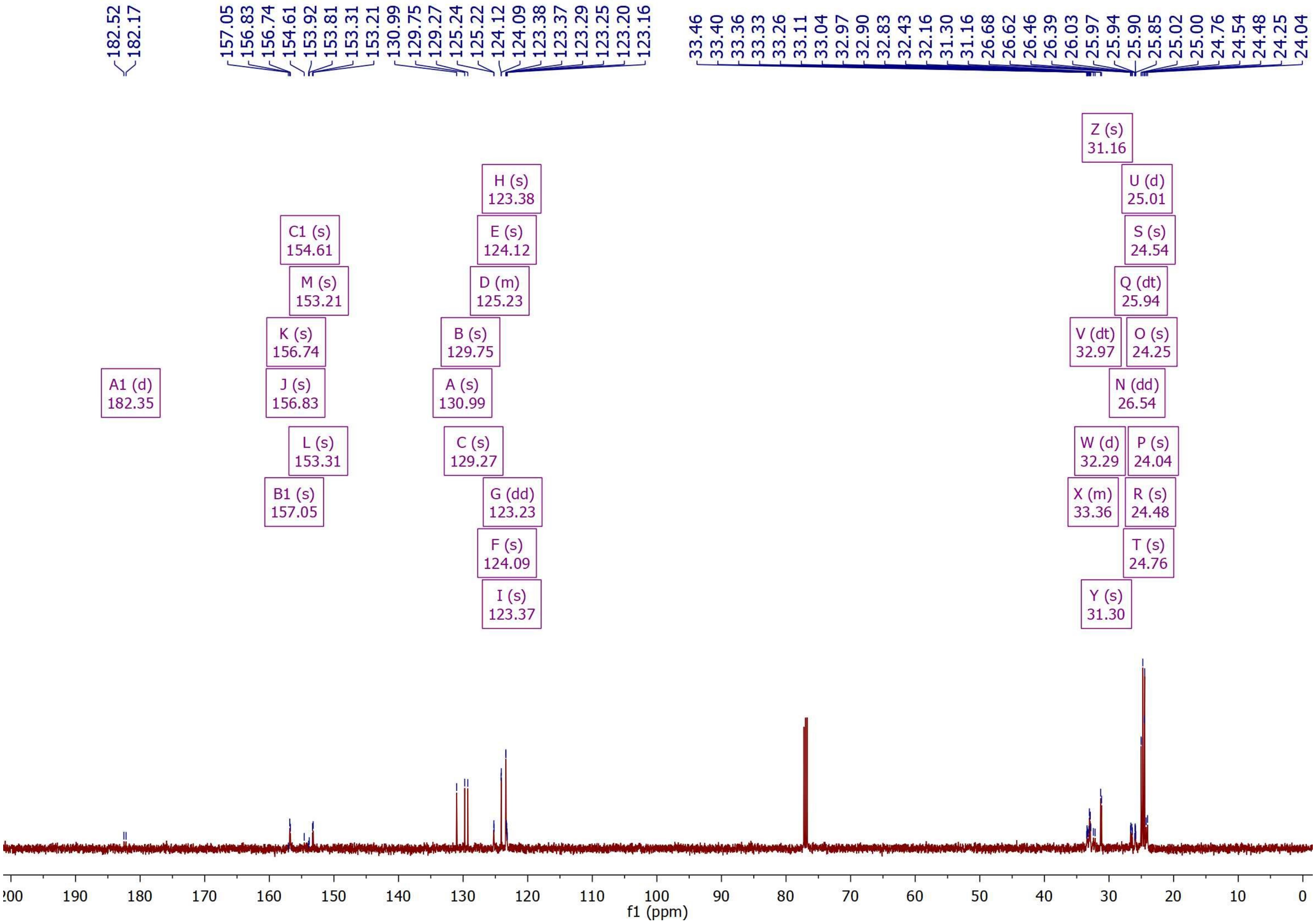

Figure S-50 $^{13}C\{^{1}H\}$ UDEFT NMR spectrum ($CDCl_3$) of compound 2_{Dipp} (R’ = Me)

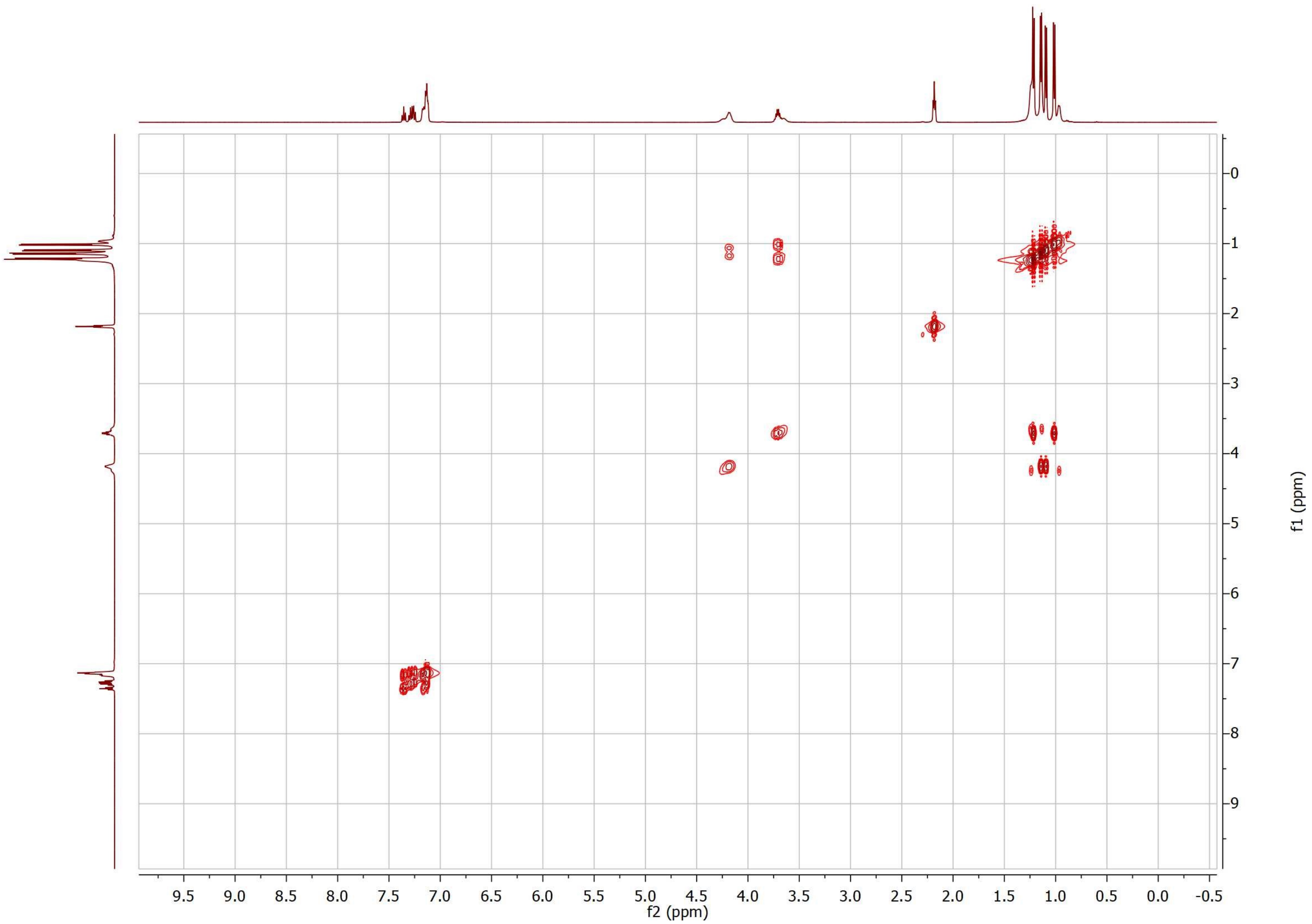

Figure S-51 ^{1}H COSY NMR spectrum ($CDCl_3$) of compound 2_{Dipp} (R' = Me)

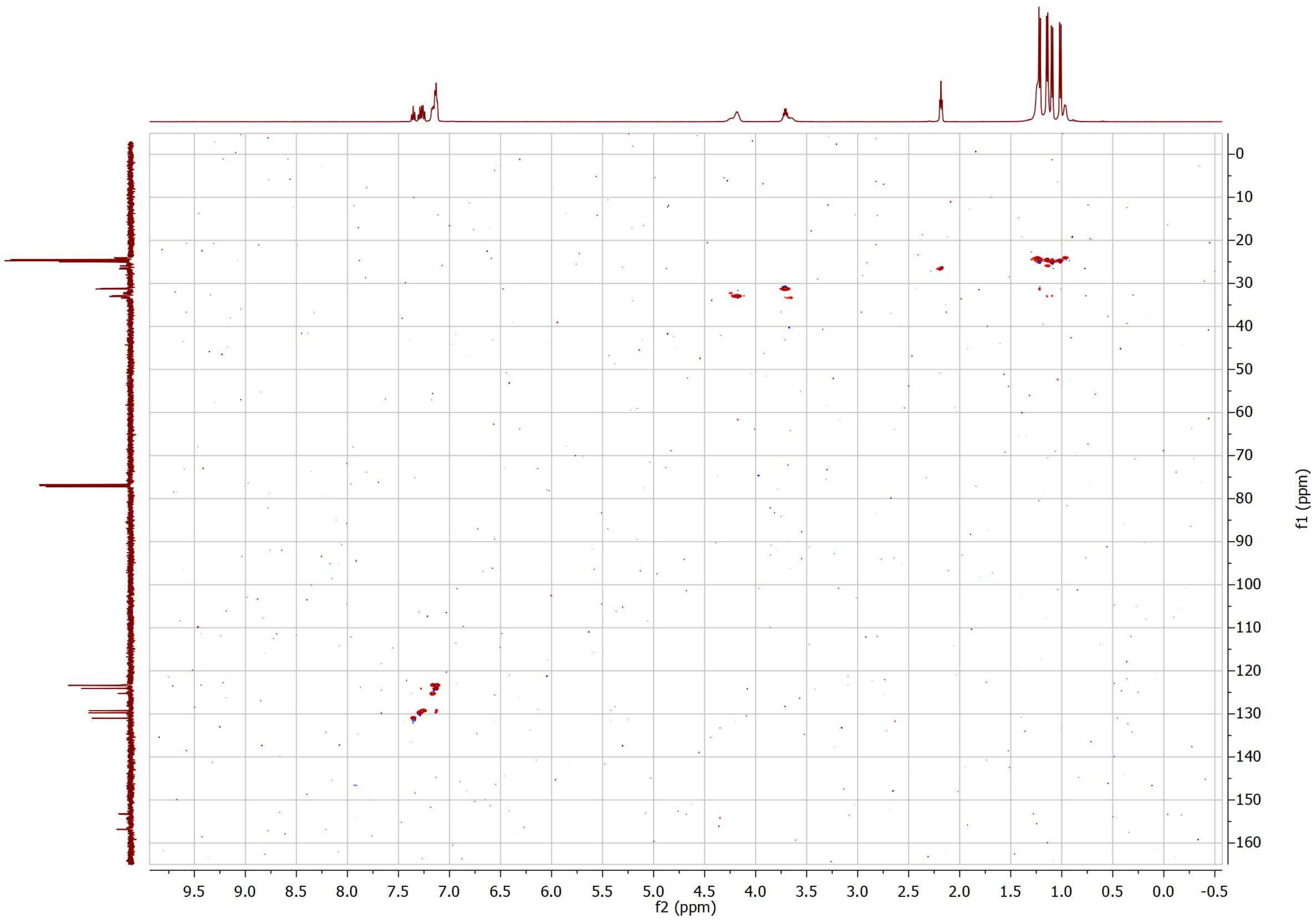

Figure S-52 HSQC NMR spectrum ($CDCl_3$) of compound 2_{Dipp} (R’ = Me)

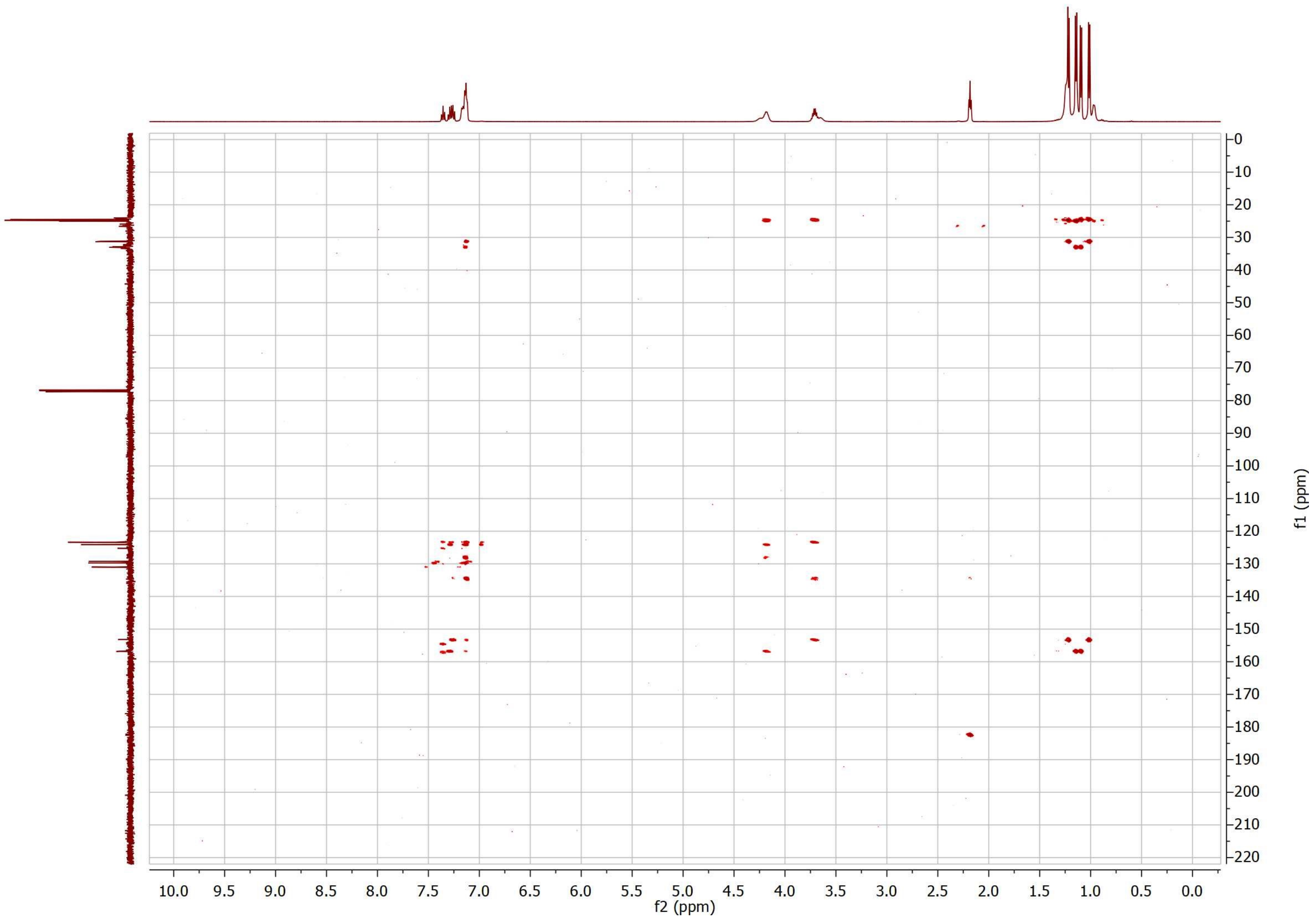

Figure S-53 HMBC NMR spectrum (CDCl3) of compound 2_{Dipp} (R' = Me)

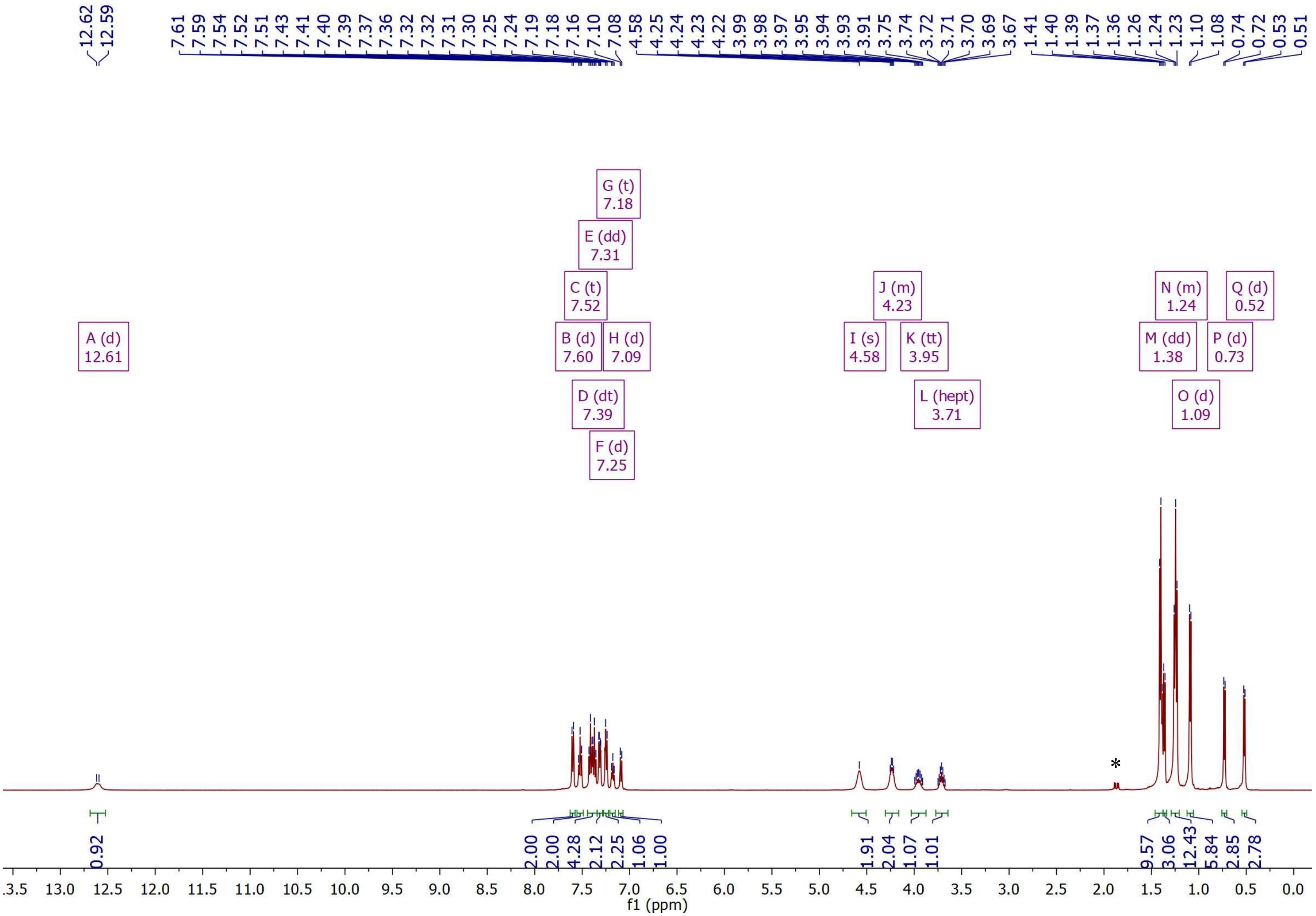

Figure S-54 ^{1}H NMR spectrum ($CDCl_3$) of compound $[1_{Dipp}]^{+}$ (R' = Ph) (* = consistent impurity)

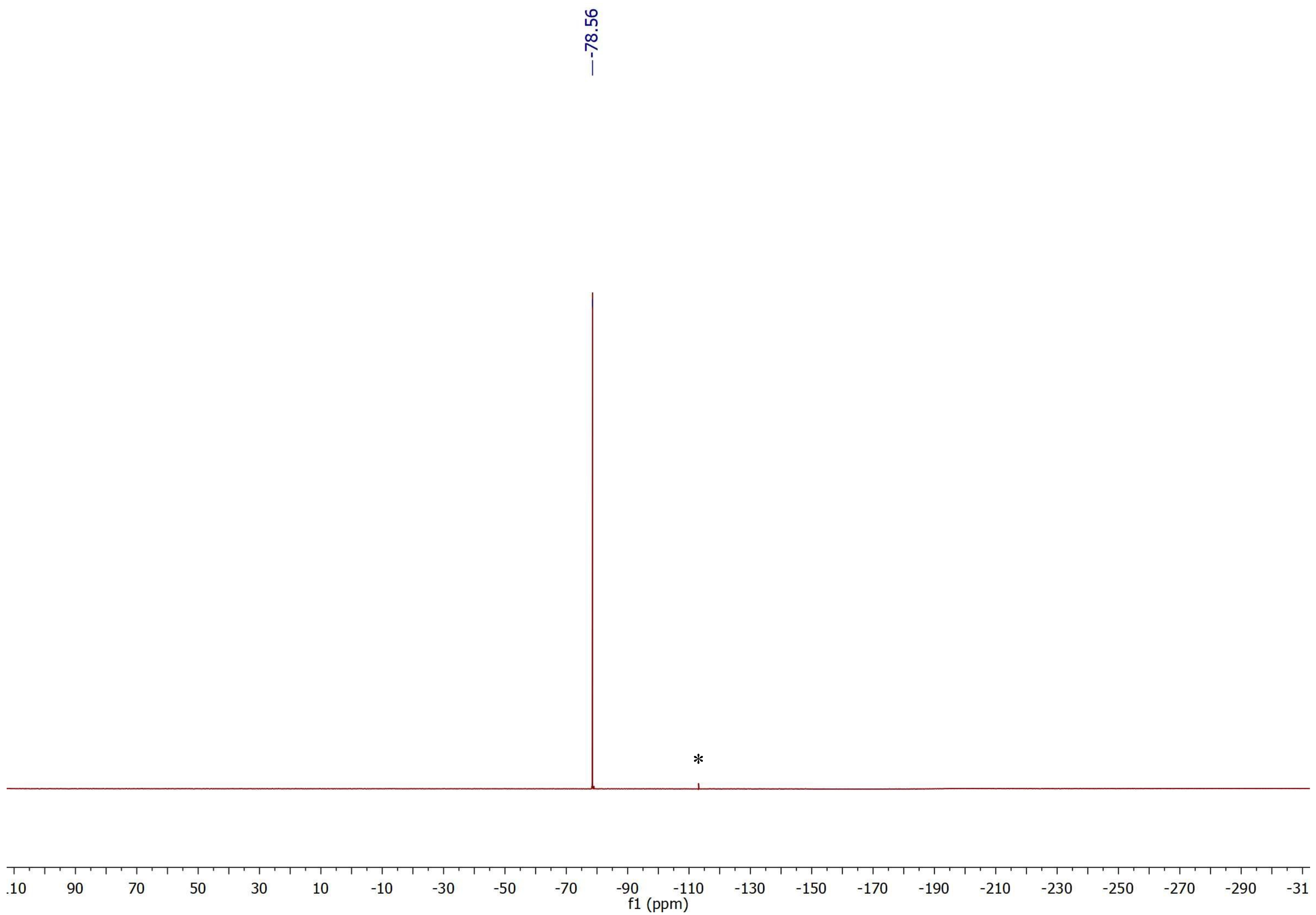

Figure S-55 ^{19}F NMR spectrum (CDCl$_3$) of compound [1_{Dipp}]$^+$ (R' = Ph) (* = unknown impurity)

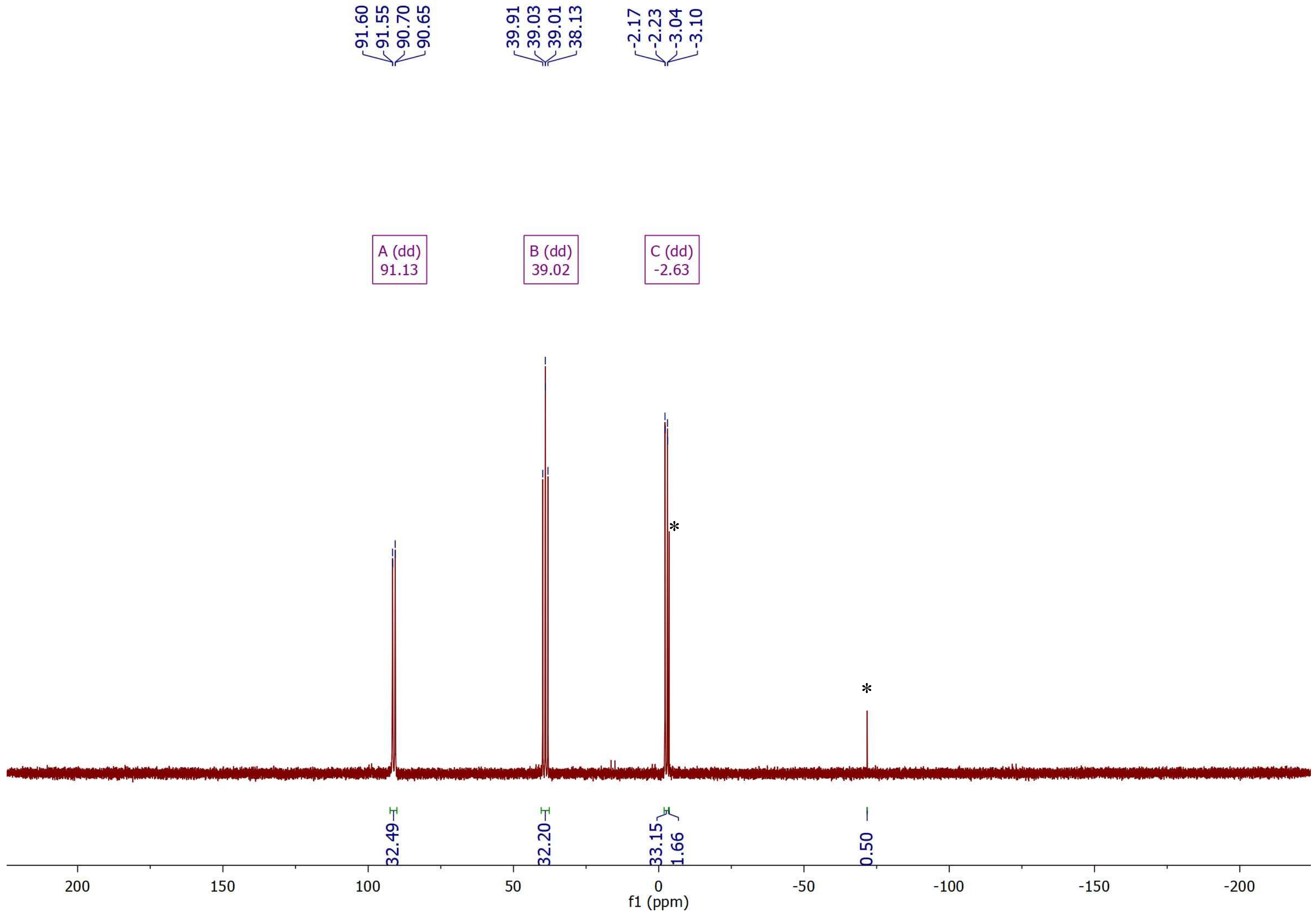

Figure S-56 $^{31}P\{^{1}H\}$ NMR spectrum (CDCl$_3$) of compound $[1_{Dipp}]^+$ (R' = Ph) (* = consistent impurity)

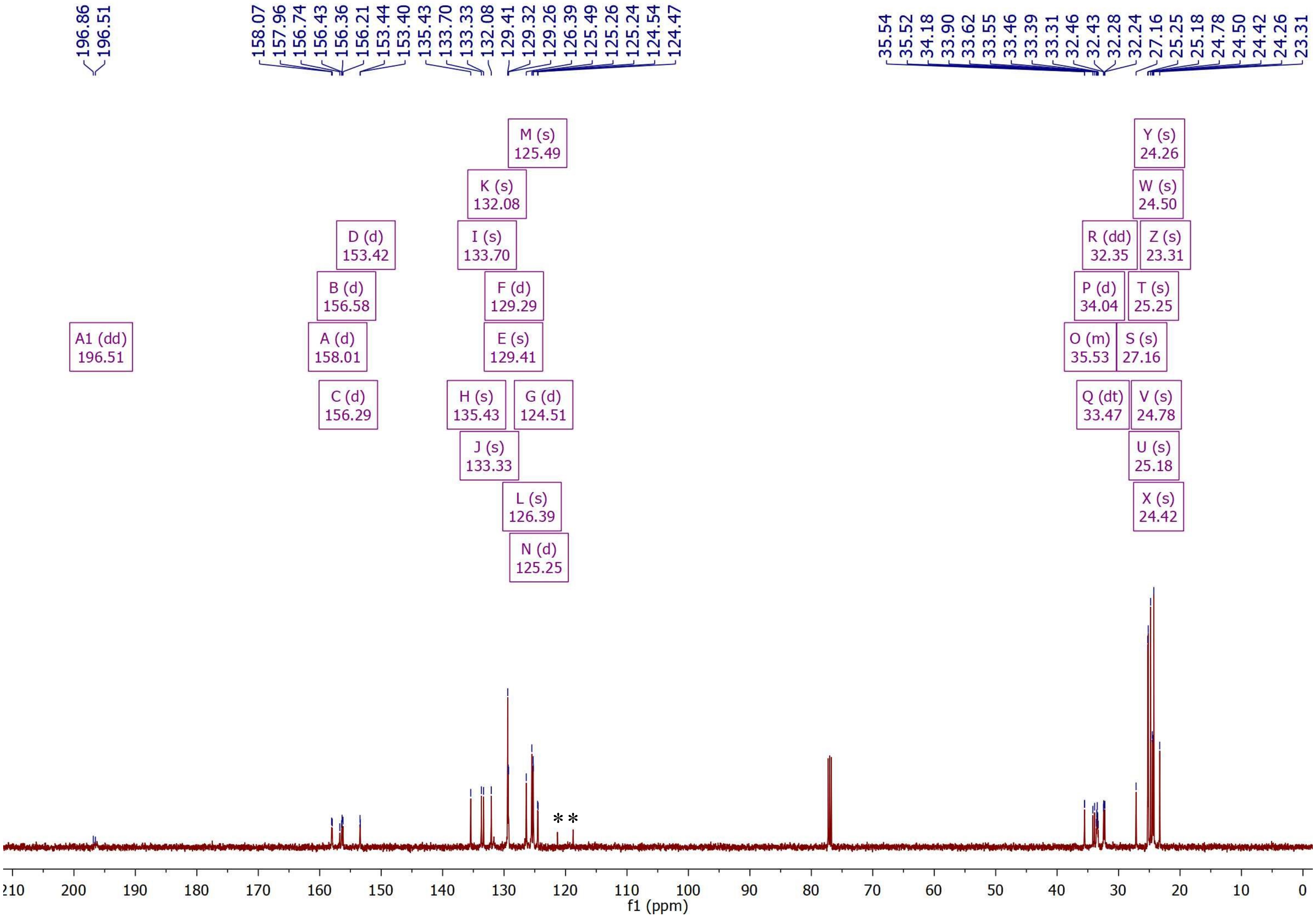

Figure S-57 $^{13}C\{^{1}H\}$ UDEFT NMR spectrum ($CDCl_3$) of compound $[1_{Dipp}]^+$ (R' = Ph) (* = consistent impurity)

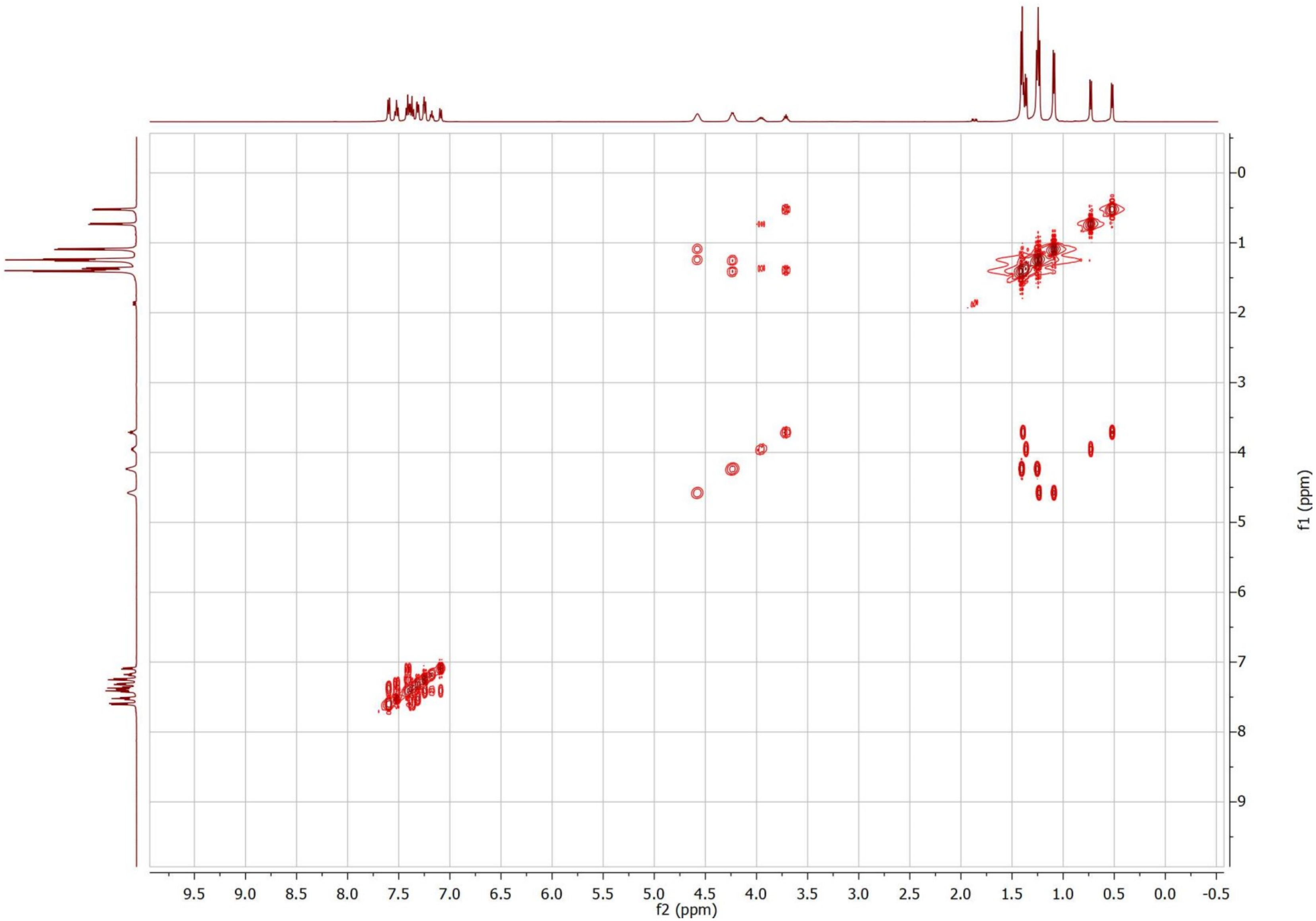

Figure S-58 ^{1}H COSY NMR spectrum ($CDCl_3$) of compound $[1_{Dipp}]^+$ (R' = Ph)

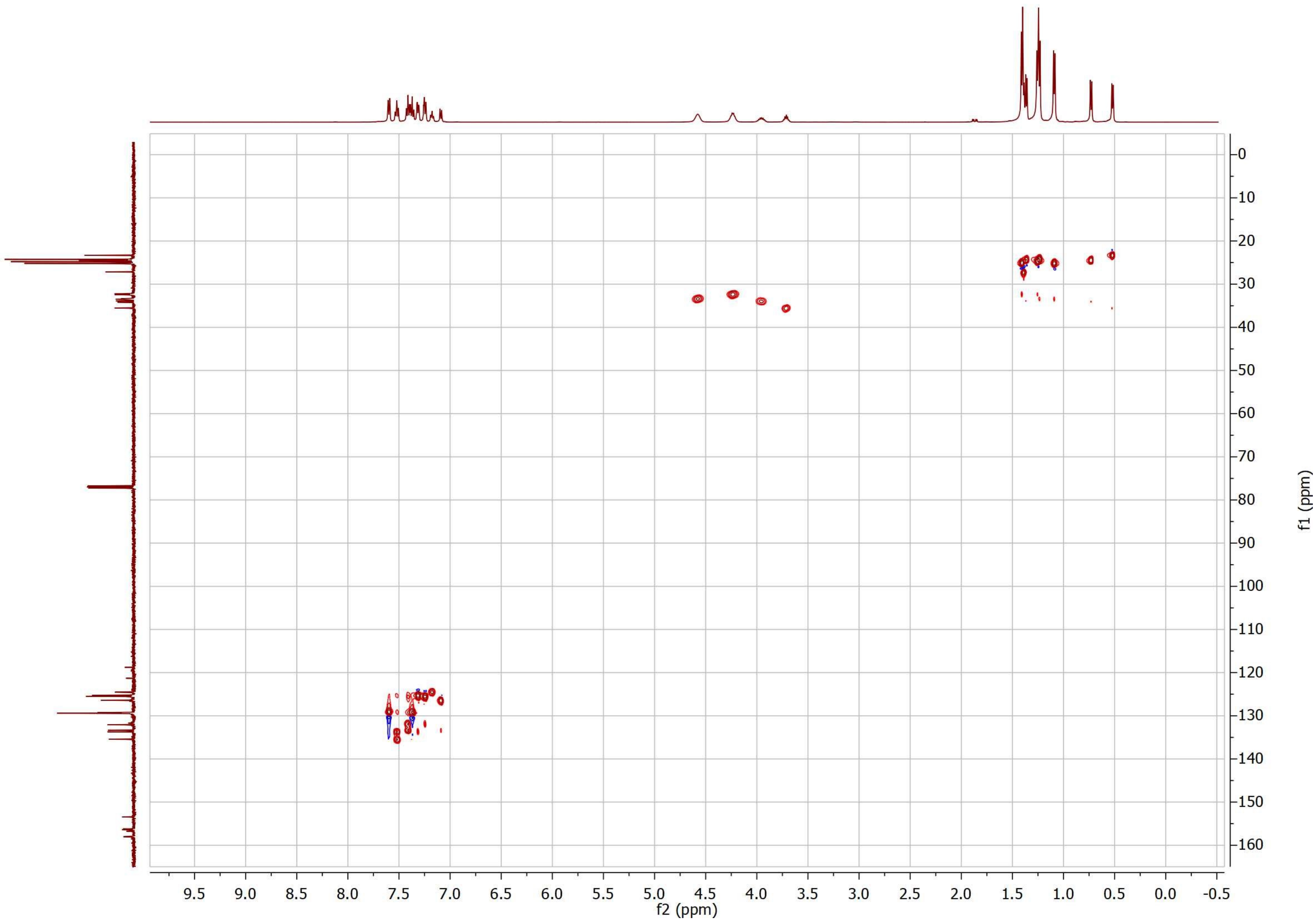

Figure S-59 HSQC NMR spectrum ($CDCl_3$) of compound $[1_{Dipp}]^+$ (R' = Ph)

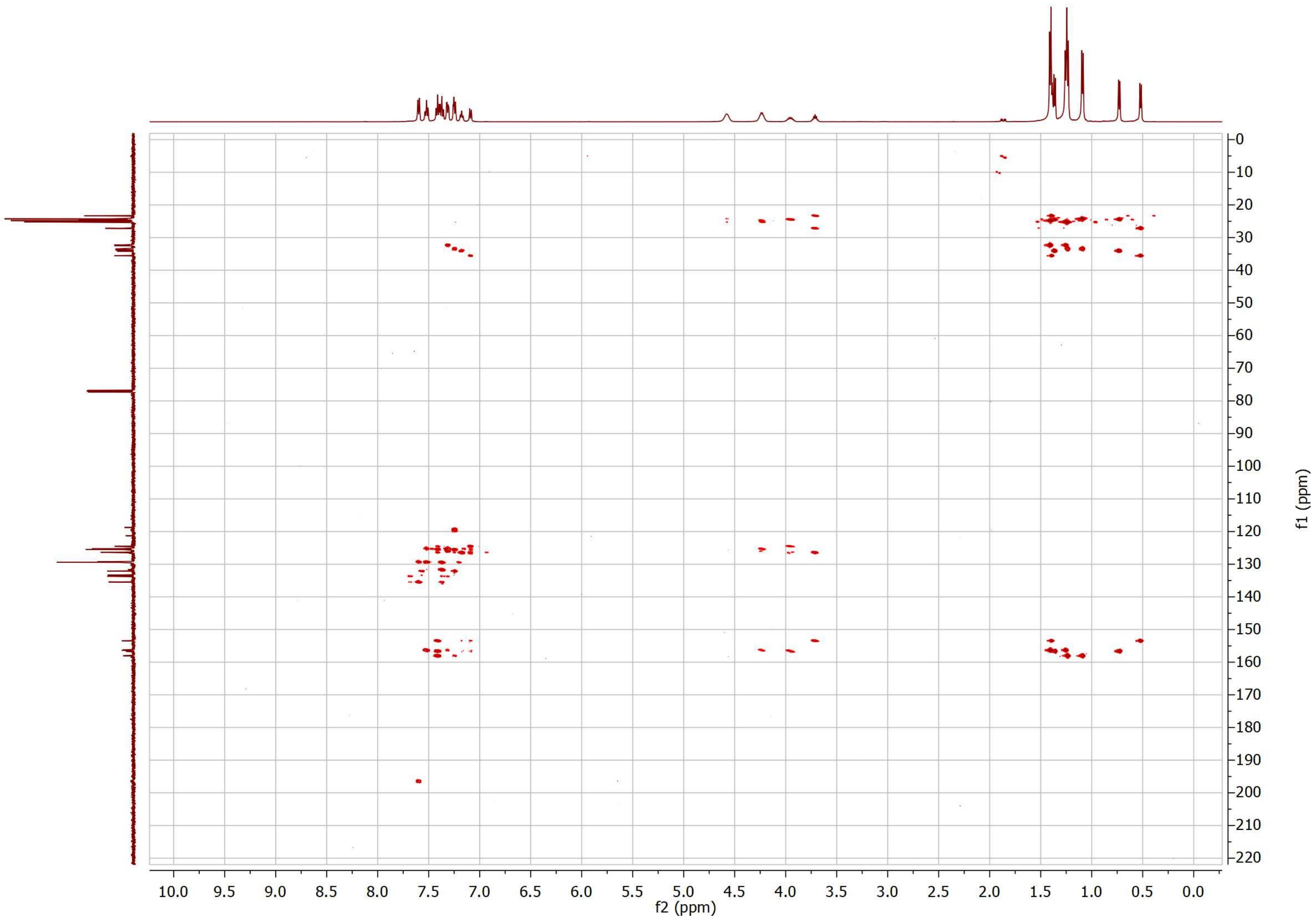

Figure S-60 HMBC NMR spectrum ($CDCl_3$) of compound $[1_{Dipp}]^+$ (R' = Ph)

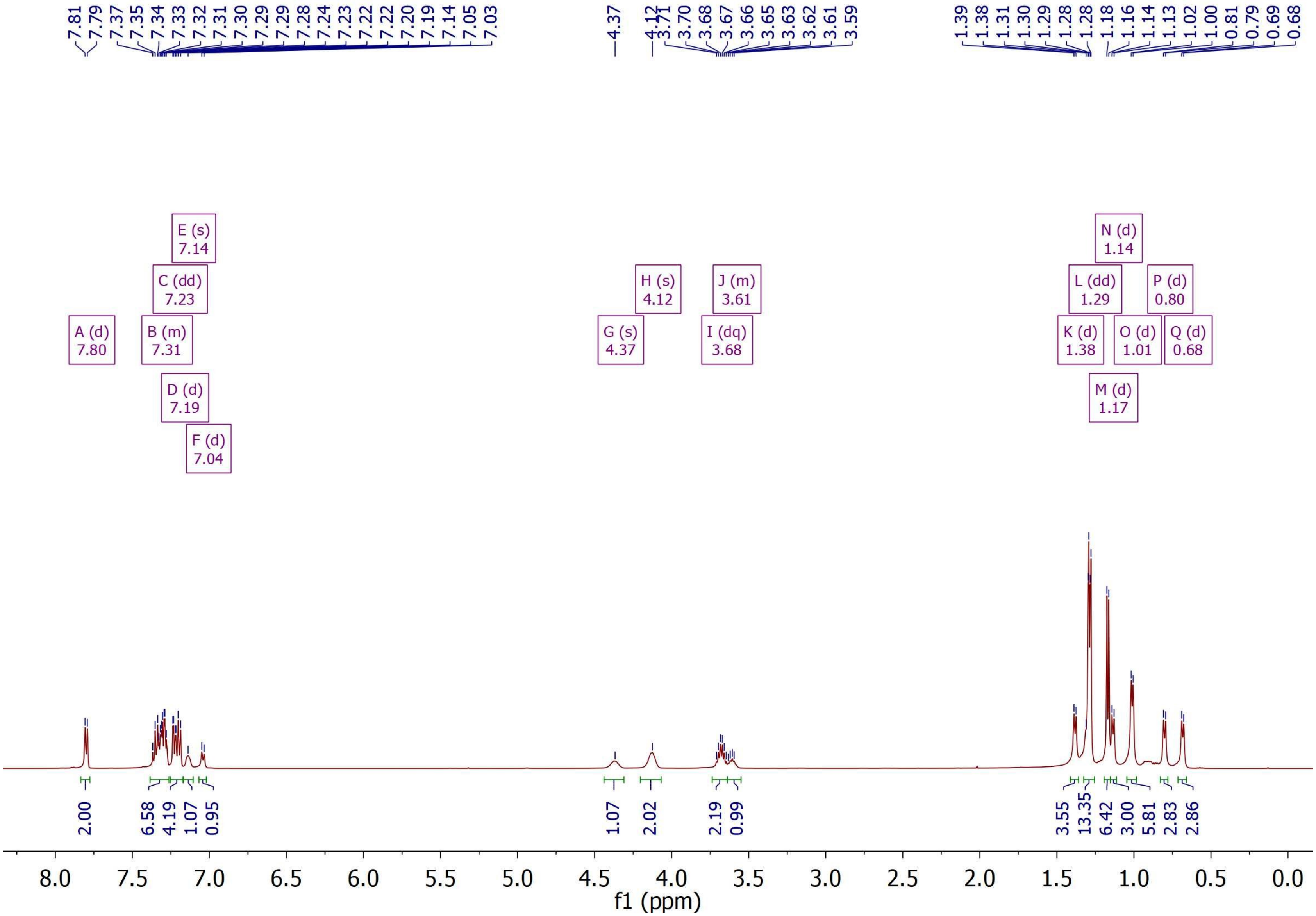

Figure S-61 ^{1}H NMR spectrum ($CDCl_3$) of compound 2_{Dipp} (R' = Ph)

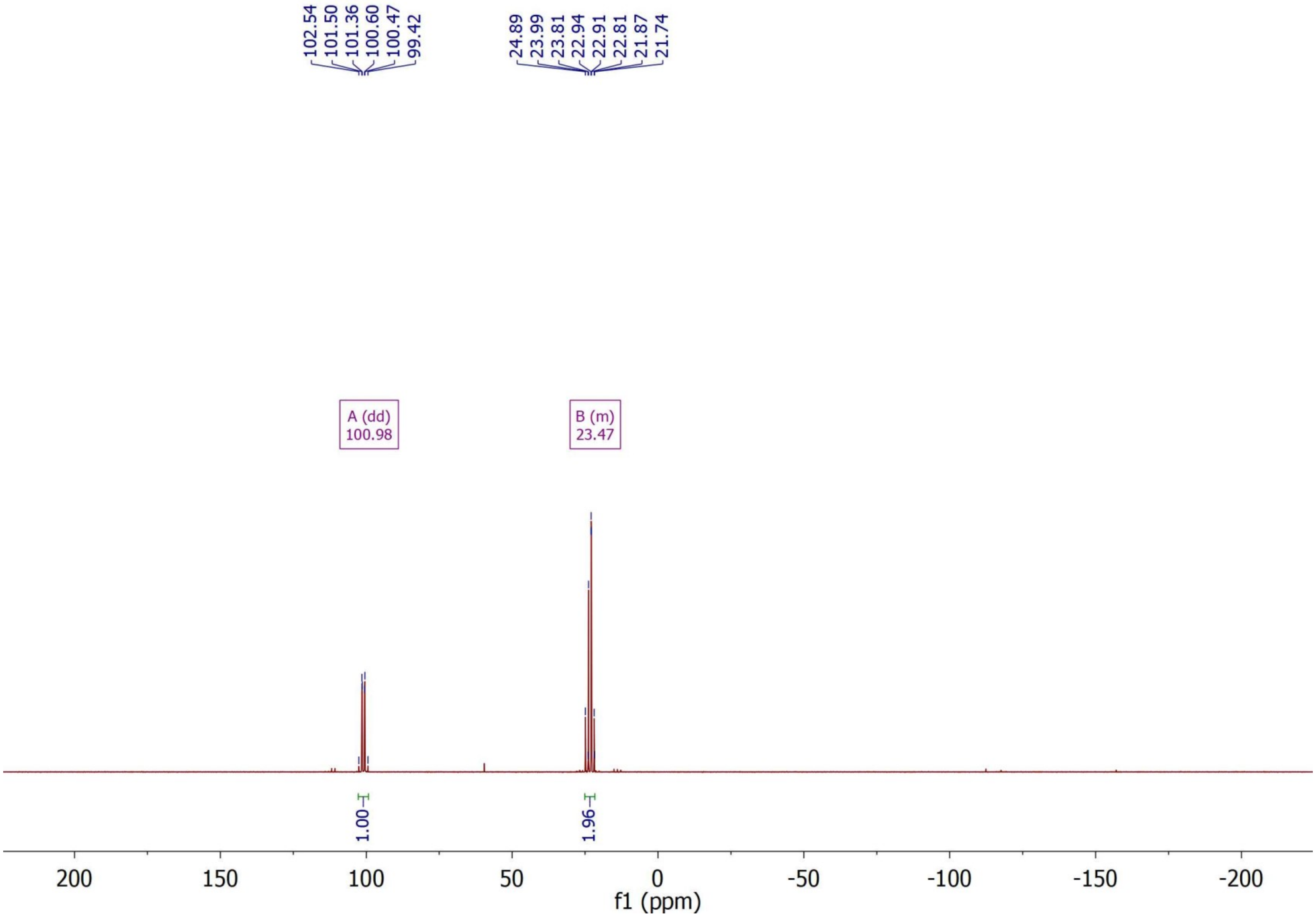

Figure S-62 $^{31}P\{^{1}H\}$ NMR spectrum ($CDCl_3$) of compound 2_{Dipp} (R' = Ph)

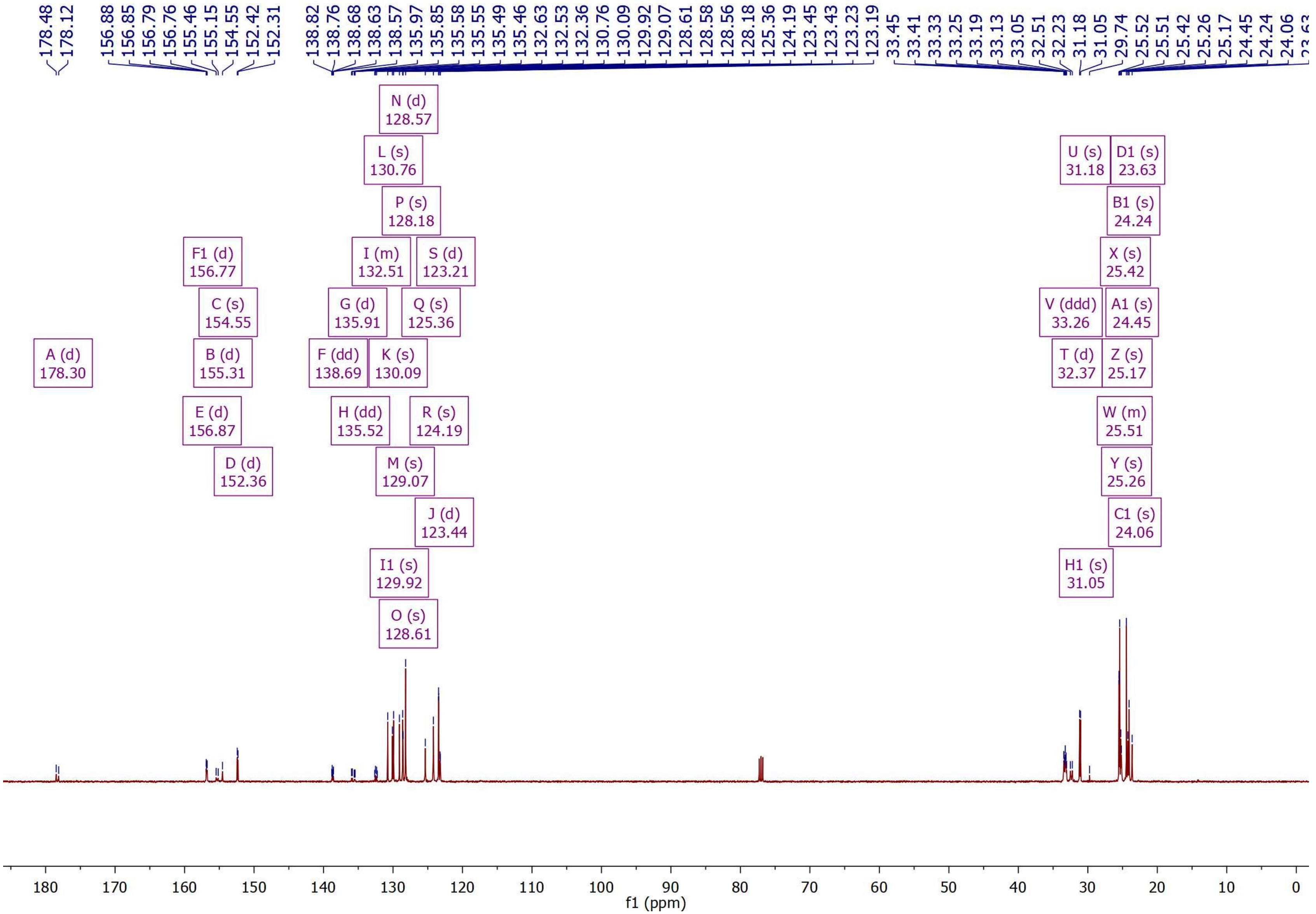

Figure S-63 $^{13}C\{^1H\}$ UDEFT NMR spectrum ($CDCl_3$) of compound 2_{Dipp} (R' = Ph)

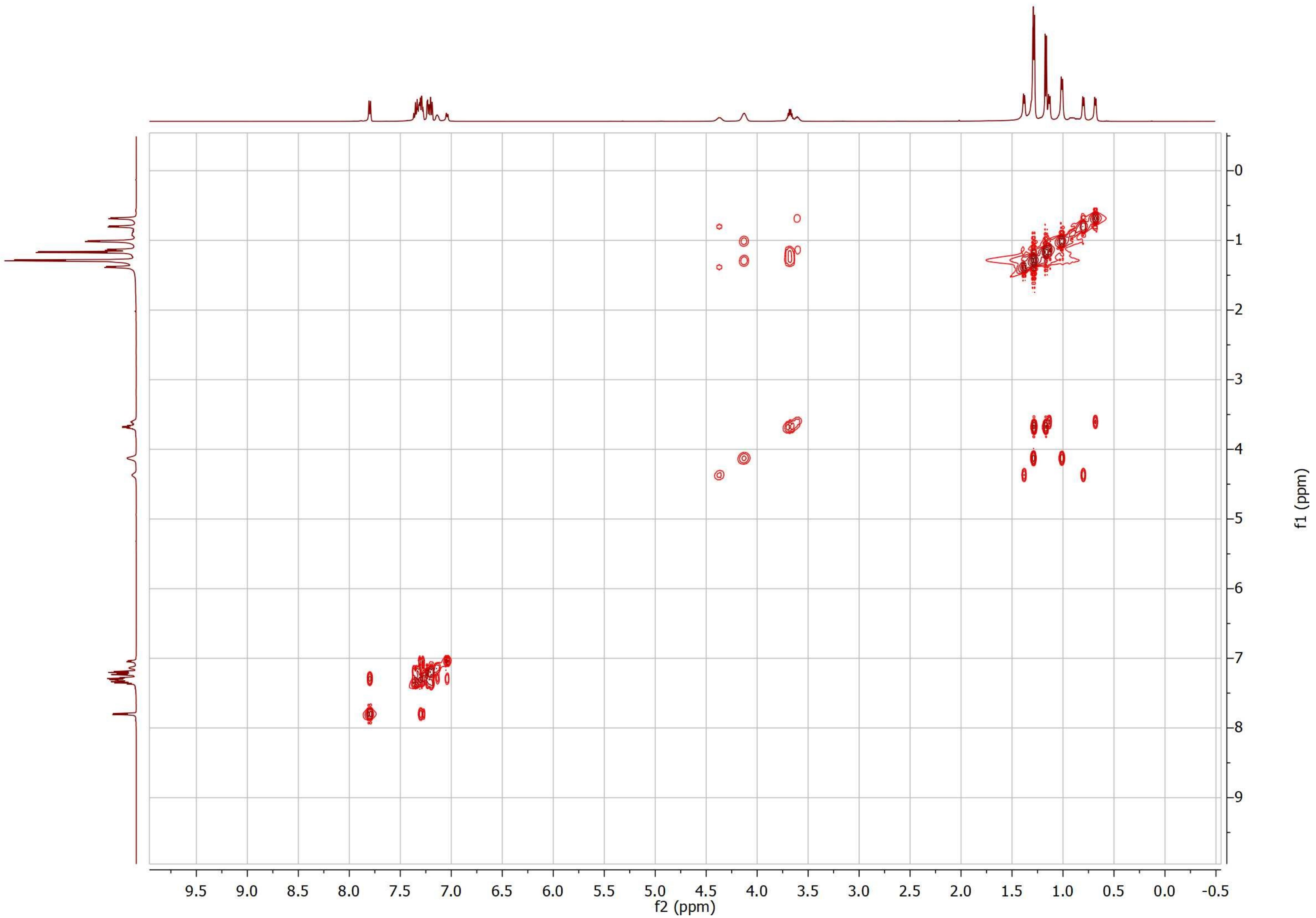

Figure S-64 ^{1}H COSY NMR spectrum ($CDCl_3$) of compound 2_{Dipp} (R' = Ph)

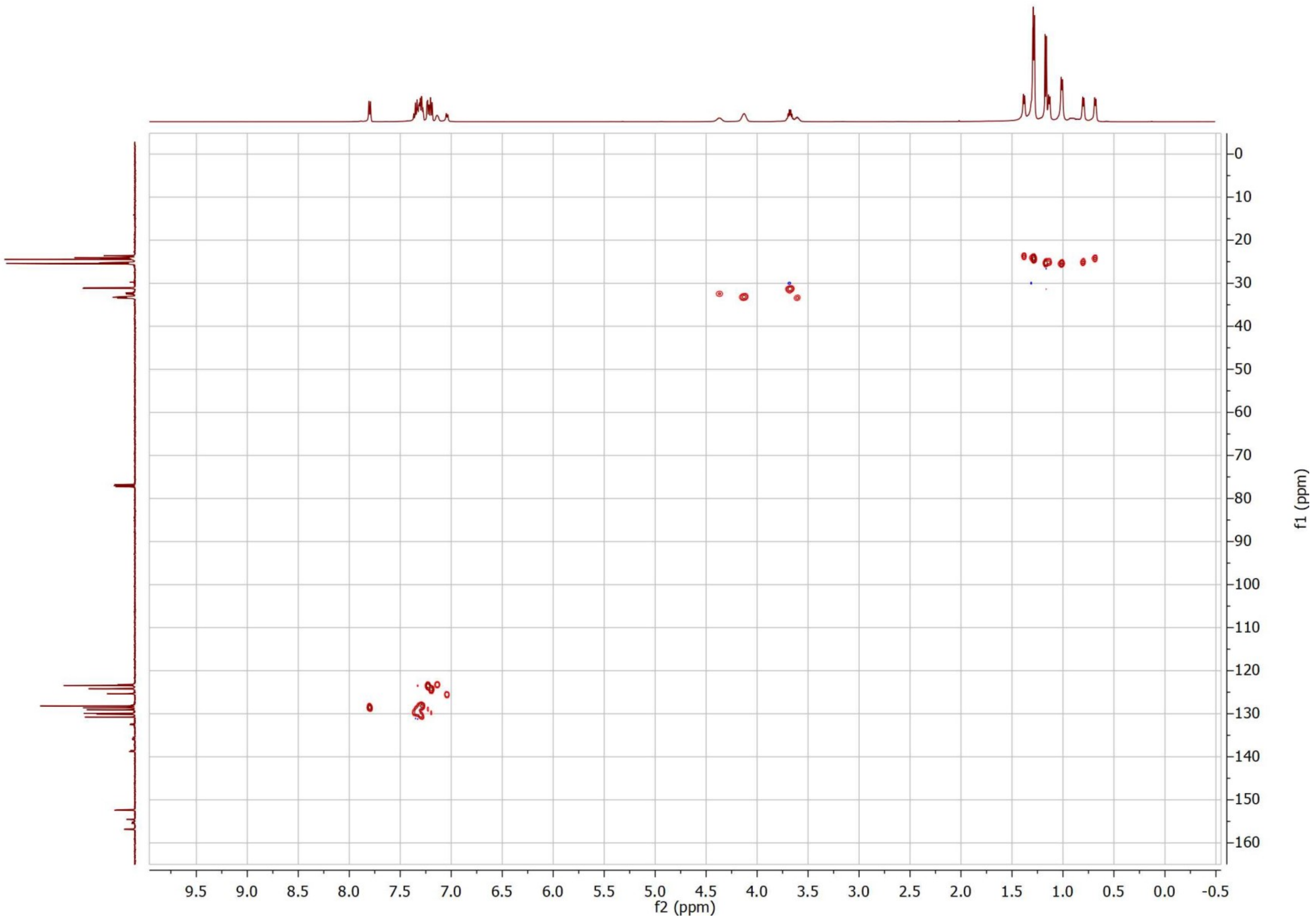

Figure S-65 HSQC NMR spectrum ($CDCl_3$) of compound 2_{Dipp} (R' = Ph)

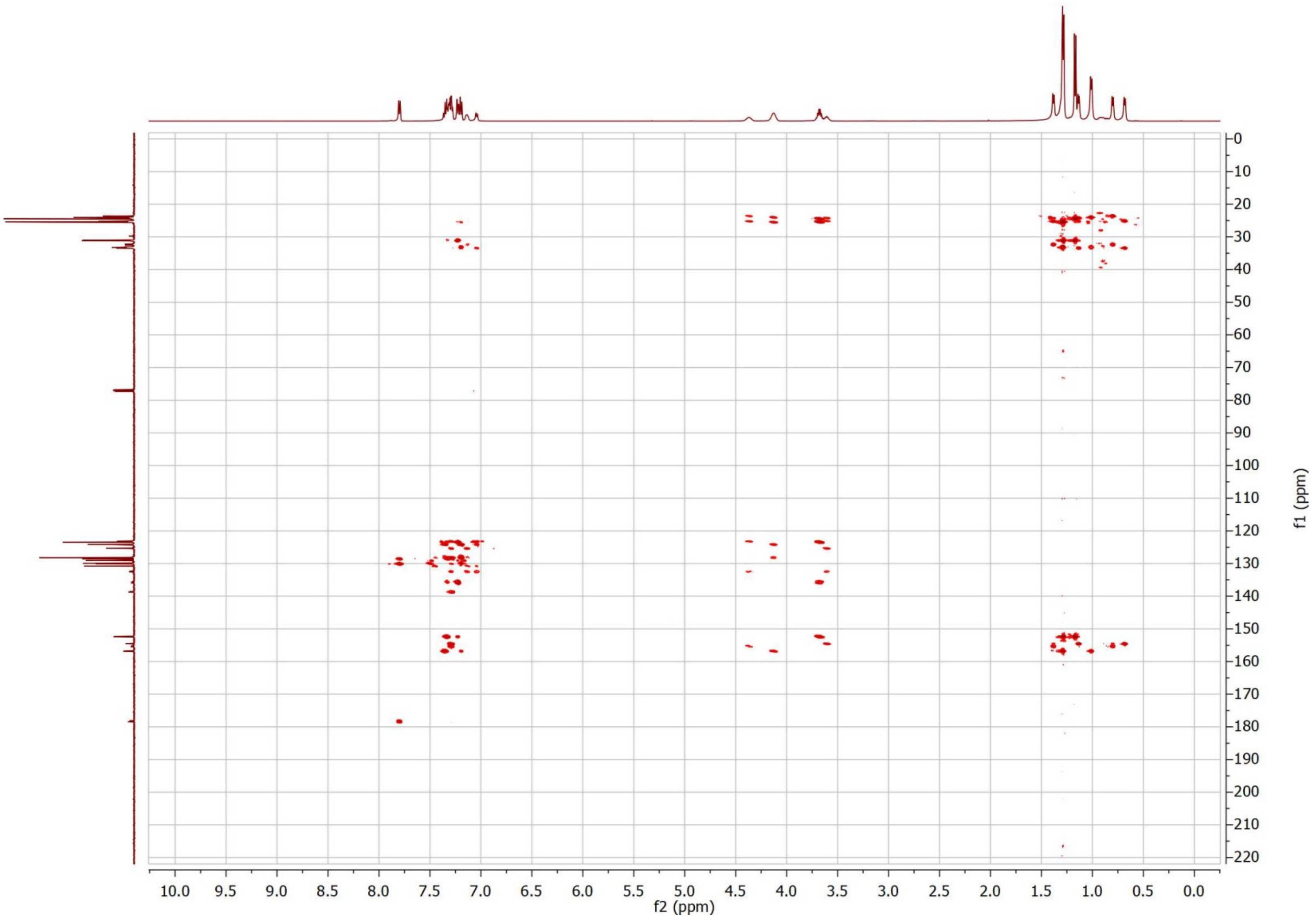

Figure S-66 HMBC NMR spectrum ($CDCl_3$) of compound 2_{Dipp} (R' = Ph)

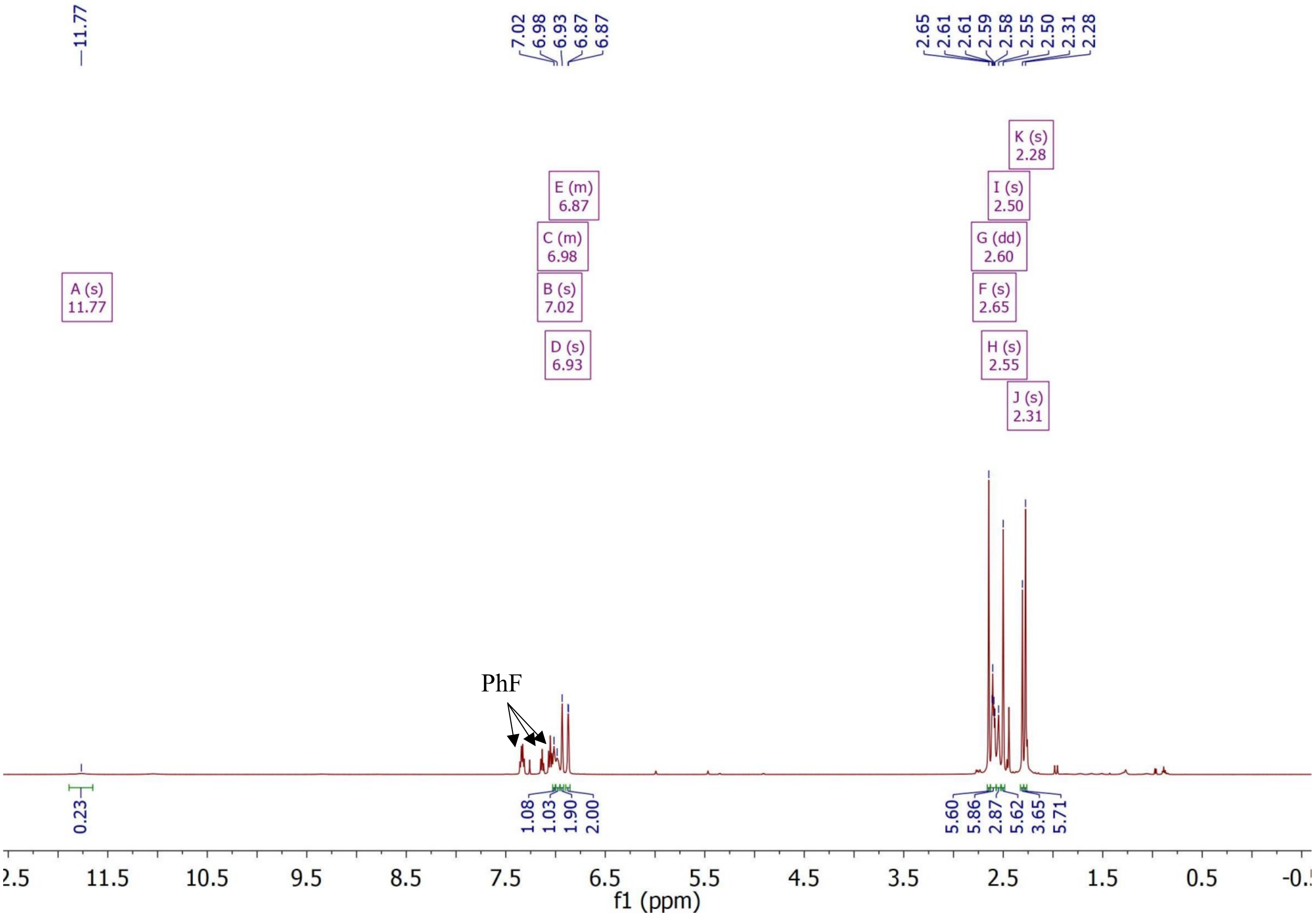

Figure S-67 ^{1}H NMR spectrum ($CDCl_3$) of compound $[1_{Mes}]^+$ (R' = Me)

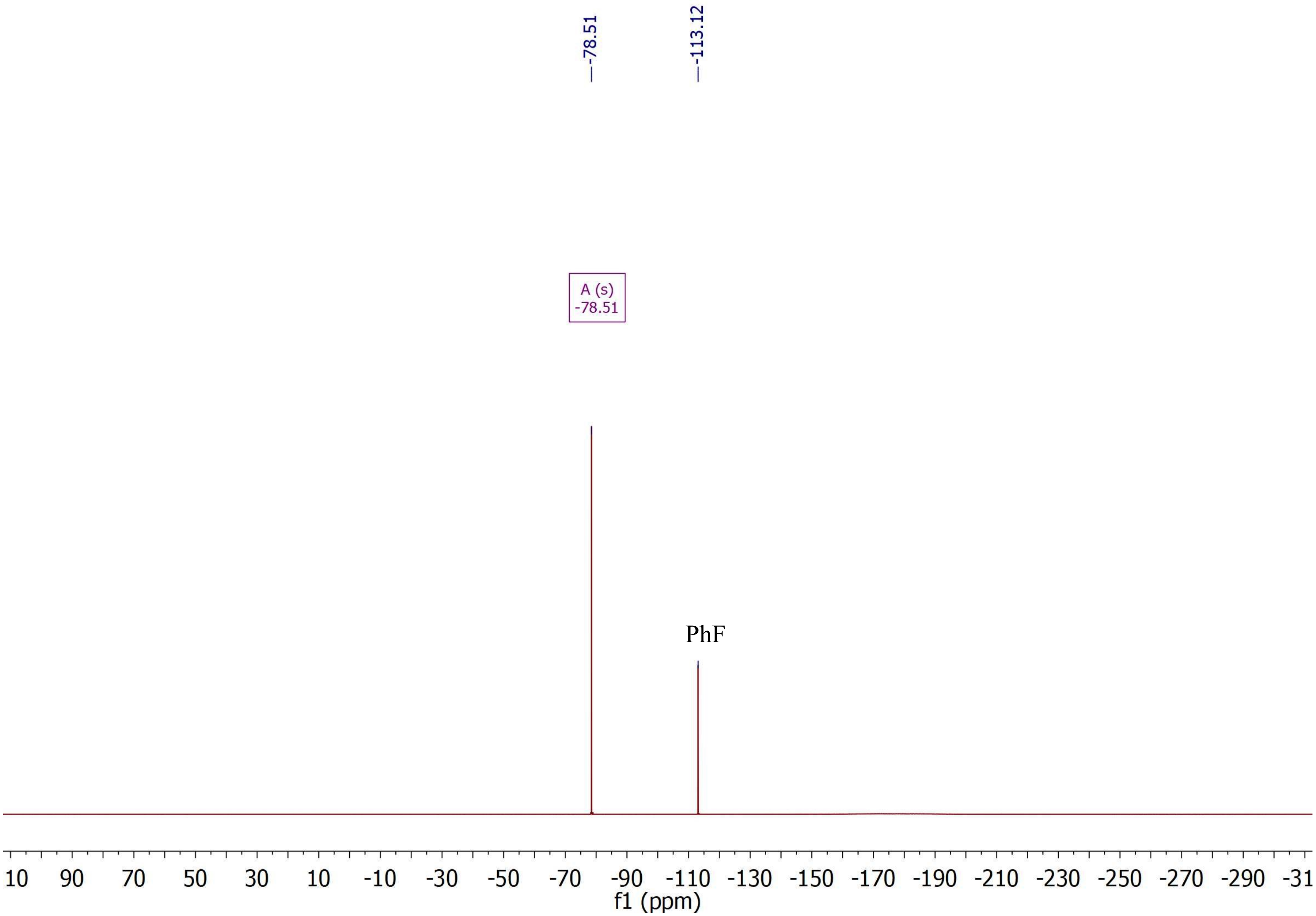

Figure S-68 ^{19}F NMR spectrum (CDCl$_3$) of compound [1_{Mes}]$^+$ (R' = Me)

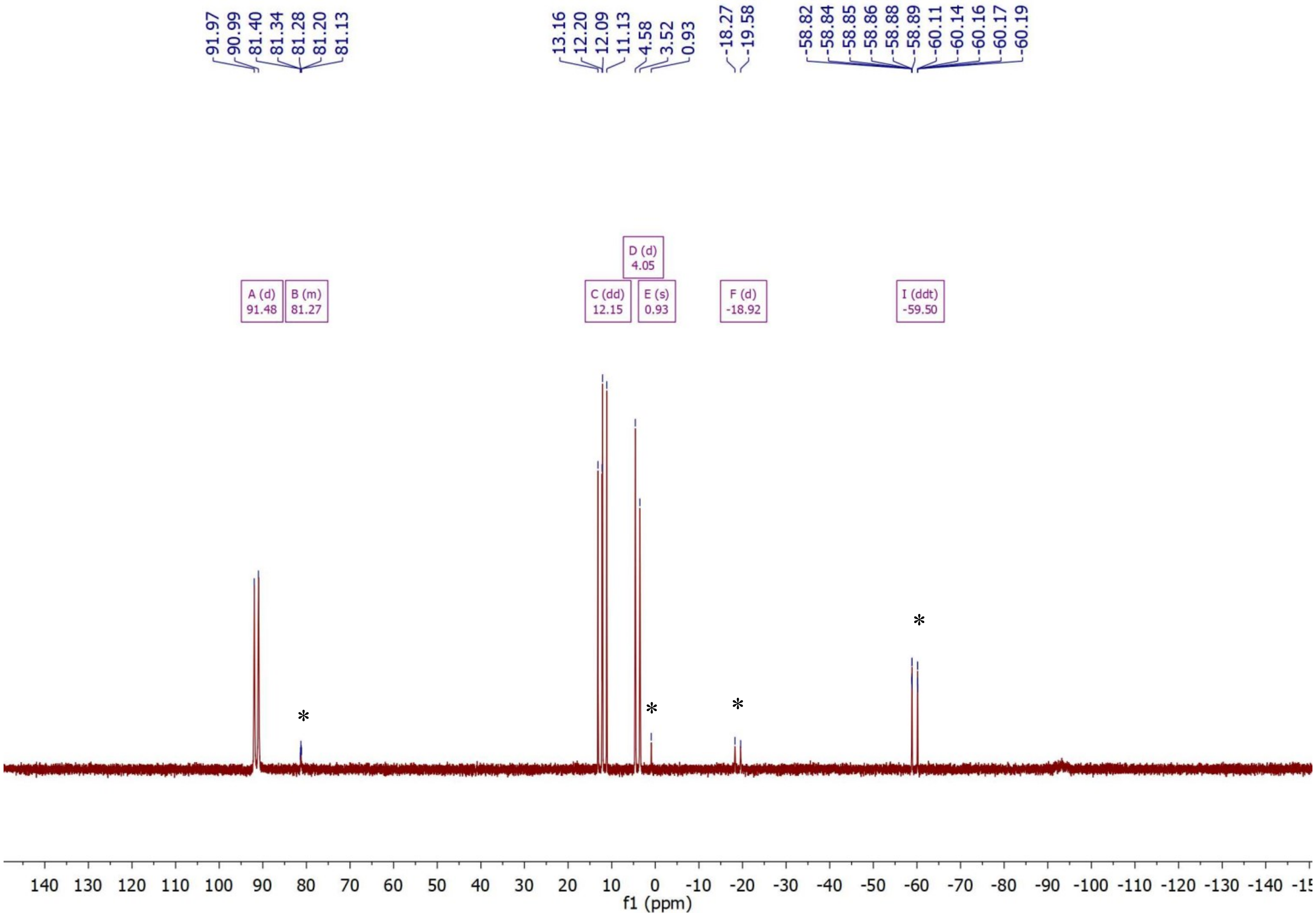

Figure S-69 ^{31}P NMR spectrum (CDCl$_3$) of compound $[1_{Mes}]^+$ (R' = Me) (* = degradative impurities)

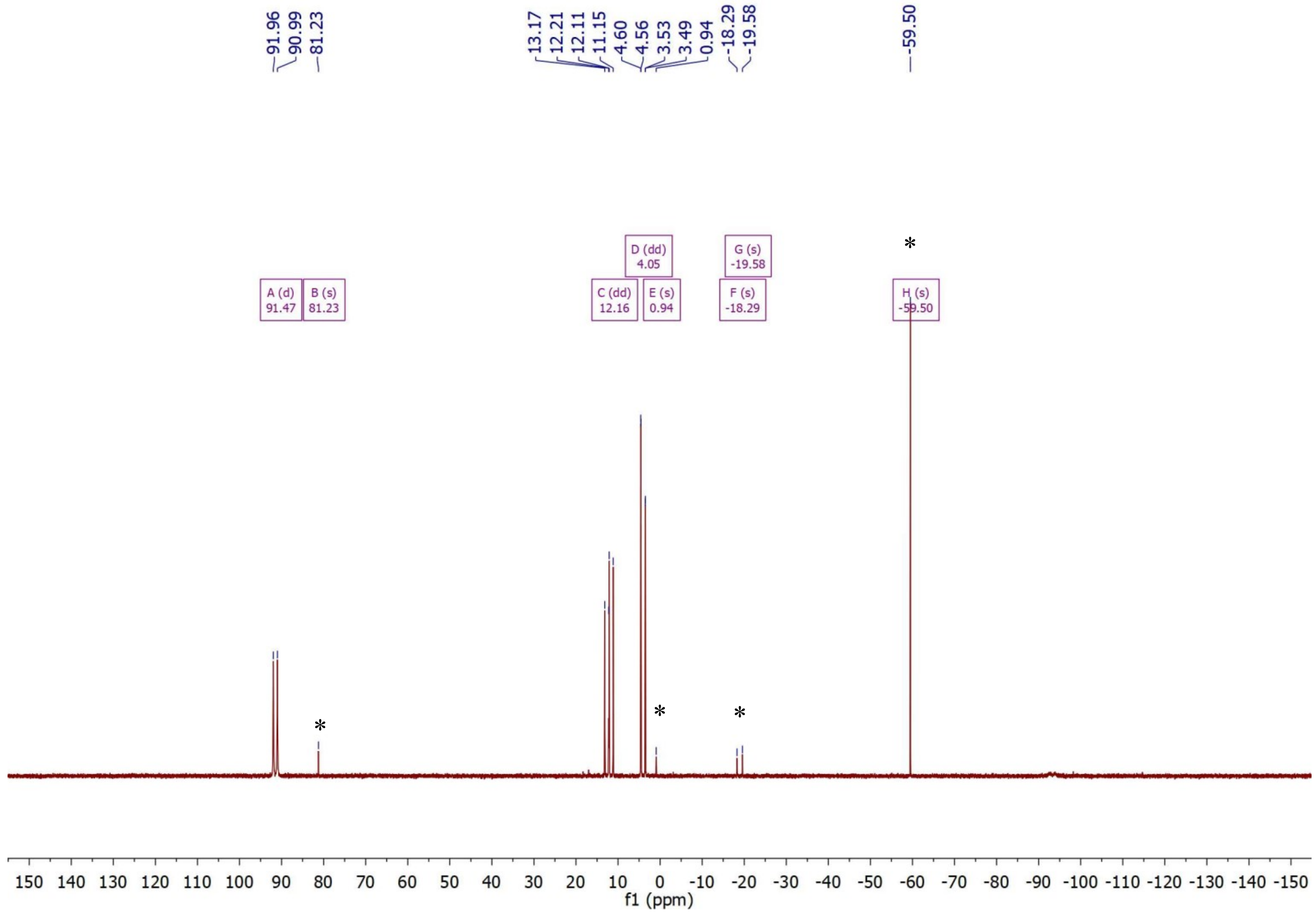

Figure S-70 $^{31}P\{^{1}H\}$ NMR spectrum ($CDCl_3$) of compound $[1_{Mes}]^+$ (R' = Me) (* = degradative impurities)

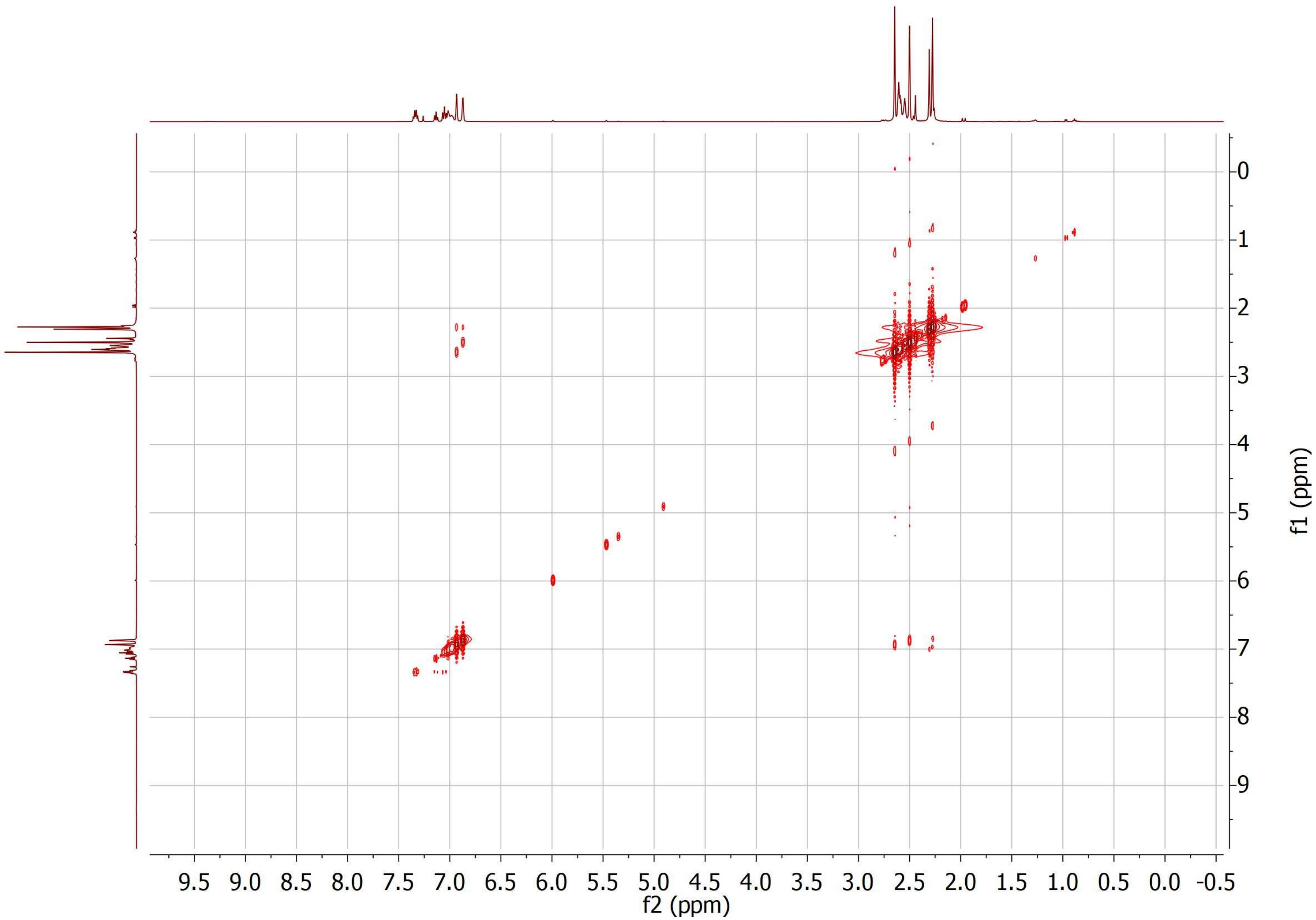

Figure S-71 ^{1}**H COSY NMR spectrum (CDCl**$_3$**) of [1**$_{Mes}$**]**$^{+}$ **(R' = Me)**

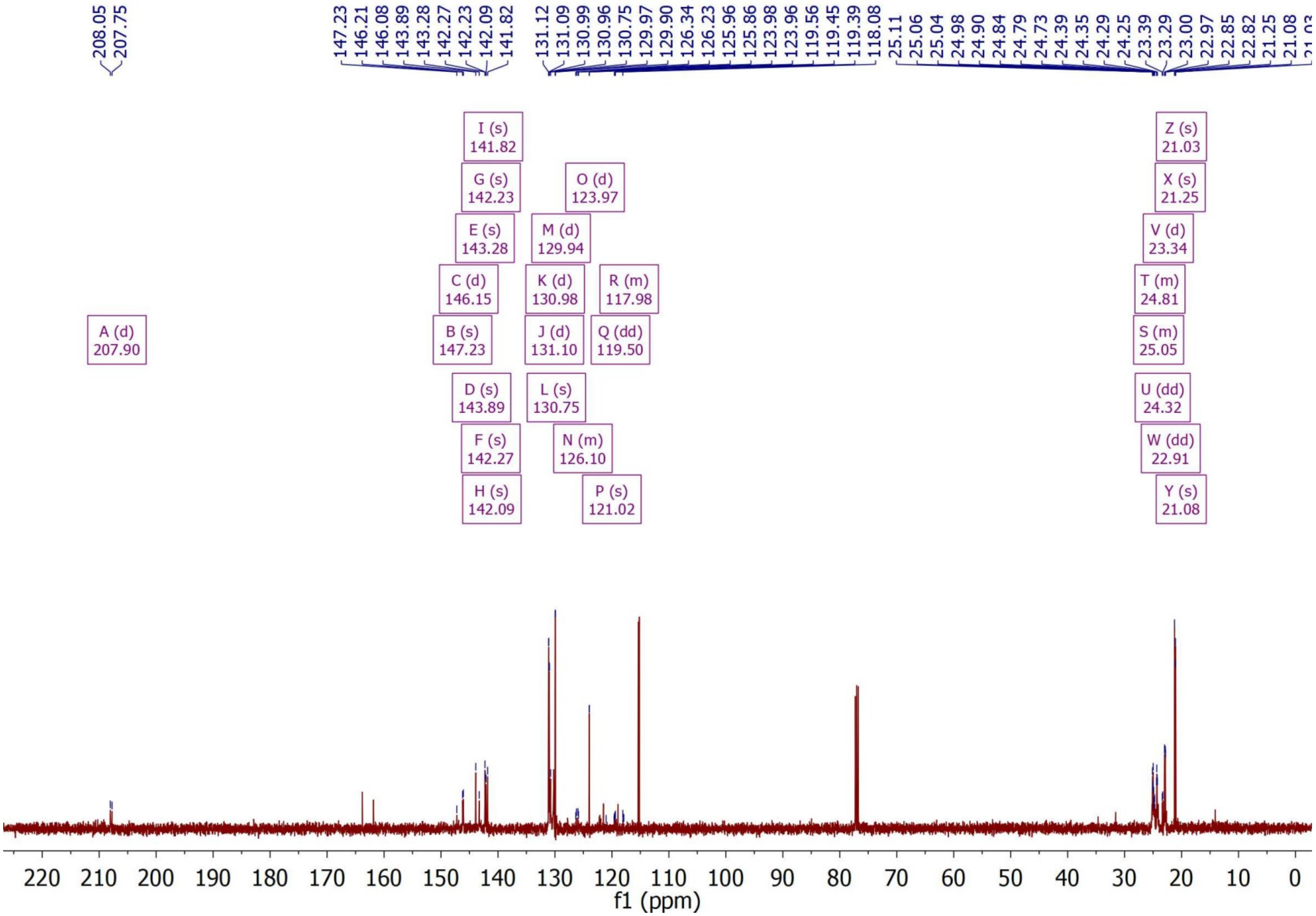

Figure S-72 $^{13}C\{^{1}H\}$ UDEFT NMR spectrum ($CDCl_3$) of compound $[1_{Mes}]^+$ (R' = Me)

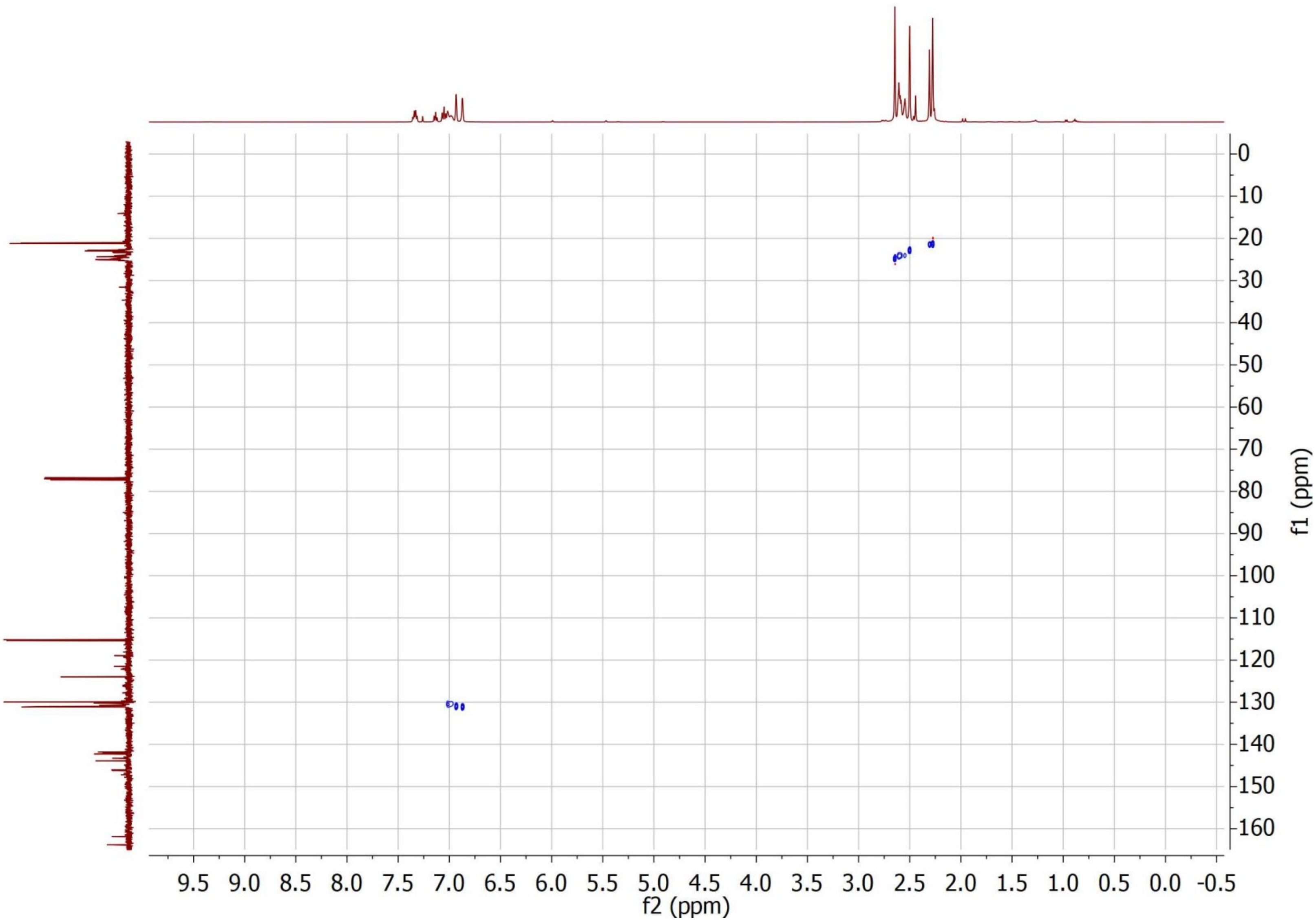

Figure S-73 HSQC NMR spectrum ($CDCl_3$) of compound $[1_{Mes}]^+$ (R' = Me)

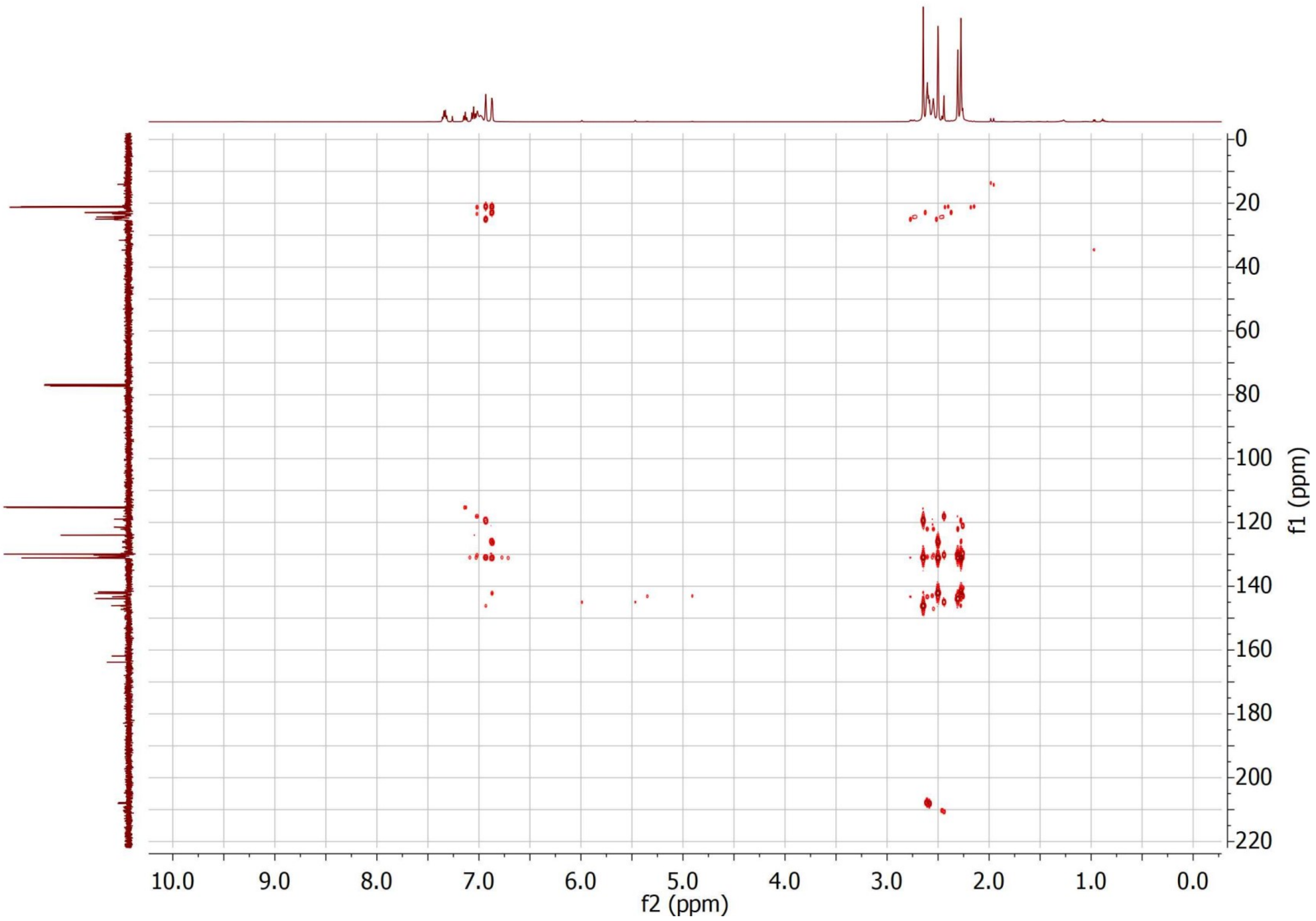

Figure S-74 HMBC NMR spectrum ($CDCl_3$) of compound $[1_{Mes}]^+$ (R' = Me)

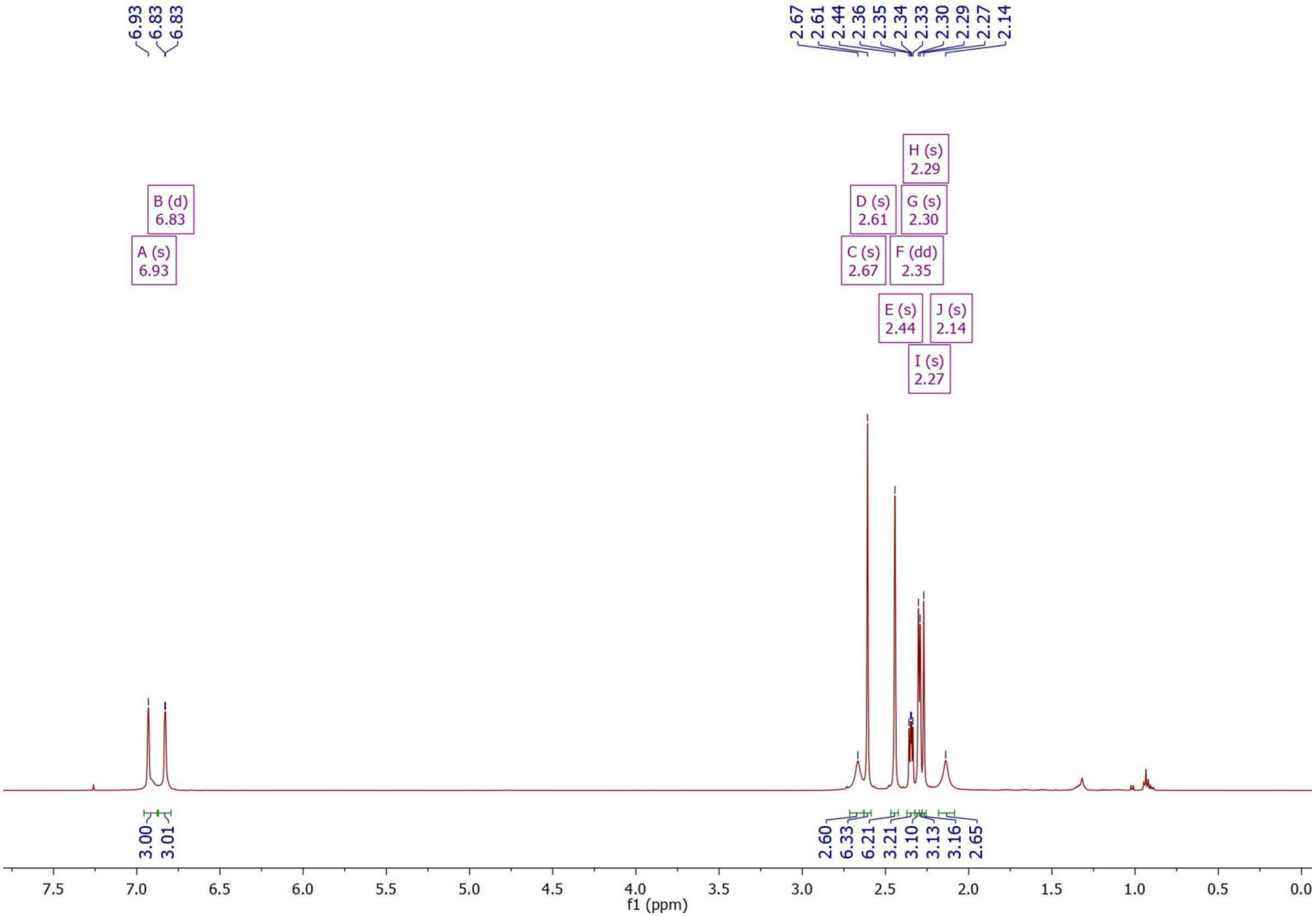

Figure S-75 ^{1}H NMR spectrum ($CDCl_3$) of compound 2_{Mes} (R' = Me)

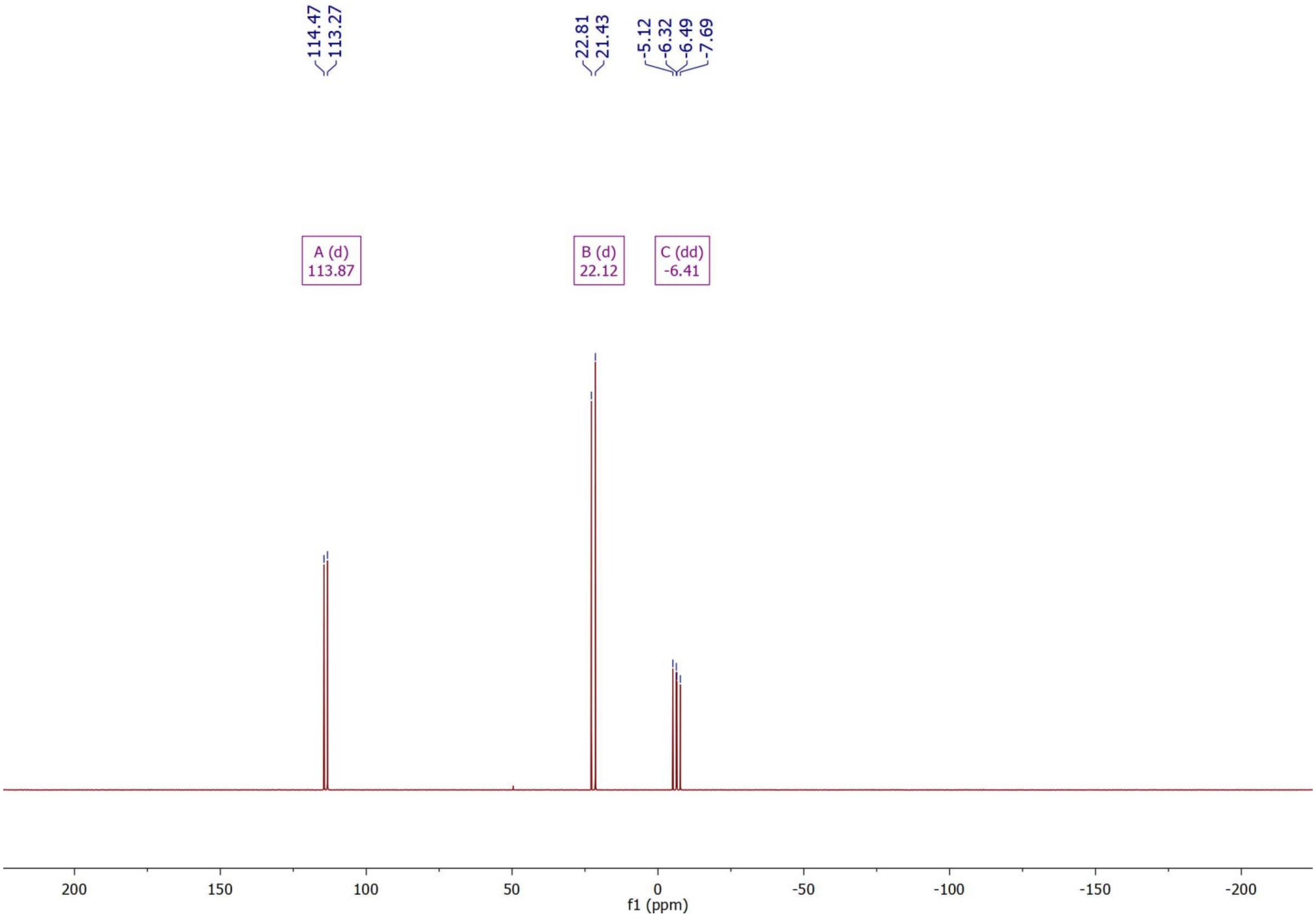

Figure S-76 $^{31}P\{^{1}H\}$ NMR spectrum ($CDCl_3$) of compound 2_{Mes} (R' = Me)

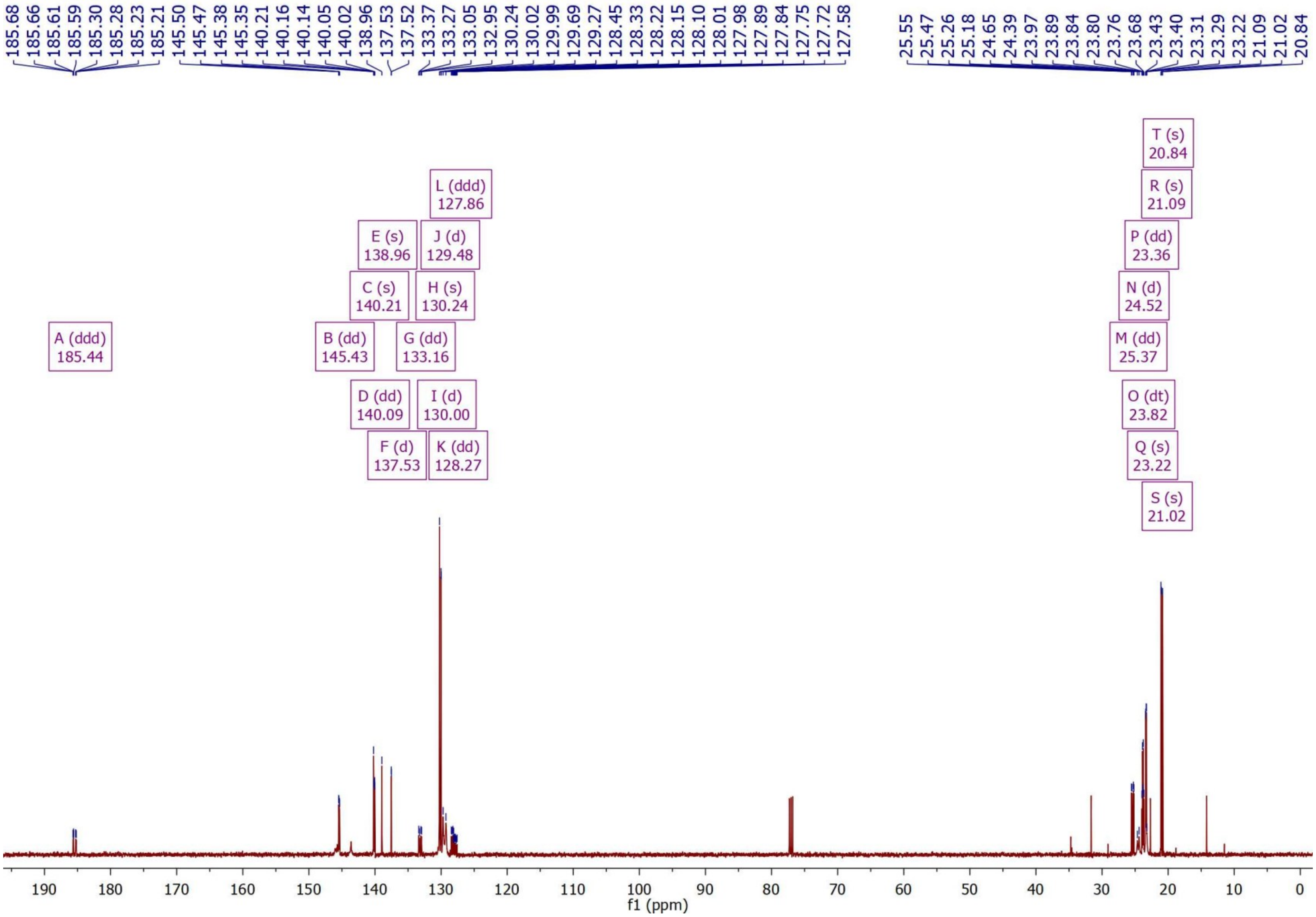

Figure S-77 $^{13}C\{^{1}H\}$ NMR spectrum ($CDCl_3$) of compound 2_{Mes} (R' = Me)

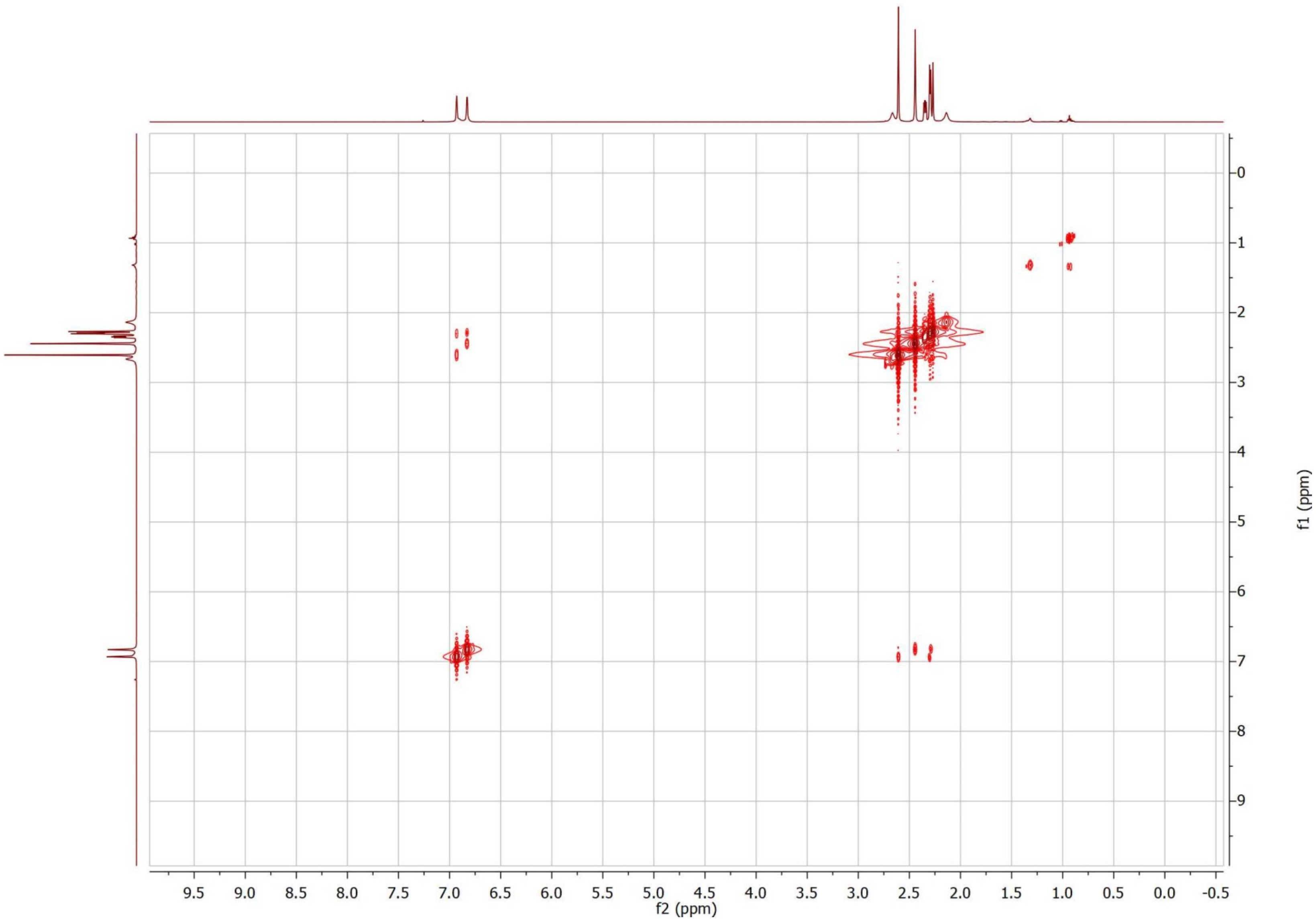

Figure S-78 ^{1}H COSY NMR spectrum (CDCl$_3$) of compound 2$_{Mes}$ (R’ = Me)

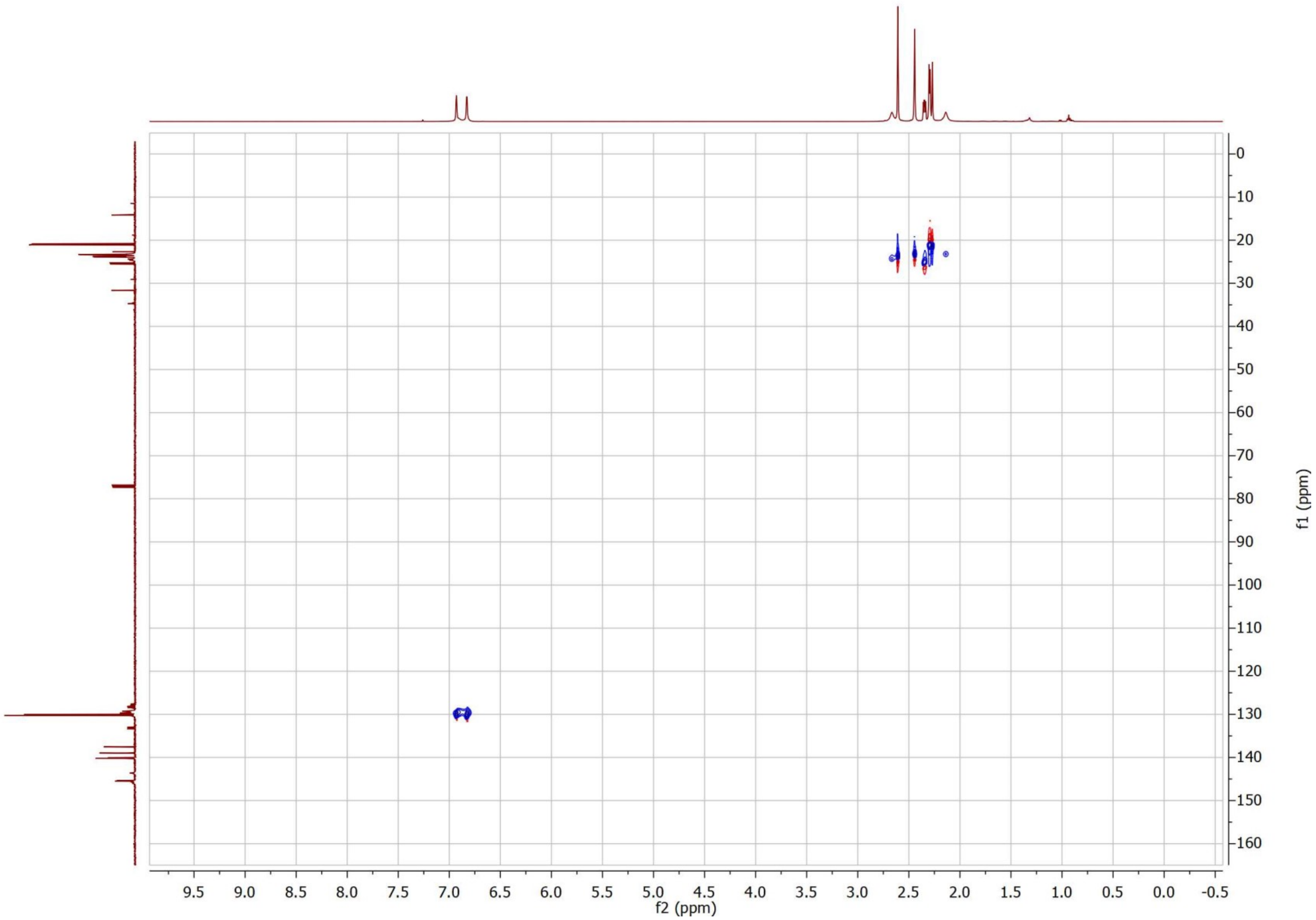

Figure S-79 HSQC NMR spectrum ($CDCl_3$) of compound 2_{Mes} (R' = Me)

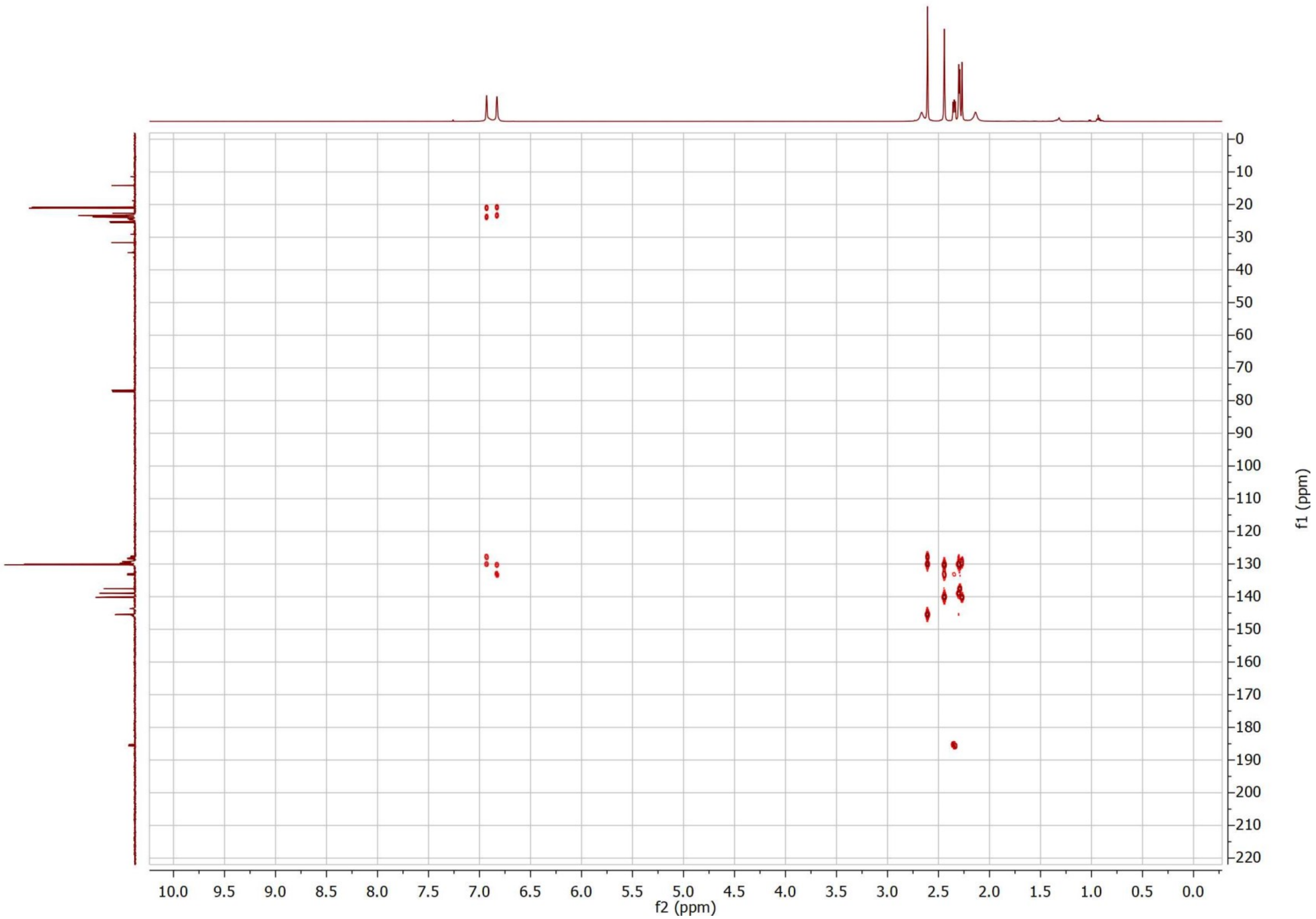

Figure S-80 HMBC NMR spectrum ($CDCl_3$) of compound 2_{Mes} (R' = Me)

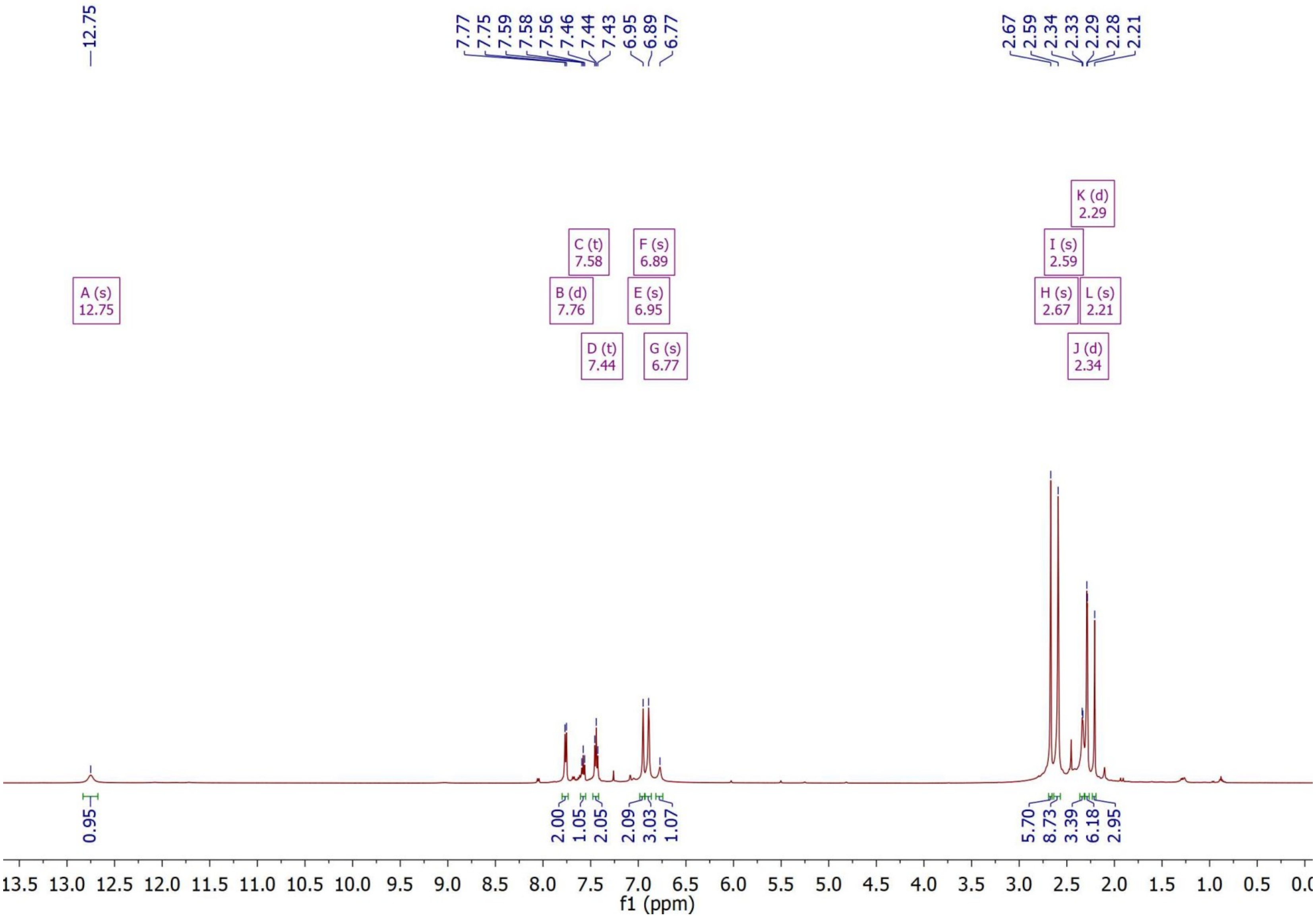

Figure S-81 ^{1}H NMR spectrum ($CDCl_3$) of compound $[1_{Mes}]^+$ (R' = Ph)

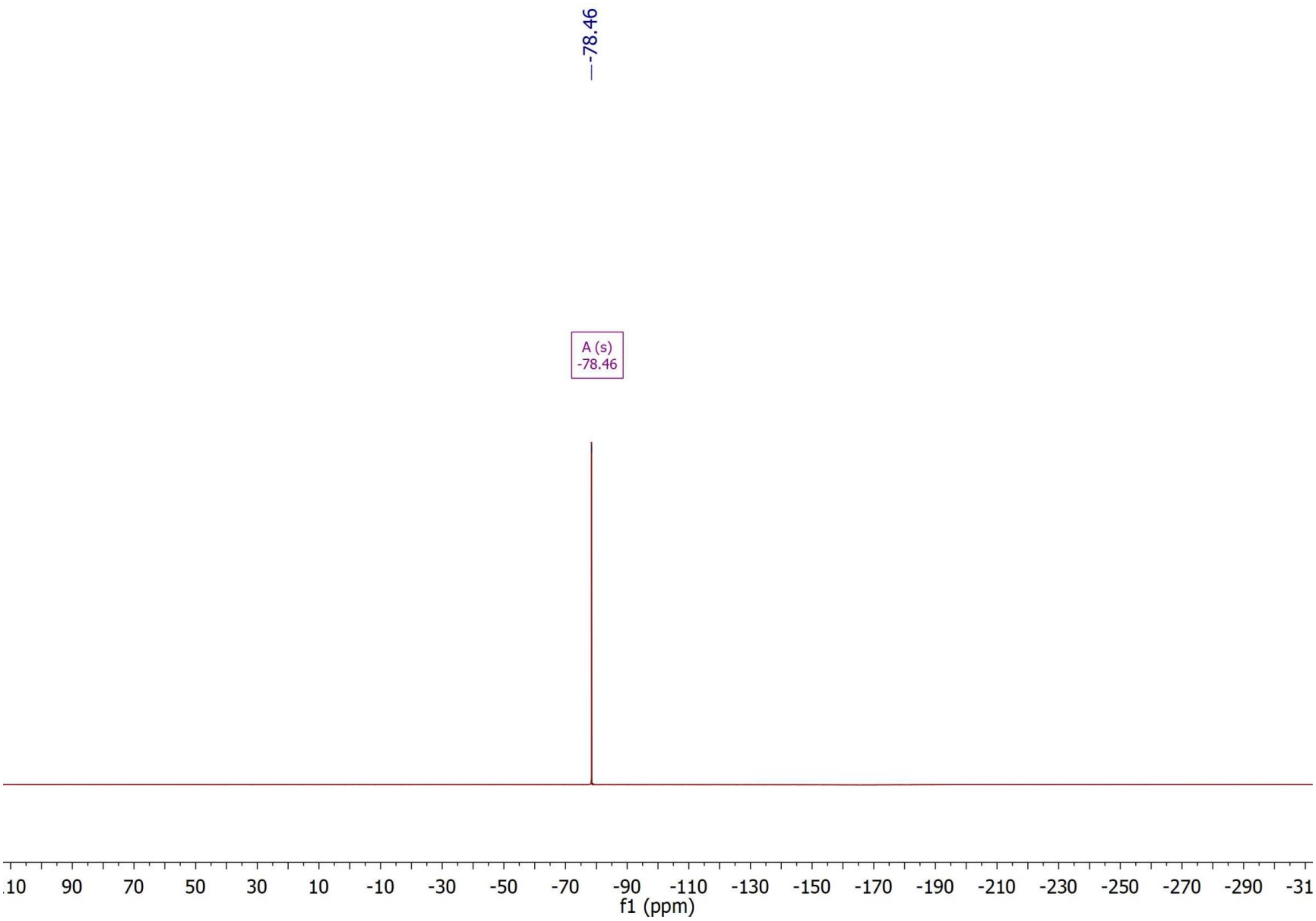

Figure S-82 ^{19}F NMR spectrum (CDCl$_3$) of compound [1$_{Mes}$]$^+$ (R' = Ph)

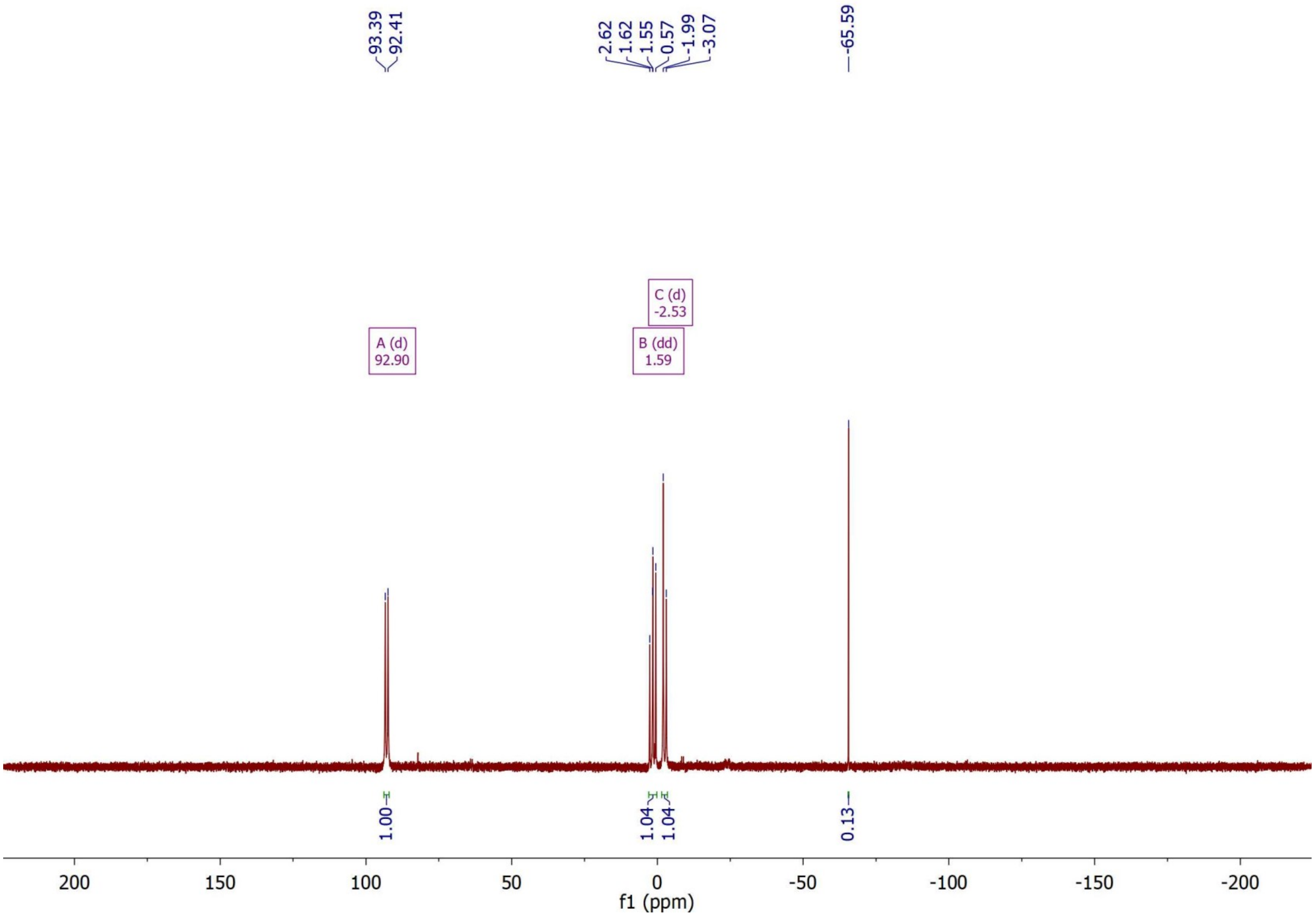

Figure S-83 $^{31}P\{^{1}H\}$ NMR spectrum ($CDCl_3$) of compound $[1_{Mes}]^+$ (R' = Ph)

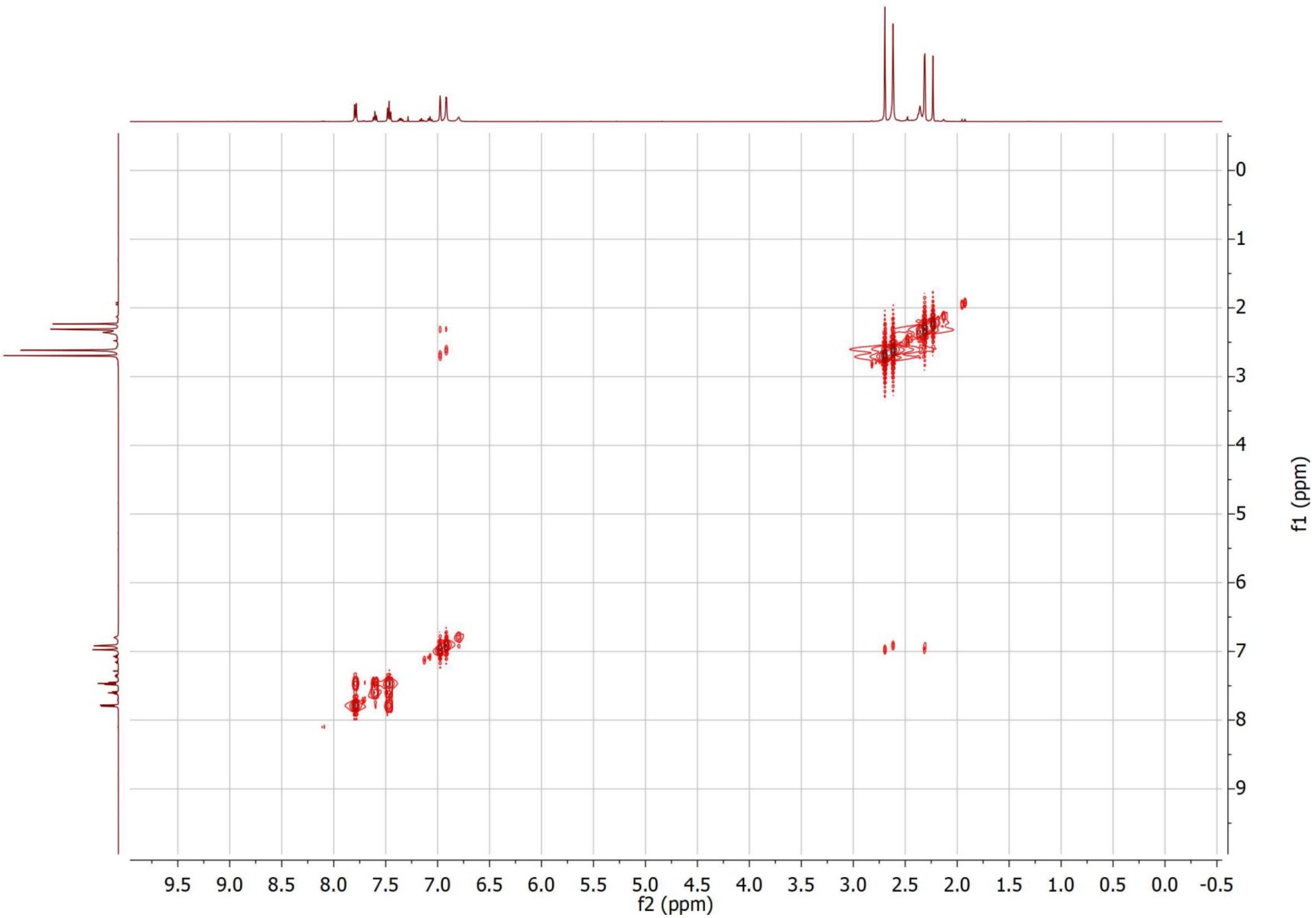

Figure S-84 ^{1}H COSY NMR spectrum ($CDCl_3$) of compound $[1_{Mes}]^{=}$ (R' = Ph)

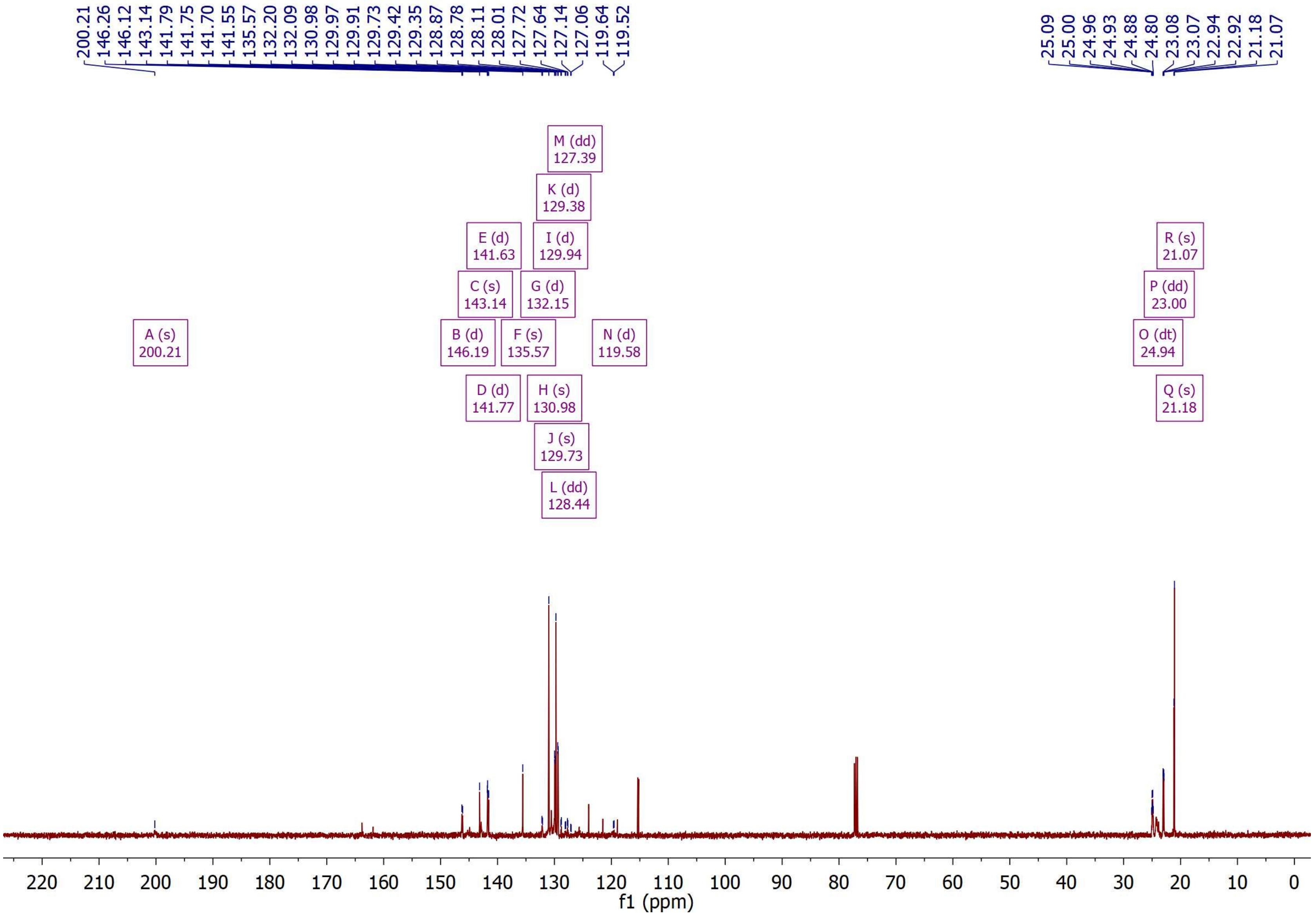

Figure S-85 $^{13}C\{^{1}H\}$ UDEFT NMR spectrum ($CDCl_3$) of compound $[1_{Mes}]^+$ (R' = Ph)

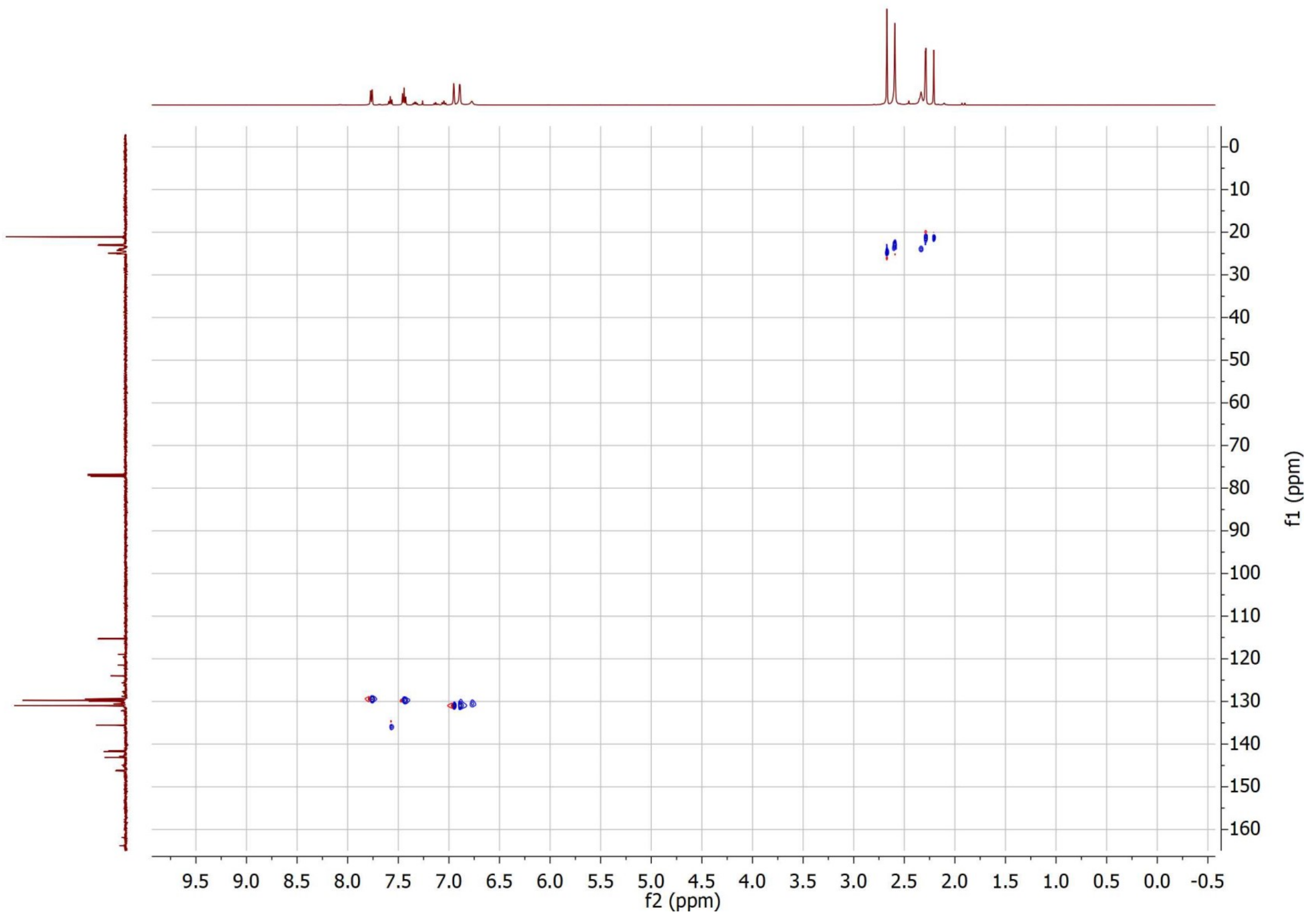

Figure S-86 HSQC NMR spectrum ($CDCl_3$) of compound $[1_{Mes}]^+$ (R' = Ph)

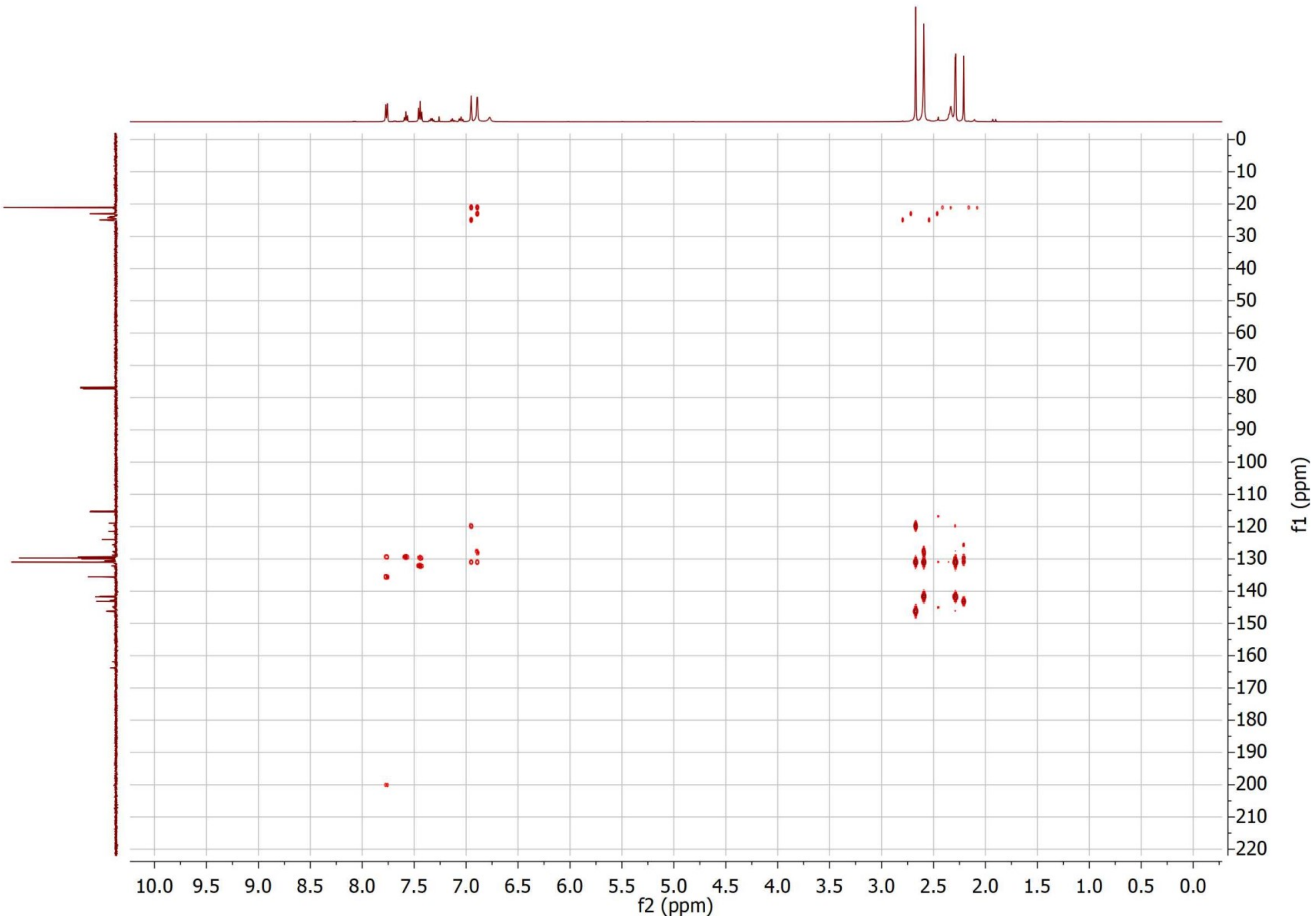

Figure S-87 HMBC NMR spectrum ($CDCl_3$) of compound $[1_{Mes}]^+$ (R' = Ph)

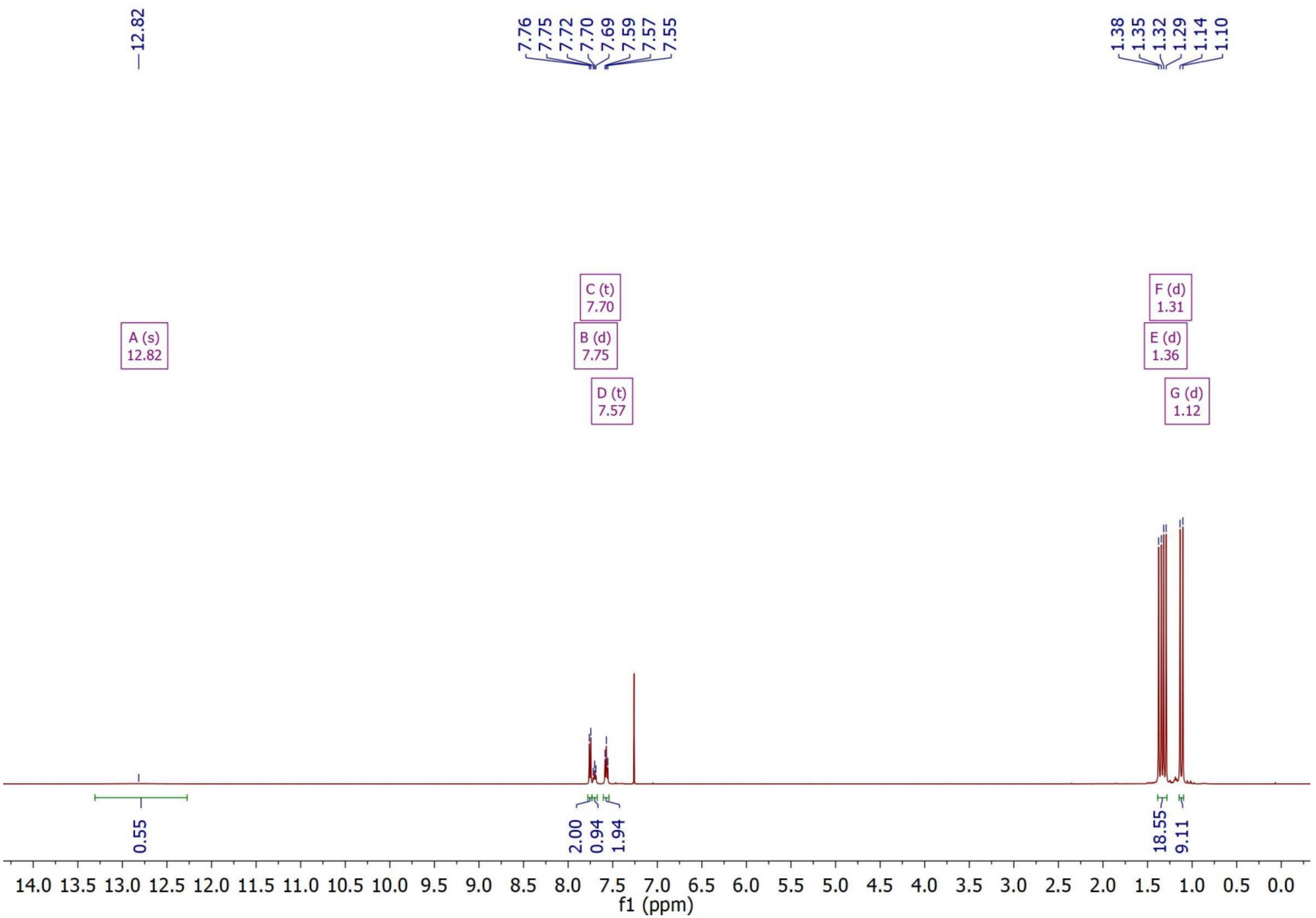

Figure S-88 ^{1}H NMR spectrum ($CDCl_3$) of compound $[1_{tBu}]^+$ (R' = Ph)

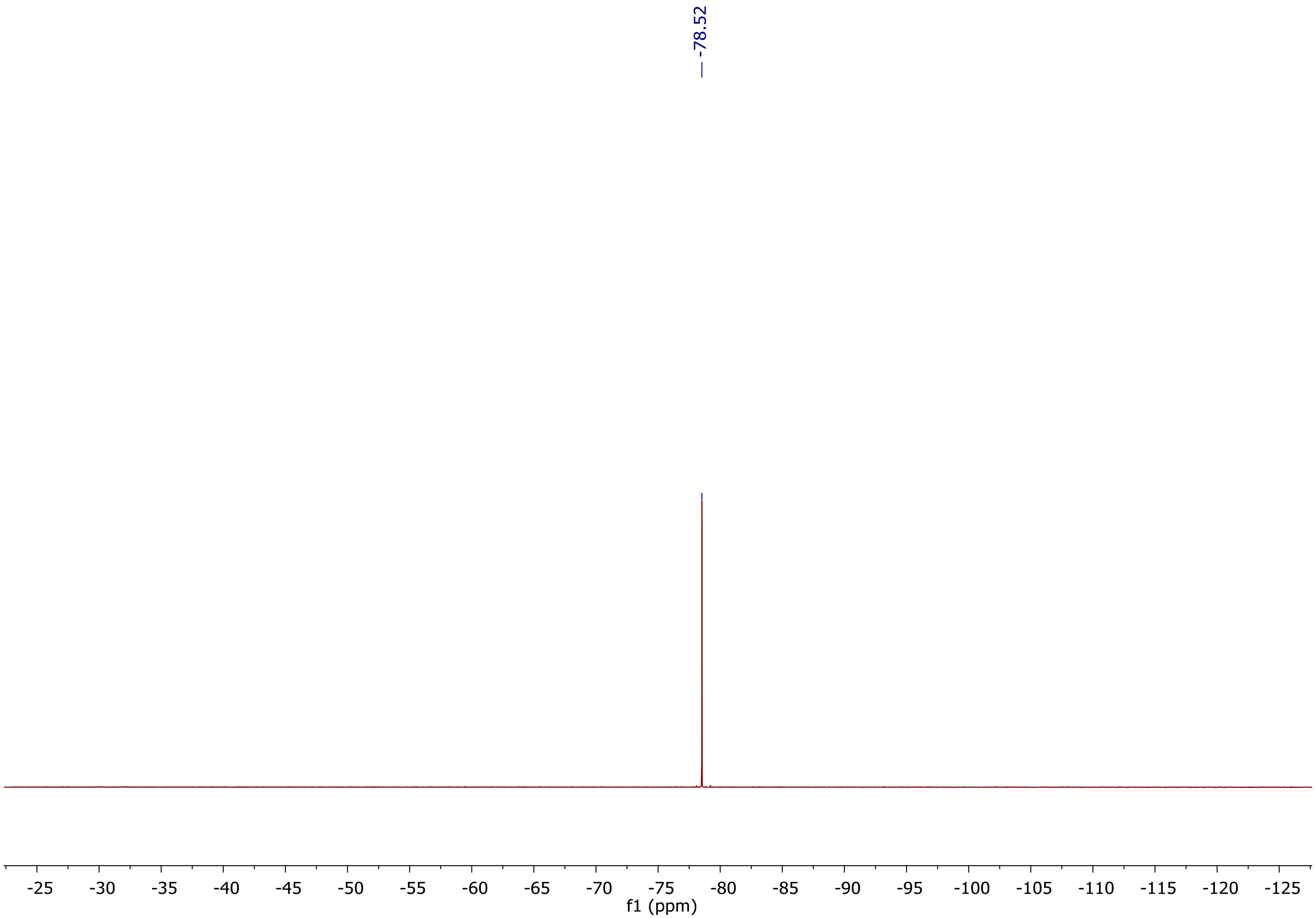

Figure S-89 ^{19}F NMR spectrum ($CDCl_3$) of compound $[1_{tBu}]^+$ (R' = Ph)

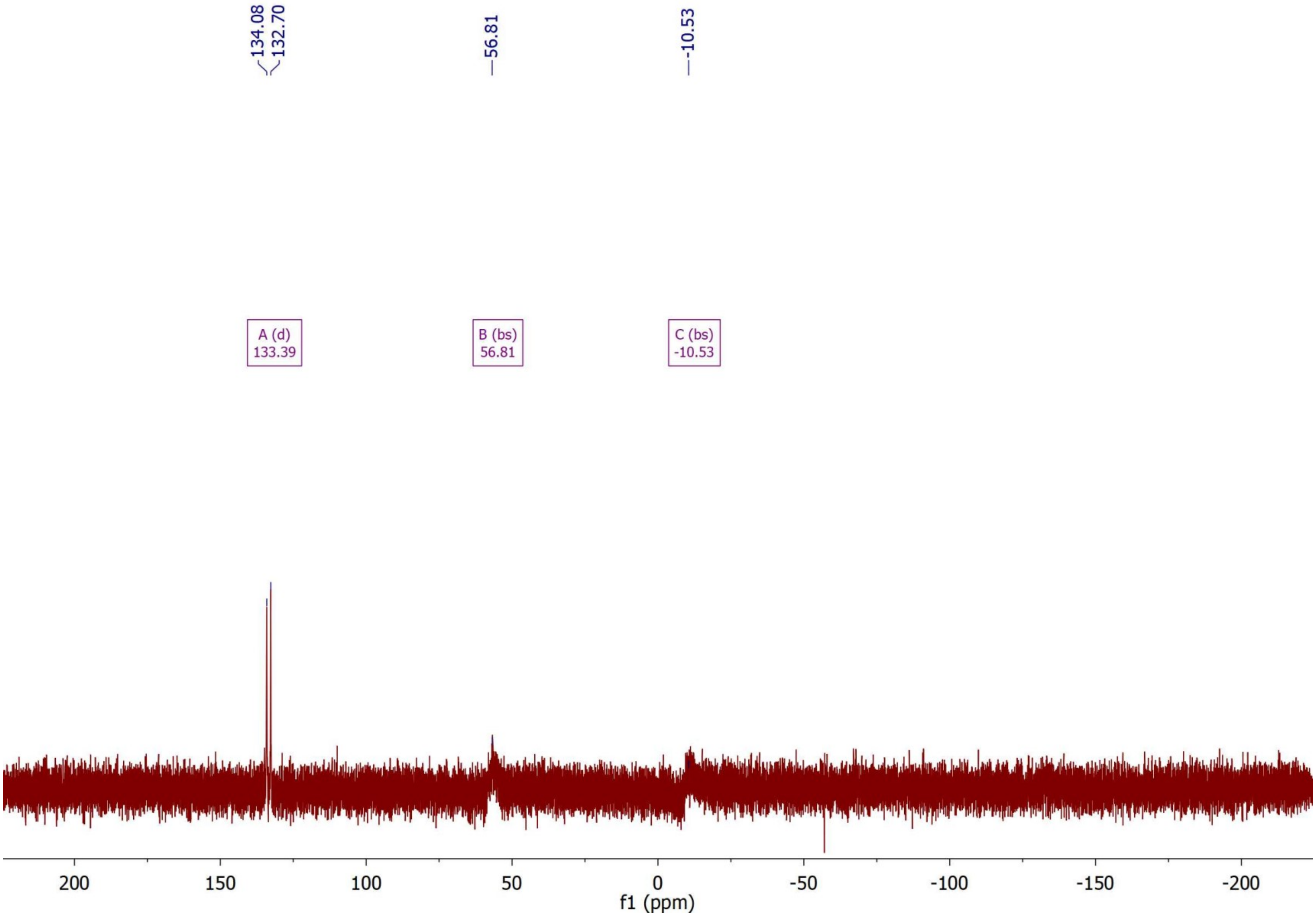

Figure S-90 $^{31}P\{^{1}H\}$ NMR spectrum ($CDCl_3$) of compound $[1_{tBu}]^+$ (R' = Ph)

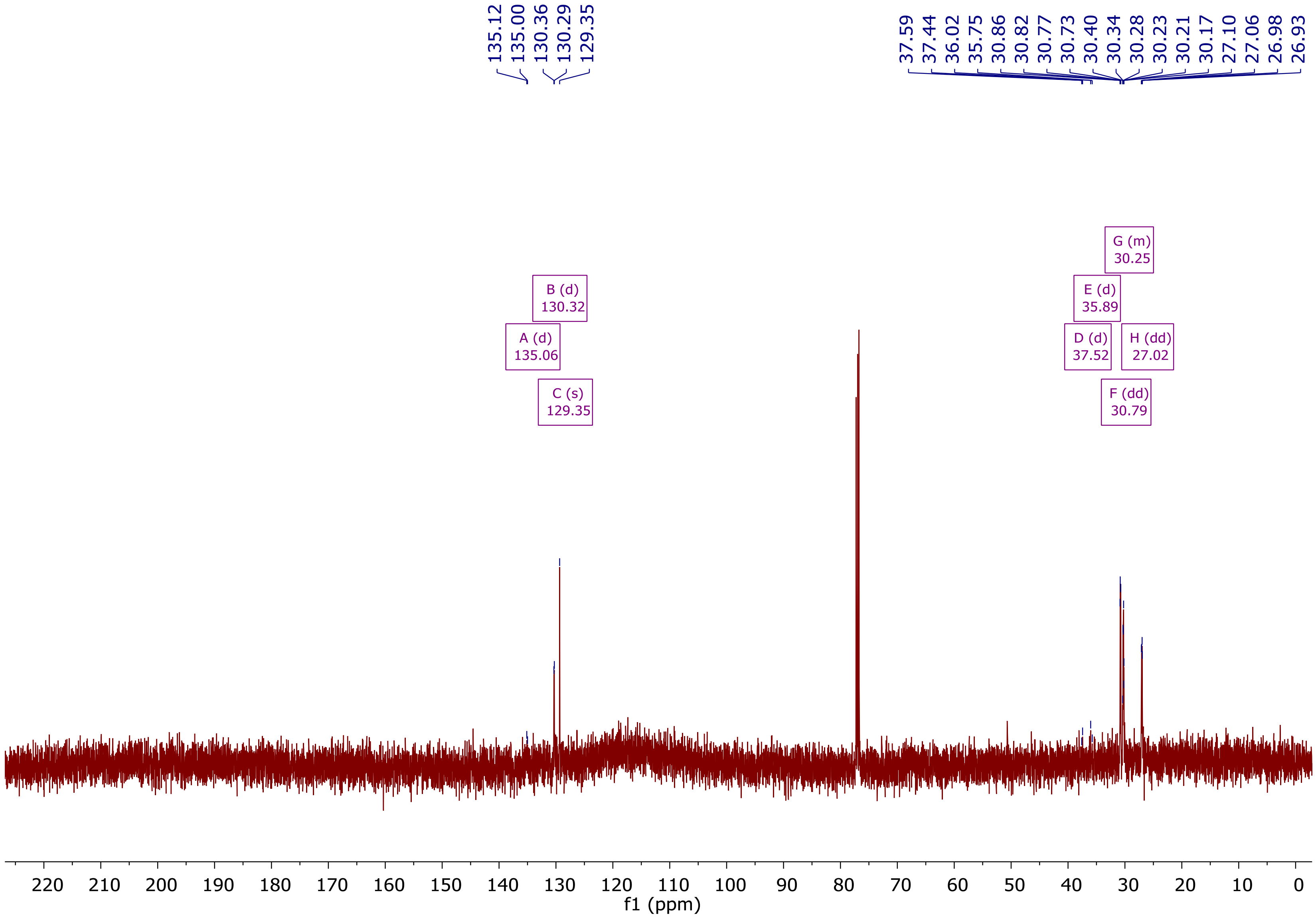

Figure S-91 $^{13}C\{^1H\}$ UDEFT NMR spectrum ($CDCl_3$) of compound $[1_{tBu}]^+$ (R' = Ph)

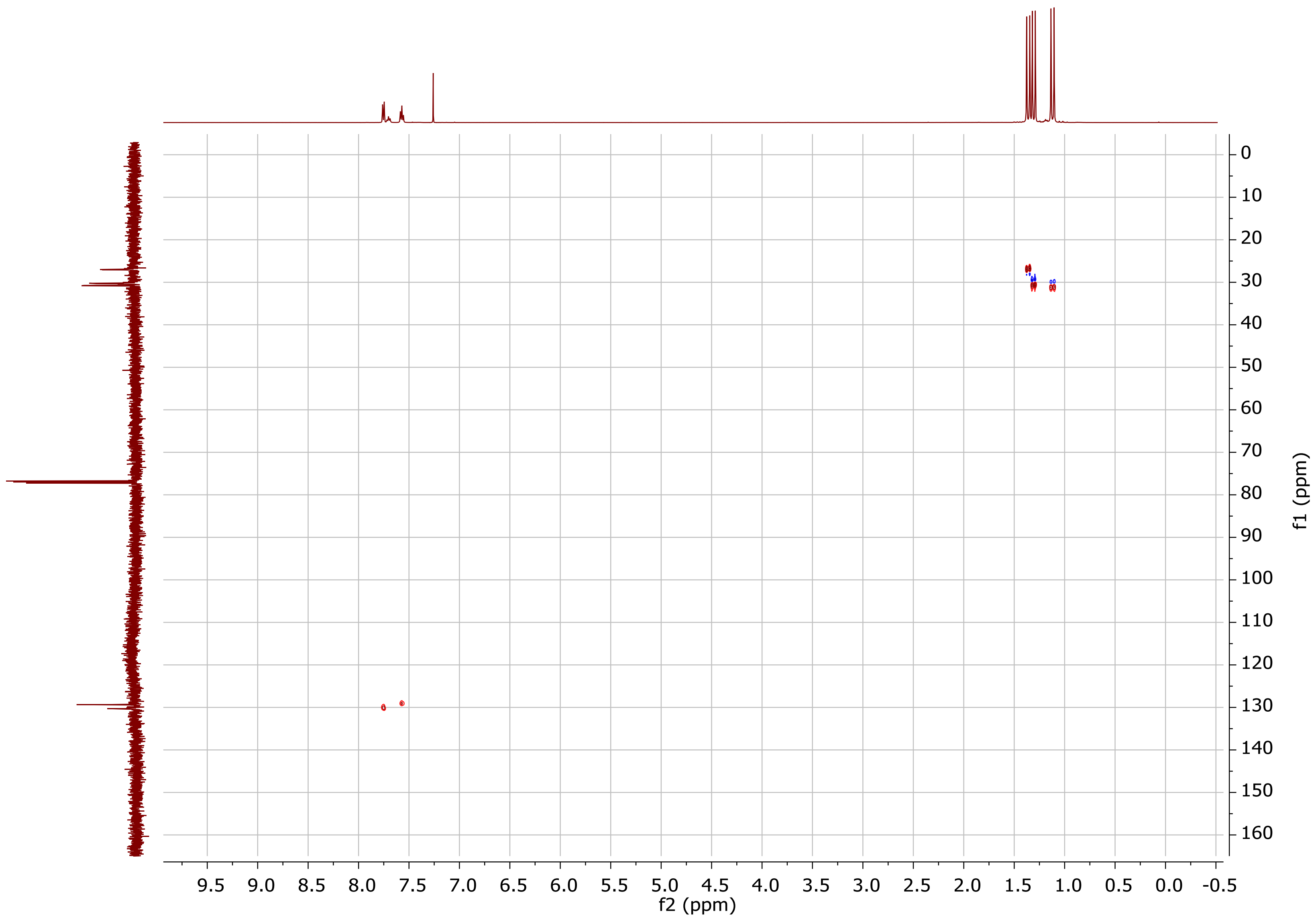

Figure S-92 HSQC NMR spectrum ($CDCl_3$) of compound $[1_{tBu}]^+$ (R' = Ph)

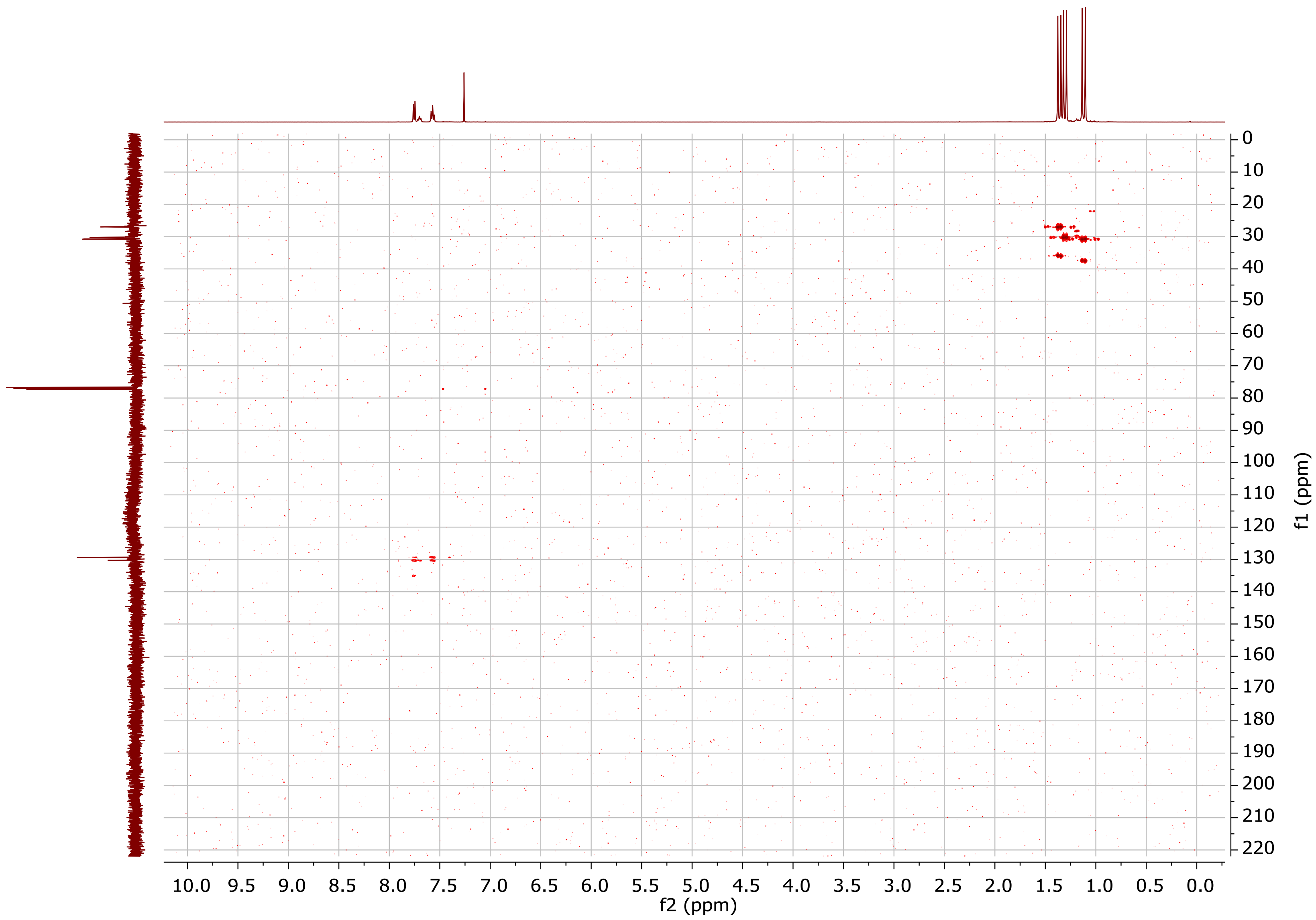

Figure S-93 HMBC NMR spectrum ($CDCl_3$) of compound $[1_{tBu}]^+$ (R’ = Ph)

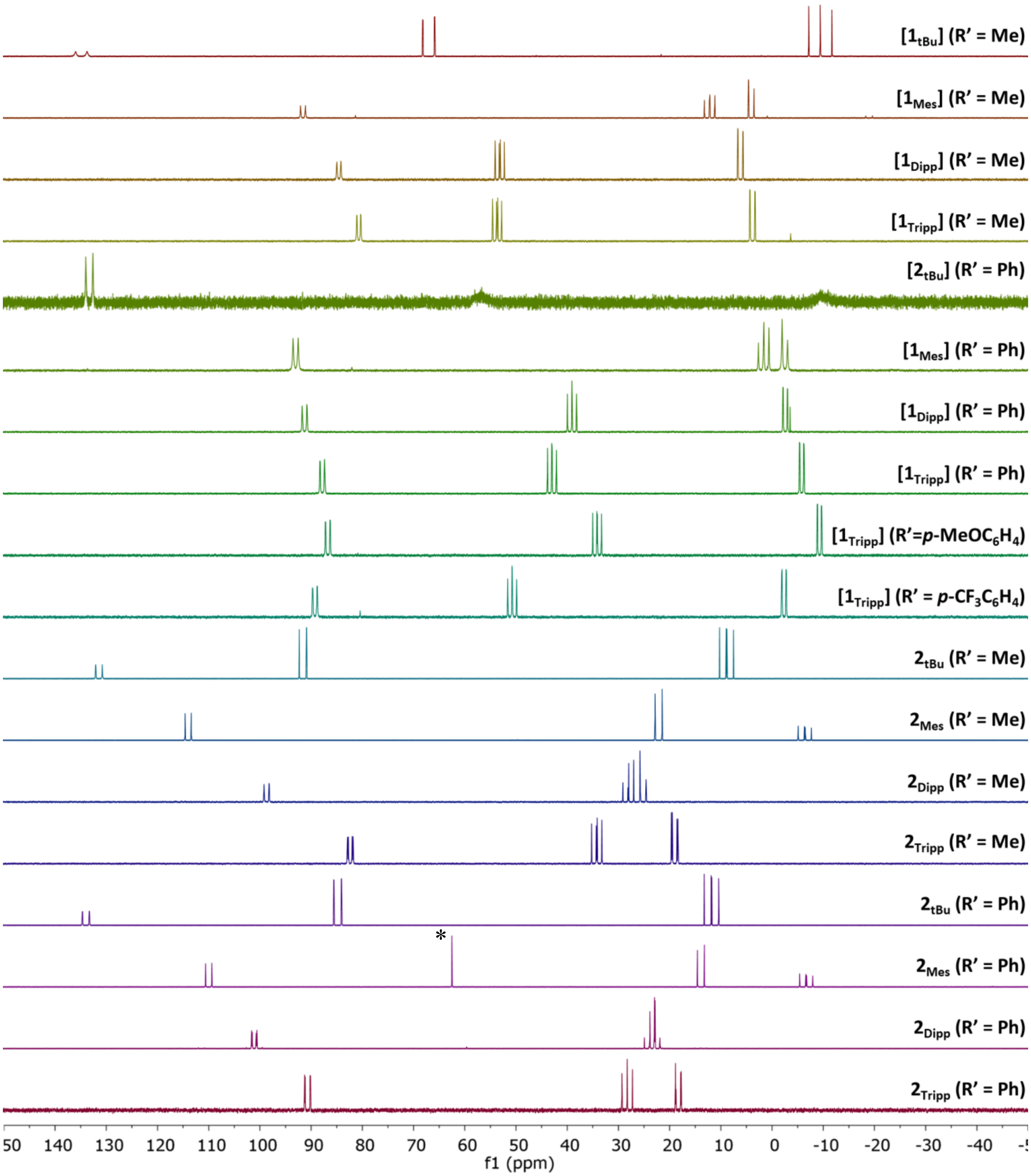

Figure S-94 Stacked $^{31}P\{^{1}H\}$ NMR spectra in $CDCl_3$ of 1-aza-2,3,4-triphospholenes synthesized herein (* denotes unknown impurities)

3. UV-Visible Spectroscopy and Photostability Tests

3.1 General Remarks

Triflate salts used for UV-Visible spectroscopy were washed with copious amounts of *n*-hexane and recrystallized prior to use. Free bases were recrystallized prior to use and separation from [NEt$_3$H][OTf] was verified by ^{1}H NMR spectrometry. All solution UV-Vis spectra were obtained in DCM in Spectrosil® Far UV quartz window cuvettes sealed with GL14 caps (Starna Cells, Inc.). Samples were prepared using volumetric flasks in a dinitrogen-filled MBraun 200B glovebox which was maintained at 20 °C. Spectra were obtained either using an Agilent Technologies Cary 60 UV-Vis spectrometer at the medium speed setting from which a DCM background was obtained separately and manually subtracted, or on an Agilent Technologies Cary 60 UV-Vis spectrometer from 300-800 nm coupled to a Cary Single Cell Peltier thermocouple at 20 °C at the medium speed setting from which a DCM background was subtracted automatically during acquisition. All compounds in the study were found to follow Beer-Lambert law, as determined by the acquisition of solution UV-VIS spectra of all samples across one order of magnitude. Multiple absorption spectra were not acquired for **2_{tBu} (R' = Me)** and **2_{Dipp} (R' = Me)** which exhibited no clear absorbance bands beyond 350 nm.

The solid state UV-Visible spectra of **$[1_{Tipp}]^+$ (R = Me), $[1_{Tipp}]^+$ (R = Ph)**, **$[1_{tBu}]^+$ (R = Me)**, and **$[1_{tBu}]^+$ (R = Me)** were obtained by drop casting a DCM solution of the respective molecules onto the inner side of a Spectrosil® Far UV quartz window cuvette sealed with a GL14 cap (Starna Cells, Inc.). This resulted in the slow-evaporation of the DCM and the deposition of small microcrystalline films which were found to be of adequate translucency to obtain UV-Vis spectra using an Agilent Technologies Cary 60 UV-Vis spectrometer, as for a solution spectrum. This method afforded spectra which revealed absorbance bands in accordance with those seen in solution, but was found to be inadequate for **$[1_{tBu}]^+$ (R = Me)**. As such, amorphous powder of **$[1_{tBu}]^+$ (R = Me)** was placed between two glass slides in a nitrogen-filled glovebox and was sealed at the edges with epoxy glue to protect it from atmospheric moisture before removing the sample from the glovebox. The solid-state reflectance UV-Vis spectrum of **$[1_{tBu}]^+$ (R = Me)** was then collected on a Perkin Elmer Lambda 1050 UV/Vis/NIR spectrometer equipped with a Perkin Elmer 150mm InGaAs integrating , demonstrating a band consistent with solution measurements of **$[1_{tBu}]^+$ (R = Me)**.

[1$_{Tipp}$]$^+$ (R' = Me)

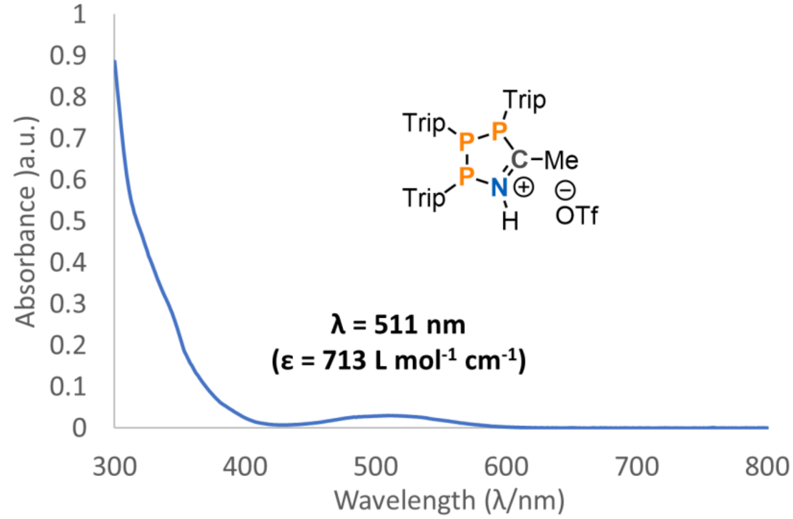

Figure S-95 UV-Visible spectrum of [1$_{Tipp}$]$^+$ (R' = Me), 4.660x10-5 M DCM solution

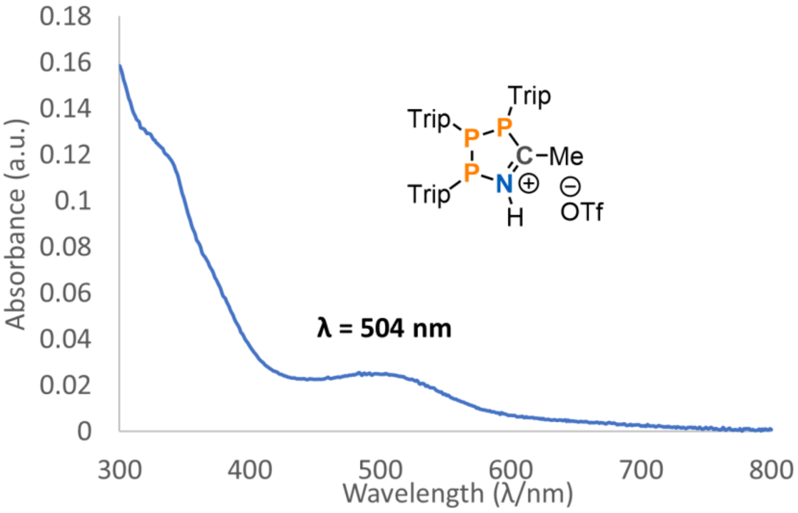

Figure S-96 UV-Visible spectrum of [1$_{Tipp}$]$^+$ (R' = Me), solid state dropcast thin film

[1$_{Tipp}$]$^+$ (R' = Ph)

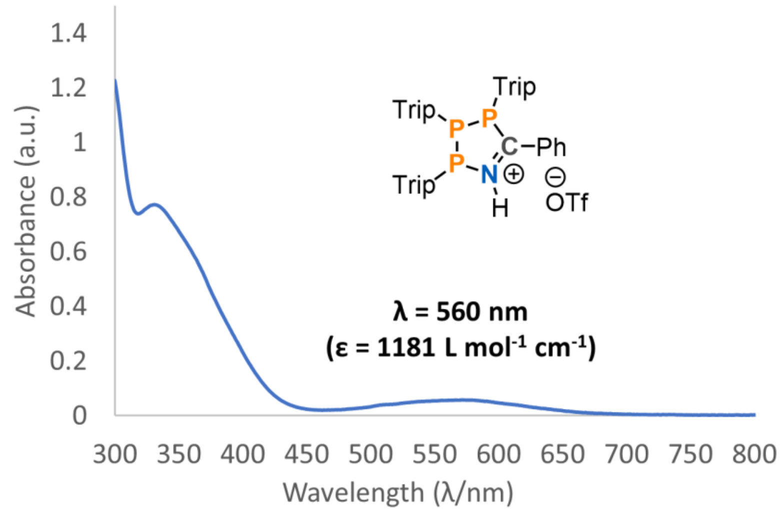

Figure S-97 UV-Visible spectrum of [1$_{Tipp}$]$^+$ (R' = Ph), 5.229x10^{-5} M DCM solution

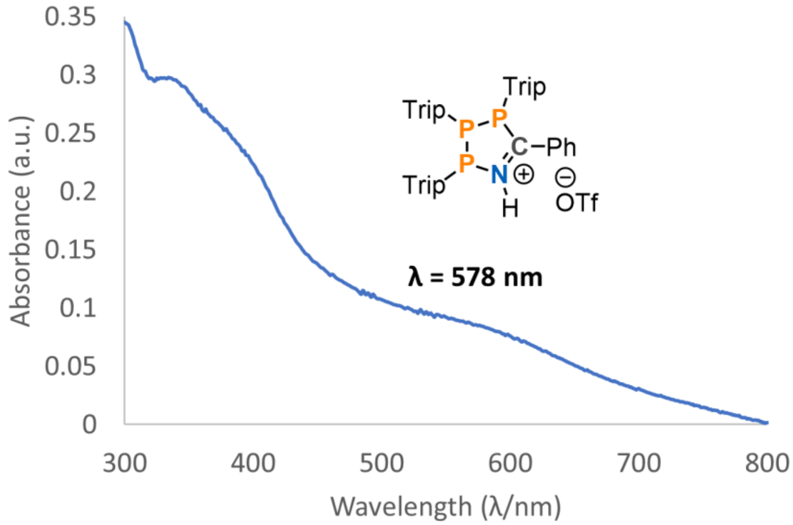

Figure S-98 UV-Visible spectrum of [1$_{Tipp}$]$^+$ (R' = Ph), solid state dropcast thin film

$[1_{Tipp}]^+$ (R' = *p*-$MeOC_6H_4$)

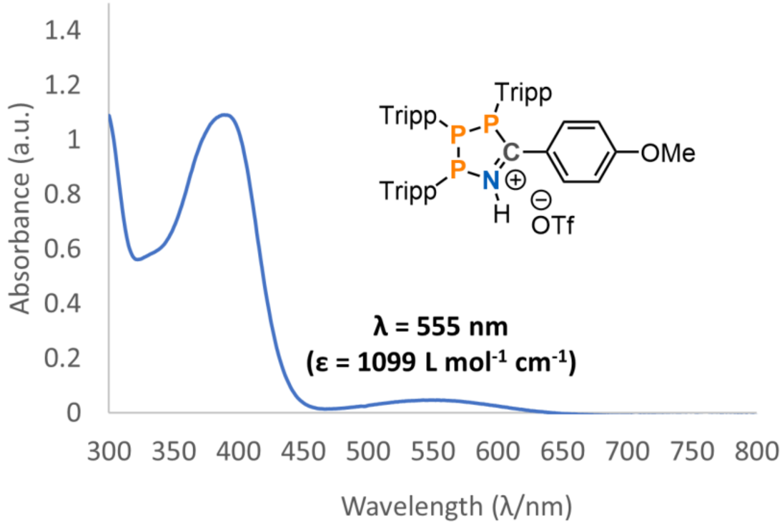

Figure S-99 UV-Visible spectrum of $[1_{Tipp}]^+$ (R' = *p*-$MeOC_6H_4$), 5.070x10^{-5} M DCM solution

$[1_{Tipp}]^+$ (R' = *p*-$CF_3C_6H_4$)

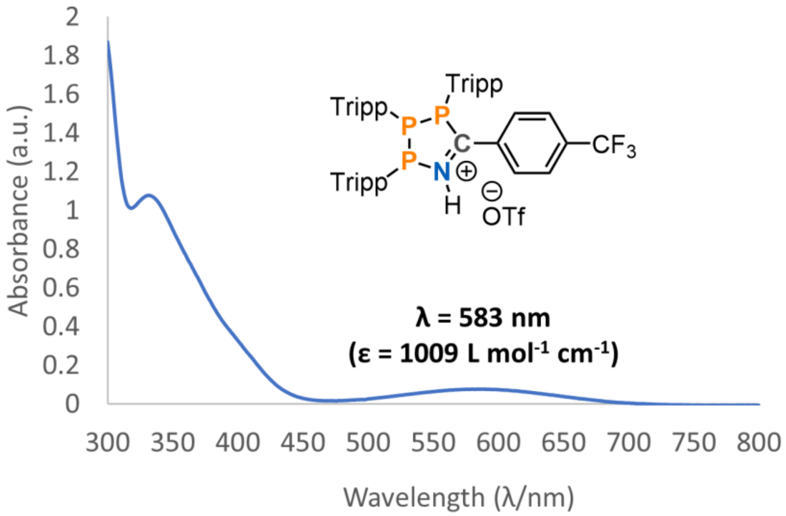

Figure S-100 UV-Visible spectrum of $[1_{Tipp}]^+$ (R' = *p*-$CF_3C_6H_4$), 8.202x10^{-6} M DCM solution

[1$_{Dipp}$]$^+$ (R' = Me)

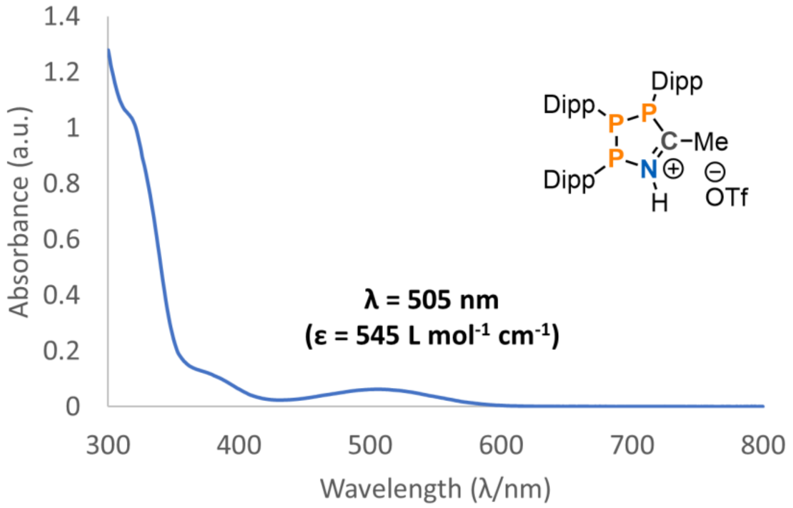

Figure S-101 UV-Visible spectrum of [1$_{Dipp}$]$^+$ (R' = Me), 1.085x10^{-4} M DCM solution

[1$_{Dipp}$]$^+$ (R' = Ph)

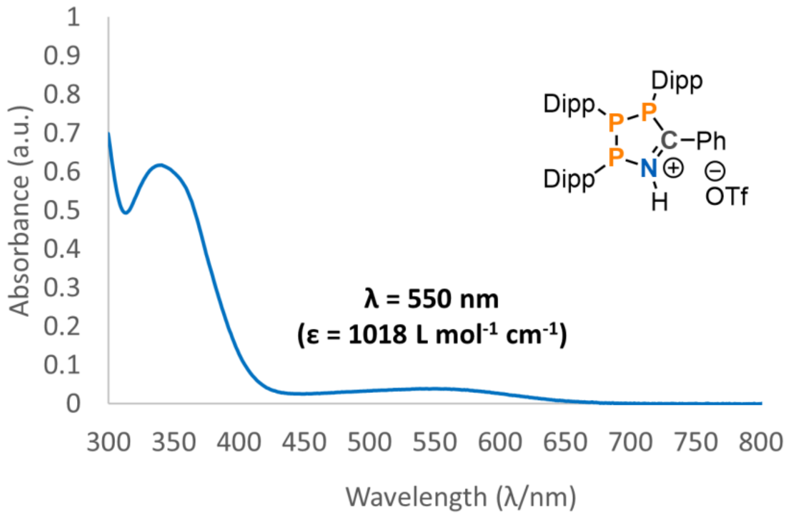

Figure S-102 UV-Visible spectrum of [1$_{Dipp}$]$^+$ (R' = Ph), 6.025x10^{-5} M DCM solution

[1$_{Mes}$]$^+$ (R' = Ph)

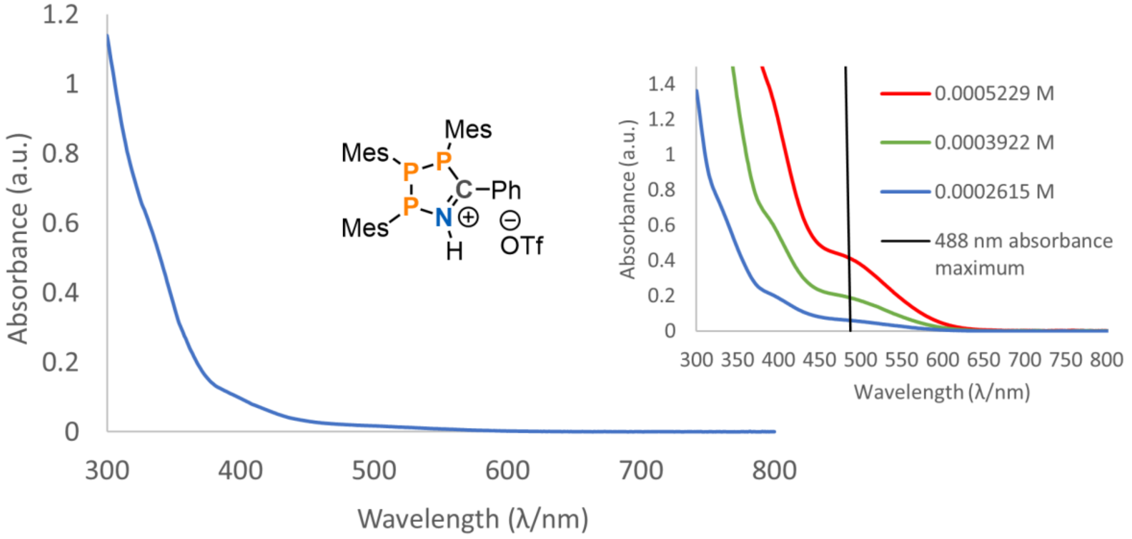

Figure S-103 UV-Visible spectrum of [1$_{Mes}$]$^+$ (R' = Ph), 1.112x10^{-4} M DCM solution; inset: higher concentration solutions of [1$_{Mes}$]$^+$ (R' = Ph) demonstrating the appearance of a low absorptivity absorbance band attributed to a charge transfer phenomenon in this system.

[1_{tBu}]$^+$ (R' = Me)

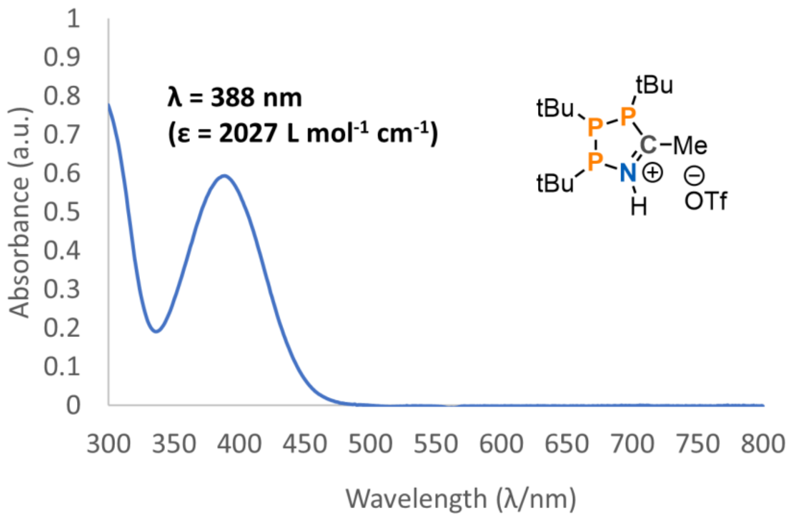

Figure S-104 UV-Visible spectrum of [1_{tBu}]$^+$ (R' = Me), 2.855x10^{-4} M DCM solution

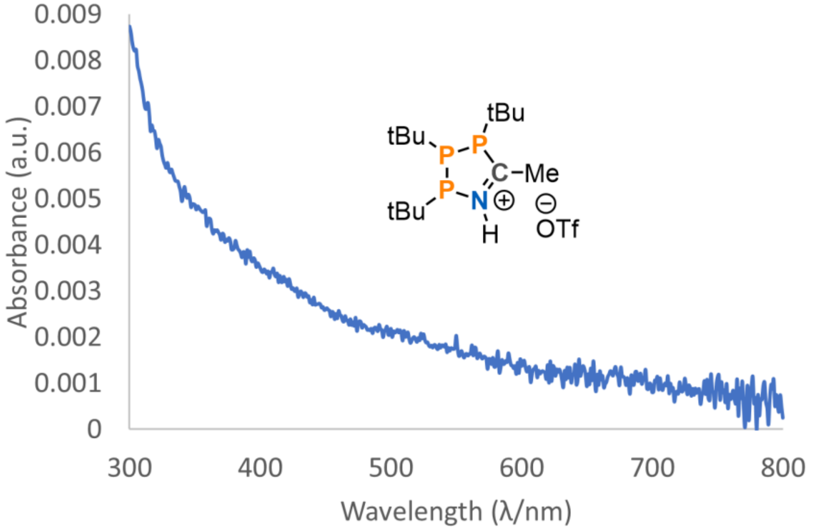

Figure S-105 UV-Visible spectrum of [1_{tBu}]$^+$ (R' = Me), solid state, drop-cast thin film

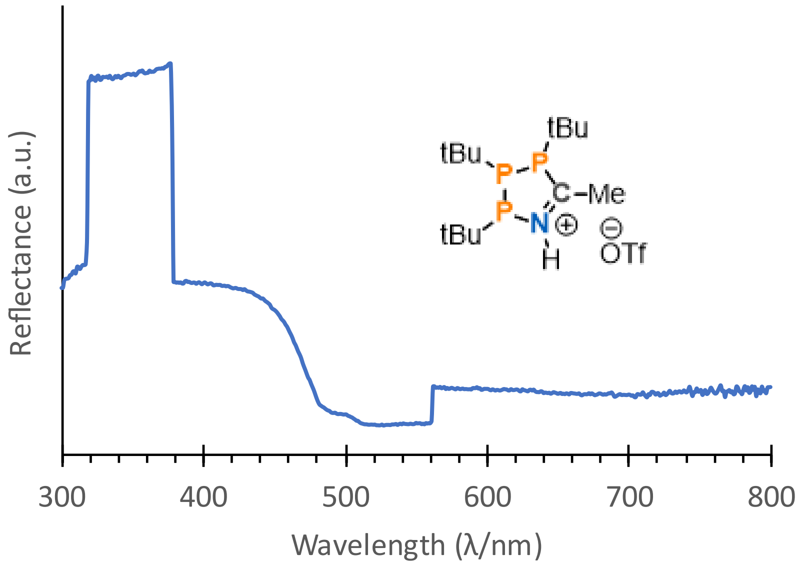

Figure S-106 Background-subtracted solid-state reflectance UV-Vis spectrum of **$[1_{tBu}]^+$ (R = Me)**. Vertical displacements in the spectrum at 560 nm, 380 nm, and 320 nm are due to the switching of lamps in the instrument.

[1_{tBu}]$^+$ (R' = Ph)

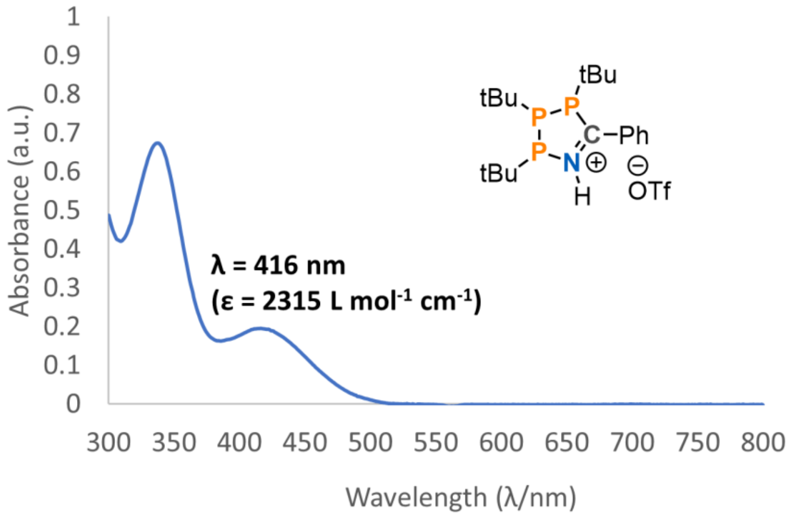

Figure S-107 UV-Visible spectrum of [1_{tBu}]$^+$ (R' = Ph), 8.827x10^{-5} M DCM solution

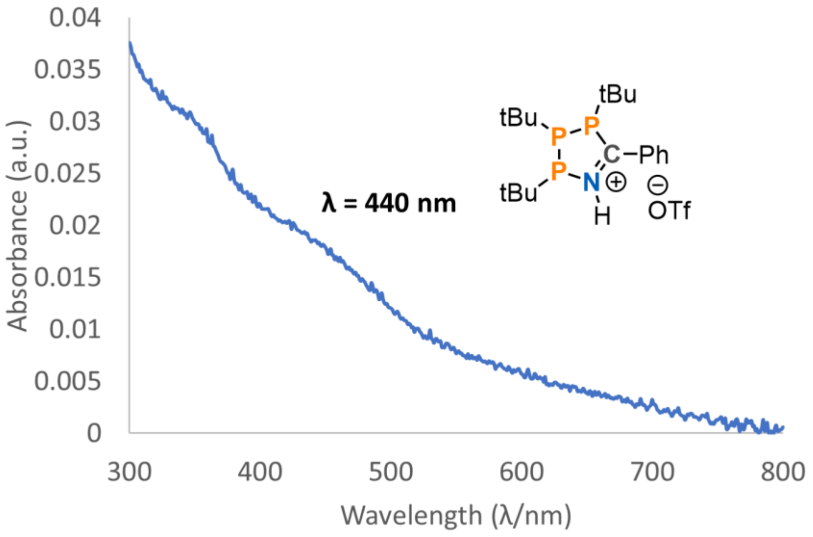

Figure S-108 UV-Visible spectrum of [1_{tBu}]$^+$ (R' = Ph), solid state dropcast thin film

2$_{Tipp}$ (R’ = Me)

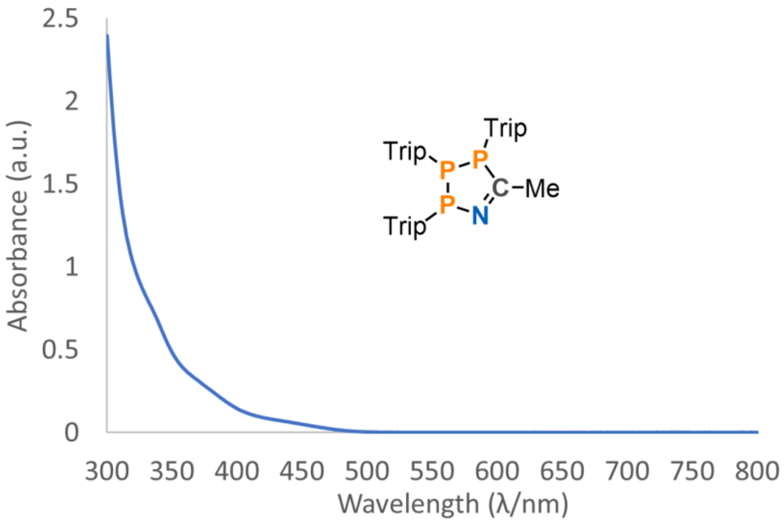

Figure S-109 UV-Visible spectrum of 2$_{Tipp}$ (R’ = Me), 6.720x10^{-5} M DCM solution

2$_{Tipp}$ (R’ = Ph)

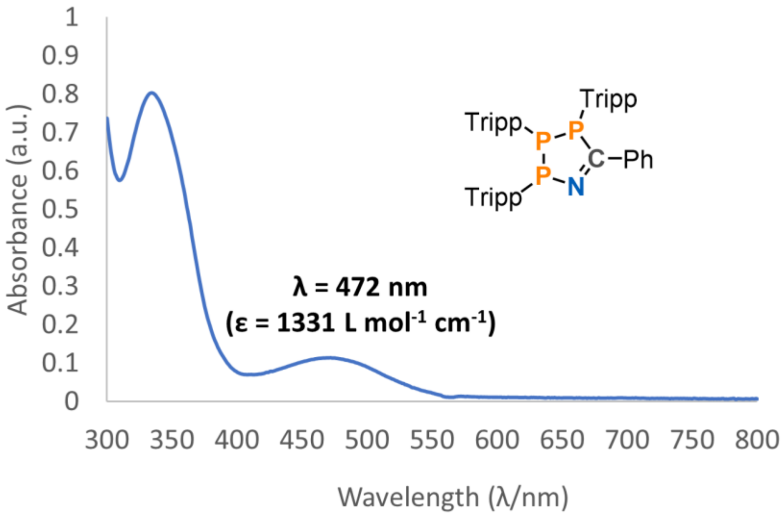

Figure S-110 UV-Visible spectrum of 2$_{Tipp}$ (R’ = Ph), 6.947x10^{-5} M DCM solution

2_{Dipp} (R' = Me)

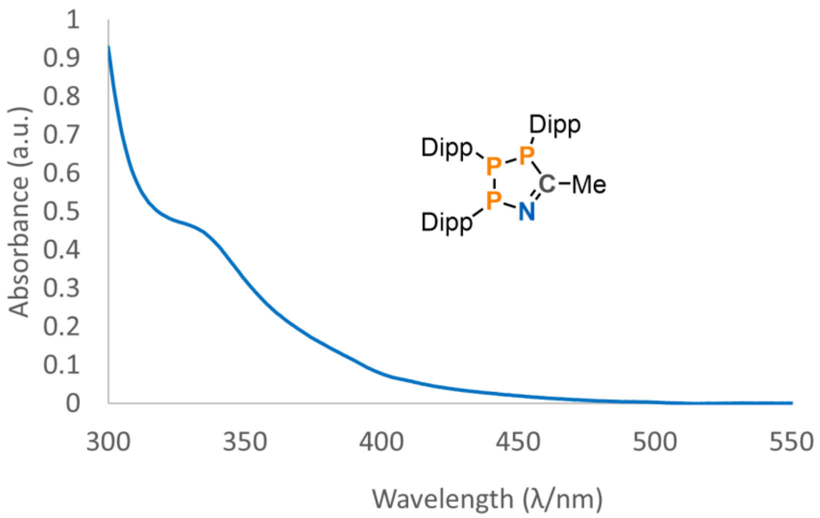

Figure S-111 UV-Visible spectrum of 2_{Dipp} (R' = Me), 9.719×10^{-5} M DCM solution

2_{Dipp} (R' = Ph)

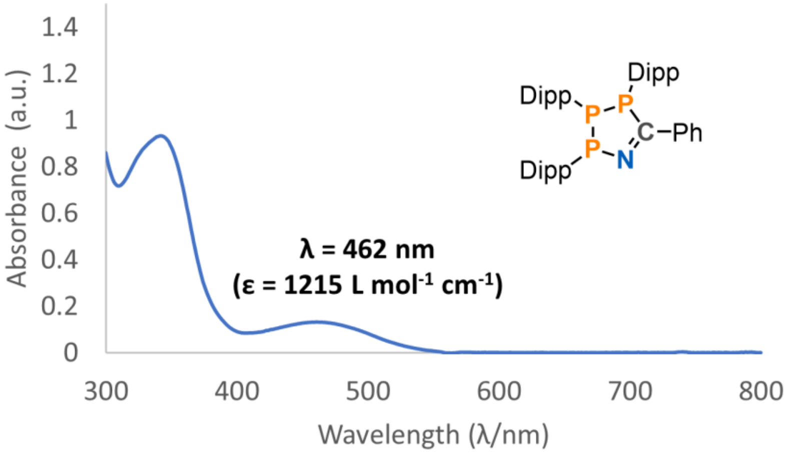

Figure S-112 UV-Visible spectrum of 2_{Dipp} (R' = Ph), 1.108×10^{-4} M DCM solution

2_{tBu} (R' = Me)

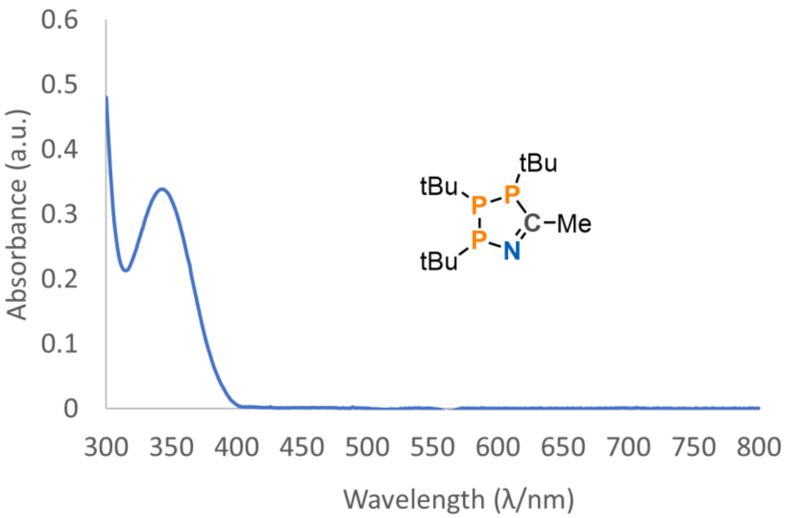

Figure S-113 UV-Visible spectrum of 2_{tBu} (R' = Me), 1.283x10^{-4} M DCM solution

2_{tBu} (R' = Ph)

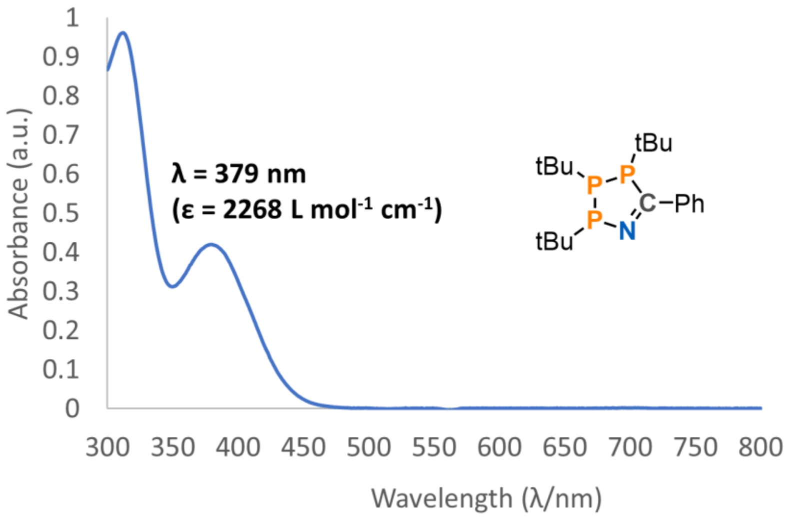

Figure S-114 UV-Visible spectrum of 2_{tBu} (R' = Ph), 1.851x10^{-4} M DCM solution

Photostability of **[1_{Tipp}]$^+$ (R = Ph)**

Photostability of **[1_{Tipp}]$^+$ (R = Ph)** was assessed by exposing a solution of **[1_{Tipp}]$^+$ (R = Ph)** in a J Young NMR tube to a solar irradiation LED lamp (Bridgelux® Gen 7 Vero® 29 Array White LED) with a strength of 48 mW cm^{-1} for 72 h and comparing the NMR spectrum before and after to determine if substantial degradation had occurred.

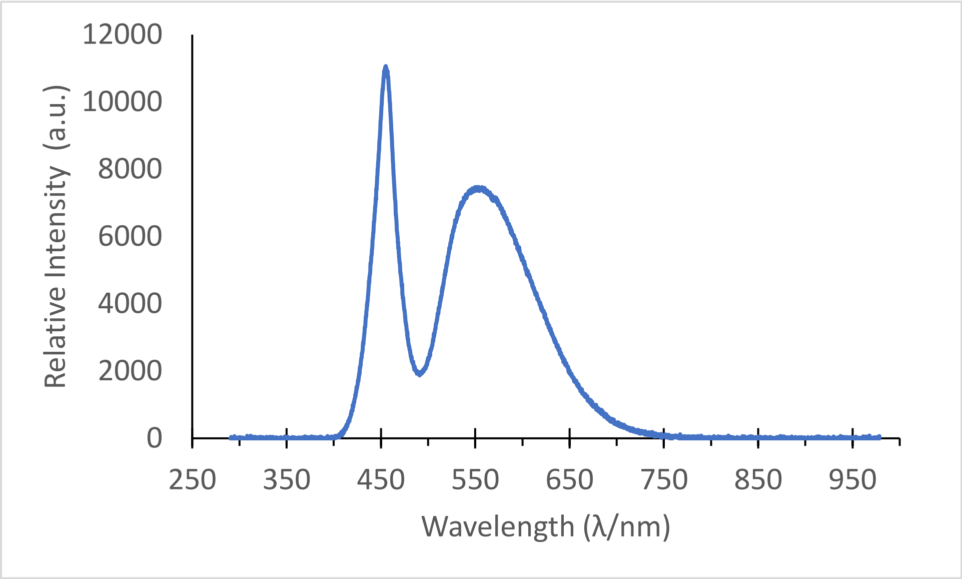

Figure S-115 Spectrum of solar simulation lamp, with strength of 48 mW cm^{-1}

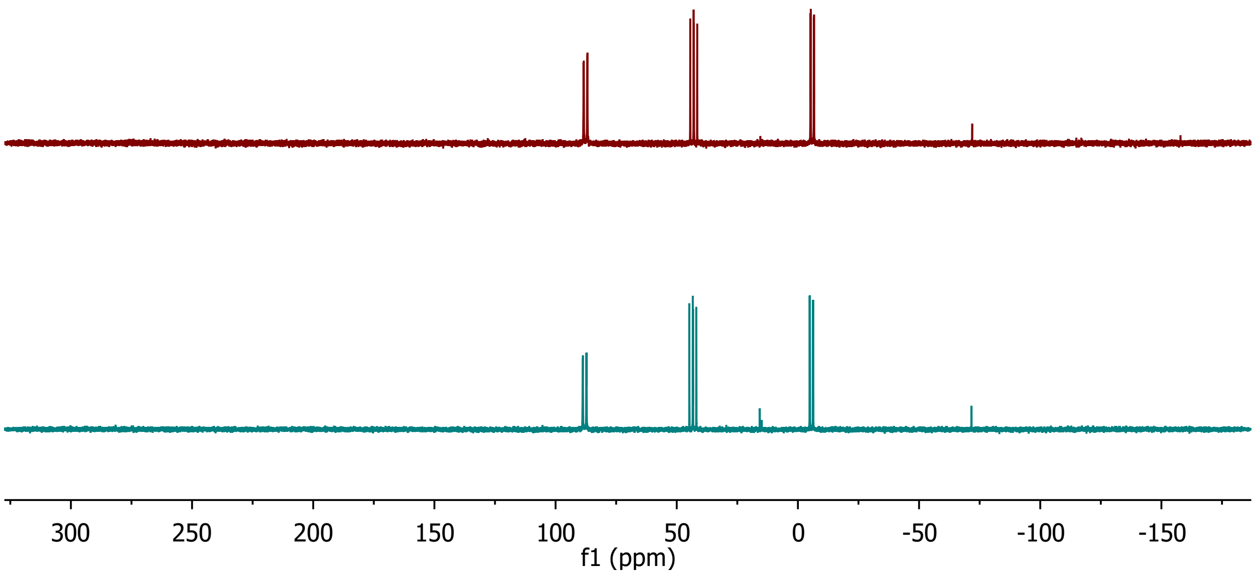

Figure S-116 Stacked spectra of **[1_{Tipp}]$^+$ (R = Ph)** before irradiation (top, red) and after irradiation for 72 h (bottom, green)

4. EPR Analysis

EPR spectra in both solid state and solution are shown for **[1$_{Tipp}$]$^+$ (R' = Ph)**, as a prototypical example to demonstrate the diamagnetic nature of the species reported herein.

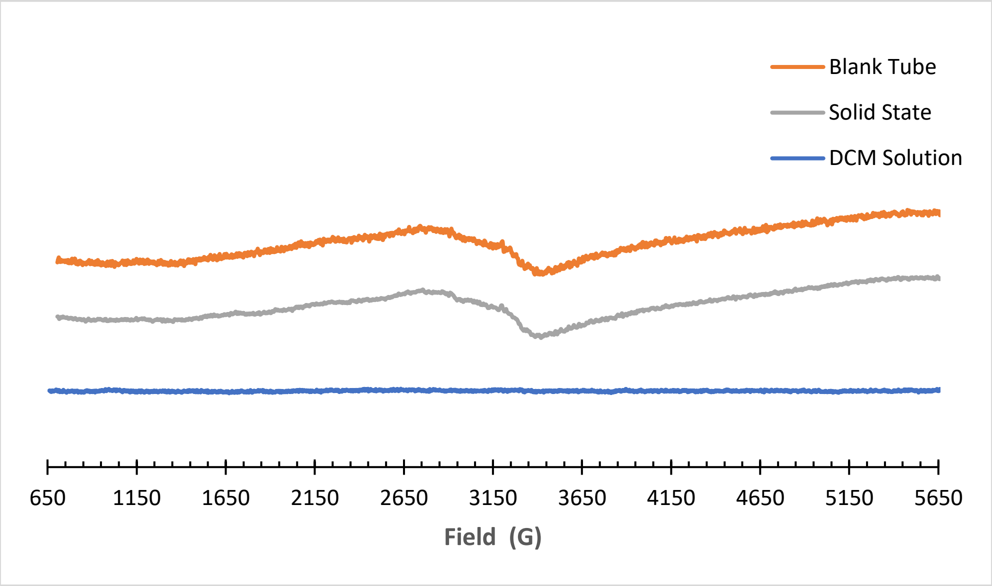

5. Crystallographic Details

5.1 General Remarks

Red blocks of **[1$_{Tipp}$]$^+$ (R' = Me)** were grown from layering *n*-hexane over a fluorobenzene solution at -30 °C. Dark blue blocks of **[1$_{Tipp}$]$^+$ (R' = Ph)** were grown from layering *n*-hexane over a fluorobenzene solution at -30 °C. Red blocks of **{[1$_{Dipp}$]$^+$ (R' = Me)}·PhF** were grown from layering *n*-hexane over a fluorobenzene solution at -30 °C. Dark blue blocks of **[1$_{Dipp}$]$^+$ (R' = Ph)** were grown from layering *n*-hexane over a fluorobenzene solution at -30 °C. Yellow blocks of **[1$_{Mes}$]$^+$ (R' = Me)**·were grown via the slow diffusion of a *n*-hexane/pentane mixture into a fluorobenzene solution doped with a small amount of toluene at -30 °C. Orange blocks of **[1$_{Mes}$]$^+$ (R' = Ph)** were grown from layering a fluorobenzene solution with *n*-hexane at -30 °C. Orange blocks of **{[1$_{tBu}$]$^+$ (R' = Ph)}·DCM** were grown by layering a DCM solution with *n*-hexane at -30 °C. Black-blue rectangular prisms of **[1$_{Tipp}$]$^+$ (R' = *p*-CF$_3$C$_6$H$_4$)** were grown by layering a fluorobenzene solution with *n*-hexane at -30 °C. Burgundy prisms of **[1$_{Tipp}$]$^+$ (R' = *p*-MeOC$_6$H$_4$)** were grown by layering a fluorobenzene solution with *n*-hexane at -30 °C. Pale yellow prisms of **2$_{Tipp}$ (R' = Me)** were grown from a saturated pentane solution at -30 °C. Orange needles of **2$_{Tipp}$ (R' = Ph)** were grown from a saturated acetonitrile solution at -30 °C. Yellow blocks of **2$_{Dipp}$ (R' = Me)** were grown from a saturated *n*-hexane solution at -30 °C. Orange prisms of **2$_{Dipp}$ (R' = Ph)** were grown from a saturated pentane solution at -30 °C. Colourless blocks of **2$_{Mes}$ (R' = Me)** were grown from a saturated *n*-hexane solutions at -30 °C. Yellow blocks of **2$_{Mes}$ (R' = Ph)** were grown from a saturated *n*-hexane solutions at -30 °C. Yellow needles of **2$_{tBu}$ (R' = Ph)** were grown from a saturated *n*-hexane solution at -30 °C.

The single crystal X-ray diffraction experiments were carried out on a Bruker APEX II diffractometer using either Mo-Kα radiation (λ = 0.71073 Å) or Cu-Kα radiation (λ = 1.54178 Å) and collected at 90 (2) or 100(2) K. The data collection was performed using a CCD area detector from a single crystal mounted on a glass fibre. The total number of runs and images was based on the strategy calculation from the program APEX3. Data reduction, scaling and absorption corrections were performed using SAINT. The structures were solved and the space group determined by the SHELXT[6] structure solution program using Intrinsic Phasing and refined by Least Squares using version 2018/3 of SHELXL[7] in Olex2.[8] All non-hydrogen atoms were refined anisotropically. Carbon-bound hydrogen atom positions were calculated geometrically and refined using the riding model, whereas all nitrogen-bound hydrogen atoms were located in the Fourier

difference map and freely refined. In the case of **[1$_{tBu}$]$^+$ (R' = Ph)** the PLATON SQUEEZE solvent mask was used due to the presence of severely disordered DCM molecule in the lattice.[9] A disordered CF_3 group in **[1$_{Tipp}$]$^+$ (R' = *p*-$CF_3C_6H_4$)** was successfully modelled over two positions and refined with the sum of the occupancies set to 1. A fluorobenzene molecule in the **{[1$_{Dipp}$]$^+$ (R' = Me)}·PhF** solvate demonstrated positional disorder and was successfully modelled over 3 positions with the sum of the occupancies set to 1. **2$_{Mes}$ (R' = Me)** crystallized with two crystallographically unique molecules in the asymmetric unit (Z = 8, Z' = 2), related to each other by two-fold symmetry along the c-axis. **2$_{Mes}$ (R' = Ph)** also crystallized with two crystallographically unique molecules in the asymmetric unit (Z = 4, Z' = 2), related to each other by inversion symmetry.

Below we briefly discuss some trends in core P_3CN bond lengths of the synthesized 1-aza-2,3,4-triphospholenes. For this discussion, we will refer to the phosphorus atoms of the central P_3CN framework as P_C (phosphorus bound to the nitrile carbon atom), P_N (phosphorus bound to the nitrile nitrogen atom), and P_P for the phosphorus atom bound between P_C and P_N.

C=N: When comparing the **[1$_R$]$^+$ (R' = Me)** series, the length of C=N bonds are within experimental error for both, with values of 1.299(2) Å in **[1$_{Dipp}$]$^+$** to 1.309(9) Å in **[1$_{Mes}$]$^+$** and the **2$_R$ (R' = Me)** series was similarly invariant with bond lengths of 1.278(6) Å in **2$_{tBu}$** to 1.289(6) Å in **2$_{Tipp}$**. Similarly, the C=N bonds for the **[1$_R$]$^+$ (R' = Ph)** series are within experimental error, with values of 1.309(4) in **[1$_{Mes}$]$^+$** to 1.312(4) Å in **[1$_{Dipp}$]$^+$**, while deprotonation to **2$_R$ (R' = Ph)** lowered the bond lengths to a narrow range of 1.280(3) Å in **2$_{Mes}$** to 1.294(5) Å in **2$_{Tipp}$**. Overall, a shortening in C=N bond length is observed upon deprotonation,

P$_C$-P: **[1$_{Tipp}$]$^+$ (R' = Me)** and **[1$_{Dipp}$]$^+$ (R' = Me)** have slightly longer bonds (2.226(1) Å and 2.2360(5) Å, respectively) than **[1$_{Mes}$]$^+$ (R' = Me)** and **[1$_{tBu}$]$^+$ (R' = Me)** (2.192(3) Å and 2.192(1) Å, respectively). Deprotonation results in **2$_{Tipp}$ (R' = Me)** and **2$_{Dipp}$ (R' = Me)** having bonds shortened to 2.185(1) Å and 2.1947(5) Å, while both crystallographically independent molecules in **2$_{Mes}$ (R' = Me)** (2.193(1) Å, 2.192(1) Å) and **2$_{tBu}$ (R' = Me)** (2.191(2) Å) are within experimental error relative to their protonated forms. Similar trends were seen in **[1$_R$]$^+$ (R' = Ph)** species. P_C-P distances incrementally increased from 2.1958(4) Å in **[1$_{tBu}$]$^+$ (R' = Ph)** to 2.2262(4) Å in **[1$_{Tipp}$]$^+$ (R' = Ph)**. For **2$_R$ (R' = Ph)** species, similar trends to **2$_R$ (R' = Me)** species are seen in P_C-P bonds, with **2$_{tBu}$ (R' = Ph)** showing virtually no change in length (2.1935(3) Å) from

[1_{tBu}]$^+$ (R' = Ph), and slight but steady shortening in the lengths of **2_{Mes} (R' = Ph)** (2.1861(9)/2.1907(8) Å), **2_{Dipp} (R' = Ph)** (2.1972(5) Å), and **2_{Tipp} (R' = Ph)** (2.201(1) Å) compared to their protonated states.

P_N-P: In the case of P_N-P bonds, all **[1_R]$^+$ (R' = Me)** species are within error (2.233(1) Å, 2.2387(6) Å, 2.237(3) Å and 2.212(1) Å for R = Tipp, Dipp, Mes, *t*Bu respectively), but upon deprotonation P_N-P bonds in **2_R (R = Tipp, Dipp, Mes; R' = Me)** are lengthened to comparable values of 2.265(2), 2.2682(5), and 2.271(2)/2.266(2) Å in R = Tipp, Dipp, and Mes respectively, while **2_{tBu} (R' =Me)** slightly lengthens to 2.227(1) Å. Similar trends were seen in **[1_R]$^+$ (R' = Ph)** species. P_N-P distances ranged from 2.2086(6) Å for **[1_{tBu}]$^+$ (R' = Ph)** to 2.2458(6) Å for **[1_{Tipp}]$^+$ (R' = Ph)**, while bond lengths increase steadily from **2_{tBu} (R' = Ph)** (2.2322(3) Å) to **2_{Mes} (R' = Ph)** (2.2497(9)/2.256(1) Å) to **2_{Dipp} (R' = Ph)** (2.2725(9) Å), while **2_{Tipp} (R' = Ph)** increases only slightly to 2.256(1) Å.

P_N-N and P_C-C: We noted consistent differences in the distances of P_N-N and P_C-C bond distances between protonated and deprotonated states across all the species in our study regardless of substituent at phosphorus. This is illustrated by **[1_{Tipp}]$^+$ (R' = Ph)/2_{Tipp} (R' = Ph)** as a comparative example. When considering the P_N-N bond, **[1_{Tipp}]$^+$ (R' = Ph)** measures 1.775(1) Å similar to those of other **[1_R]$^+$ (R' = Ph)** species, while deprotonation lowers the P_N-N bond length to 1.708(3) Å. In the case of the P_C-C bond, **[1_{Tipp}]$^+$ (R' = Ph)** measures 1.820(1) Å similarly to the other **[1_R]$^+$ (R' = Ph)** species, while deprotonation increases the bond length to 1.853(4) Å. In comparing bond distances in the P3CN core across the series of **[1_R]$^+$ (R' = Ph, *p*-MeOC_6H_4, *p*-$CF_3C_6H_4$)**, no substantial differences were found, and as such their structural differences manifest solely in a change of the angle of deplanarization.

Table S-1 Summary of Crystallographic Data, Part 1

	**$[1_{Tipp}]^+$ (R' = Me) CCDC 2293093**	**$[1_{Tipp}]^+$ (R' = Ph) CCDC 2293094**	**$\{[1_{Dipp}]^+$ (R' = Me)$\}\cdot$PhF CCDC 2293086**	**$[1_{Dipp}]^+$ (R' = Ph) CCDC 2293087**	**$[1_{Mes}]^+$ (R' = Me) CCDC 2293088**	**$[1_{Mes}]^+$ (R' = Ph) CCDC 2293089**	**$[1_{tBu}]^+$ (R' = Ph) CCDC 2293090**
Empirical formula	$C_{48}H_{73}F_3NO_3P_3S$	$C_{53}H_{75}NO_3F_3P_3S$	$C_{45}H_{60}F_4NO_3P_3S$	$C_{44}H_{57}NO_3F_3P_3S$	$C_{30}H_{37}F_3NO_3P_3S$	$C_{35}H_{39}F_3NO_3P_3S$	$C_{20}H_{33}F_3NO_3P_3S$
Formula weight	894.04	956.11	863.91	829.87	641.57	703.64	517.44
Temperature/K	100(2)	100(2)	100(2)	100(2)	100(2)	100(2)	100(2)
Crystal system	monoclinic	triclinic	monoclinic	triclinic	monoclinic	triclinic	monoclinic
Space group	$P2_1/c$	P-1	$P2_1/n$	P-1	$P2_1/n$	P-1	$P2_1/c$
a/Å	13.0397(5)	13.2057(6)	10.6808(4)	10.8554(10)	11.5917(10)	11.005(2)	9.5059(5)
b/Å	21.2666(8)	14.4979(7)	20.4088(8)	11.7203(11)	22.103(2)	12.506(2)	14.4773(8)
c/Å	18.7063(7)	16.1649(8)	20.8267(8)	18.4078(16)	13.3286(12)	15.198(3)	21.4699(12)
α/°	90	65.0680(10)	90	106.573(2)	90	113.278(6)	90
β/°	101.2170(10)	79.1030(10)	96.752(2)	104.787(2)	115.243(3)	98.207(7)	91.586(2)
γ/°	90	64.9930(10)	90	94.530(3)	90	93.456(7)	90
Volume/Å^3	5088.4(3)	2543.1(2)	4508.4(3)	2141.1(3)	3088.8(5)	1886.1(6)	2953.6(3)
Z	4	2	4	2	4	2	4
ρ_{calc}g/cm^3	1.167	1.249	1.273	1.287	1.380	1.239	1.164
μ/mm^{-1}	0.207	0.212	0.234	0.241	0.311	0.261	0.309
F(000)	1920	1024	1832	880	1344	736	1088
Crystal size/mm^3	0.13 × 0.1 × 0.05	0.14 × 0.12 × 0.06	0.35 × 0.22 × 0.2	0.2 × 0.17 × 0.12	0.15 × 0.09 × 0.05	0.11 × 0.09 × 0.04	0.41 × 0.37 × 0.35
Radiation	MoKα (λ = 0.71073)	MoKα (λ = 0.71073)	Mo Kα (λ = 0.71073)	MoKα (λ = 0.71073)	MoKα (λ = 0.71073)	MoKα (λ = 0.71073)	MoKα (λ = 0.71073)
2Θ range for data collection/°	3.184 to 50.862	2.778 to 61.258	2.804 to 61.174	3.676 to 52.918	3.686 to 44.992	3.574 to 55.912	6.31 to 61.024
Index ranges	-13 ≤ h ≤ 15, -25 ≤ k ≤ 25, -22 ≤ l ≤ 22	-18 ≤ h ≤ 18, -20 ≤ k ≤ 20, -23 ≤ l ≤ 23	-15 ≤ h ≤ 15, -29 ≤ k ≤ 23, -29 ≤ l ≤ 29	-13 ≤ h ≤ 13, -14 ≤ k ≤ 14, -22 ≤ l ≤ 23	-12 ≤ h ≤ 11, 0 ≤ k ≤ 23, 0 ≤ l ≤ 14	-14 ≤ h ≤ 14, -16 ≤ k ≤ 16, -20 ≤ l ≤ 18	-13 ≤ h ≤ 13, 0 ≤ k ≤ 20, 0 ≤ l ≤ 30
Reflections collected	50698	63351	60974	34614	4016	20519	48692
Independent reflections	9359 [R_{int} = 0.0762, R_{sigma} = 0.0706]	15654 [R_{int} = 0.0503, R_{sigma} = 0.0480]	13812 [R_{int} = 0.0373, R_{sigma} = 0.0341]	8818 [R_{int} = 0.0502, R_{sigma} = 0.0553]	4016 [R_{int} = 0.222, R_{sigma} = 0.1394]	9011 [R_{int} = 0.0667, R_{sigma} = 0.1052]	8885 [R_{int} = 0.045, R_{sigma} = 0.0331]
Data/restraints/parameters	9359/36/582	15654/0/599	13812/0/534	8818/0/512	4016/0/381	9011/0/428	8885/0/293
Goodness-of-fit on F^2	1.1	1.012	1.026	1.018	1.064	0.967	1.081
Final R indexes [I>=2σ (I)]	R_1 = 0.0674, wR_2 = 0.1632	R_1 = 0.0400, wR_2 = 0.0880	R_1 = 0.0408, wR_2 = 0.1005	R_1 = 0.0515, wR_2 = 0.1183	R_1 = 0.0852, wR_2 = 0.1812	R_1 = 0.0526, wR_2 = 0.1084	R_1 = 0.0410, wR_2 = 0.1112
Final R indexes [all data]	R_1 = 0.1110, wR_2 = 0.1816	R_1 = 0.0613, wR_2 = 0.0979	R_1 = 0.0575, wR_2 = 0.1094	R_1 = 0.0823, wR_2 = 0.1376	R_1 = 0.1575, wR_2 = 0.2095	R_1 = 0.0928, wR_2 = 0.1261	R_1 = 0.0507, wR_2 = 0.1148
Largest diff. peak/hole / e Å^{-3}	1.61/-0.62	0.61/-0.41	0.66/-0.47	0.56/-0.47	0.38/-0.44	0.40/-0.40	0.49/-0.62

Table S-2 Summary of Crystallographic Data, Part 2

	**2_{Tipp} (R' = Me) CCDC 2293100**	**2_{Tipp} (R' = Ph) CCDC 2293101**	**2_{Dipp} (R' = Me) CCDC 2293095**	**2_{Dipp} (R' = Ph) CCDC 2293096**	**2_{Mes} (R' = Me) CCDC 2293097**	**2_{Mes} (R' = Ph) CCDC 2293098**	**2_{tBu} (R' = Ph) CCDC 2293099**
Empirical formula	$C_{47}H_{72}NP_3$	$C_{52}H_{74}NP_3$	$C_{38}H_{54}NP_3$	$C_{43}H_{56}NP_3$	$C_{29}H_{36}NP_3$	$C_{34}H_{38}NP_3$	$C_{19}H_{32}NP_3$
Formula weight	743.96	806.03	617.73	679.79	491.5	553.56	367.36
Temperature/K	90(2)	100(2)	100(2)	100(2)	100(2)	100(2)	100(2)
Crystal system	monoclinic	monoclinic	triclinic	triclinic	orthorhombic	triclinic	triclinic
Space group	$P2_1/c$	$P2_1/c$	P-1	P-1	$Pca2_1$	P-1	P-1
a/Å	16.9451(7)	12.9840(4)	11.3450(8)	8.7516(4)	15.6132(9)	13.3026(7)	7.3017(5)
b/Å	13.2118(6)	8.7134(3)	11.9683(8)	11.3824(5)	12.7998(7)	15.2580(8)	9.9102(7)
c/Å	20.9116(10)	42.5367(14)	15.3035(10)	20.2888(11)	26.6995(16)	16.3831(8)	15.2826(11)
α/°	90	90	80.276(2)	86.071(2)	90	103.595(2)	91.383(2)
β/°	104.798(4)	98.699(3)	79.095(2)	89.119(3)	90	90.873(2)	101.010(2)
γ/°	90	90	63.100(2)	79.852(2)	90	109.755(2)	104.534(2)
Volume/Å^3	4526.3(4)	4757.0(3)	1811.4(2)	1984.75(17)	5335.8(5)	3025.6(3)	1047.75(13)
Z	4	4	2	2	8	4	2
ρ_{calc}g/cm^3	1.092	1.125	1.133	1.138	1.224	1.215	1.164
μ/mm^{-1}	1.422	1.39	0.19	0.179	0.241	0.22	0.284
F(000)	1624	1752	668	732	2096	1176	396
Crystal size/mm^3	0.18 × 0.12 × 0.07	0.1 × 0.01 × 0.01	0.26 × 0.14 × 0.09	0.24 × 0.12 × 0.07	0.26 × 0.16 × 0.05	0.2 × 0.096 × 0.064	0.27 × 0.22 × 0.18
Radiation	CuKα (λ = 1.54178)	CuKα (λ = 1.54178)	MoKα (λ = 0.71073)	MoKα (λ = 0.71073)	MoKα (λ = 0.71073)	MoKα (λ = 0.71073)	MoKα (λ = 0.71073)
2Θ range for data collection/°	5.394 to 112.118	4.202 to 112.108	2.722 to 61.234	2.012 to 52.862	3.05 to 55.85	2.934 to 55.928	2.722 to 61.014
Index ranges	-18 ≤ h ≤ 17, 0 ≤ k ≤ 14, 0 ≤ l ≤ 22	-13 ≤ h ≤ 12, -9 ≤ k ≤ 9, -44 ≤ l ≤ 45	-16 ≤ h ≤ 16, -17 ≤ k ≤ 17, -21 ≤ l ≤ 21	-10 ≤ h ≤ 10, -14 ≤ k ≤ 13, -25 ≤ l ≤ 25	-20 ≤ h ≤ 20, -16 ≤ k ≤ 16, -35 ≤ l ≤ 35	-17 ≤ h ≤ 17, -20 ≤ k ≤ 19, -21 ≤ l ≤ 21	-10 ≤ h ≤ 10, -14 ≤ k ≤ 14, -21 ≤ l ≤ 21
Reflections collected	52653	39675	53484	28331	75209	52172	24581
Independent reflections	5896 [R_{int} = 0.199, R_{sigma} = 0.1066]	6153 [R_{int} = 0.1149, R_{sigma} = 0.0775]	11113 [R_{int} = 0.0357, R_{sigma} = 0.0328]	8107 [R_{int} = 0.0308, R_{sigma} = 0.0355]	12747 [R_{int} = 0.0594, R_{sigma} = 0.0461]	13662 [R_{int} = 0.0682, R_{sigma} = 0.0675]	6401 [R_{int} = 0.0354, R_{sigma} = 0.0301]
Data/restraints/parameters	5896/0/479	6153/390/561	11113/0/392	8107/0/436	12747/1/615	13662/0/703	6401/0/217
Goodness-of-fit on F^2	1.066	1.042	0.93	1.037	1.036	1.042	1.032
Final R indexes [I>=2σ (I)]	R_1 = 0.0669, wR_2 = 0.1526	R_1 = 0.0596, wR_2 = 0.1308	R_1 = 0.0363, wR_2 = 0.1134	R_1 = 0.0373, wR_2 = 0.0860	R_1 = 0.0405, wR_2 = 0.0832	R_1 = 0.0519, wR_2 = 0.1250	R_1 = 0.0268, wR_2 = 0.0704
Final R indexes [all data]	R_1 = 0.1001, wR_2 = 0.1677	R_1 = 0.0867, wR_2 = 0.1455	R_1 = 0.0523, wR_2 = 0.1289	R_1 = 0.0507, wR_2 = 0.0925	R_1 = 0.0601, wR_2 = 0.0907	R_1 = 0.0755, wR_2 = 0.1369	R_1 = 0.0301, wR_2 = 0.0723
Largest diff. peak/hole / e Å^{-3}	0.43/-0.26	0.66/-0.35	0.43/-0.19	0.33/-0.21	0.34/-0.22	0.40/-0.32	0.44/-0.23

Table S-3 Summary of Crystallographic Data, Part 3

	$[1_{Tipp}]^+$ (R' = *p*-MeOC_6H_4) CCDC 2293092	$[1_{Tipp}]^+$ (R' = *p*-$CF_3C_6H_4$) CCDC 2293091	$P_6Mes_6 \cdot Et_2O$ CCDC 2293102
Empirical formula	$C_{54}H_{77}F_3NO_4P_3S$	$C_{54}H_{73}F_6NO_3P_3S$	$C_{58}H_{76}OP_6$
Formula weight	986.13	1024.11	975.00
Temperature/K	100(2)	100(2)	100(2)
Crystal system	triclinic	triclinic	monoclinic
Space group	*P*-1	*P*-1	*P*2/*c*
a/Å	13.7735(12)	14.4906(6)	13.273(4)
b/Å	14.3310(12)	15.0696(7)	8.550(3)
c/Å	16.4614(14)	17.5125(9)	24.630(8)
α/°	67.221(2)	69.989(2)	90
β/°	80.664(3)	82.093(2)	97.626(4)
γ/°	64.226(2)	72.732(2)	90
Volume/Å^3	2697.7(4)	3428.6(3)	2770.3(15)
Z	2	2	2
ρ_{calc}g/cm^3	1.214	0.992	1.169
μ/mm^{-1}	0.203	0.167	0.232
F(000)	1056	1088	1044
Crystal size/mm^3	0.27 × 0.1 × 0.03	0.46 × 0.3 × 0.19	0.25×0.20×0.10
Radiation	MoKα (λ = 0.71073)	MoKα (λ = 0.71073)	MoKα (λ = 0.71073)
2Θ range for data collection/°	2.684 to 56.782	3.216 to 52.826	1.668 to 25.332
Index ranges	-18 ≤ h ≤ 17, -19 ≤ k ≤ 18, -21 ≤ l ≤ 21	-17 ≤ h ≤ 18, -18 ≤ k ≤ 18, -21 ≤ l ≤ 21	-15 ≤ h ≤ 15, -10 ≤ k ≤ 10, -29 ≤ l ≤ 19
Reflections collected	50897	71096	21991
Independent reflections	13452 [R_{int} = 0.0643, R_{sigma} = 0.0742]	14047 [R_{int} = 0.0320, R_{sigma} = 0.0258]	5012 [R_{int} = 0.1005, R_{sigma} = 0.0912]
Data/restraints/parameters	13452/311/677	14047/87/658	5012/147/365
Goodness-of-fit on F^2	1.013	1.045	0.998
Final R indexes [I>=2σ (I)]	R_1 = 0.0517, wR_2 = 0.1099	R_1 = 0.0539, wR_2 = 0.1478	R_1 = 0.0694, wR_2 = 0.1921
Final R indexes [all data]	R_1 = 0.0972, wR_2 = 0.1284	R_1 = 0.0647, wR_2 = 0.1567	R_1 = 0.1136, wR_2 = 0.2279
Largest diff. peak/hole / e Å^{-3}	0.45/-0.47	0.70/-0.50	0.902/-0.508

5.2 Crystal Structure of [1_{Tipp}]$^+$ (R' = Me)

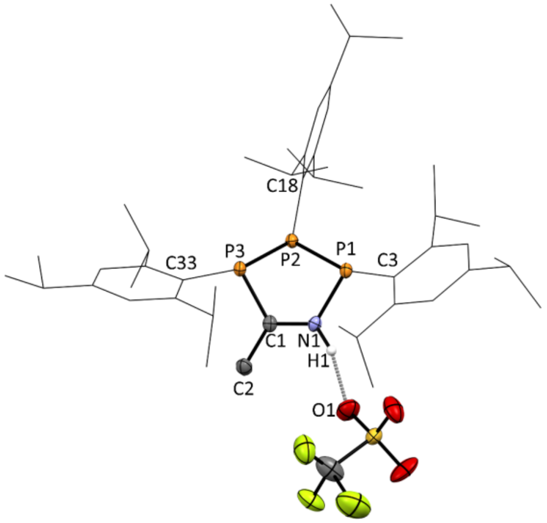

Figure S-117 Solid state molecular structure of **[1_{Tipp}]$^+$ (R' = Me)**. Thermal ellipsoids are drawn at the 50% probability level. Tipp substituents shown in wireframe for clarity, and all protons except H1 have been omitted for clarity. Selected bond lengths [Å] and angles [°]: P1-P2 2.233(1), P3-P2 2.226(1), P1-N1 1.787(3), P3-C1 1.810(4), C1-N1 1.300(5), C1-C2 1.496(5), N1-H1 0.95(5), O1-H1 1.79(5), P1-C3 1.835(4), P2-C18 1.844(4), P3-C33 1.79(5); P1-N1-C1 120.3(3), P3-C1-N1 118.5(3), P1-P2-P3 86.90(5), P2-P1-N1 90.7(1), P2-P3-C1 91.4(1), P2-P3-C33 113.3(1), C1-P3-C33 107.0(2), P2-P1-C3 100.3(1), N1-P1-C3 109.3(2), P1-P2-C18 99.6(1), P3-P2-C18 112.2(1).

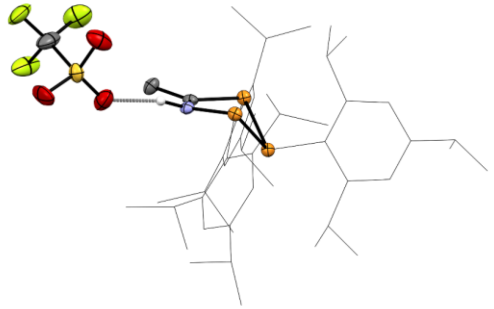

Figure S-118 Side view of the solid state molecular structure of **[1_{Tipp}]$^+$ (R' = Me)**. Thermal ellipsoids are drawn at the 50% probability level. Tipp substituents shown in wireframe for clarity, and all protons except H1 have been omitted for clarity.

5.3 Crystal Structure of [$\mathbf{1_{Tipp}}$]$^+$ (R' = Ph)

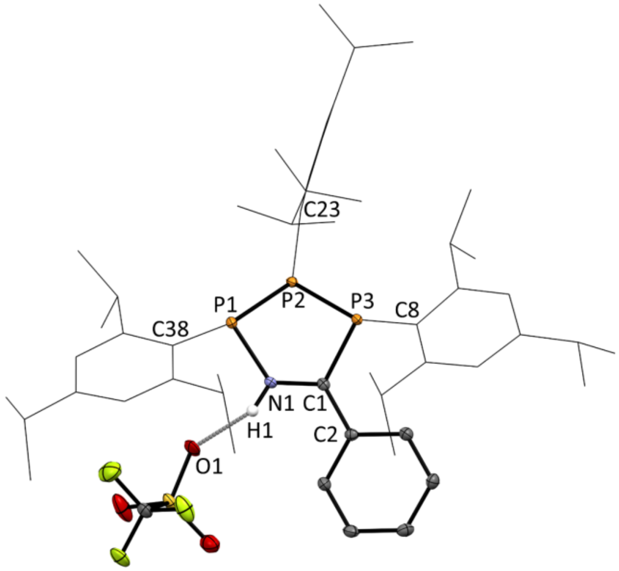

Figure S-119 Solid state molecular structure of **[1_{Tipp}]$^+$ (R' = Ph)**. Thermal ellipsoids are drawn at the 50% probability level. Tipp substituents shown in wireframe for clarity, and all protons except H1 have been omitted for clarity. Selected bond lengths [Å] and angles [°]: P1-P2 2.2458(6), P3-P2 2.2262(4), P1-N1 1.775(1), P3-C1 1.820(1), C1-N1 1.312(2), C1-C2 1.471(2), N1-H1 0.89(2), O1-H1 1.84(2), P1-C38 1.830(2), P2-C23 1.855(1), P3-C8 1.826(2); P1-N1-C1 125.0(1), P3-C1-N1 115.3(1), P1-P2-P3 88.30(2), P2-P1-N1 92.00(4), P2-P3-C1 95.01(5), P2-P3-C8 107.37(5), C1-P3-C8 111.19(7), P2-P1-C38 111.68(5), N1-P1-C38 101.43(6), P1-P2-C23 109.34(5), P3-P2-C23 98.82(5).

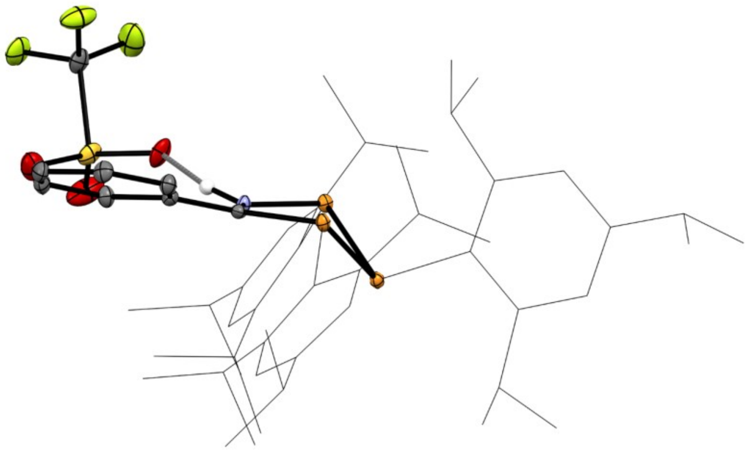

Figure S-120 Side view of the solid state molecular structure of **[1_{Tipp}]$^+$ (R' = Ph)**. Thermal ellipsoids are drawn at the 50% probability level. Tipp substituents shown in wireframe for clarity, and all protons except H1 have been omitted for clarity.

5.4 Crystal Structure of $[\mathbf{1}_{\mathrm{Tipp}}]^+$ (R' = *p*-MeOC$_6$H$_4$)

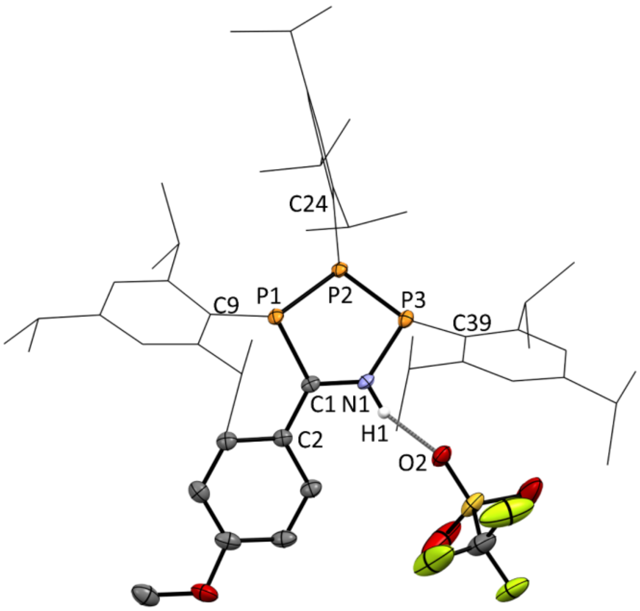

Figure S-121 Solid state molecular structure of **$[\mathbf{1}_{\mathrm{Tipp}}]^+$ (R' = *p*-MeOC$_6$H$_4$)**. Thermal ellipsoids are drawn at the 50% probability level. Tipp substituents shown in wireframe for clarity, and all protons except H1 have been omitted for clarity. Selected bond lengths [Å] and angles [°]: P3-P2 2.2435(8), P1-P2 2.226(1), P3-N1 1.773(2), P1-C1 1.824(2), C1-N1 1.319(4), C1-C2 1.451(3), N1-H1 0.86, P3-C39 1.836(3), P2-C24 1.853(2), P1-C9 1.830(3), H1-O2 1.978; P3-N1-C1 125.2(2),P1-C1-N 114.5(2), P2-P3-N1 91.42(7), P2-P1-C1 94.28(8), P1-P2-P3 87.97(3), P2-P3-C39 112.51(8), N1-P3-C39 100.4(1), P2-P1-C9 106.70(8), C1-P1-C9 111.0(1), P3-P2-C24 109.02(8), P1-P2-C24 100.04(8).

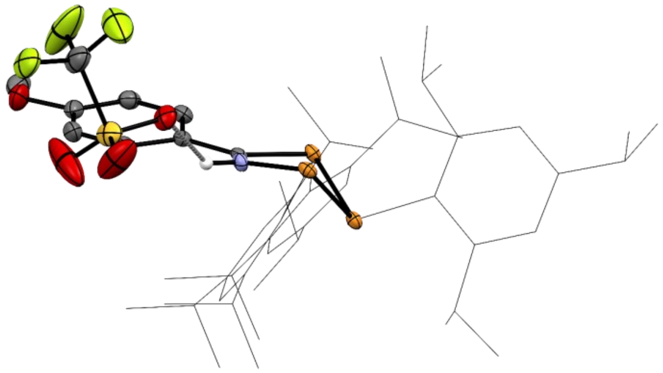

Figure S-122 Side view of the solid state molecular structure of **$[\mathbf{1}_{\mathrm{Tipp}}]^+$ (R' = *p*-MeOC$_6$H$_4$)**. Thermal ellipsoids are drawn at the 50% probability level. Tipp substituents shown in wireframe for clarity, and all protons except H1 have been omitted for clarity.

5.5 Crystal Structure of $[1_{Tipp}]^+$ (R' = *p*-$CF_3C_6H_4$)

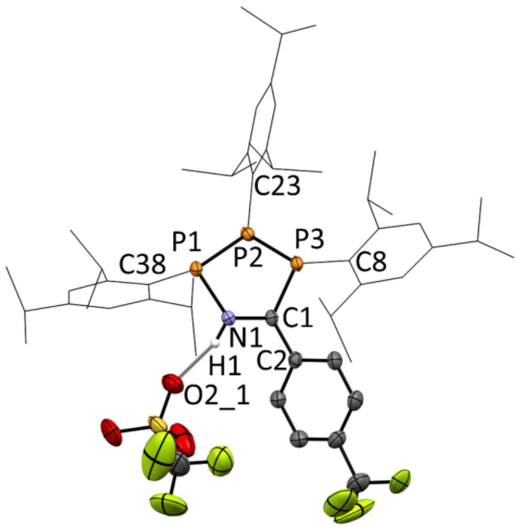

Figure S-123 Solid state molecular structure of $[\mathbf{1}_{Tipp}]^+$ **(R' = *p*-$CF_3C_6H_4$)**. Thermal ellipsoids are drawn at the 50% probability level. Tipp substituents shown in wireframe for clarity, and all protons except H1 have been omitted for clarity. Selected bond lengths [Å] and angles [°]: P1-P2 2.2557(7), P2-P3 2.2247(7), P1-N1 1.7767(16), P3-C1 1.8190(19), N1-C1 1.311(2), C1-C2 1.468(3), N1-H1 0.860, O2_1-H1 1.984, P1-C38 1.8295(19), P2-C23 1.8410(19), P3-C8 1.8318(19); P1-N1-C1 123.15(13), P3-C1-N1 114.76(14), P1-P2-P3 85.76(2), P2-P1-N1 89.70(5), P2-P3-C1 92.35(6), P2-P3-C8 104.12(7), C1-P3-C8 111.56(9), P2-P1-C38 110.61(7), N1-P1-C38 101.40(8), P3-P2-C23 102.11(6), P1-P2-C23 114.04(6).

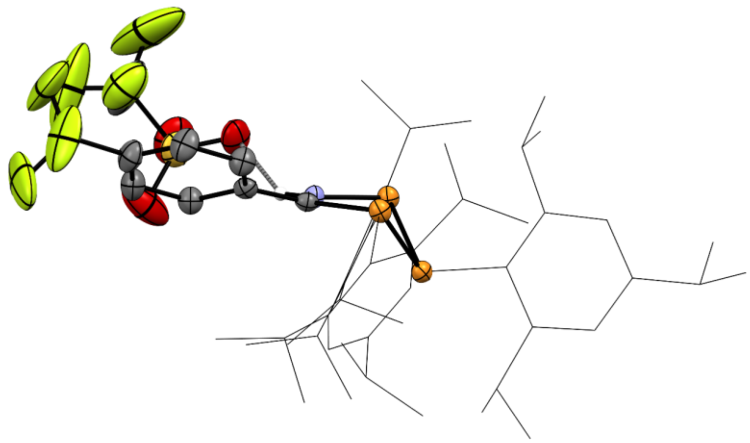

Figure S-124 Side view of the solid state molecular structure of $[\mathbf{1}_{Tipp}]^+$ **(R' = *p*-$CF_3C_6H_4$)**. Thermal ellipsoids are drawn at the 50% probability level. Tipp substituents shown in wireframe for clarity, and all protons except H1 have been omitted for clarity.

5.6 Crystal Structure of [1_{Dipp}]$^+$ (R' = Me)

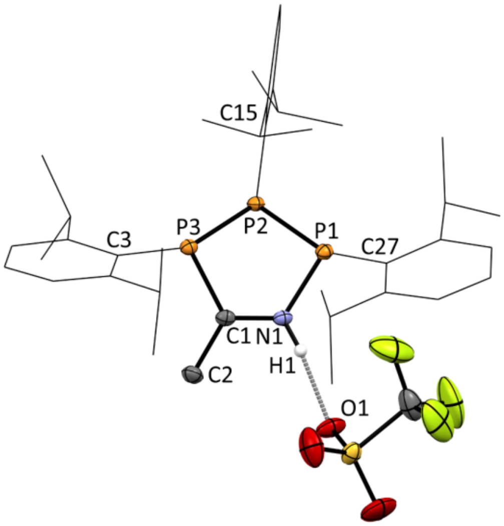

Figure S-125 Solid state molecular structure of **[1_{Dipp}]$^+$ (R' = Me)**. Thermal ellipsoids are drawn at the 50% probability level. Dipp substituents shown in wireframe for clarity, and all protons except H1 have been omitted for clarity. Selected bond lengths [Å] and angles [°]: P1-P2 2.2387(6), P2-P3 2.2360(5), P1-N1 1.781(1), P3- C1 1.816(1), C1-N1 1.299(2), C1-C2 1.491(2), N1-H1 0.85(2), O1-H1 1.89(2), P1-C27 1.831(1), P2-C15 1.848(1), P3-C3 1.836(1); P1-N1-C1 121.58(9), P3-C1-N1 117.8(1), P1-P2-P3 86.98(2), P2-P1-N1 90.53(4), P2-P3-C1 91.13(4), N1-P1-C27 104.52(6), P2-P1-C27 105.82(4), C1-P3-C3 105.57(6), P2-P3-C3 111.54(4), P1-P2-C15 103.96(5), P3-P2-C15 110.46(5).

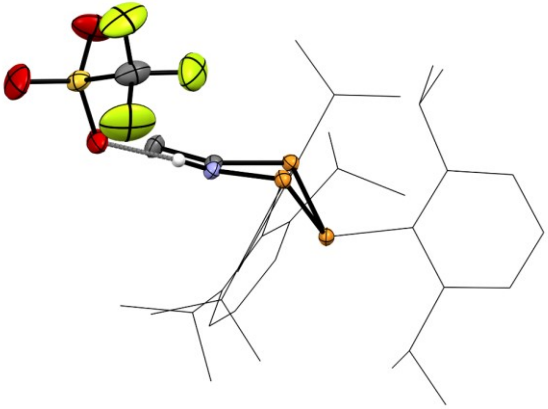

Figure S-126 Side view of the solid state molecular structure of **[1_{Dipp}]$^+$ (R' = Me)**. Thermal ellipsoids are drawn at the 50% probability level. Dipp substituents shown in wireframe for clarity, and all protons except H1 have been omitted for clarity.

5.7 Crystal Structure of $[1_{Dipp}]^+$ (R' = Ph)

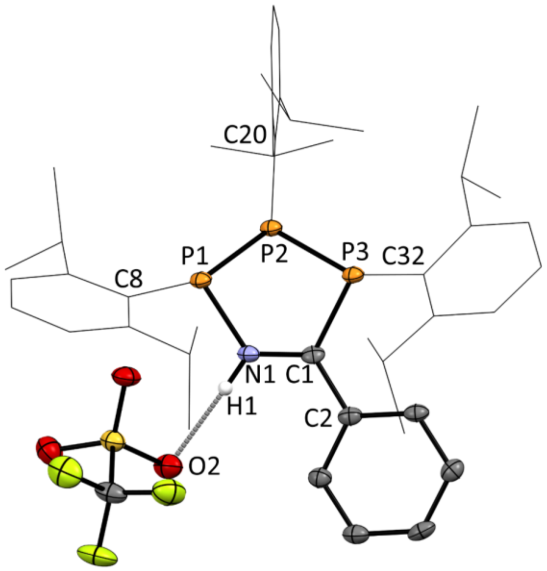

Figure S-127 Solid state molecular structure of **$[1_{Dipp}]^+$ (R' = Ph)**. Thermal ellipsoids are drawn at the 50% probability level. Dipp substituents shown in wireframe for clarity, and all protons except H1 have been omitted for clarity. Selected bond lengths [Å] and angles [°]: P1-P2 2.243(1), P3-P2 2.212(1), P1-N1 1.790(2), P3-C1 1.822(3), C1-N1 1.312(4), C1-C2 1.473(3), N1-H1 0.87(4), P1-C8 1.838(3), P2-C20 1.854(3), P3-C32 1.842(4), O2-H1 2.04(4);P1-N1-C1 124.0(2), P3-C1-N1 115.8(2), P2-P1-N1 91.23(8), P2-P3-C1 93.98(9), P3-P2-P1 88.53(4), P2-P1-C8 104.6(1), N1-P1-C8 104.7(1), P2-P3-C32 105.80(9), C1-P3-C32 112.4(1), P1-P2-C20 107.61(9), P3-P2-C20 103.55(9).

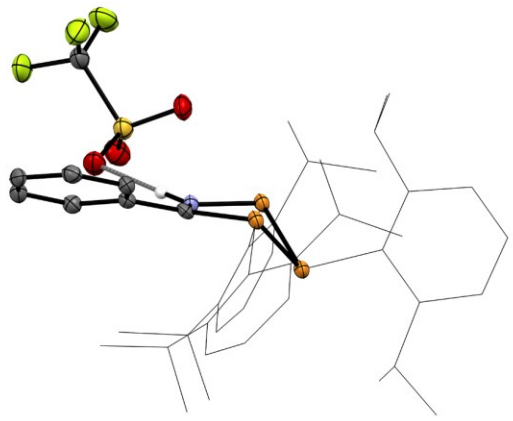

Figure S-128 Side view of the solid state molecular structure of **$[1_{Dipp}]^+$ (R' = Ph)**. Thermal ellipsoids are drawn at the 50% probability level. Dipp substituents shown in wireframe for clarity, and all protons except H1 have been omitted for clarity.

5.8 Crystal Structure of [**1**$_{\mathbf{Mes}}$]$^{+}$ (R' = Me)

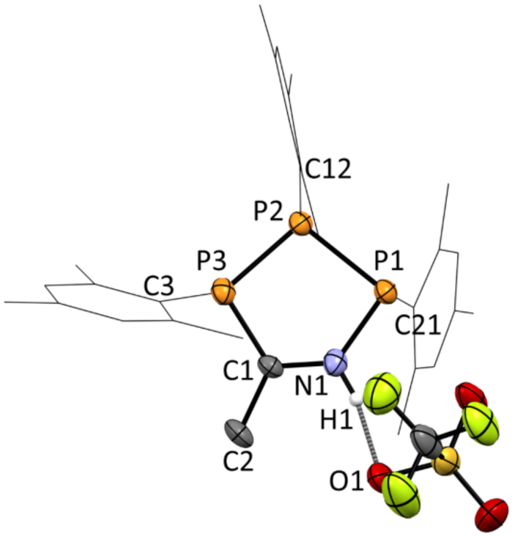

Figure S-129 Solid state molecular structure of **[1$_{\mathbf{Mes}}$]$^{+}$ (R' = Me)**. Thermal ellipsoids are drawn at the 50% probability level. Mes substituents shown in wireframe for clarity, and all protons except H1 have been omitted for clarity. Selected bond lengths [Å] and angles [°]: P1-P2 2.237(3), P2-P3 2.192(3), P1-N1 1.778(8), P3-C1 1.782(8), C1-N1 1.309(9), C1-C2 1.50(1), N1-H1 0.88, O1-H1 1.993, P1-C21 1.835(7), P2-C12 1.849(7), P3-C3 1.825(6); P1-N1-C1 127.4(6), P3-C1-N1 118.8(6), P1-P2-P3 95.5(1), P2-P1-N1 95.4(2), P2-P3-C1 98.6(3), N1-P1-C21 102.0(3), P2-P1-C21 99.0(3), C1-P3-C3 106.3(4), P2-P3-C3 109.7(3), P1-P2-C12 106.3(3), P3-P2-C12 100.5(3).

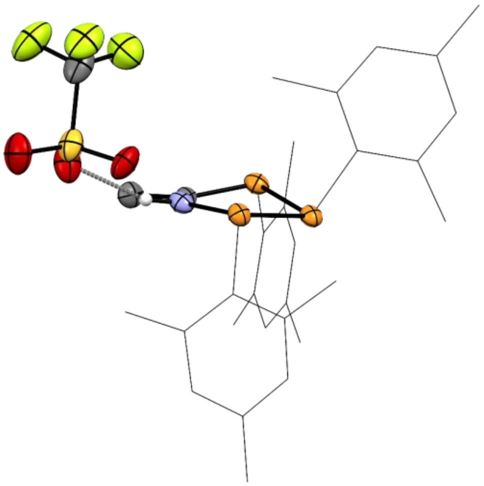

Figure S-130 Side view of the solid state molecular structure of **[1$_{\mathbf{Mes}}$]$^{+}$ (R' = Me)**. Thermal ellipsoids are drawn at the 50% probability level. Mes substituents shown in wireframe for clarity, and all protons except H1 have been omitted for clarity.

5.9 Crystal Structure of $[1_{Mes}]^+$ (R' = Ph)

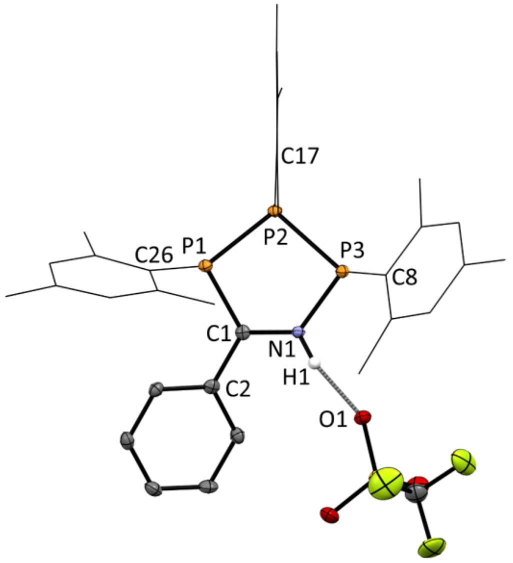

Figure S-131 Solid state molecular structure of **$[1_{Mes}]^+$ (R' = Ph)**. Thermal ellipsoids are drawn at the 50% probability level. Mes substituents shown in wireframe for clarity, and all protons except H1 have been omitted for clarity. Selected bond lengths [Å] and angles [°]:P3-P2 2.238(1), P1-P2 2.206(1), P3-N1 1.772(2), P1-C1 1.804(2), C1-N1 1.309(4), C1-C2 1.475(3), N1-H1 0.87(3), P3-C8 1.830(3), P2-C17 1.844(2), P1-C26 1.823(3), H1-O1 1.92(3); P3-N1-C1 126.3(2), P1-C1-N1 118.8(2), P2-P3-N1 95.34(8), P2-P1-C1 97.42(9), P1-P2-P3 93.42(4), P2-P3-C8 100.68(8), N1-P3-C8 106.6(1), P2-P1-C26 110.44(9), C1-P1-C26 105.1(1), P3-P2-C17 104.64(8), P1-P2-C17 103.39(8).

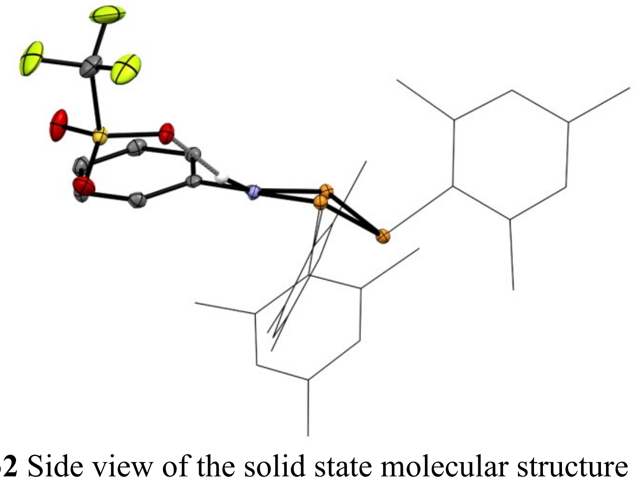

Figure S-132 Side view of the solid state molecular structure of **$[1_{Mes}]^+$ (R' = Ph)**. Thermal ellipsoids are drawn at the 50% probability level. Mes substituents shown in wireframe for clarity, and all protons except H1 have been omitted for clarity.

5.10 Crystal Structure of [1_{tBu}]$^+$ (R' = Ph)

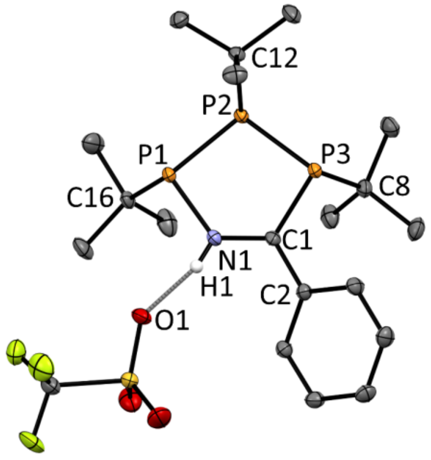

Figure S-133 Solid state molecular structure of **[1_{tBu}] (R' = Ph)**. Thermal ellipsoids are drawn at the 50% probability level, and all protons except H1 and DCM protons have been omitted for clarity. Selected bond lengths [Å] and angles [°]: P2-P3 2.2086(6), P1-P3 2.1958(6), P2-N1 1.768(2), P1-C1 1.817(2), C1-N1 1.311(2), C1-C4 1.469(2), N1-H1 0.87(3), P2-C5 1.870(2), P3-C2 1.891(2), P1-C6 1.893(2), H1-O1 1.88(3); P2-N1-C1 125.8(1), P1-C1-N1 120.0(1), P3-P2-N1 97.35(5), P3-P1-C1 98.65(6), P1-P3-P2 95.30(2), P3-P2-C5 105.31(6), N1-P2-C5 102.37(8), P3-P1-C6 105.24(6), C1-P1-C6 106.27(8), P2-P3-C2 99.62(6), P1-P3-C2 101.07(6).

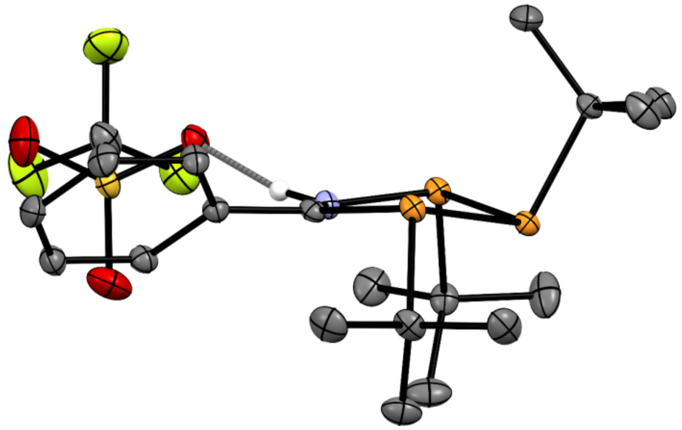

Figure S-134 Side view of the solid state molecular structure of **[1_{tBu}]$^+$ (R' = Ph)**. Thermal ellipsoids are drawn at the 50% probability level, and all protons except H1 and DCM protons have been omitted for clarity.

5.11 Crystal Structure of $\mathbf{2_{Tipp}}$ (R' = Me)

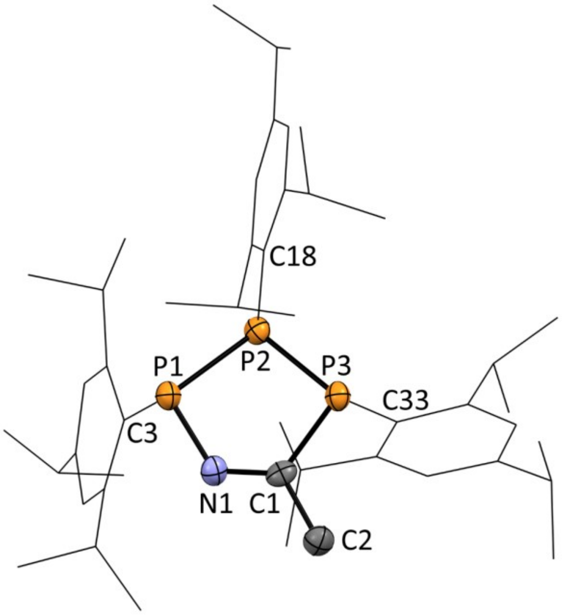

Figure S-135 Solid state molecular structure of $\mathbf{2_{Tipp}}$ **(R' = Me)**. Thermal ellipsoids are drawn at the 50% probability level. Tipp substituents shown in wireframe for clarity, and protons have been omitted for clarity. Selected bond lengths [Å] and angles [°]: P1-P2 2.265(2), P2-P3 2.185(1), P1-N1 1.715(4), P3-C1 1.850(4), C1-N1 1.289(6), C1-C2 1.502(6), P1-C3 1.866(4), P2-C18 1.849(5), P3-C33 1.848(4); P1-N1-C1 123.3(3), P3-C1-N1 123.2(3), P1-P2-P3 93.62(6), P2-P1-N1 98.4(1), P2-P3-C1 96.2(1), N1-P1-C3 100.9(2), P2-P1-C3 99.5(1), C1-P3-C33 104.6(2), P2-P3-C33 106.0(1), P1-P2-C18 107.7(1), P3-P2-C18 101.4(1).

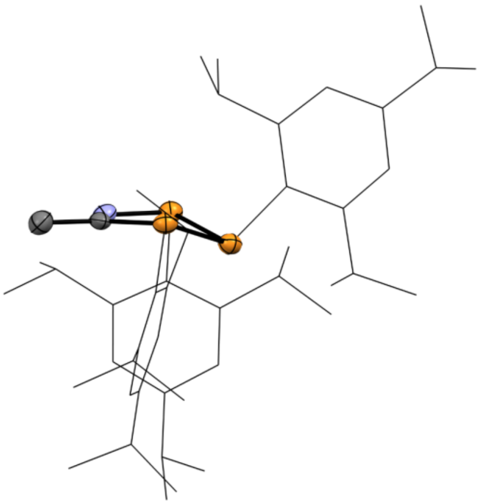

Figure S-136 Side view of the solid state molecular structure of $\mathbf{2_{Tipp}}$ **(R' = Me)**. Thermal ellipsoids are drawn at the 50% probability level. Tipp substituents shown in wireframe for clarity, and protons have been omitted for clarity.

5.12 Crystal Structure of $\mathbf{2_{Tipp}}$ (R' = Ph)

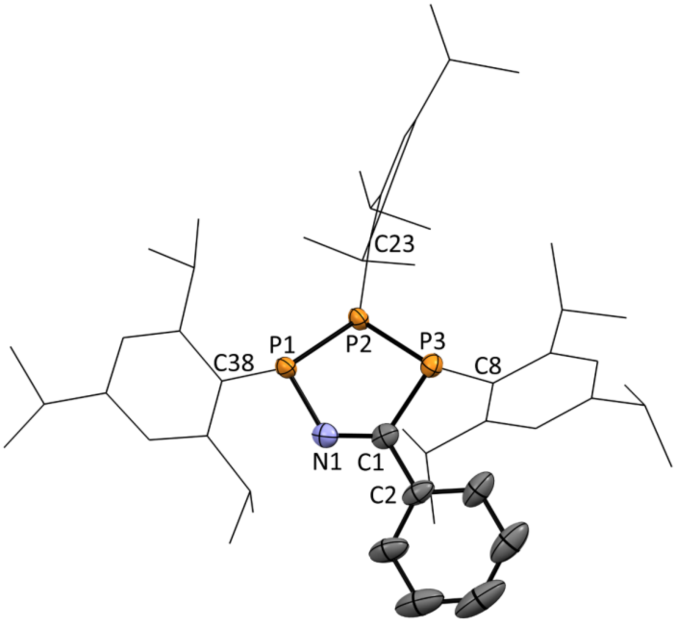

Figure S-137 Solid state molecular structure of $\mathbf{2_{Tipp}}$ **(R' = Ph)**. Thermal ellipsoids are drawn at the 50% probability level. Tipp substituents shown in wireframe for clarity, and protons have been omitted for clarity. Selected bond lengths [Å] and angles [°]: P1-P2 2.256(1), P3-P2 2.201(1), P1-N1 1.708(3), P3-C1 1.853(4), C1-N1 1.294(5), C1-C2 1.489(5), P1-C38 1.842(4), P2-C23 1.859(4), P3-C8 1.857(4); P1-N1-C1 120.7(3), P3-C1-N1 121.9(3), P2-P1-N1 97.1(1), P2-P3-C1 94.4(1), P3-P2-P1 89.90(5), P2-P1-C38 104.3(1), N1-P1-C38 108.7(2), P2-P3-C8 104.8(1), C1-P3-C8 109.4(2), P1-P2-C23 110.1(1), P3-P2-C23 98.7(1).

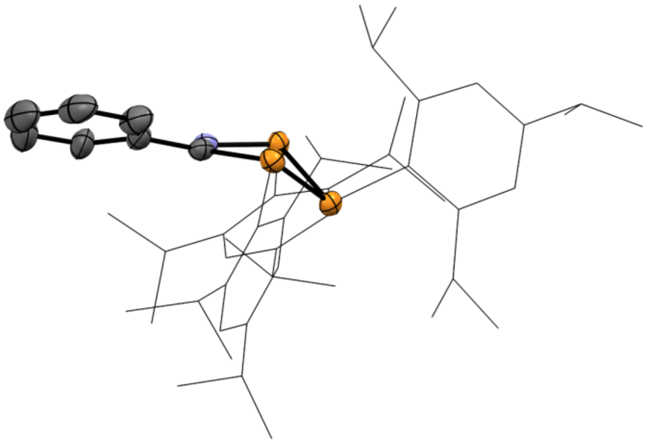

Figure S-138 Side view of the solid state molecular structure of $\mathbf{2_{Tipp}}$ **(R' = Ph)**. Thermal ellipsoids are drawn at the 50% probability level. Tipp substituents shown in wireframe for clarity, and protons have been omitted for clarity.

5.13 Crystal Structure of 2_{Dipp} (R' = Me)

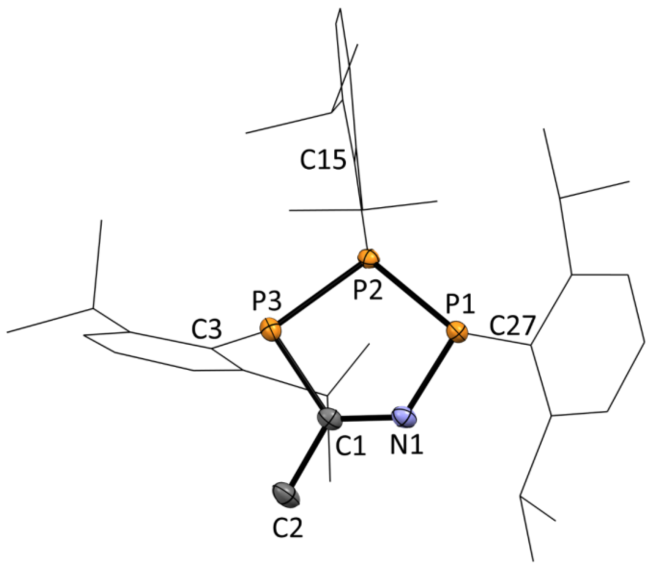

Figure S-139 Solid state molecular structure of **2_{Dipp} (R' = Me)**. Thermal ellipsoids are drawn at the 50% probability level. Dipp substituents shown in wireframe for clarity, and protons have been omitted for clarity. Selected bond lengths [Å] and angles [°]: P1-P2 2.2682(5), P2-P3 2.1947(5), P1-N1 1.714(1), P3-C1 1.846(1), C1-N1 1.280(2), C1-C2 1.500(2), P1-C27 1.858(1), P2-C15 1.852(1), P3-C3 1.852(2);P1-N1-C1 122.73(9), P3-C1-N1 122.7(1), P1-P2-P3 91.87(2), P2-P1-N1 97.10(4), P2-P3-C1 94.69(4), N1-P1-C27 105.39(5), P2-P1-C27 98.78(4), C1-P3-C3 105.02(6), P2-P3-C3 103.55(4), P1-P2-C15 110.36(4), P3-P2-C15 103.73(4).

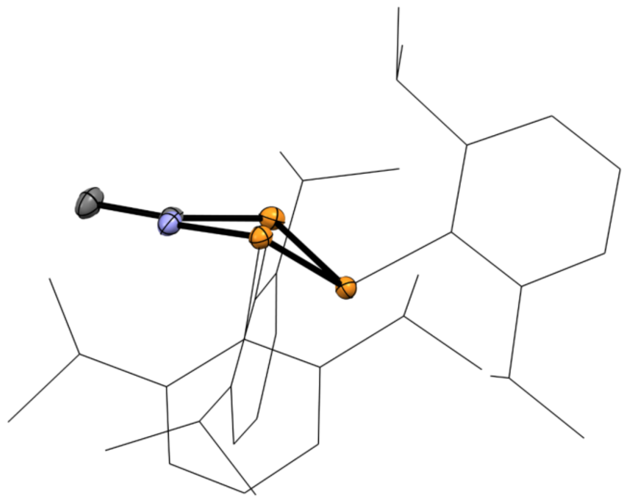

Figure S-140 Side view of the solid state molecular structure of **2_{Dipp} (R' = Me)**. Thermal ellipsoids are drawn at the 50% probability level. Dipp substituents shown in wireframe for clarity, and protons have been omitted for clarity.

5.14 Crystal Structure of 2_{Dipp} (R’ = Ph)

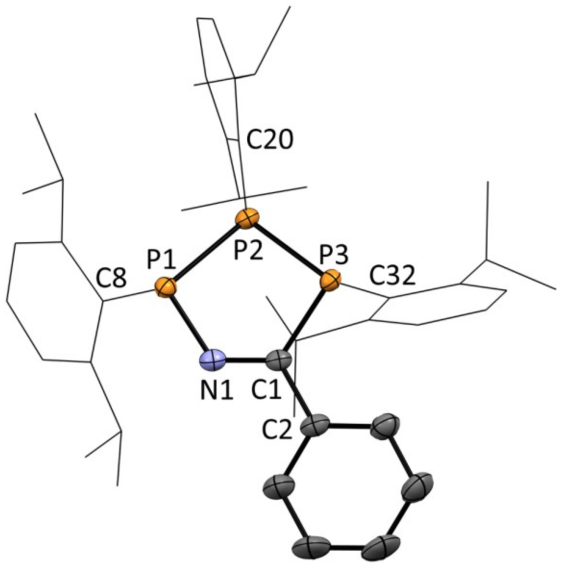

Figure S-141 Solid state molecular structure of **2_{Dipp} (R’ = Ph)**. Thermal ellipsoids are drawn at the 50% probability level. Dipp substituents shown in wireframe for clarity, and protons have been omitted for clarity. Selected bond lengths [Å] and angles [°]: P1-P2 2.2725(6), P3-P2 2.1972(5), P1-N1 1.697(1), P3-C1 1.870(2), C1-N1 1.282(2), C1-C2 1.498(2), P1-C8 1.864(2), P2-C20 1.858(1), P3-C32 1.859(2); P1-N1-C1 123.0(1), P3-C1-N1 122.5(1), P2-P3-C1 96.14(5), P2-P1-N1 98.79(5), P1-P2-P3 91.91(2), P2-P1-C8 103.97(5), N1-P1-C8 107.82(7), P2-P3-C32 104.97(5), C1-P3-C32 106.23(7), P1-P2-C20 100.97(5), P3-P2-C20 106.94(5).

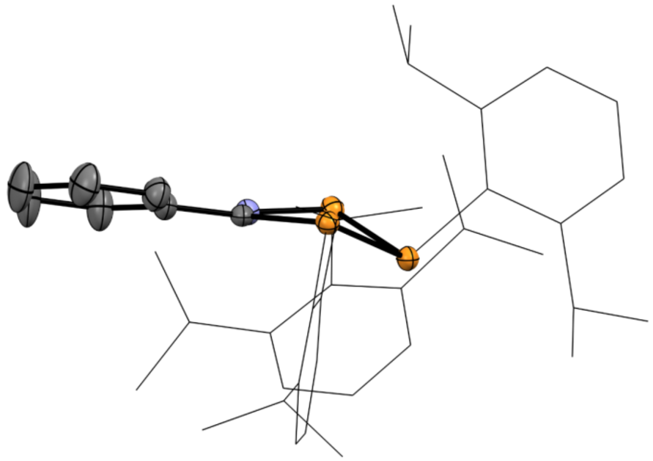

Figure S-142 Side view of the solid state molecular structure of **2_{Dipp} (R’ = Ph)**. Thermal ellipsoids are drawn at the 50% probability level. Dipp substituents shown in wireframe for clarity, and protons have been omitted for clarity.

5.15 Crystal Structure of $\mathbf{2_{Mes}}$ (R' = Me)

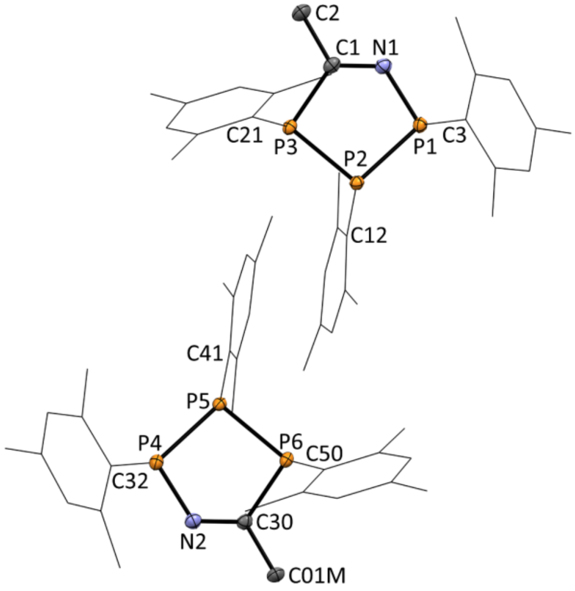

Figure S-143 Solid state molecular structure of $\mathbf{2_{Mes}}$ **(R' = Me)**. Thermal ellipsoids are drawn at the 50% probability level. Mes substituents shown in wireframe for clarity, and protons have been omitted for clarity. Selected bond lengths [Å] and angles [°]: P1-P2 2.266(2), P3-P2 2.193(1), P1-N1 1.705(3), P3-C1 1.855(4), C1-N1 1.279(5), C1-C2 1.506(5), P1-C3 1.849(3), P2-C12 1.850(3), P3-C21 1.843(4), P4-P5 2.271(2), P6-P5 2.192(1), P4-N2 1.706(4), P6-C30 1.853(4), C30-N2 1.280(5), C30-C01M 1.512(5), P4-C32 1.842(5), P5-C41 1.853(3), P6-C50 1.841(4); P1-N1-C1 122.7(3), P3-C1-N1 123.6(3), P2-P1-N1 99.8(1), P2-P3-C1 96.7(1), P3-P2-P1 92.78(5), P2-P1-C3 100.0(1), N1-P1-C3 105.0(2), P2-P3-C21 106.6(1), C1-P3-C21 105.2(2), P1-P2-C12 106.5(1), P3-P2-C12 101.4(1), P4-N2-C30 122.9(3), P6-C30-N2 123.4(3), P5-P4-N2 99.5(1), P5-P6-C30 96.8(1), P6-P5-P4 92.62(5), P5-P4-C32 99.0(1), N2-P4-C32 105.3(2), P5-P6-C50 106.5(1), C30-P6-C50 104.6(2), P4-P5-C41 107.0(1), P6-P5-C41 101.5(1).

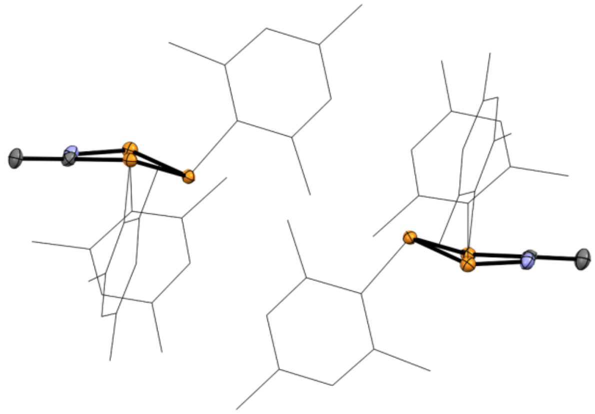

Figure S-144 Side view of the solid state molecular structure of $\mathbf{2_{Mes}}$ **(R' = Me)**. Thermal ellipsoids are drawn at the 50% probability level. Mes substituents shown in wireframe for clarity, and protons have been omitted for clarity.

5.16 Crystal Structure of $\mathbf{2_{Mes}}$ (R' = Ph)

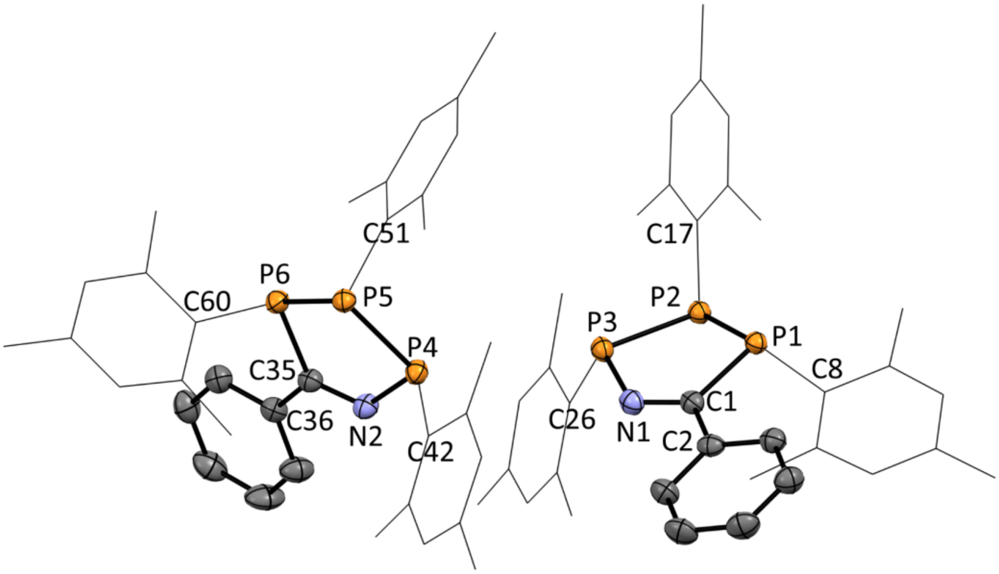

Figure S-145 Solid state molecular structure of **$\mathbf{2_{Mes}}$ (R' = Ph)**. Thermal ellipsoids are drawn at the 50% probability level. Mes substituents shown in wireframe for clarity, and protons have been omitted for clarity. Selected bond lengths [Å] and angles [°]: P1-P2 2.1862(8), P2-P3 2.2496(8), P4-P5 2.256(1), P5-P6 2.1907(8), P3-N1 1.693(2), P4-N2 1.693(2), P1-C1 1.869(2), P6-C35 1.869(3), C1-N1 1.280(2), C35-N2 1.281(3), C1-C2 1.487(3), C35-C36 1.489(3), P1-C8 1.844(3), P2-C17 1.846(2), P3-C26 1.836(2), P4-C42 1.837(2), P5-C51 1.848(2), P6-C60 1.842(3); P1-C1-N1 121.9(2), P3-N1-C1 123.9(2), P1-P2-P3 92.98(3), P2-P1-C1 96.62(7), P2-P3-N1 99.50(7), P4-N2-C35 123.8(2), P6-C35-N2 122.0(2), P4-P5-P6 92.69(3), P5-P4-N2 99.43(7), P5-P6-C35 96.71(7), C1-P1-C8 108.3(1), P2-P1-C8 102.58(7), N1-P3-C26 105.4(1), P2-P3-C26 102.99(8), P1-P2-C17 101.69(8), P3-P2-C17 105.24(8), N2-P4-C42 106.4(1), P5-P4-C42 102.70(8), C35-P6-C60 106.9(1), P5-P6-C60 103.01(8), P4-P5-C51 103.15(8), P6-P5-C51 104.26(8).

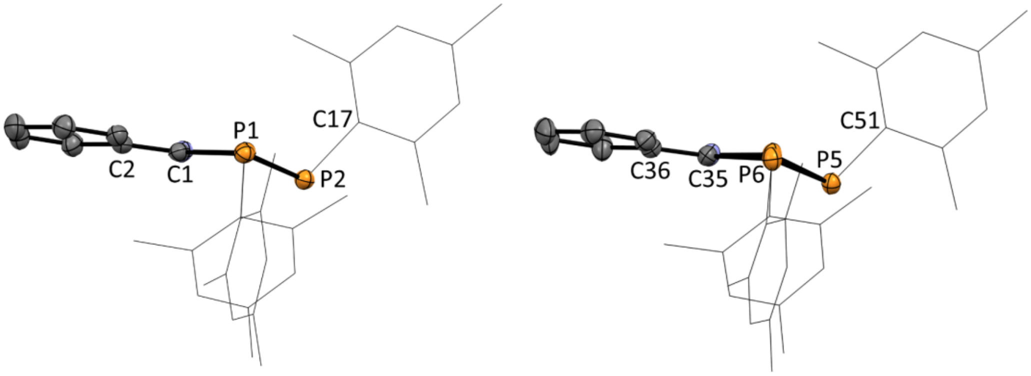

Figure S-146 Side view of each individual molecule in the asymmetric unit of **$\mathbf{2_{Mes}}$ (R' = Ph)**. Thermal ellipsoids are drawn at the 50% probability level. Mes substituents shown in wireframe for clarity, and protons have been omitted for clarity.

5.17 Crystal Structure of $\mathbf{2}_{t\mathrm{Bu}}$ (R' = Ph)

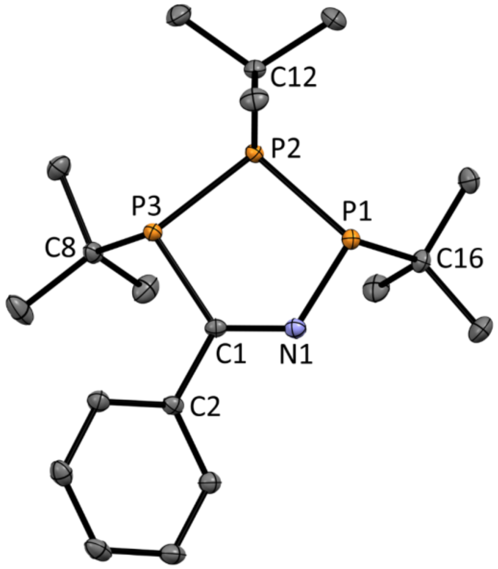

Figure S-147 Solid state molecular structure of **$\mathbf{2}_{t\mathrm{Bu}}$ (R' = Ph)**. Thermal ellipsoids are drawn at the 50% probability level, and all protons have been omitted for clarity. Selected bond lengths [Å] and angles [°]: P1-P2 2.2322(3), P3-P2 2.1935(3), P1-N1 1.7043(8), P3-C1 1.8732(8), C1-N1 1.288(1), C1-C2 1.495(1), P1-C16 1.873(1), P2-C12 1.897(1), P3-C8 1.896(1); P1-N1-C1 121.68(7), P3-C1-N1 122.90(7), P2-P1-N1 100.37(3), P2-P3-C1 96.65(3), P3-P2-P1 93.01(1), P2-P1-C16 105.65(3), N1-P1-C16 102.19(4), P2-P3-C8 102.52(3), C1-P3-C8 104.48(4), P1-P2-C12 101.49(3), P3-P2-C12 101.57(3).

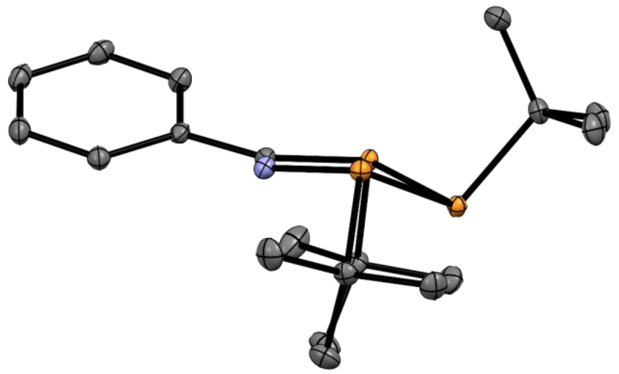

Figure S-148 Side view of the solid state molecular structure of **$\mathbf{2}_{t\mathrm{Bu}}$ (R' = Ph)**. Thermal ellipsoids are drawn at the 50% probability level, and protons have been omitted for clarity.

5.18 Crystal Structure of $P_6Mes_6{\cdot}Et_2O$

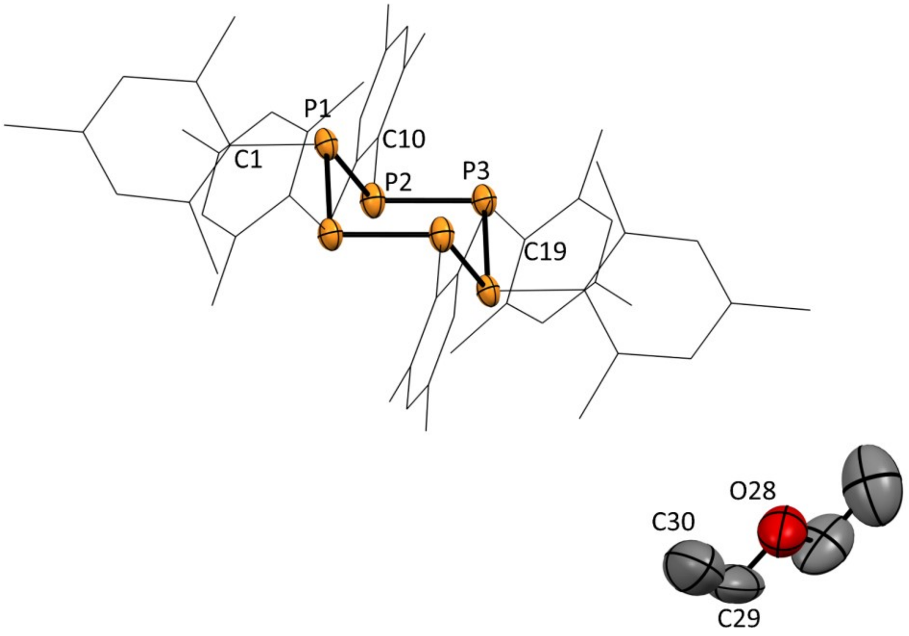

Figure S-149 Solid state molecular structure of $\mathbf{P_6Mes_6{\cdot}Et_2O}$. Thermal ellipsoids are drawn at the 50% probability level, and all protons have been omitted for clarity. Selected bond lengths [Å] and angles [°]: P1-P2 = 2.239(2), P2-P3 = 2.237(1), P3-P1 = 2.241(2), P1-C1 = 1.867(4), P2-C10 = 1.82(2), P3-C19 = 1.858(4), C30-C29 = 1.50(2), C29-O28 = 1.40; P3-P1-P2 = 96.76(5), P1-P2-P3 = 97.71(5), P2-P3-P1 = 103.50(6), plane(P1-P2-P3)-plane(P1-P3-P1-P3) = 74.48.

5.19 Comparison of P_N and P_C distances

	**[1$_{tBu}$]$^+$ (R' = Me)**	**[1$_{Mes}$]$^+$ (R' = Me)**		**[1$_{Dipp}$]$^+$ (R' = Me)**	**[1$_{Tipp}$]$^+$ (R' = Me)**		
P_N-P_C Distance (Å)	3.237(1)	3.278(3)		3.0797(5)	3.067(1)		
% change	NA	1.27		-5.11	-5.54		
	**2$_{tBu}$ (R' = Me)**	**2$_{Mes}$ (R' = Me) A**	**2$_{Mes}$ (R' = Me) B**	**2$_{Dipp}$ (R' = Me)**	**2$_{Tipp}$ (R' = Me)**		
P_N-P_C Distance (Å)	3.190(1)	3.227(1)	3.228(1)	3.2072(5)	3.245(2)		
% change	NA	1.16	1.19	0.54	1.72		
	**[1$_{tBu}$]$^+$ (R' = Ph)**	**[1$_{Mes}$]$^+$ (R' = Ph)**		**[1$_{Dipp}$]$^+$ (R' = Ph)**	**[1$_{Tipp}$]$^+$ (R' = Ph)**	**[1$_{Tipp}$]$^+$ (R' = *p*-MeOC$_6$H$_4$)**	**[1$_{Tipp}$]$^+$ (R' = *p*-CF$_3$C$_6$H$_4$)**
P_N-P_C Distance (Å)	3.2549(6)	3.235(1)		3.110(1)	3.1149(7)	3.104(1)	3.0489(6)
% change	NA	0.06		-4.66	-4.49	-4.86	-6.76
	**2$_{tBu}$ (R' = Ph)**	**2$_{Mes}$ (R' = Ph) A**	**2$_{Mes}$ (R' = Ph) B**	**2$_{Dipp}$ (R' = Ph)**	**2$_{Tipp}$ (R' = Ph)**		
P_N-P_C Distance (Å)	3.2107(4)	3.2171(7)	3.2178(9)	3.2131(5)	3.149(1)		
% change	NA	0.11	0.22	0.07	-1.96		

6. Computational Details

6.1 General Remarks

Computations were carried out using Gaussian09[10] or Gaussian16[11] and the standalone version of NBO 6.0.[12]

Structure optimizations employed the GGA DFT functional BP86[13] in conjunction with Grimme's dispersion correction D3(BJ)[14] and the def2-SVP basis set[15] (notation BP86-D3/def2-SVP). All structures were fully optimized and confirmed as minima by frequency analyses. The vertical excitation energies of the first ten singlet and triplet states have been predicted by TD-DFT computations using the B3LYP[16] functional as well as the cc-pVTZ[17] basis set using the respective BP86-D3/def2-SVP optimized gas-phase S^0 geometry. TD-DFT calculations were carried either in the gas phase or using Truhlar's continuum solvation model SMD for CH_2Cl_2 (scrf=smd,dichloromethane).[18] For the visualization of the charge density difference between the ground state and selected excited states we used MultiWfn 3.6 employing Gaussian16 formatted checkpoint files.[19] For the visualization of 3D-quantum chemical results we used GaussView6.1.1.[20] In addition to the electronic supporting information we provide a multi-structure xyz-file including all calculated molecules. For a better understanding and a more intuitive view of the calculated 3D structures, we strongly recommend using this file e.g. with the free program MERCURY.[21] The overlays of the calculated and molecular structures determined by SC-XRD experiments were visualized using VMD.[22]

Please note that all computations were carried out for single, isolated molecules in the gas phase (ideal gas approximation). There may well be significant differences between gas phase and condensed phase.

6.2 Summary of Calculated Data

Table S-4. Summary of calculated data, including electronic energies and values for H_{tot} and G_{tot}. All calculations were carried out on the BP86-D3/def2-SVP level of theory.

Compound	NIMAG	HF	ZPE	H_{tot} [a.u.]	$G°_{tot}$ [a.u.]
$[\mathbf{1}_{Tipp}]^+$ (R' = Me)	0	-3873.49838	683.66705	-3872.33616	-3872.51881
$[\mathbf{1}_{Tipp}]^+$ (R' = Ph)	0	-4065.12643	716.30174	-4063.90892	-4064.09967
$[\mathbf{1}_{Tipp}]^+$ (R' = p-$MeOC_6H_4$)	0	-4179.57680	736.16803	-4178.32498	-4178.52147
$[\mathbf{1}_{Tipp}]^+$ (R' = p-$CF_3C_6H_4$)	0	-4401.92343	718.91313	-4400.69792	-4400.89935
$[\mathbf{1}_{Dipp}]^+$ (R' = Me)	0	-3519.87818	529.90627	-3518.974501	-3519.12878
$[\mathbf{1}_{Dipp}]^+$ (R' = Ph)	0	-3711.51196	563.18492	-3710.55224	-3710.71122
$[\mathbf{1}_{Mes}]^+$ (R' = Ph)		-3357.92383	405.81360	-3357.22576	-3357.36557
$[\mathbf{1}_{tBu}]^+$ (R' = Me)	0	-2591.41525	278.26302	-2590.93615	-2591.03779
$[\mathbf{1}_{tBu}]^+$ (R' = Ph)	0	-2783.04469	311.20849	-2782.50999	-2782.61844
$\mathbf{2}_{Tipp}$ (R' = Me)	0	-2911.91540	658.96099	-2910.80152	-2910.96518
$\mathbf{2}_{Tipp}$ (R' = Ph)	0	-3103.54735	691.84108	-3102.37813	-3102.54813
$\mathbf{2}_{tBu}$ (R' = Me)	0	-1629.84352	254.30348	-1629.41175	-1629.49031
$\mathbf{2}_{tBu}$ (R' = Ph)	0	-1821.46914	287.12662	-1820.98198	-1821.06857

Table S-5. Selected computed and experimental bond lengths and angles of **[1$_{Tipp}$]$^+$ (R’ = Me)**.

**Bond**	**BP86**	**Experiment**	Optimized (red) and X-Ray (blue)
**P_N−P_P**	2.296	2.234	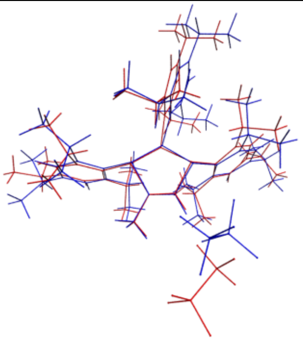
**P_P−P_C**	2.259	2.226	
**P_C−C**	1.832	1.810	
**C−N**	1.308	1.300	
**N−P_N**	1.808	1.788	
**P_N−P_P−P_C**	88.06	86.90	
**P_C−C−N**	116.74	118.5	
**C−N−P_N**	124.68	120.3	
**N−P_N−P_P**	89.95	90.66	
**P_P−P_C−C**	92.22	91.42	

Table S-6. Selected computed and experimental bond lengths and angles of **[1$_{Tipp}$]$^+$ (R’ = Ph)**.

**Bond**	**BP86**	**Experiment**	Optimized (red) and X-Ray (blue)
**P_N−P_P**	2.294	2.246	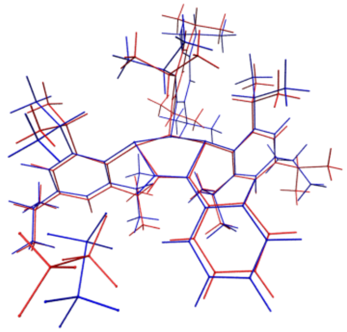
**P_P−P_C**	2.252	2.226	
**P_C−C**	1.842	1.820	
**C−N**	1.318	1.312	
**N−P_N**	1.798	1.775	
**P_N−P_P−P_C**	85.88	88.30	
**P_C−C−N**	114.20	115.28	
**C−N−P_N**	124.46	125.04	
**N−P_N−P_P**	89.37	92.00	
**P_P−P_C−C**	92.07	95.01	

Table S-7. Selected computed and experimental bond lengths and angles of **[1$_{Dipp}$]$^+$ (R’ = Me)**.

**Bond**	**BP86**	**Experiment**	Optimized (red) and X-Ray (blue)
**P_N−P_P**	2.277	2.239	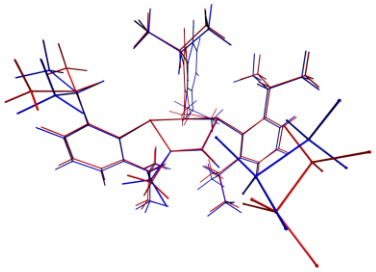
**P_P−P_C**	2.269	2.236	
**P_C−C**	1.836	1.816	
**C−N**	1.310	1.299	
**N−P_N**	1.783	1.782	
**P_N−P_P−P_C**	86.08	86.98	
**P_C−C−N**	118.31	117.76	
**C−N−P_N**	120.91	121.59	
**N−P_N−P_P**	91.87	90.53	
**P_P−P_C−C**	92.68	91.13	

Table S-8. Selected computed and experimental bond lengths and angles of **$[1_{Dipp}]^+$ (R' = Ph)**.

**Bond**	**BP86**	**Experiment**	Optimized (red) and X-Ray (blue)
**P_N−P_P**	2.291	2.243	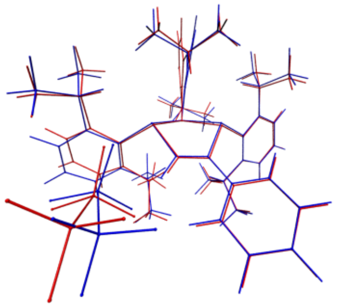
**P_P−P_C**	2.253	2.212	
**P_C−C**	1.848	1.822	
**C−N**	1.318	1.311	
**N−P_N**	1.800	1.790	
**P_N−P_P−P_C**	86.89	88.53	
**P_C−C−N**	115.15	115.82	
**C−N−P_N**	124.25	124.01	
**N−P_N−P_P**	89.33	91.24	
**P_P−P_C−C**	93.04	93.98	

Table S-9. Selected computed and experimental bond lengths and angles of **$[1_{tBu}]^+$ (R' = Me)**.

**Bond**	**BP86**	**Experiment**	Optimized (red) and X-Ray (blue)
**P_N−P_P**	2.257	2.212	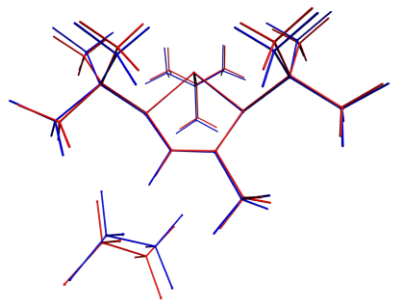
**P_P−P_C**	2.229	2.192	
**P_C−C**	1.839	1.806	
**C−N**	1.308	1.305	
**N−P_N**	1.771	1.769	
**P_N−P_P−P_C**	94.35	94.62	
**P_C−C−N**	119.96	119.5	
**C−N−P_N**	126.72	126.0	
**N−P_N−P_P**	96.48	97.32	
**P_P−P_C−C**	97.81	99.07	

Table S-10. Selected computed and experimental bond lengths and angles of **$[1_{tBu}]^+$ (R' = Ph)**.

**Bond**	**BP86**	**Experiment**	Optimized (red) and X-Ray (blue)
**P_N−P_P**	2.251	2.209	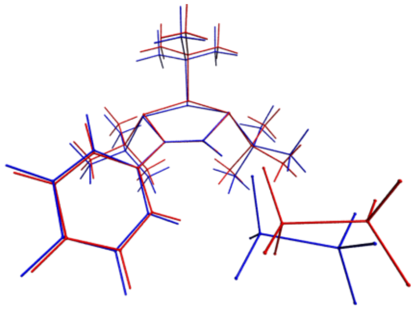
**P_P−P_C**	2.229	2.196	
**P_C−C**	1.837	1.816	
**C−N**	1.317	1.313	
**N−P_N**	1.777	1.767	
**P_N−P_P−P_C**	94.50	95.27	
**P_C−C−N**	121.42	119.89	
**C−N−P_N**	124.71	125.81	
**N−P_N−P_P**	97.18	97.33	
**P_P−P_C−C**	97.53	98.70	

Table S-11. Selected computed and experimental bond lengths and angles of **2_{Tipp} (R' = Me)**.

**Bond**	**BP86**	**Experiment**	Optimized (red) and X-Ray (blue)
**P_N–P_P**	2.309	2.265	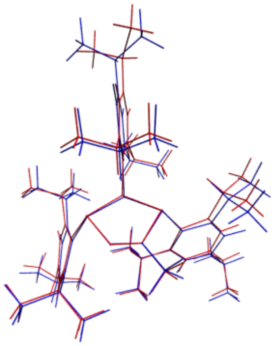
**P_P–P_C**	2.207	2.185	
**P_C–C**	1.879	1.850	
**C–N**	1.289	1.289	
**N–P_N**	1.736	1.716	
**P_N–P_P–P_C**	93.39	93.62	
**P_C–C–N**	122.75	123.2	
**C–N–P_N**	124.28	123.2	
**N–P_N–P_P**	97.94	98.42	
**P_P–P_C–C**	96.52	96.16	

Table S-12. Selected computed and experimental bond lengths and angles of **2_{Tipp} (R' = Ph)**.

**Bond**	**BP86**	**Experiment**	Optimized (red) and X-Ray (blue)
**P_N–P_P**	2.298	2.256	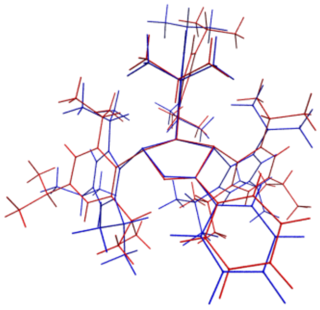
**P_P–P_C**	2.227	2.201	
**P_C–C**	1.883	1.853	
**C–N**	1.299	1.294	
**N–P_N**	1.722	1.708	
**P_N–P_P–P_C**	91.43	89.90	
**P_C–C–N**	121.33	121.9	
**C–N–P_N**	123.71	120.7	
**N–P_N–P_P**	96.62	97.06	
**P_P–P_C–C**	94.36	94.41	

Table S-13. Selected computed and experimental bond lengths and angles of **2_{tBu} (R' = Me)**.

**Bond**	**BP86**	**Experiment**	Optimized (red) and X-Ray (blue)
**P_N–P_P**	2.275	2.227	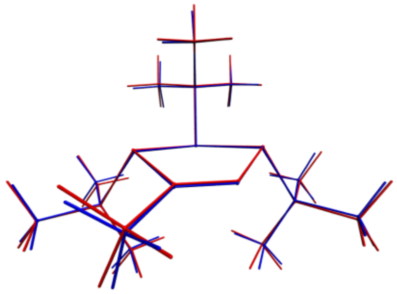
**P_P–P_C**	2.225	2.191	
**P_C–C**	1.890	1.867	
**C–N**	1.290	1.277	
**N–P_N**	1.727	1.713	
**P_N–P_P–P_C**	92.29	92.45	
**P_C–C–N**	122.34	123.1	
**C–N–P_N**	123.03	121.7	
**N–P_N–P_P**	99.99	100.52	
**P_P–P_C–C**	96.78	96.62	

Table S-14. Selected computed and experimental bond lengths and angles of **2_{tBu} (R' = Ph)**.

Bond	BP86	Experiment	Optimized (red) and X-Ray (blue)
**P_N−P_P**	2.273	2.232	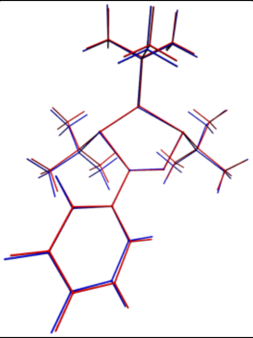
**P_P−P_C**	2.225	2.193	
**P_C−C**	1.893	1.873	
**C−N**	1.296	1.288	
**N−P_N**	1.716	1.704	
**P_N−P_P−P_C**	92.93	93.02	
**P_C−C−N**	122.94	122.91	
**C−N−P_N**	122.71	121.68	
**N−P_N−P_P**	99.89	100.37	
**P_P−P_C−C**	96.53	96.65	

6.3 Calculated UV-Vis spectra

Table S-15 Calculated electronic excitations of **[1$_{\text{Tipp}}$]$^{+}$ (R' = Me)** (values in parentheses correspond to those using smd=dichloromethane)

State	Symmetry	λ [nm]	Oscillator strength	Main excitation
S1	^{1}A	500.4<br>(499.8)	0.0025<br>(0.0076)	HOMO→LUMO
S2	^{1}A	388.4<br>(368.52)	0.0148<br>(0.0033)	HOMO→LUMO+1
S3	^{1}A	369.1<br>(360.08)	0.0017<br>(0.0449)	HOMO-1→LUMO

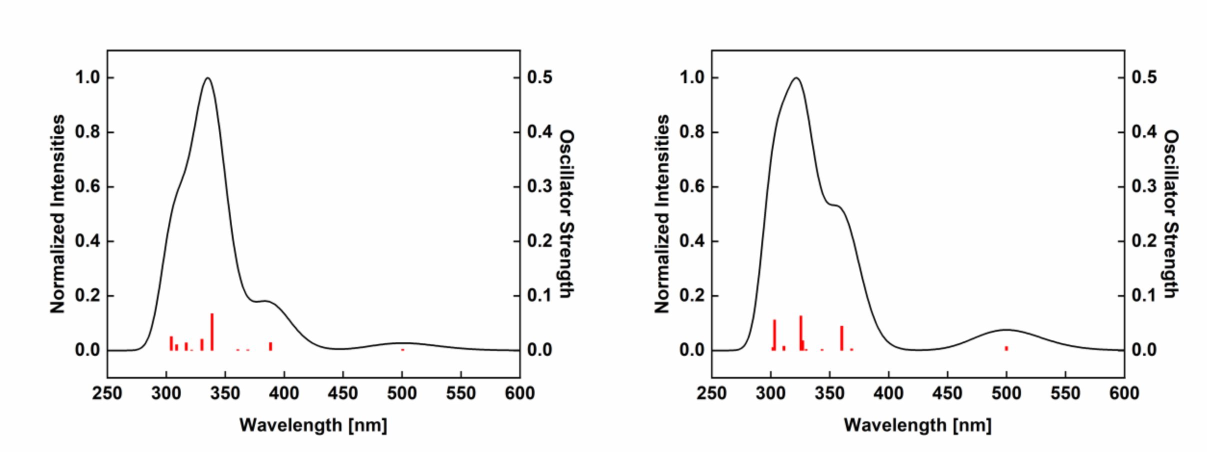

Figure S-150 Predicted UV-vis absorption spectra of **[1$_{\text{Tipp}}$]$^{+}$ (R' = Me)** including oscillator strengths (given as red bars) at the B3LYP/cc-pVTZ level of theory theory in the gas phase (left) and solution (smd=CH_2Cl_2; right). The phenomenological broadening (Gaussian) used to generate the illustrated spectra is σ = 0.2 eV

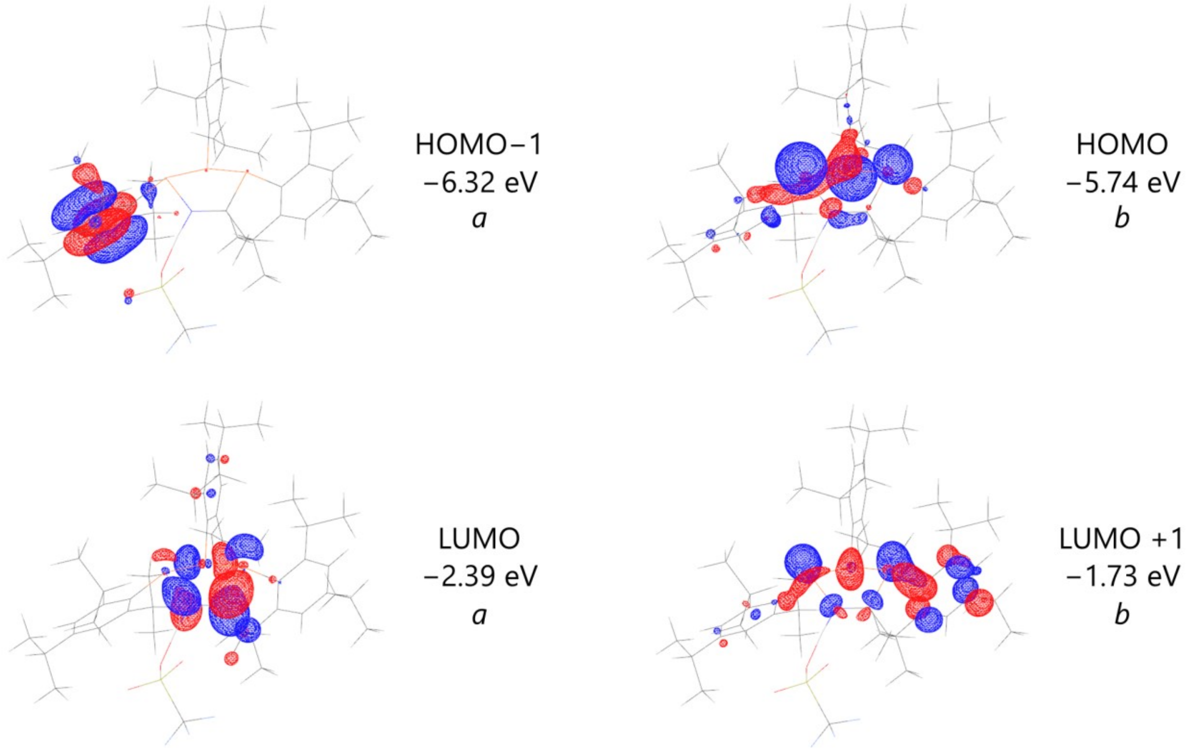

Figure S-151 Relevant Kohn-Sham orbitals of **[1_{Tipp}]$^+$ (R' = Me)** (B3LYP-D3/cc-PVTZ)

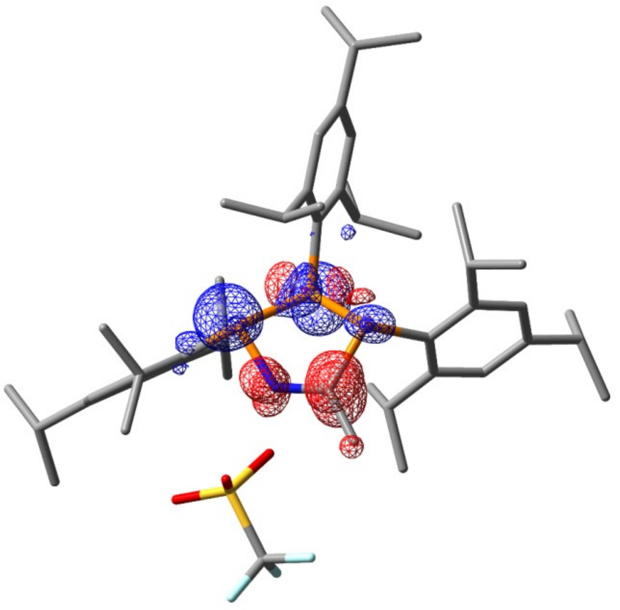

Figure S-152 Charge density difference diagram of the first excited state (HOMO-LUMO) illustrating charge transfers during excitation of complex **[1_{Tipp}]$^+$ (R' = Me)**. The blue regions correspond to the electron donor regions and the red regions to corresponding acceptor regions (D index = 1.260 Å)

Table S-16 Calculated electronic excitations of **[1$_{Tipp}$]$^+$ (R' = Ph)** (values in parentheses correspond to those using smd=dichloromethane).

State	Symmetry	λ [nm]	Oscillator strength	Main excitation
S1	^{1}A	567.2 (568.9)	0.0156 (0.0201)	HOMO→LUMO
S2	^{1}A	425.3 (403.6)	0.0021 (0.0976)	HOMO-1→LUMO
S3	^{1}A	412.7 (383.91)	0.0272 (0.0029)	HOMO-2→LUMO

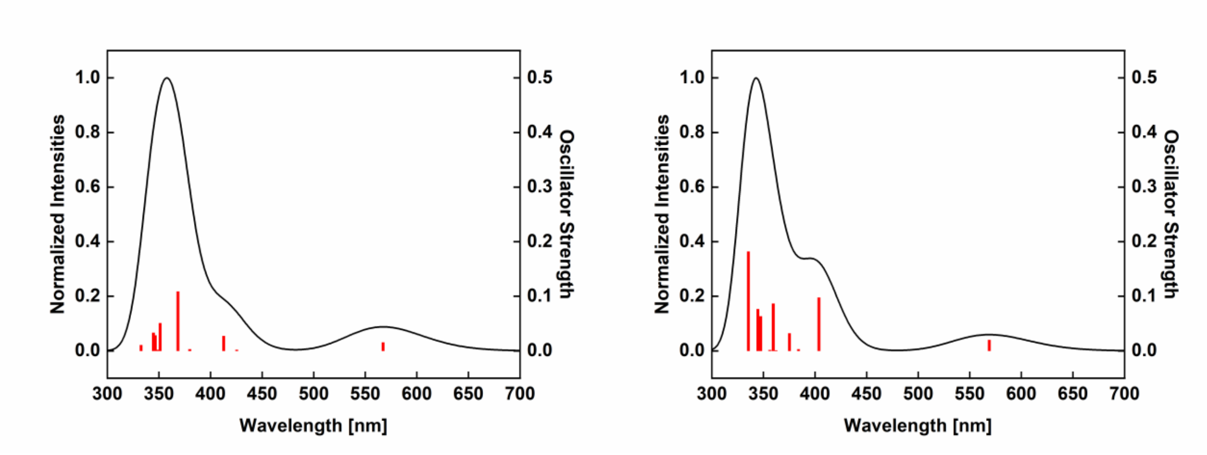

Figure S-153 Predicted UV-vis absorption spectra of **[1$_{Tipp}$]$^+$ (R' = Ph)** including oscillator strengths (given as red bars) at the B3LYP/cc-pVTZ level of theory in the gas phase (left) and solution (smd=CH_2Cl_2; right). The phenomenological broadening (Gaussian) used to generate the illustrated spectra is σ = 0.2 eV

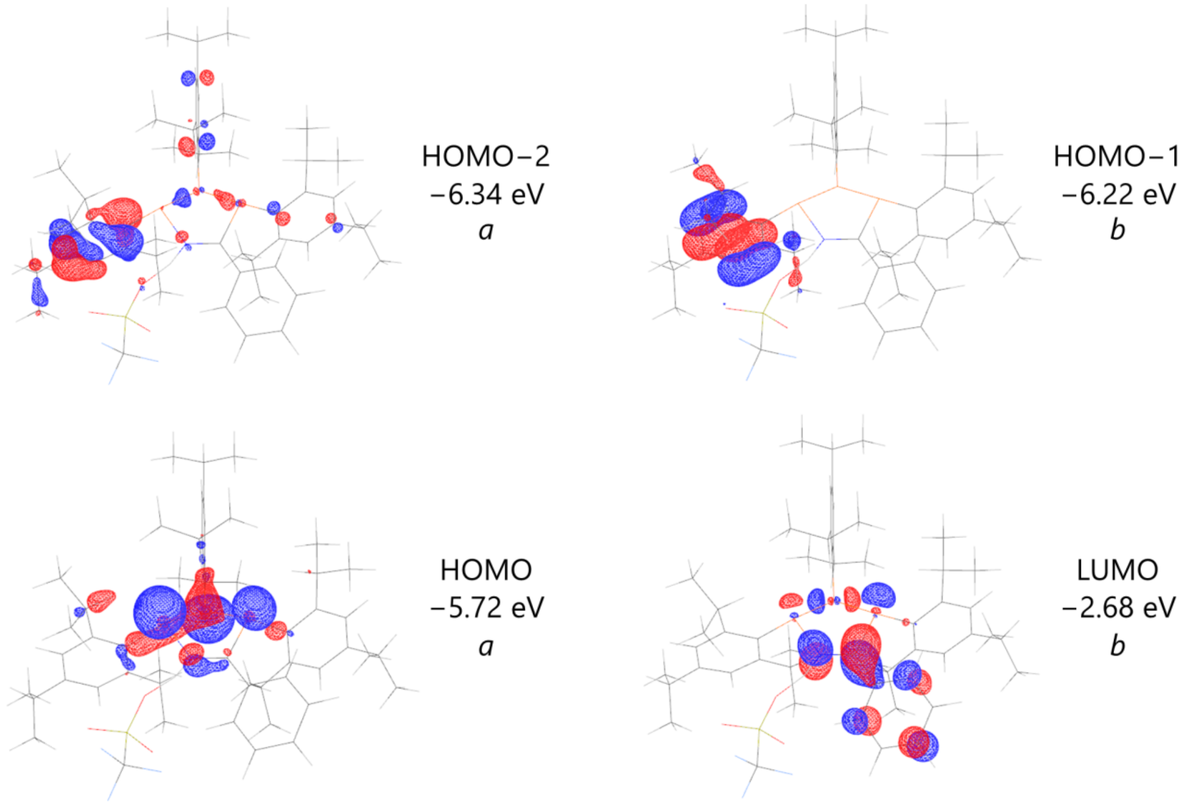

Figure S-154 Relevant Kohn-Sham orbitals of **[1$_{\mathbf{Tipp}}$]$^+$ (R' = Ph)** (B3LYP-D3/cc-PVTZ)

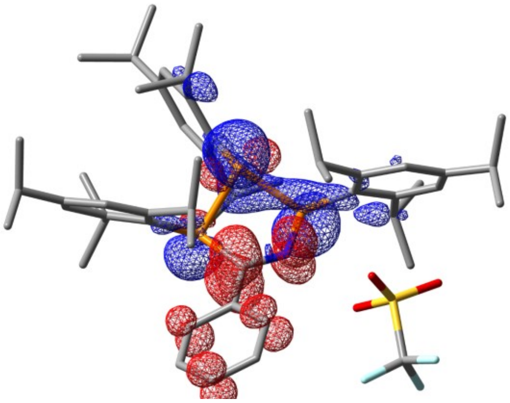

Figure S-155 Charge density difference diagram of the first excited state (HOMO-LUMO) illustrating charge transfers during excitation of complex **[1$_{\mathbf{Tipp}}$]$^+$ (R' = Ph)**. The blue regions correspond to the electron donor regions and the red regions to corresponding acceptor regions (D index = 2.377 Å).

Table S-17 Calculated electronic excitations of **[1$_{Tipp}$]$^+$ (R' = *p*-MeOC$_6$H$_4$)** (values in parentheses correspond to those using smd=dichloromethane).

State	Symmetry	λ [nm]	Oscillator strength	Main excitation
S1	^1A	543.8 (540.9)	0.0182 (0.0217)	HOMO→LUMO
S2	^1A	411.8 (397.5)	0.0042 (0.2602)	HOMO-1→LUMO
S3	^1A	402.5 (375.1)	0.0670 (0.0738)	HOMO-2→LUMO

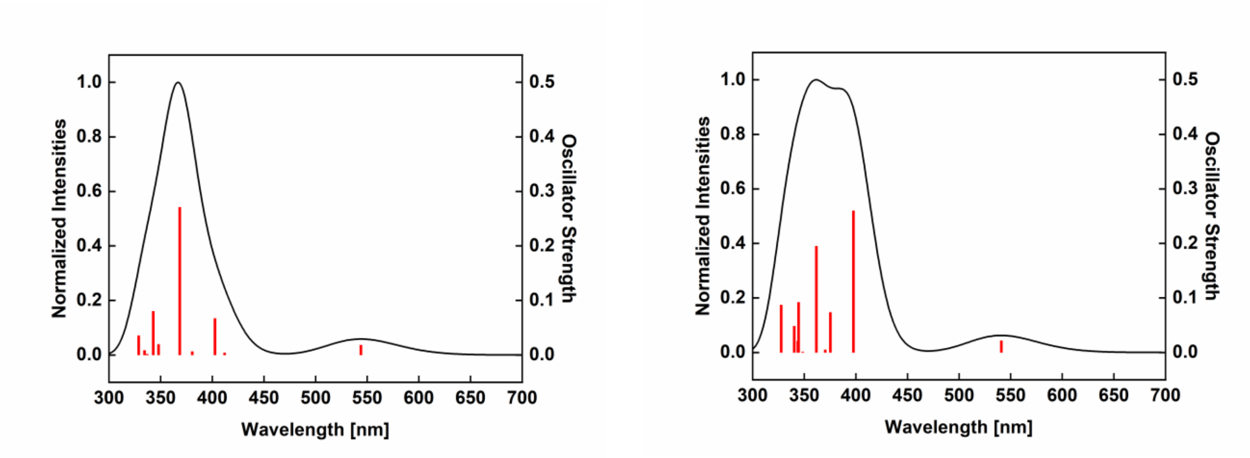

Figure S-156 Predicted UV-vis absorption spectra of **[1$_{Tipp}$]$^+$ (R' = *p*-MeOC$_6$H$_4$)** including oscillator strengths (given as red bars) at the B3LYP/cc-pVTZ level of theory in the gas phase (left) and solution (sm=CH_2Cl_2; right). The phenomenological broadening (Gaussian) used to generate the illustrated spectra is σ = 0.2 eV.

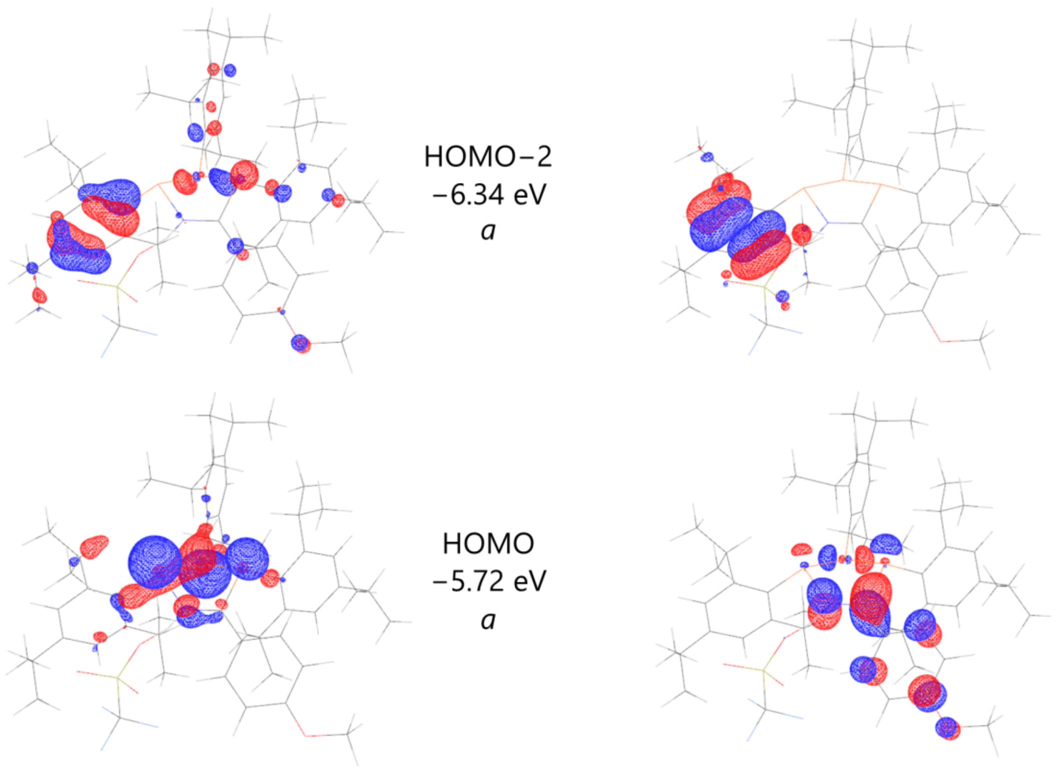

Figure S-158 Relevant Kohn-Sham orbitals of **[1$_{Tipp}$]$^+$ (R’ = *p*-MeOC$_6$H$_4$)** (B3LYP-D3/cc-PVTZ)

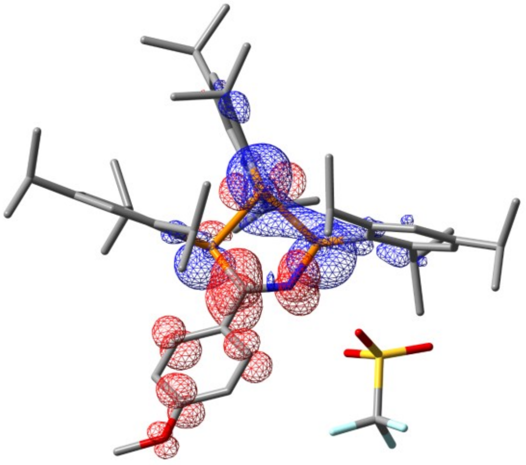

Figure S-157 Charge density difference diagram of the first excited state (HOMO-LUMO) illustrating charge transfers during excitation of complex **[1$_{Tipp}$]$^+$ (R’ = *p*-MeOC$_6$H$_4$)**. The blue regions correspond to the electron donor regions and the red regions to corresponding acceptor regions (D index = 2.074).

Table S-18 Calculated electronic excitations of **[1$_{Tipp}$]$^+$ (R' = *p*-CF$_3$C$_6$H$_4$)** (values in parentheses correspond to those using smd=dichloromethane).

State	Symmetry	λ [nm]	Oscillator strength	Main excitation
S1	^1A	598.8 (605.8)	0.0181 (0.0230)	HOMO→LUMO
S2	^1A	448.3 (424.09)	0.0019 (0.0808)	HOMO-1→LUMO
S3	^1A	433.3 (405.11)	0.0242 (0.0029)	HOMO-2→LUMO

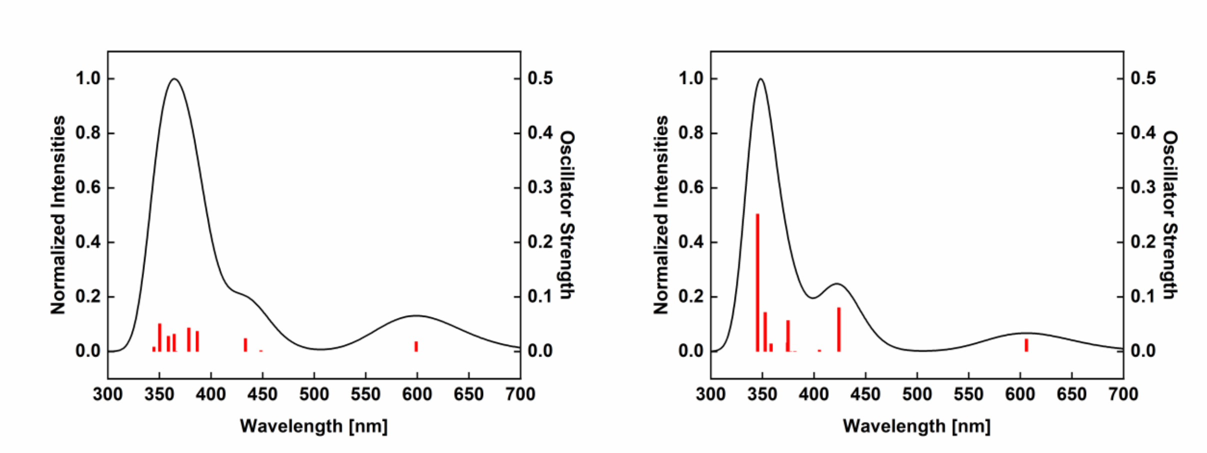

Figure S-159 Predicted UV-vis absorption spectra of **[1$_{Tipp}$]$^+$ (R' = *p*-CF$_3$C$_6$H$_4$)** including oscillator strengths (given as red bars) at the B3LYP/cc-pVTZ level of theory. The in the gas phase (left) and solution (smd=CH_2Cl_2; right). phenomenological broadening (Gaussian) used to generate the illustrated spectra is σ = 0.2 eV.

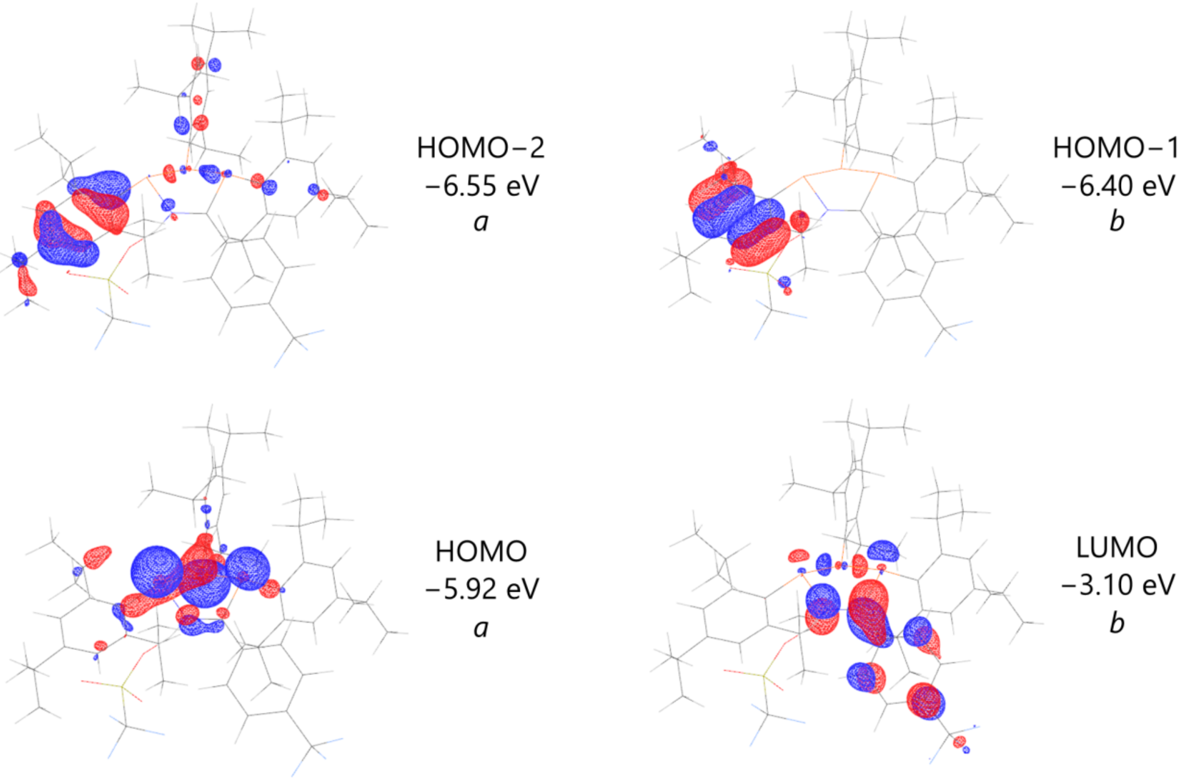

Figure S-160 Relevant Kohn-Sham orbitals of $[1_{Tipp}]^+$ (R’ = *p*-$CF_3C_6H_4$) (B3LYP-D3/cc-PVTZ)

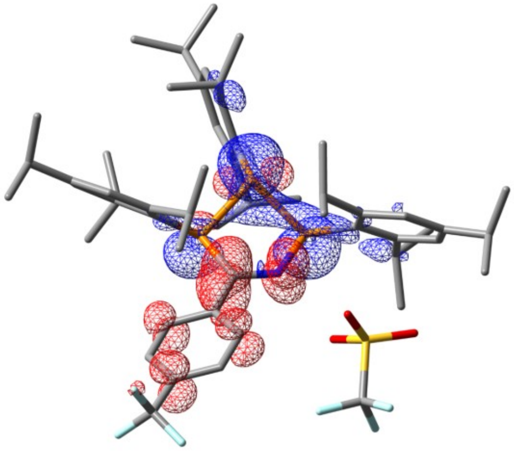

Figure S-161 Charge density difference diagram of the first excited state (HOMO-LUMO) illustrating charge transfers during excitation of complex $[1_{Tipp}]^+$ (R’ = *p*-$CF_3C_6H_4$). The blue regions correspond to the electron donor regions and the red regions to corresponding acceptor regions (D index = 2.266 Å).

Table S-19 Calculated electronic excitations of **[1$_{\mathbf{Dipp}}$]$^+$ (R' = Me)** (values in parentheses correspond to those using smd=dichloromethane).

State	Symmetry	λ [nm]	Oscillator strength	Main excitation
S1	^1A	472.5 (471.5)	0.0047 (0.0067)	HOMO→LUMO
S2	^1A	379.8 (379.2)	0.0091 (0.0164)	HOMO→LUMO+1
S3	^1A	333.9 (330.9)	0.0406 (0.0783)	HOMO-1→LUMO

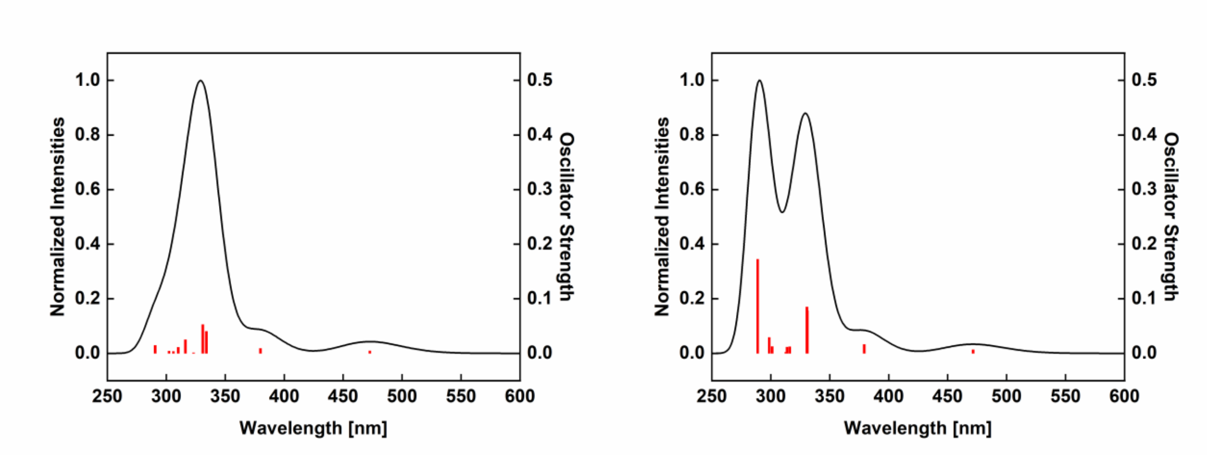

Figure S-162 Predicted UV-vis absorption spectra of **[1$_{\mathbf{Dipp}}$]$^+$ (R' = Me)** including oscillator strengths (given as red bars) at the B3LYP/cc-pVTZ level of theory in the gas phase (left) and solution (smd=CH_2Cl_2; right). The phenomenological broadening (Gaussian) used to generate the illustrated spectra is σ = 0.2 eV.

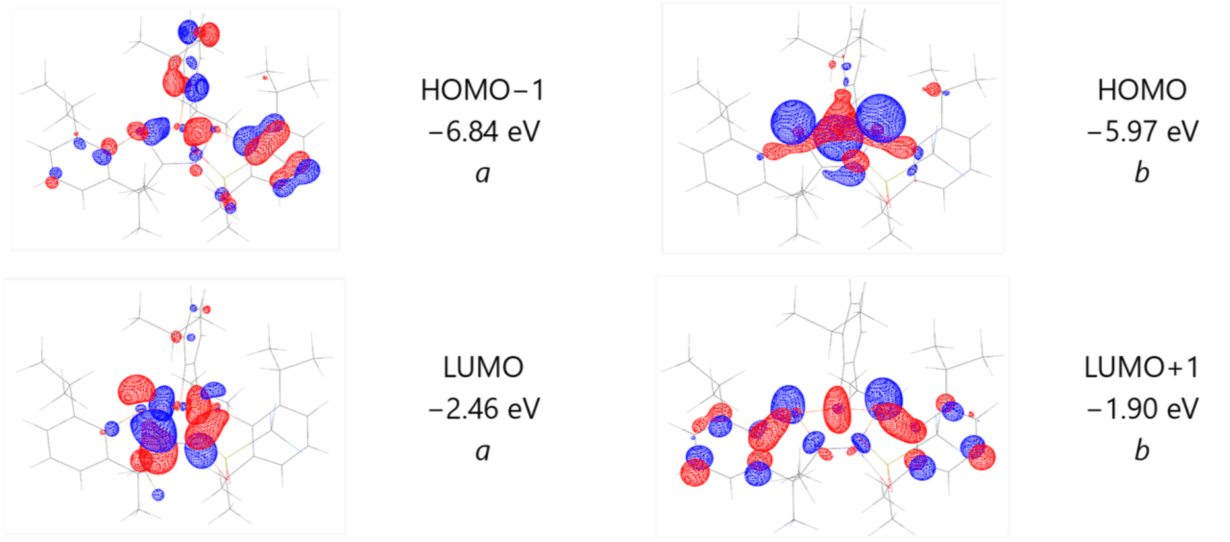

Figure S-163 Relevant Kohn-Sham orbitals of **$[1_{Dipp}]^+$ (R' = Me)** (B3LYP-D3/cc-PVTZ)

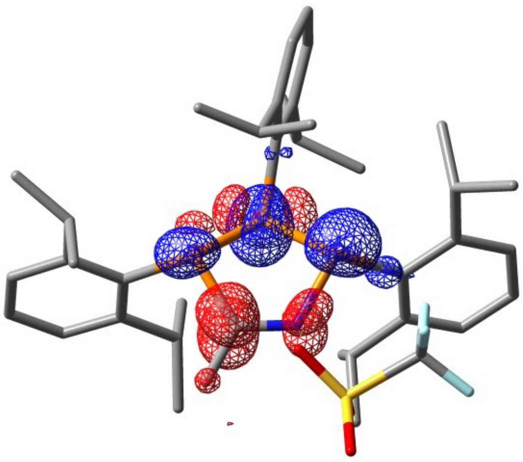

Figure S-164 Charge density difference diagram of the first excited state (HOMO-LUMO) illustrating charge transfers during excitation of complex **$[1_{Dipp}]^+$ (R' = Me)**. The blue regions correspond to the electron donor regions and the red regions to corresponding acceptor regions (D index = 0.998 Å).

Table S-20 Calculated electronic excitations of **[1$_{Dipp}$]$^+$ (R' = Ph)** (values in parentheses correspond to those using smd=dichloromethane).

State	Symmetry	λ [nm]	Oscillator strength	Main excitation
S1	^1A	583.7 (572.6)	0.0215 (0.0267)	HOMO→LUMO
S2	^1A	425.9 (399.98)	0.0026 (0.1072)	HOMO-1→LUMO
S3	^1A	406.1 (380.32)	0.0392 (0.0044)	HOMO-2→LUMO

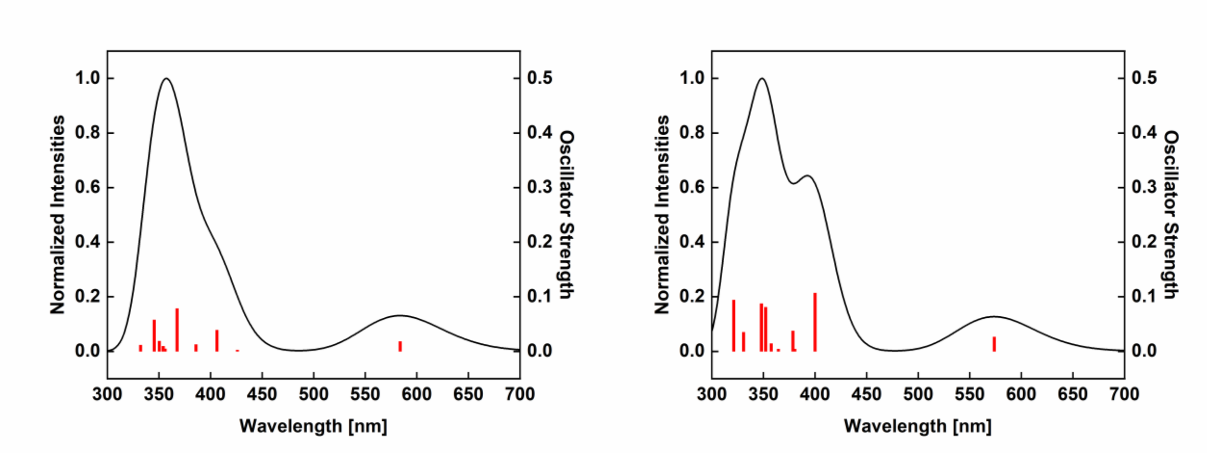

Figure S-165 Predicted UV-vis absorption spectra of **[1$_{Dipp}$]$^+$ (R' = Ph)** including oscillator strengths (given as red bars) at the B3LYP/cc-pVTZ level of theory in the gas phase (left) and solution (smd=CH_2Cl_2; right). The phenomenological broadening (Gaussian) used to generate the illustrated spectra is σ = 0.2 eV.

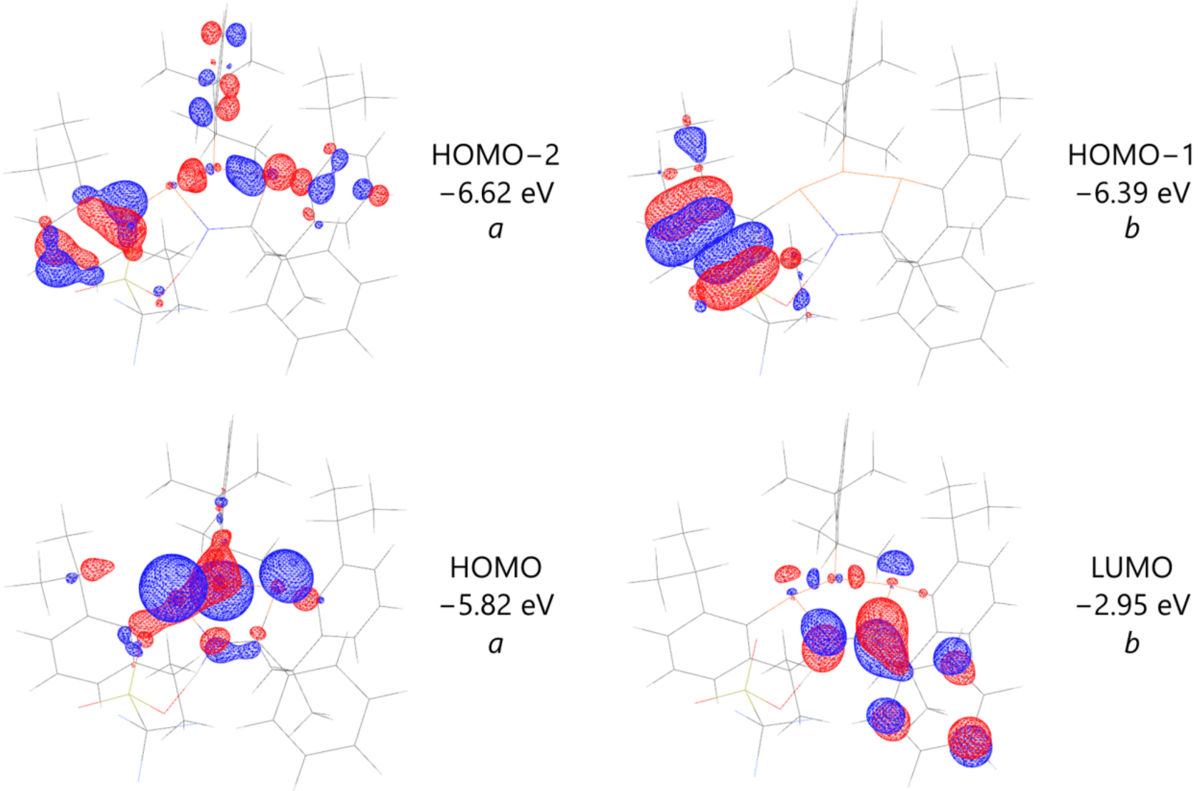

Figure S-166 Relevant Kohn-Sham orbitals of **[1$_{\mathbf{Dipp}}$]$^{+}$ (R' = Ph)** (B3LYP-D3/cc-PVTZ)

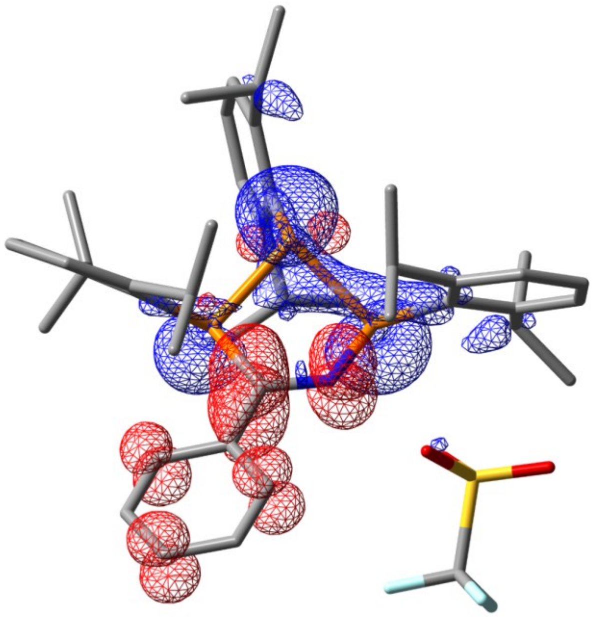

Figure S-167 Charge density difference diagram of the first excited state (HOMO-LUMO) illustrating charge transfers during excitation of complex **[1$_{\mathbf{Dipp}}$]$^{+}$ (R' = Ph)**. The blue regions correspond to the electron donor regions and the red regions to corresponding acceptor regions (D index = 2.304 Å).

Table S-21 Calculated electronic excitations of **[1$_{Mes}$]$^+$ (R' = Ph)** (values in parentheses correspond to those using smd=dichloromethane).

State	Symmetry	λ [nm]	Oscillator strength	Main excitation
S1	^1A	467.8<br>(470.1)	0.0413<br>(0.0534)	HOMO→LUMO
S2	^1A	428.9<br>(427.7)	0.0622<br>(0.1155)	HOMO-1→LUMO<br>HOMO-2→LUMO
S3	^1A	425.0<br>(385.2)	0.0076<br>(0.0001)	HOMO-1→LUMO<br>HOMO-2→LUMO

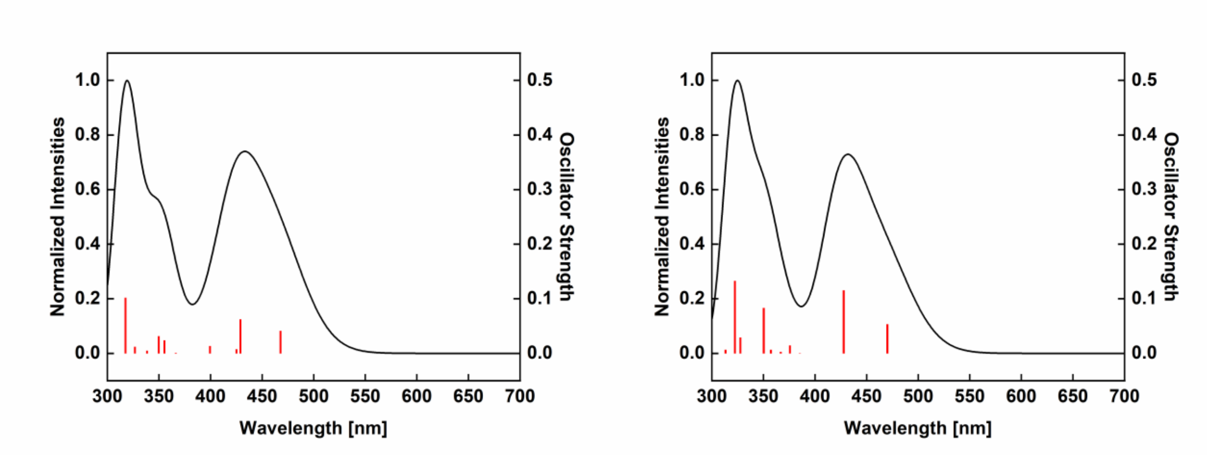

Figure S-168 Predicted UV-vis absorption spectra of **[1$_{Mes}$]$^+$ (R' = Ph)** including oscillator strengths (given as red bars) at the B3LYP/cc-pVTZ level of theory in the gas phase (left) and solution (smd=CH_2Cl_2; right). The phenomenological broadening (Gaussian) used to generate the illustrated spectra is σ = 0.2 eV

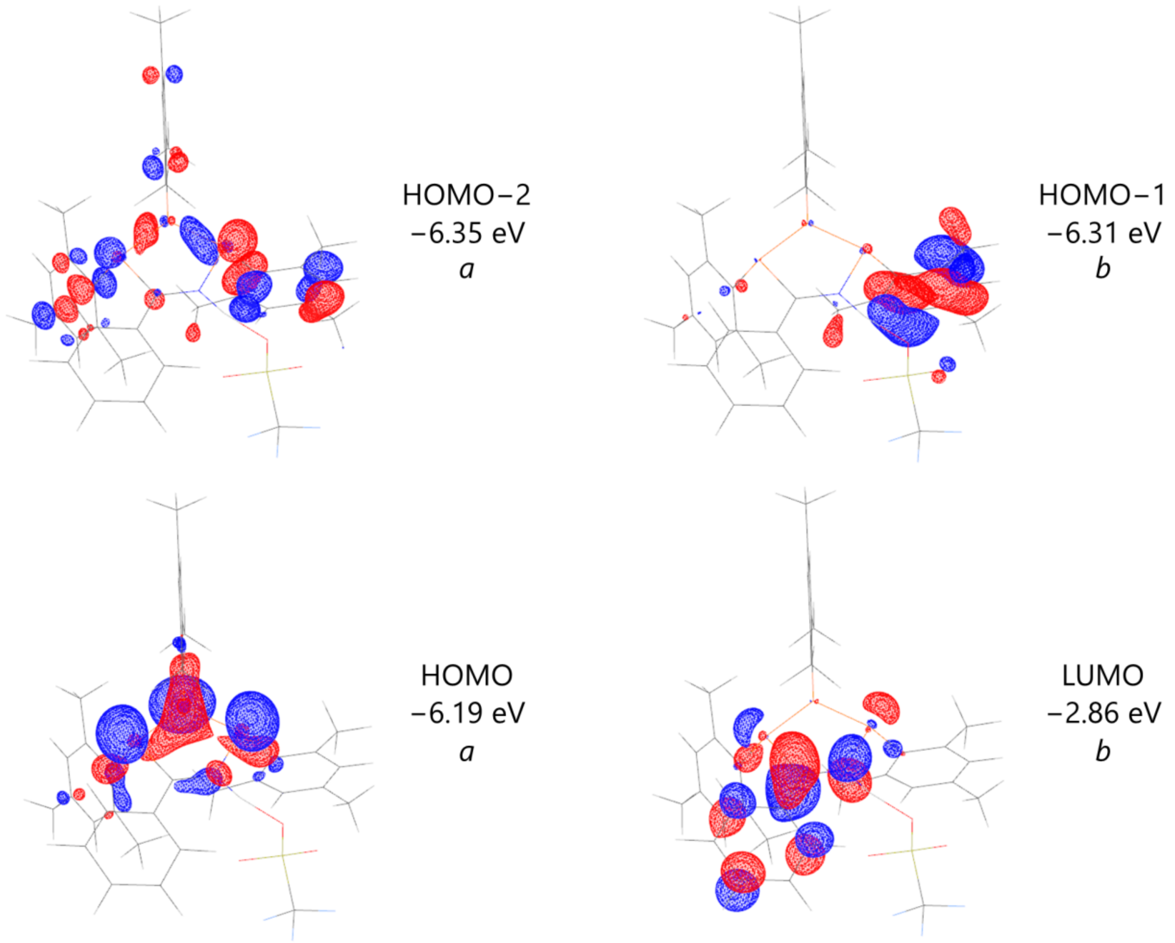

Figure S-169 Relevant Kohn-Sham orbitals of **[1$_{Mes}$]$^{+}$ (R' = Ph)** (B3LYP-D3/cc-PVTZ)

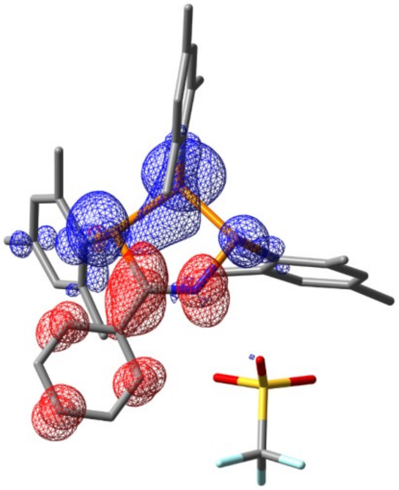

Figure S-170 Charge density difference diagram of the first excited state (HOMO-LUMO) illustrating charge transfers during excitation of complex [1$_{Mes}$]$^{+}$ (R' = Ph). The blue regions correspond to the electron donor regions and the red regions to corresponding acceptor regions (D index = 2.637 Å)

Table S-22 Calculated electronic excitations of **[1$_{tBu}$]$^+$ (R' = Me)** (values in parentheses correspond to those using smd=dichloromethane).

State	Symmetry	λ [nm]	Oscillator strength	Main excitation
S1	^1A	380.6 (369.12)	0.0368 (0.0583)	HOMO→LUMO
S2	^1A	304.1 (299.02)	0.0311 (0.0487)	HOMO-1→LUMO
S3	^1A	291.0 (274.36)	0.0057 (0.0458)	HOMO-2→LUMO

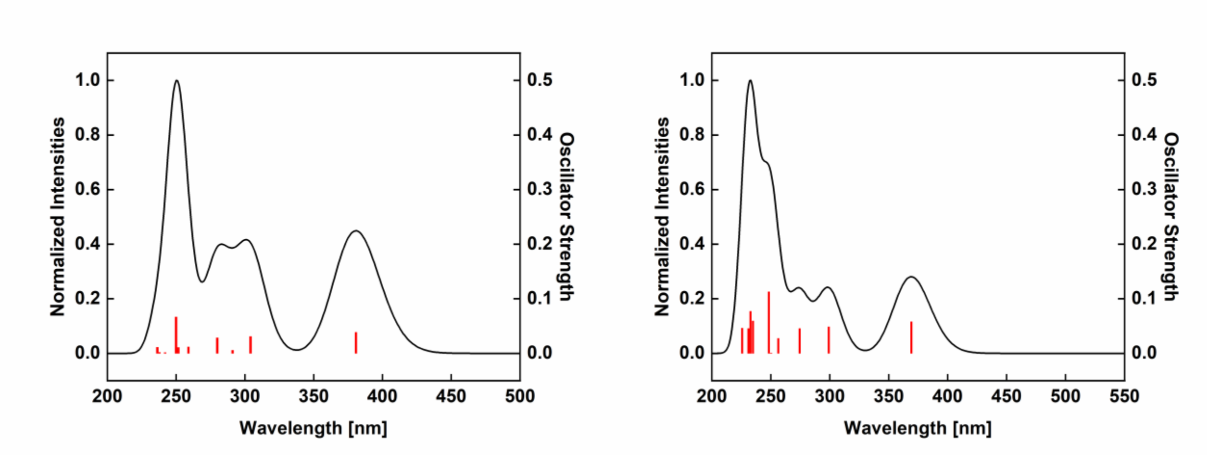

Figure S-171 Predicted UV-vis absorption spectra of **[1$_{tBu}$]$^+$ (R' = Me)** including oscillator strengths (given as red bars) at the B3LYP/cc-pVTZ level of theory in the gas phase (left) and solution (smd=CH_2Cl_2; right). The phenomenological broadening (Gaussian) used to generate the illustrated spectra is σ = 0.2 eV

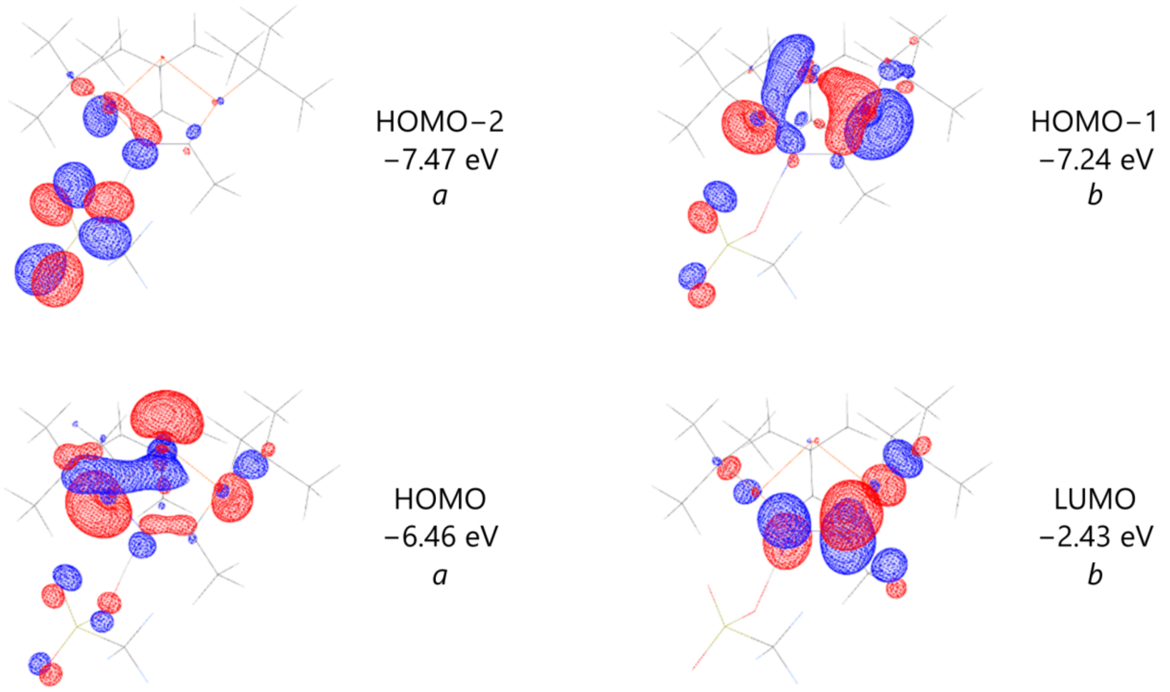

Figure S-172 Relevant Kohn-Sham orbitals of **$[1_{tBu}]^+$ (R' = Me)** (B3LYP-D3/cc-PVTZ)

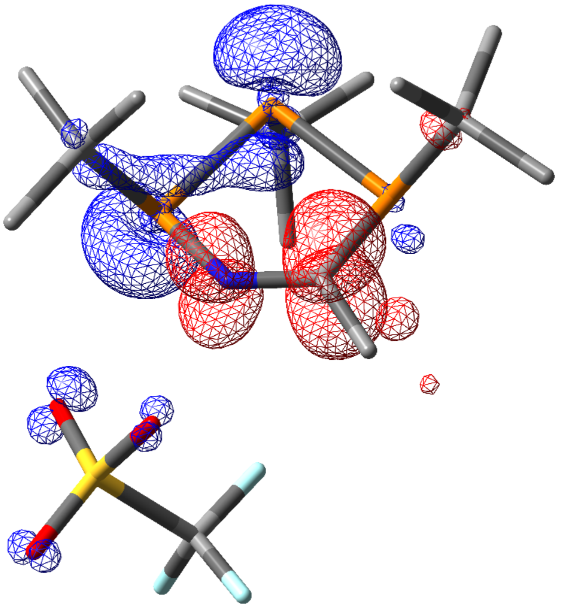

Figure S-173 Charge density difference diagram of the first excited state (HOMO-LUMO) illustrating charge transfers during excitation of complex **$[1_{tBu}]^+$ (R' = Me)**. The blue regions correspond to the electron donor regions and the red regions to corresponding acceptor regions (D index = 1.863 Å)

Table S-23 Calculated electronic excitations of **[1$_{tBu}$]$^+$ (R' = Ph)** (values in parentheses correspond to those using smd=dichloromethane).

State	Symmetry	λ [nm]	Oscillator strength	Main excitation
S1	^1A	400.5 (408.6)	0.0512 (0.0688)	HOMO→LUMO
S2	^1A	334.1 (331.5)	0.0865 (0.2369)	HOMO-1→LUMO
S3	^1A	325.8 (303.7)	0.0537 (0.0237)	HOMO-2→LUMO

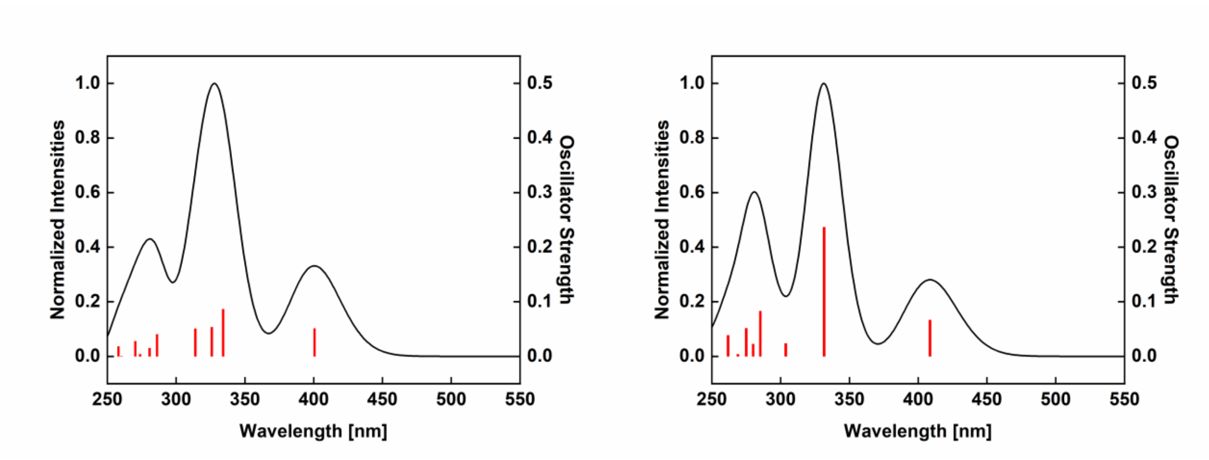

Figure S-174 Predicted UV-vis absorption spectra of **[1$_{tBu}$]$^+$ (R' = Ph)** including oscillator strengths (given as red bars) at the B3LYP/cc-pVTZ level of theory in the gas phase (left) and solution (smd=CH_2Cl_2; right). The phenomenological broadening (Gaussian) used to generate the illustrated spectra is σ = 0.2 eV

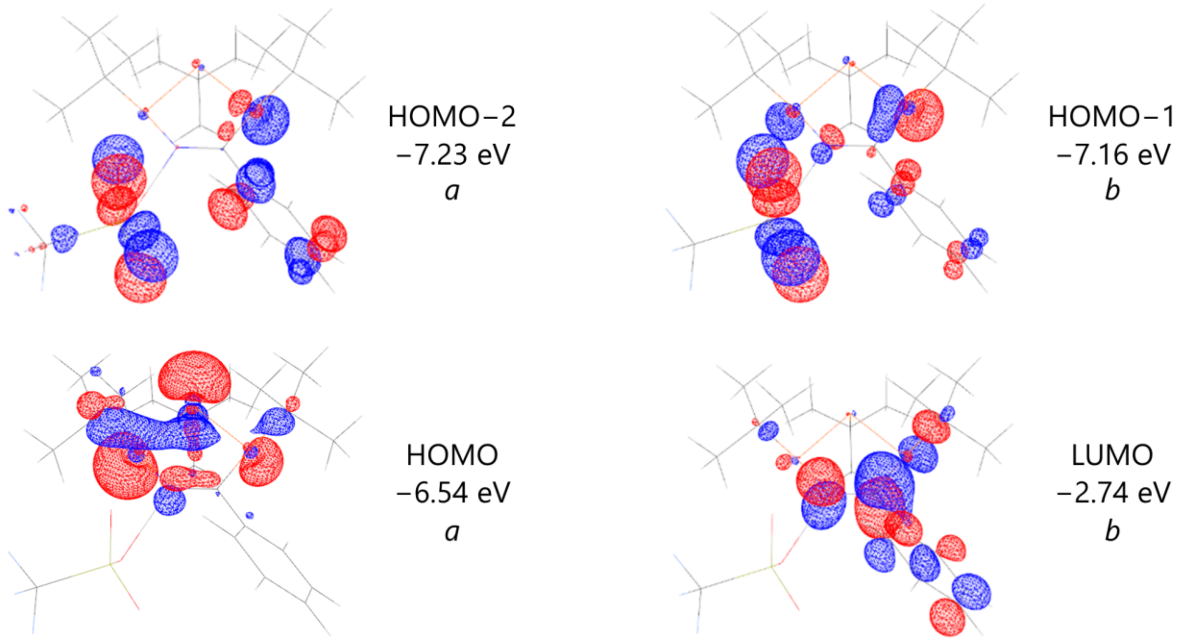

Figure S-175 Relevant Kohn-Sham orbitals of **$[1_{tBu}]^+$ (R’ = Ph)** (B3LYP-D3/cc-PVTZ)

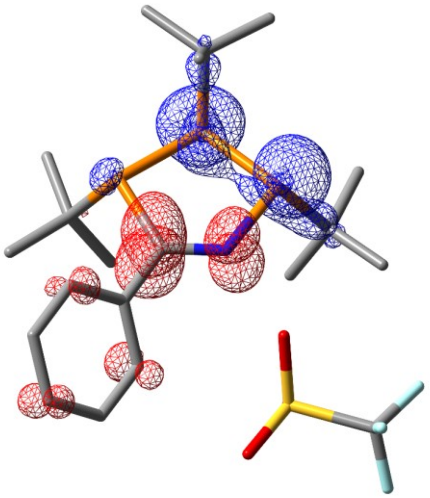

Figure S-176 Charge density difference diagram of the first excited state (HOMO-LUMO) illustrating charge transfers during excitation of complex **$[1_{tBu}]^+$ (R’ = Ph)**. The blue regions correspond to the electron donor regions and the red regions to corresponding acceptor regions (D index = 2.404 Å)

Table S-24 Calculated electronic excitations of $\mathbf{2}_{t\mathbf{Bu}}$ **(R' = Me)** (values in parentheses correspond to those using smd=dichloromethane).

State	Symmetry	λ [nm]	Oscillator strength	Main excitation
S1	^{1}A	332.8 (328.1)	0.0232 (0.0372)	HOMO→LUMO
S2	^{1}A	284.3 (281.09)	0.0276 (0.0456)	HOMO-1→LUMO
S3	^{1}A	279.5 (275.9)	0.0239 (0.0330)	HOMO→LUMO+1

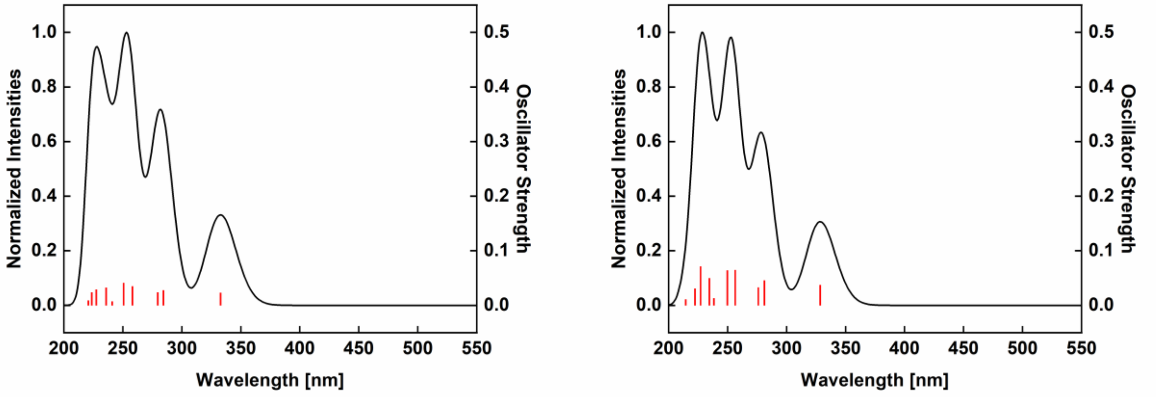

Figure S-177 Predicted UV-vis absorption spectra of $\mathbf{2}_{t\mathbf{Bu}}$ **(R' = Me)** including oscillator strengths (given as red bars) at the B3LYP/cc-pVTZ level of theory in the gas phase (left) and solution (smd=CH_2Cl_2; right). The phenomenological broadening (Gaussian) used to generate the illustrated spectra is σ = 0.2 eV.

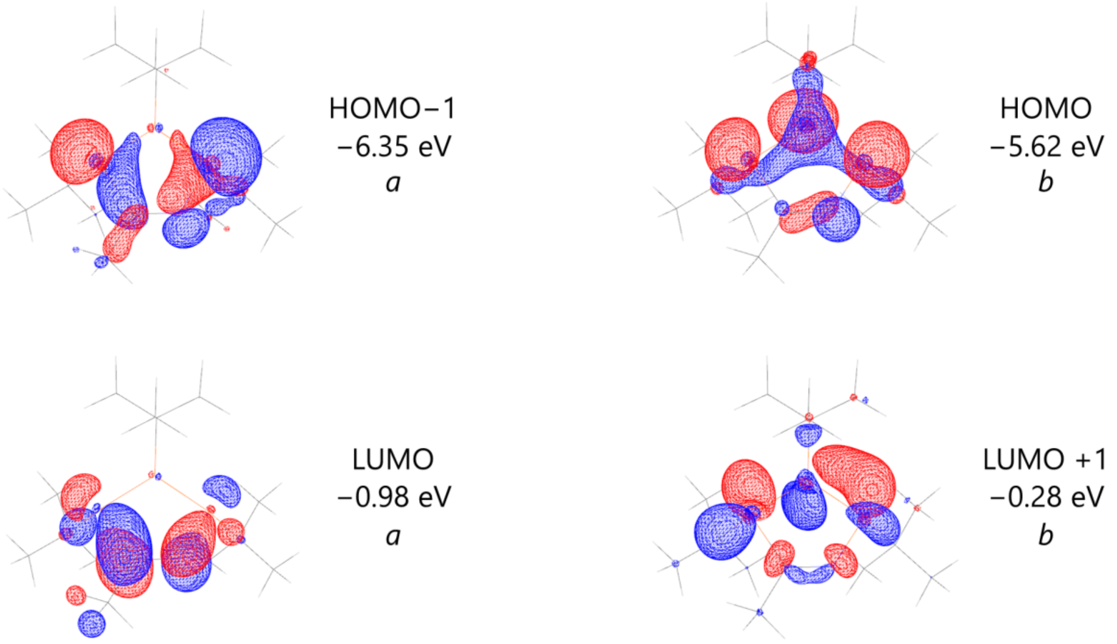

Figure S-178 Relevant Kohn-Sham orbitals $\mathbf{2}_{t\mathbf{Bu}}$ **(R’ = Me)** (B3LYP-D3/cc-PVTZ)

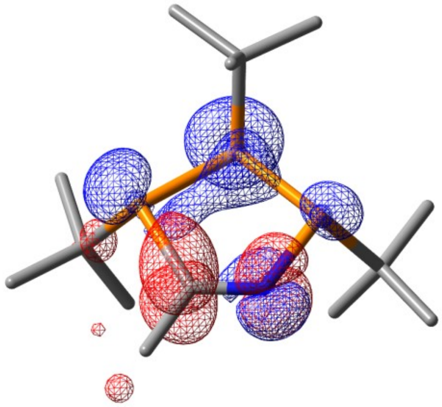

Figure S-179 Charge density difference diagram of the first excited state (HOMO-LUMO) illustrating charge transfers during excitation of complex $\mathbf{2}_{t\mathbf{Bu}}$ **(R’ = Me)**. The blue regions correspond to the electron donor regions and the red regions to corresponding acceptor regions (D index = 1.396 Å).

Table S-25 Calculated electronic excitations of $\mathbf{2}_{t\mathbf{Bu}}$ **(R' = Ph)** (values in parentheses correspond to those using smd=dichloromethane).

State	Symmetry	λ [nm]	Oscillator strength	Main excitation
S1	^{1}A	393.4<br>(392.1)	0.0495<br>(0.0768)	HOMO→LUMO
S2	^{1}A	327.2<br>(327.1)	0.1179<br>(0.1771)	HOMO-1→LUMO
S3	^{1}A	291.8<br>(288.2)	0.0025<br>(0.0022)	HOMO→LUMO+1

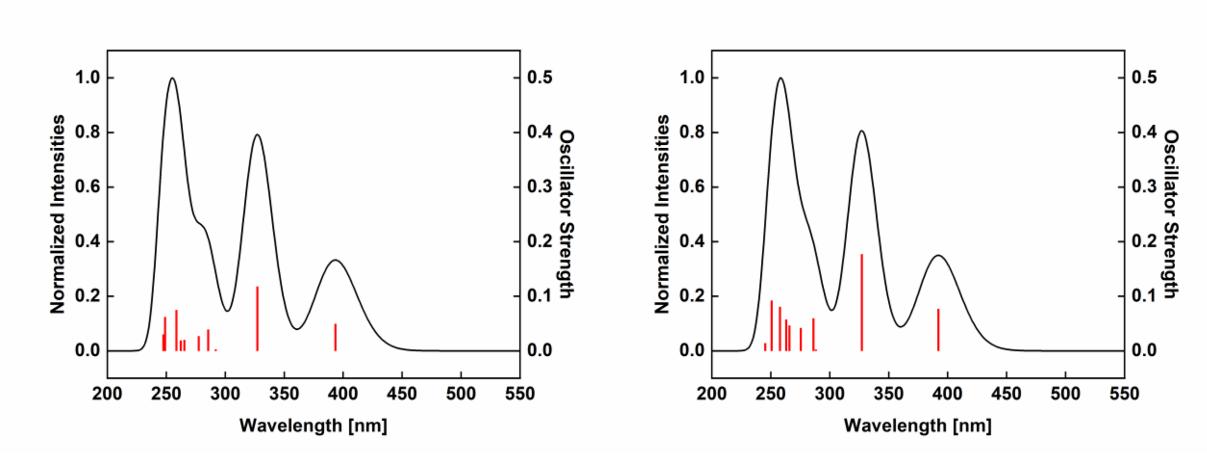

Figure S-180 Predicted UV-vis absorption spectra of $\mathbf{2}_{t\mathbf{Bu}}$ **(R' = Ph)** including oscillator strengths (given as red bars) at the B3LYP/cc-pVTZ level of theory in the gas phase (left) and solution (smd=CH_2Cl_2; right). The phenomenological broadening (Gaussian) used to generate the illustrated spectra is σ = 0.2 eV

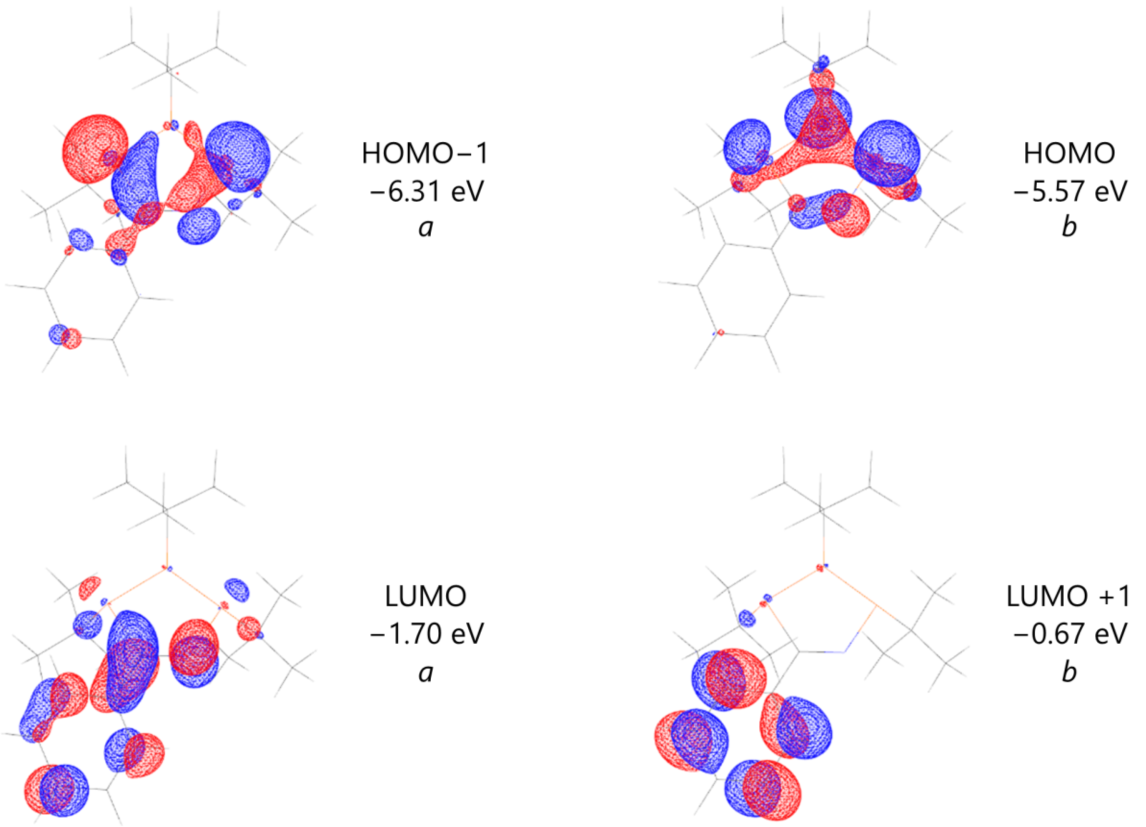

Figure S-181 Relevant Kohn-Sham orbitals of **2_{tBu} (R' = Ph)** (B3LYP-D3/cc-PVTZ)

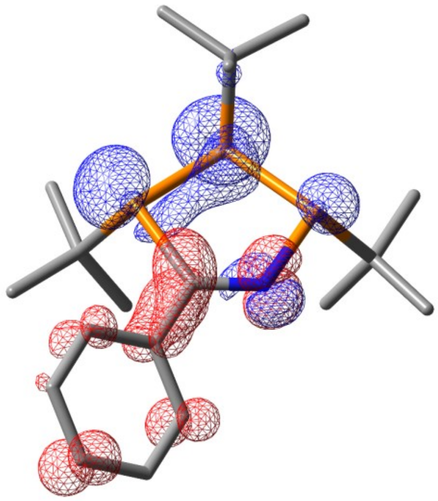

Figure S-182 Charge density difference diagram of the first excited state (HOMO-LUMO) illustrating charge transfers during excitation of complex **2_{tBu} (R' = Ph)**. The blue regions correspond to the electron donor regions and the red regions to corresponding acceptor regions (D index = 2.701 Å).

Table S-26 Calculated electronic excitations of **2_{Tipp} (R' = Me)** (values in parentheses correspond to those using smd=dichloromethane).

State	Symmetry	*λ* [nm]	Oscillator strength	Main excitation
S1	^1A	364.8	0.0042	HOMO→LUMO
S2	^1A	346.3	0.0044	HOMO→LUMO+1
S3	^1A	332.9	0.0696	HOMO→LUMO+2

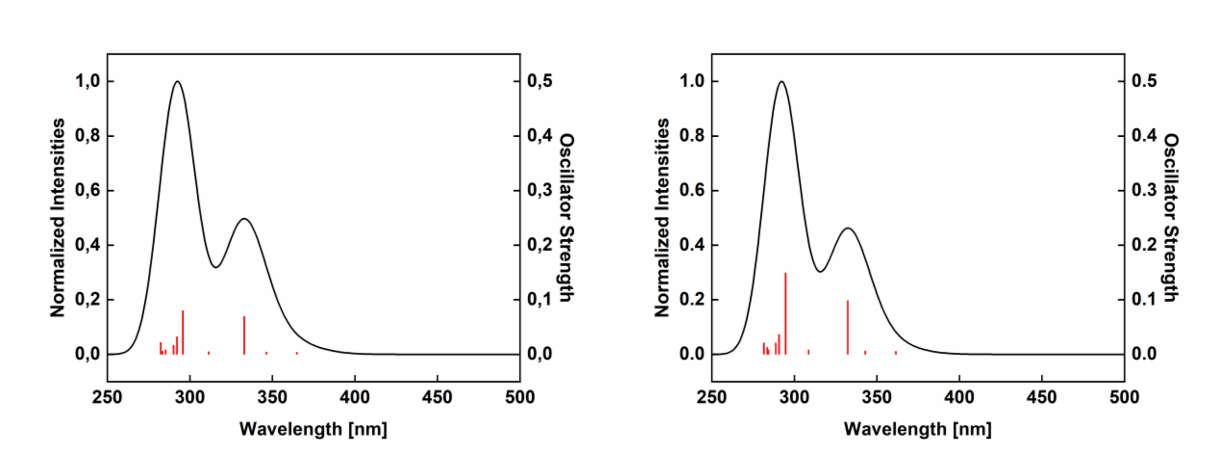

Figure S-183 Predicted UV-vis absorption spectra of **2_{Tipp} (R' = Me)** including oscillator strengths (given as red bars) at the B3LYP/cc-pVTZ level of theory. The phenomenological broadening (Gaussian) used to generate the illustrated spectra is σ = 0.2 eV.

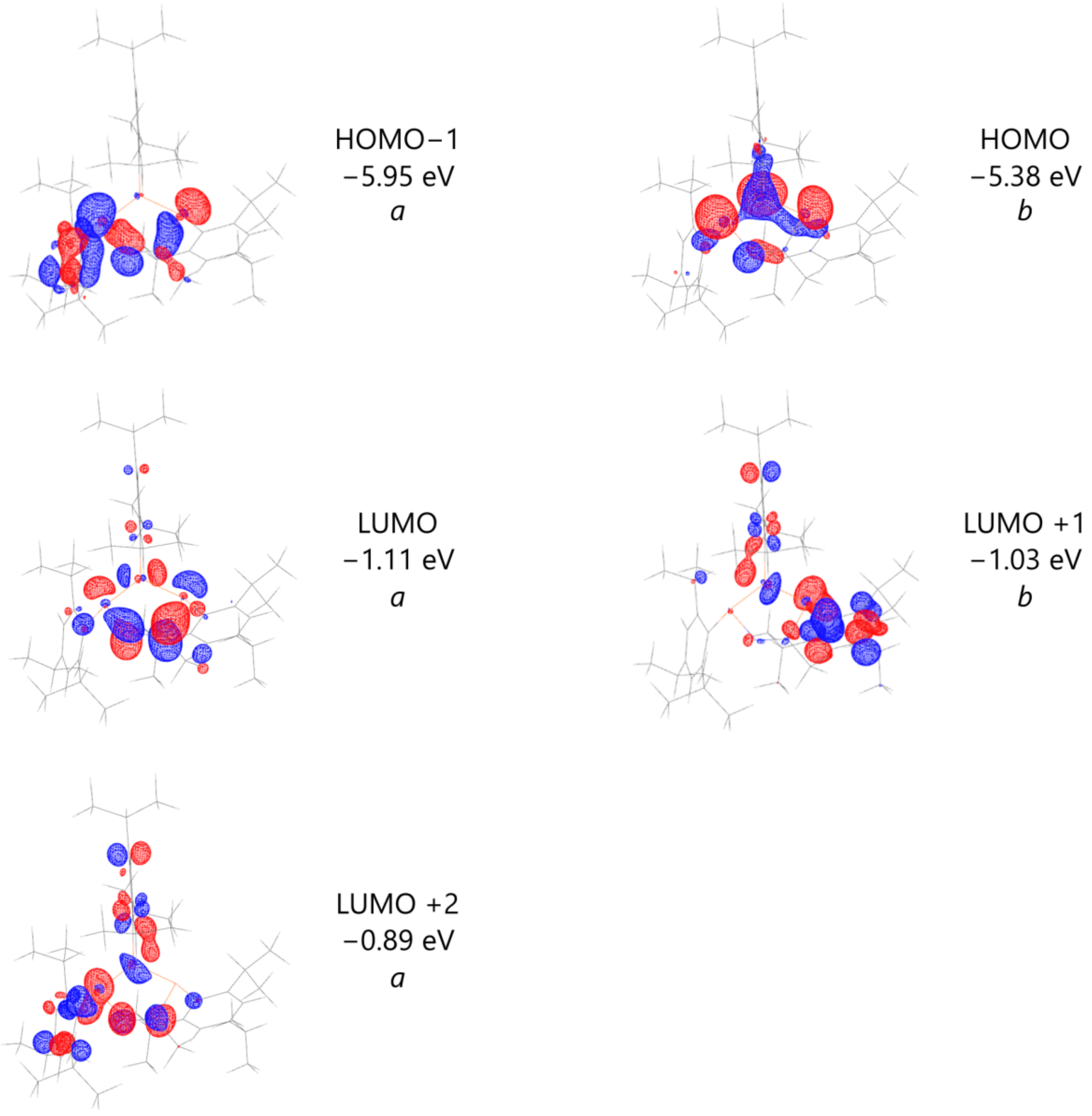

Figure S-184 Relevant Kohn-Sham orbitals of **2**$_{\mathbf{Tipp}}$ **(R' = Me)** (B3LYP-D3/cc-PVTZ)

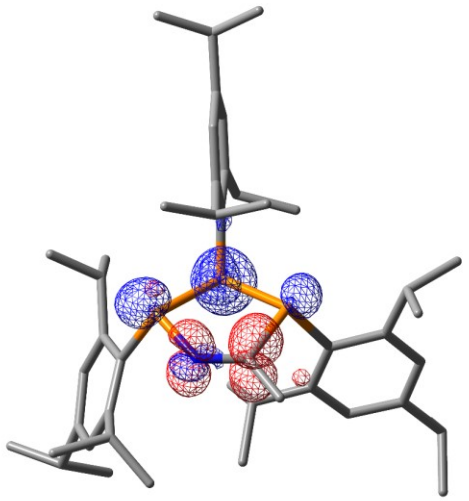

Figure S-185 Charge density difference diagram of the first excited state (HOMO-LUMO) illustrating charge transfers during excitation of complex 2_{Tipp} (R' = Me). The blue regions correspond to the electron donor regions and the red regions to corresponding acceptor regions (D index = 1.241 Å).

Table 27 Calculated electronic excitations of **2_{Tipp} (R' = Ph)**

State	Symmetry	λ [nm]	Oscillator strength	Main excitation
S1	1A	474.1	0.0216	HOMO→LUMO
S2	1A	374.5	0.0821	HOMO-1→LUMO
S3	1A	362.2	0.0207	HOMO→LUMO+1

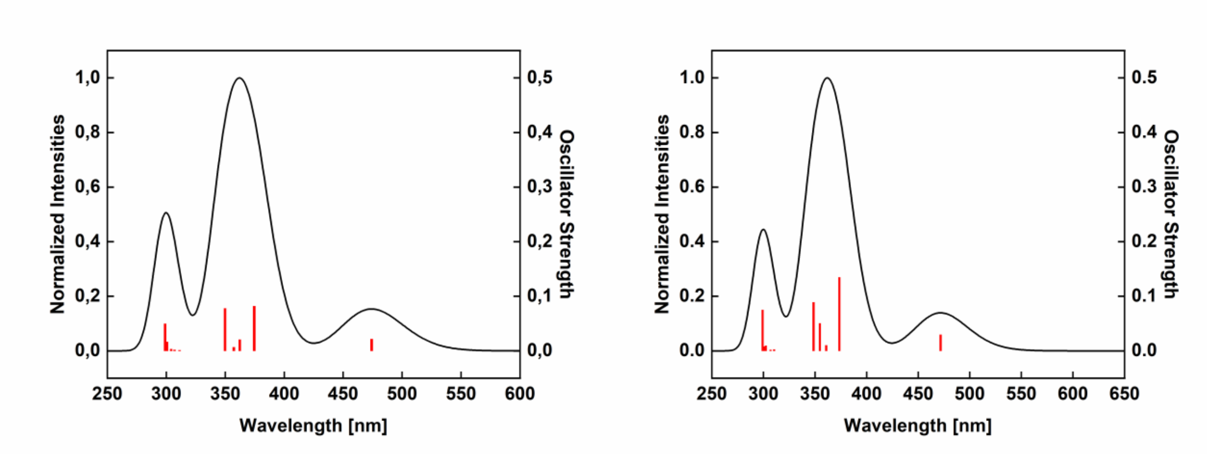

Figure S-186 Predicted UV-vis absorption spectra of **2_{Tipp} (R' = Ph)** including oscillator strengths (given as red bars) at the B3LYP/cc-pVTZ level of theory. The phenomenological broadening (Gaussian) used to generate the illustrated spectra is σ = 0.2 eV.

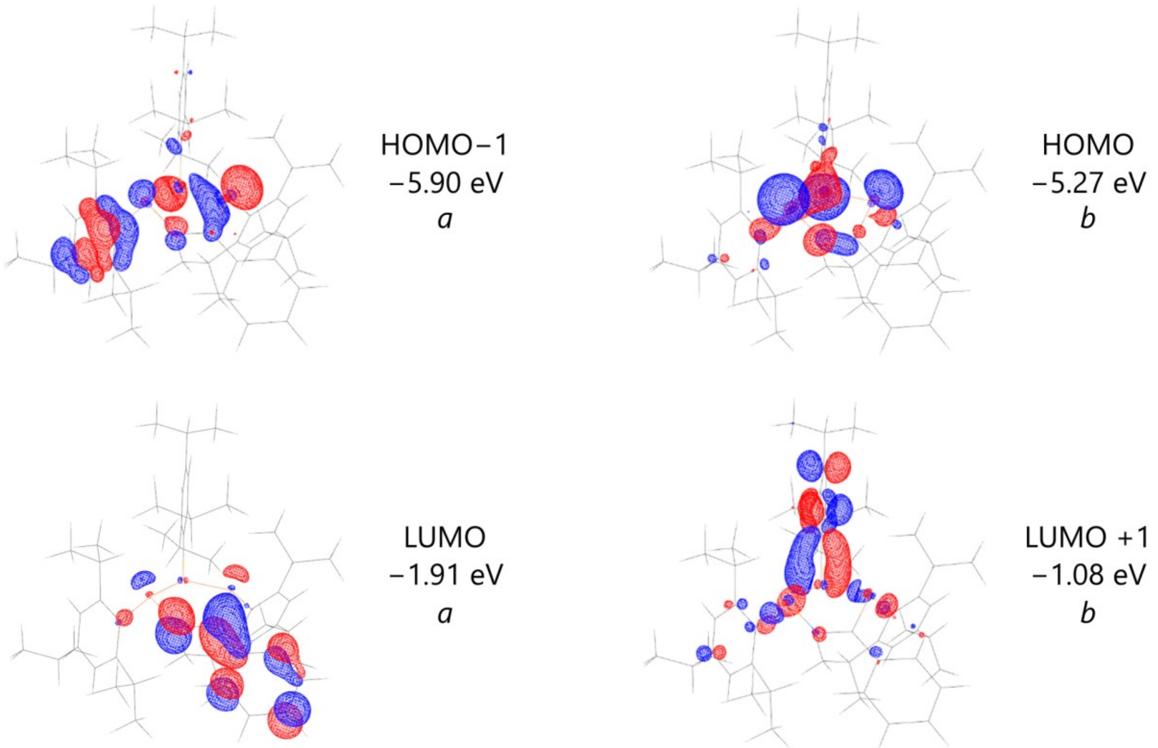

Figure S-187 Relevant Kohn-Sham orbitals of **2$_{\mathbf{Tipp}}$ (R' = Ph)** (B3LYP-D3/cc-PVTZ).

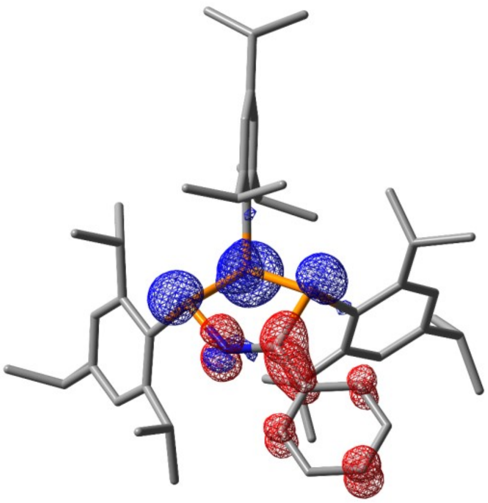

Figure S-188 Charge density difference diagram of the first excited state (HOMO-LUMO) illustrating charge transfers during excitation of complex **2$_{\mathbf{Tipp}}$ (R' = Ph)**. The blue regions correspond to the electron donor regions and the red regions to corresponding acceptor regions (D index = 2.777 Å).

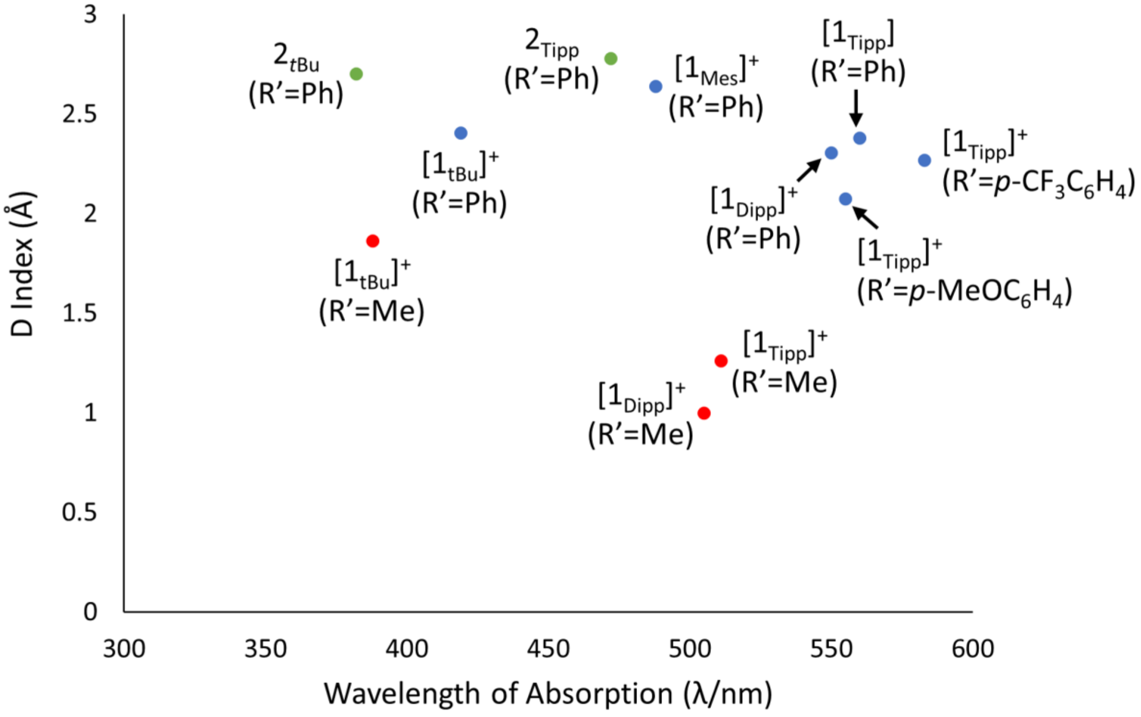

Figure S-189 Diagram showing the relation between the longest wavelength absorption (λ_{max}) and the D indices of selected examples of $\mathbf{1_R}^+$ and $\mathbf{2_R}$.

6.4 NBO Analysis

To gain further insight into the electronic structure of **[1Tipp]$^+$ (R' = Ph)** and its neutral deprotonated congener **2Tipp**, NBO analyses were performed on the BP86-D3/def2-SVP//b3lyp/cc-pVTZ level of theory, revealing a charge transfer of 0.14 e from from the OTf-counteranion in **[1Tipp]$^+$ (R' = Ph)**. Two distinct σ- and π-type NBOs between the C and N atom (Figure S37) show the corresponding Natural Localized Molecular Orbital, NLMO, and the % of the individual atoms to this NLMO, indicating a double bond (WBI = 1.53) and a s-type lone pair of electrons (LP) on each of the P atoms. Both the σ- and π-component are significantly polarized towards the N-atom, with both N and C being nearly sp^2-hybridized. The LP on P3 interacts weakly with the π*-orbital of the C−N π-bond, resulting in a resonance stabilization of ca. 9 kcal/mol. The atomic charges on P1 (+0.75) and P3 (+0.55) indicate charge flow to the nitrile unit, while P2 (+0.25) shows no excess positive charge. The bonding between P1−P2 and P2−P3 is best described as minimally polarized single bonds with mainly p-character at the P atoms. The P1−N bond is significantly polarized towards the N-atom (76.08%), which is further underlined by a rather low WBI of 0.73. Para-substitution of the benzonitrile does not significantly influence the charge transfer from the triflate to the P_3CN-ring.

The main difference between **[1Tipp]$^+$ (R' = Ph)** and **2Tipp (R' = Ph)** is that the π-component of the C−N bond is less polarized towards N and that the WBI is higher compared to the triflate species (1.69 vs. 1.53). In addition, the N atom now possesses a sp^2-type LP of electrons, which is delocalized into the σ*-orbital of the P3−C bond, resulting in a stabilization energy of 16.20 kcal·mol^{-1}. The NPA charges at the atoms of the P3CN ring change only marginally.

In the following the NPA Charges (shown in bold) and Wiberg Bond indexes (shown on the end of arrows) in **[1Tipp]$^+$ (R' = Ph)** (left) and **2Tipp (R' = Ph)** (right) are summarized:

[1Tipp]$^+$ (R′ = Ph)

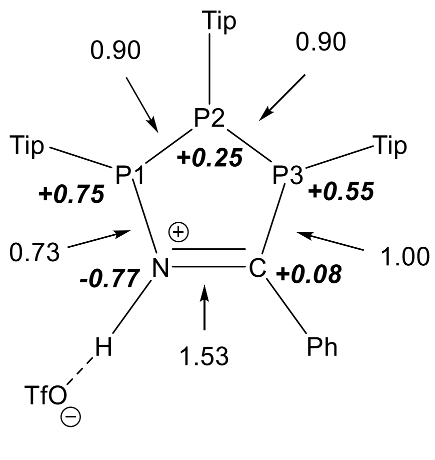

2Tipp (R′ = Ph)

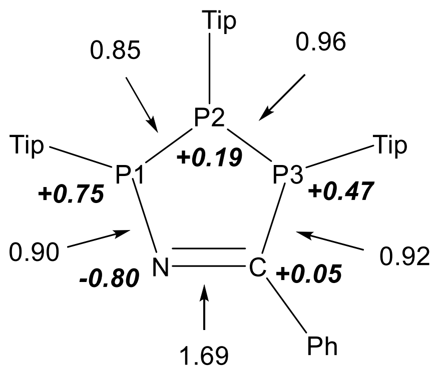

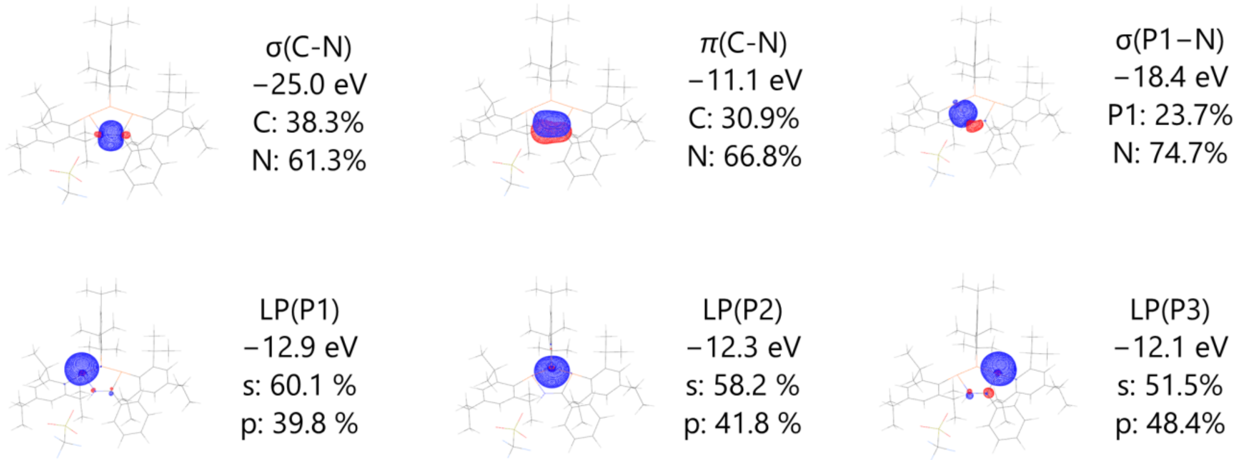

Figure S-190 Selected NLMOs of **[1$_{\mathbf{Tipp}}$]$^+$ (R' = Ph)** (BP86-D3/def2-SVP//b3lyp/cc-pVTZ)

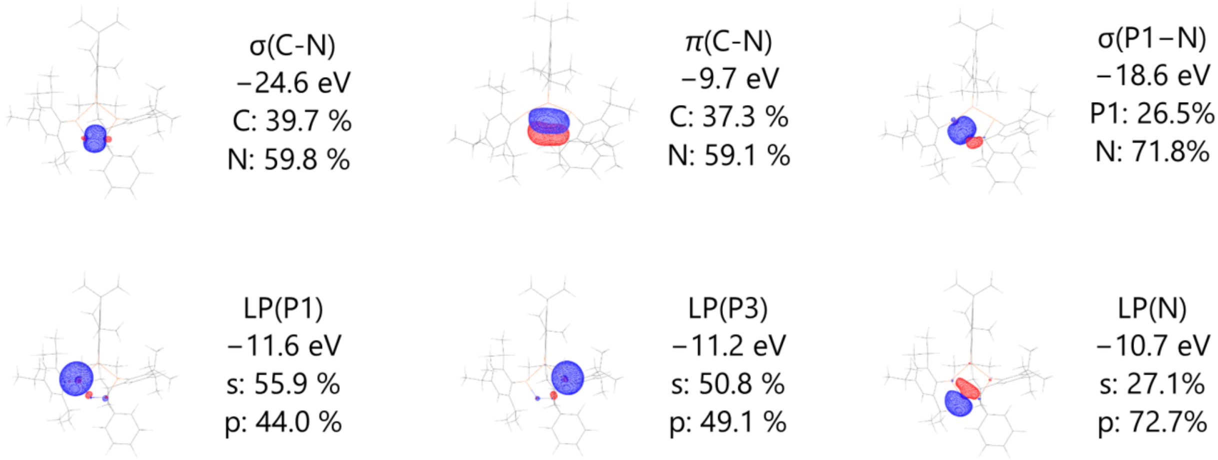

Figure S-191 Selected NLMOs of **2$_{\mathbf{Tipp}}$ (R' = Ph)** (BP86-D3/def2-SVP//b3lyp/cc-pVTZ)

6.5 Optimized Structures (.xyz files)

In addition to the electronic supporting information we provide a multi-structure xyz-file including all calculated molecules. For a better understanding and a more intuitive view of the calculated 3D structures, we strongly recommend using this file e.g. with the free program MERCURY.[21]

7. References

(1) M. Baudler, K. Glinka, A. H. Cowley, and M. Pakulski. (**1989**). Organocyclophosphanes. In *Inorganic Syntheses*, H. R. Allcock (Ed.). doi:10.1002/9780470132562.

(2) A. Schumann, F. Reiß, H. Jiao, J. Rabeah, J.-E. Siewert, I. Krummenacher, H. Braunschweig and C. Hering-Junghans, *Chem. Sci.*, 2019, **10**, 7859-7867.

(3) S. S. Chitnis, H. A. Sparkes, V. T. Annibale, N. E. Pridmore, A. M. Oliver and I. Manners, *Angew. Chem. Int. Ed.*, 2017, 56, 9536-9540.

(4) A. B. Pangborn, M. A. Giardello, R. H. Grubbs, R. K. Rosen, F. J. Timmers. Safe and Convenient Procedure for Solvent Purification. *Organometallics* 1996, **15**, 1518-1520.

(5) J. Jin, Y. Li, D. Cao, S. Wang and X. Yan, *Angew. Chem. Int. Ed.*, 2023, **62**, e202306169.

(6) Sheldrick, G. M., SHELXT - integrated space-group and crystal-structure determination. *Acta Crystallogr. A Found Adv.* 2015, **71** (Pt 1), 3-8.

(7) Sheldrick, G., Crystal structure refinement with SHELXL. *Acta Crystallogr.C Struct. Chem.* 2015, **71**, 3-8.

(8) Dolomanov, O.V.; Bourhis, L.J.; Gildea, R.J.; Howard, J.A.K.; Puschmann, H., OLEX2: A complete structure solution, refinement and analysis program (**2009**).

(9) Spek, A. L., PLATON SQUEEZE: a tool for the calculation of the disordered solvent contribution to the calculated structure factors. *Acta. Crystallogr. C Struct. Chem.* 2015, **71** (Pt 1), 9-18.

(10) Gaussian 09, Revision E.01, M. J. Frisch, G. W. Trucks, H. B. Schlegel, G. E. Scuseria, M. A. Robb, J. R. Cheeseman, G. Scalmani, V. Barone, B. Mennucci, G. A. Petersson, H. Nakatsuji, M. Caricato, X. Li, H. P. Hratchian, A. F. Izmaylov, J. Bloino, G. Zheng, J. L. Sonnenberg, M. Hada, M. Ehara, K. Toyota, R. Fukuda, J. Hasegawa, M. Ishida, T. Nakajima, Y. Honda, O. Kitao, H. Nakai, T. Vreven, J. A. Montgomery Jr., J. E. Peralta, F. Ogliaro, M. Bearpark, J. J. Heyd, E. Brothers, K. N. Kudin, V. N. Staroverov, T. Keith, R. Kobayashi, J. Normand, K. Raghavachari, A. Rendell, J. C. Burant, S. S. Iyengar, J. Tomasi, M. Cossi, N. Rega, J. M. Millam, M. Klene, J. E. Knox, J. B. Cross, V. Bakken, C. Adamo, J. Jaramillo, R. Gomperts, R. E. Stratmann, O. Yazyev, A. J. Austin, R. Cammi, C. Pomelli, J. W. Ochterski, R. L. Martin, K. Morokuma, V. G. Zakrzewski, G. A. Voth, P. Salvador, J. J. Dannenberg, S. Dapprich, A. D. Daniels, O. Farkas, J. B. Foresman, J. V. Ortiz, J. Cioslowski, D. J. Fox, Gaussian, Inc., Wallingford CT, 2013.

(11) Gaussian 16, Revision C.01, Frisch, M. J.; Trucks, G. W.; Schlegel, H. B.; Scuseria, G. E.; Robb, M. A.; Cheeseman, J. R.; Scalmani, G.; Barone, V.; Petersson, G. A.; Nakatsuji, H.; Li, X.; Caricato, M.; Marenich, A. V.; Bloino, J.; Janesko, B. G.; Gomperts, R.; Mennucci, B.; Hratchian, H. P.; Ortiz, J. V.; Izmaylov, A. F.; Sonnenberg, J. L.; Williams-Young, D.; Ding, F.; Lipparini, F.; Egidi, F.; Goings, J.; Peng, B.; Petrone, A.; Henderson, T.; Ranasinghe, D.; Zakrzewski, V. G.; Gao, J.; Rega, N.; Zheng, G.; Liang, W.; Hada, M.; Ehara, M.; Toyota, K.; Fukuda, R.; Hasegawa, J.; Ishida, M.; Nakajima, T.; Honda, Y.; Kitao, O.; Nakai, H.; Vreven, T.; Throssell, K.; Montgomery, J. A., Jr.; Peralta, J. E.; Ogliaro, F.; Bearpark, M. J.; Heyd, J. J.; Brothers, E. N.; Kudin, K. N.; Staroverov, V. N.; Keith, T. A.; Kobayashi, R.; Normand, J.; Raghavachari, K.; Rendell, A. P.; Burant, J. C.; Iyengar, S. S.; Tomasi, J.; Cossi, M.; Millam, J. M.; Klene, M.; Adamo, C.; Cammi, R.; Ochterski, J. W.; Martin, R. L.; Morokuma, K.; Farkas, O.; Foresman, J. B.; Fox, D. J. Gaussian, Inc., Wallingford CT, 2016

(12) a) E. D. Glendening, E. D.; Badenhoop, J. K.; Reed, A. E.; Carpenter, J. E.; Bohmann, J. A.; Morales, C. M.; Landis, C. R.; Weinhold, F., NBO 6.0, Theoretical Chemistry Institute, University of Wisconsin, Madison, 2013; b) Carpenter, J. E., Weinhold, F., J. Mol. Struct.: THEOCHEM 1988, 169, 41–62; c) Weinhold, F.; Carpenter, J. E., The Structure of Small Molecules and Ions, Plenum Press, 1988; d) Weinhold, F.; Landis, C. R., Valency and Bonding. A Natural Bond Orbital Donor-Acceptor Perspective, Cambridge University Press, 2005.

(13) a) Perdew, J. P.; Burke, K.; Ernzerhof, M., *Phys. Rev. Lett.* 1996, **77**, 3865–3868; b) Perdew, J. P.; Burke, K.; Ernzerhof, M., *Phys. Rev. Lett.* 1997, **78**, 1396–1396; c) Adamo, C.; Barone, V., *J. Chem. Phys.* 1999, **110**, 6158–6170.

(14) a) Grimme, S.; Antony, J.; Ehrlich, S.; Krieg, H., *J. Chem. Phys.* 2010, **132**, 154104; b) Grimme, S.; Ehrlich, S.; Goerigk, L., *J. Comput. Chem.* 2011, **32**, 1456–1465.

(15) Weigend, F.; Ahlrichs, R., *Phys. Chem. Chem. Phys.* 2005, **7**, 3297–3305.

(16) a) A. D. Becke, *Phys. Rev. A* 1988, **38**, 3098-3100; b) B. Miehlich, A. Savin, H. Stoll, H. Preuss, *Chem. Phys. Lett.* 1989, **157**, 200-206; c) C. Lee, W. Yang, and R. G. Parr, *Phys. Rev. B* 1988, **37**, 785-789.

(17) a) T. H. Dunning Jr., *J. Chem. Phys.* 1989, **90**, 1007-1023; b) R. A. Kendall, T. H. Dunning Jr., R. J. Harrison, *J. Chem. Phys.* 1992, **96**, 6796-6780; c) D. E. Woon, T. H. Dunning Jr., *J. Chem. Phys.* 1993, **98**, 1358-1371; d) K. A. Peterson, D. E. Woon, T. H. Dunning Jr., *J. Chem. Phys.*, 1994, **100,** 7410-7415; e) A. K. Wilson, T. van Mourik, T. H. Dunning Jr., *J. Mol. Struct. (Theochem)* 1996, **388**, 339-349.

(18) A. V. Marenich, C. J. Cramer, and D. G. Truhlar, *J. Phys. Chem. B*, **2009**, *113*, 6378-96.

(19) Lu, T.; Chen, F., *J. Comput. Chem.* 2012, **33**, 580.

(20) GaussView, Version 6.1, Roy Dennington, Todd A. Keith and John M. Millam, Semichem Inc., Shawnee Mission, KS, 2016.

(21) Mercury: http://www.ccdc.cam.ac.uk/mercury/

(22) W. Humphrey, A. Dalke, and K. Schulten, *J. Molec. Graphics* 1996, **14**, 33-38
